# Metabolic profiles of lung adenocarcinoma via peripheral blood and diagnostic model construction

**DOI:** 10.1038/s41598-023-34575-0

**Published:** 2023-05-05

**Authors:** Kyung Soo Kim, Seok Whan Moon, Mi Hyung Moon, Kwan Yong Hyun, Seung Joon Kim, Young Koon Kim, Kwang Youl Kim, Dong Wook Jekarl, Eun-Jee Oh, Yonggoo Kim

**Affiliations:** 1 Department of Thoracic and Cardiovascular Surgery, Seoul St. Mary’s Hospital, College of Medicine, The Catholic University of Korea, Seoul, Republic of Korea; 2 Department of Pulmonology, Seoul St. Mary’s Hospital, College of Medicine, The Catholic University of Korea, Seoul, Republic of Korea; 3 Department of Clinical Pharmacology, Inha University Hospital, Inha University, 27 Inhang-ro, Jung-gu, Incheon, 22332 Republic of Korea; 4 Department of Laboratory Medicine, Seoul St. Mary’s Hospital, College of Medicine, The Catholic University of Korea, 222, Banpo-Daero, Seocho-gu, Seoul, 06591 Republic of Korea; 5 Research and Development Institute for In Vitro Diagnostic Medical Devices, College of Medicine, The Catholic University of Korea, Seoul, Republic of Korea

**Keywords:** Cancer, Cancer metabolism, Cancer screening, Lung cancer

## Abstract

The metabolic profile of cancerous cells is shifted to meet the cellular demand required for proliferation and growth. Here we show the features of cancer metabolic profiles using peripheral blood of healthy control subjects (n = 78) and lung adenocarcinoma (LUAD) patients (n = 64). Among 121 detected metabolites, diagnosis of LUAD is based on arginine, lysophosphatidylcholine-acyl (Lyso.PC.a) C16:0, and PC-diacyl (PC.aa) C38:3. Network analysis revealed that network heterogeneity, diameter, and shortest path were decreased in LUAD. On the contrary, these parameters were increased in advanced-stage compared to early-stage LUAD. Clustering coefficient, network density, and average degree were increased in LUAD compared to the healthy control, whereas these topologic parameters were decreased in advanced-stage compared to early-stage LUAD. Public LUAD data verified that the genes encoding enzymes for arginine (*NOS, ARG, AZIN*) and for Lyso.PC and PC (*CHK, PCYT, LPCAT*) were related with overall survival. Further studies are required to verify these results with larger samples and other histologic types of lung cancer.

## Introduction

Lung cancer is one of the malignancies with the highest mortality and is second in incidence and fourth in prevalence worldwide. The global mortality and incidence of lung cancer were 18.0 and 22.4 per 100,000, respectively, in 2020^1^. Around 230,000 patients are newly diagnosed with lung cancer annually, and 130,000 died from lung cancer in the United States in 2021^2–4^. Although deaths per 100,000 are decreasing, lung cancer shows the highest death rate among all cancers^2–4^.

Metabolomics has supported genomics and proteomics to provide information regarding cancer metabolic processes that are related to tumor formation and/or proliferative potential^5,6^. These biomarker metabolites can enhance our understanding of tumor biology and have potential for applied use in early diagnosis. Among them, acylcarnithines, amino acids, biogenic amines, lysophosphatidylcholines (LPCs), phosphatidylcholine (PC), and sphingolipids can be extracted from tissue, blood, serum, or body fluids. Arginine is an amino acid that is used for creatine and urea biosynthesis. In the kidney, proline, glutamate, and glutamine can be converted to citrulline and with aspartate can be synthesized to agininosuccinate and then to arginine by agininosuccinate lyase via the urea cycle^7–9^. Arginine is required for tumor cell proliferation; however, it is also required for normal lymphocytes, which could act as a double-edged sword in cancer treatment^7^.

LPC and PC are precursors of membrane lipids related with plasma membrane remodeling in various cancers^10^. Lung cancer cells dysregulate lipid metabolism, the effects of which are reflected in the peripheral blood^8,9^. LPC is a lipid biomolecule derived from PC by phospholipase A2 or from free cholesterol and fatty acids by lecithin cholesterol acyltransferase^11,12^. Cancer cells convert LPC to produce various PCs via LPC acyltransferase 1 (LPCAT1), and the PCs are utilized for plasma membrane remodeling^13^. Decreased LPC and increased PC concentration are found in cancer tissue and in the peripheral blood of cancer patients^13,14^.

To study the metabolic nature of lung cancer using peripheral blood, metabolites from healthy control and LUAD patient samples were compared. A statistical model was constructed based on these metabolites for early diagnosis of LUAD. Network analysis was performed to analyze these biomarkers among healthy controls and early and advanced LUAD patients.

## Materials and methods

### Patients and controls

This study was approved by the Institutional Review Board of Seoul St. Mary’s Hospital. After informed consent, samples were collected from Seoul St. Mary’s Hospital Biobank, The Catholic University of Korea, Seoul, Republic of Korea. All the methods were performed in accordance with the relevant guidelines and regulations. Plasma samples from the peripheral blood of healthy patients (n = 80) and LUAD cancer patients (n = 65) were analyzed in the study. All LUAD patients underwent surgical intervention, and the diagnosis was confirmed by a pathologist during routine diagnostic procedures^15,16^. All patients were classified according to the World Health Organization definitions and according to the seventh edition of the tumor-node-metastasis (TNM) classification of the American Joint Committee on Cancer Staging Manual^17^. The TNM stages of the LUAD patients are presented (Supplementary Table 1). Blood samples from LUAD patients were collected on the day of surgery. The healthy control samples were those from routine health checkup patients. After measurements, the samples that passed quality control (QC) included 64 and 78 samples for healthy and lung patient samples, respectively.

### Targeted metabolomic analysis and sample preparation

The AbsoluteIDQ^®^ p180 (Biocrates Life Sciences AG, Innsbruck, Austria) kit is a MS-based assay for quantification of target metabolites in body fluids using stable isotope–labeled standards. The kit allows targeted analysis of 188 metabolites, covering a wide range of analytes and metabolic pathways in one targeted assay. Amino acids (21) and biogenic amines (21) are determined in the LC–MS mode; acylcarnitines (40), LPCs (14), PCs (76), sphingomyelins (15), and the sum of hexoses (1) were analyzed via flow injection analysis (FIA; Supplementary Table 2).

Metabolite measurement and sample preparation from a total of 145 plasma samples from healthy and LUAD patients were carried out according to the manufacturer’s protocol. Briefly, 10 μL of internal standard (amino acids and biogenic amines) followed by 10 μL of sample (calibrator, QC, and plasma sample) were added to the filter inserts (which contained several internal standards corresponding to glycerophospholipids, sphingomyelins, acylcarnitine, and hexoses) of the 96-well plate and dried for 30 min under a nitrogen stream. Then, 50 μL of a 5% phenylisothiocyanate (PITC) solution was added to derivatize amino acids and biogenic amines and then incubated at room temperature for 1 h. After incubation, the metabolites and internal standards were extracted using 5 mM ammonium acetate in methanol (300 µL) into the lower 96-well plate for MS analysis. One-half of the eluate was measured by liquid chromatography (LC) LC–MS/MS, and the other half was determined by FIA-MS/MS.

### Liquid chromatography and mass spectrometry

Analyses for amino acids and biogenic amines were performed using a Zorbax Eclipse XDBC18 column (3 mm × 100 mm, 3.5 µm, Agilent Technologies) at a flow rate of 0.5 ml/min with water:acetonitrile containing 0.2% formic acid in the LC gradient condition. Analyses for glycerophospholipids, sphingolipids, acylcarnitines, and hexoses were performed at a flow rate of 0.03 ml/min with elution solvent in FIA gradient conditions. Multiple reaction monitoring modes of the tandem mass spectrometer (API 4000, AB Sciex) in positive electrospray ionization (ESI) were used for quantification of target metabolites. However, only hexose was analyzed in negative ESI mode.

### Data management and analysis

Quantification was carried out using internal standards and a calibration curve^18^. Briefly, after pre-processing (peak integration and concentration determination from calibration curves) with Analyst 1.5.1 software (AB Sciex, Darmstadt, Germany), data were uploaded into Biocrates MetIDQ software. The concentrations of metabolites determined by FIA were directly calculated in MetIDQ. Specifically, the concentrations of derivative metabolites consisting of amino acids and biogenic amines were quantified precisely with a seven-point calibration curve and internal standards, while the remaining metabolites corresponding to glycerophospholipids, sphingolipids, acylcarnitines, and hexoses were calculated semi-quantitatively based on specific stable isotope internal standard intensities. The final metabolite concentration was determined with inter- and intra-plate normalization using the median of three QC2 (from Biocrates Inc.) replicate injections of each plate compared to the target value.

### Network analysis

Network topologic parameters were defined as follows^19–25^. Node (*N*) indicates each metabolite, and link (*L*) indicates correlation between the nodes and plotted by a line. The average clustering coefficient (<*C*>) was defined as the friend’s friend, which was calculated as follows: *C*_*i*_ = 2*L*_i_/*k*_*i*_(*k*_*i*_-1), where *C*_*i*_ represents the clustering coefficient, *L*_i_ represents the number of lines between neighbors of *k*_i_, and *k*_i_ represents the node *i* with degree *k*_i_. Finally, <*C*>  = 1/N $\sum_{i=1}^{N}Ci$. If *Ci* = 0, none of the node *i* neighbors are linked. If *Ci* = 0.5, there is a 50% chance that the node *i* neighbors are linked. Network density was calculated as follows: density (*d*) = *L*/*L*_max_, where *L* indicates the number of links, and *L*_max_ indicates the maximum probable links among the nodes. Increased density implies that there are more correlations between molecules. Network heterogeneity was calculated as follows: Heterogeneity  =  √variance(*k*)/mean(*k)*, which implies that network heterogeneity is variance of the degree of a node divided by the mean degree number. Network centralization (degree centralization) shows the importance of certain nodes with high connection (degree) compared to other nodes, which was calculated as follows: $C(j)=\sum_{j=1}^{n}A_{ij}$, where *A* indicates adjacency matrix and n indicates the total number of nodes. Shortest path or average shortest path was calculated as follows: <*l*> = $2{\sum }_{ij}lij$/*N*(*N*-1), where $l$ indicates the shortest path between two nodes (*i*, *j*). Average degree (<*k*>) was calculated as follows: <*k*>  = 2*L*/*N* = d*N*(*N*-1)/*N*. These calculations are performed using a Netanalyzer that was attached to Cytoscape^26–28^. Assortativity and modularity were calculated using the igraph package^29^. Assortativity coefficient indicates the association of nodes with similar weights. Modularity indicates the tendency of nodes to cluster and to form groups or clusters^19,20^.

### Public dataset analysis

To verify the tested results in this study, the LUAD dataset was used. The Genomic Data Commons (GDC) The Cancer Genome Atlas (TCGA) LUAD dataset from the National Cancer Institute was downloaded from the University of Santa Cruz (UCSC) Xena Browser platform (https://xenabrowser.net)^30,31^. Primary tumors were selected, followed by deletion of duplicate patient samples. Survival analysis of these expression datasets was conducted via the Kaplan Meier method using the R package. The arginine biosynthesis pathway was plotted with modification based on the Kyoto Encyclopedia of Genes and Genomes (KEGG) pathway (hsa00220) and on the work of Morris et al.^31,32^. The PC and LPC pathways were plotted with modification based on the respective KEGG pathway (hsa00564) and on the work of Lesko et al.^31–33^. Arginine, citrulline, ornithine, PC, and LPC concentrations from this study were interpolated using these pathways.

### Statistical analysis

Significance among the mean values for healthy and LUAD samples was measured using Mann Whitney U tests with unequal variance and Student’s *t*-tests for equal variance. Many of the metabolites demonstrated collinearity; therefore, the least absolute shrinkage and selection operator (LASSO) regression analysis was performed for selecting diagnostic factors using the glmnet R package^34,35^. To select variables, multivariate logistic regression analysis was performed using the glm R package^36^. In addition, network analysis was performed based on the correlation matrix by Pearson’s correlation analysis. Correlation coefficient and *p*-values were calculated using the Hmisc R package^37^. Correlation coefficients greater than or equal to 0.8 with statistical significance were selected. Unweighted, undirected networks were constructed with these selected nodes, and network topological studies were plotted using Cytoscape^26–28^. From public datasets, Kaplan Meier analysis was performed using the survival^38^ R package and plotted using the survminer R package^39–41^. For receiver operating characteristics curve analysis, the pROC R package was used^42^. Data were analyzed using dplyr and plotted using ggplot2^41^. All the statistical analysis was performed using the R program^43^.

## Results

### Measured metabolites

**Figure 1:**
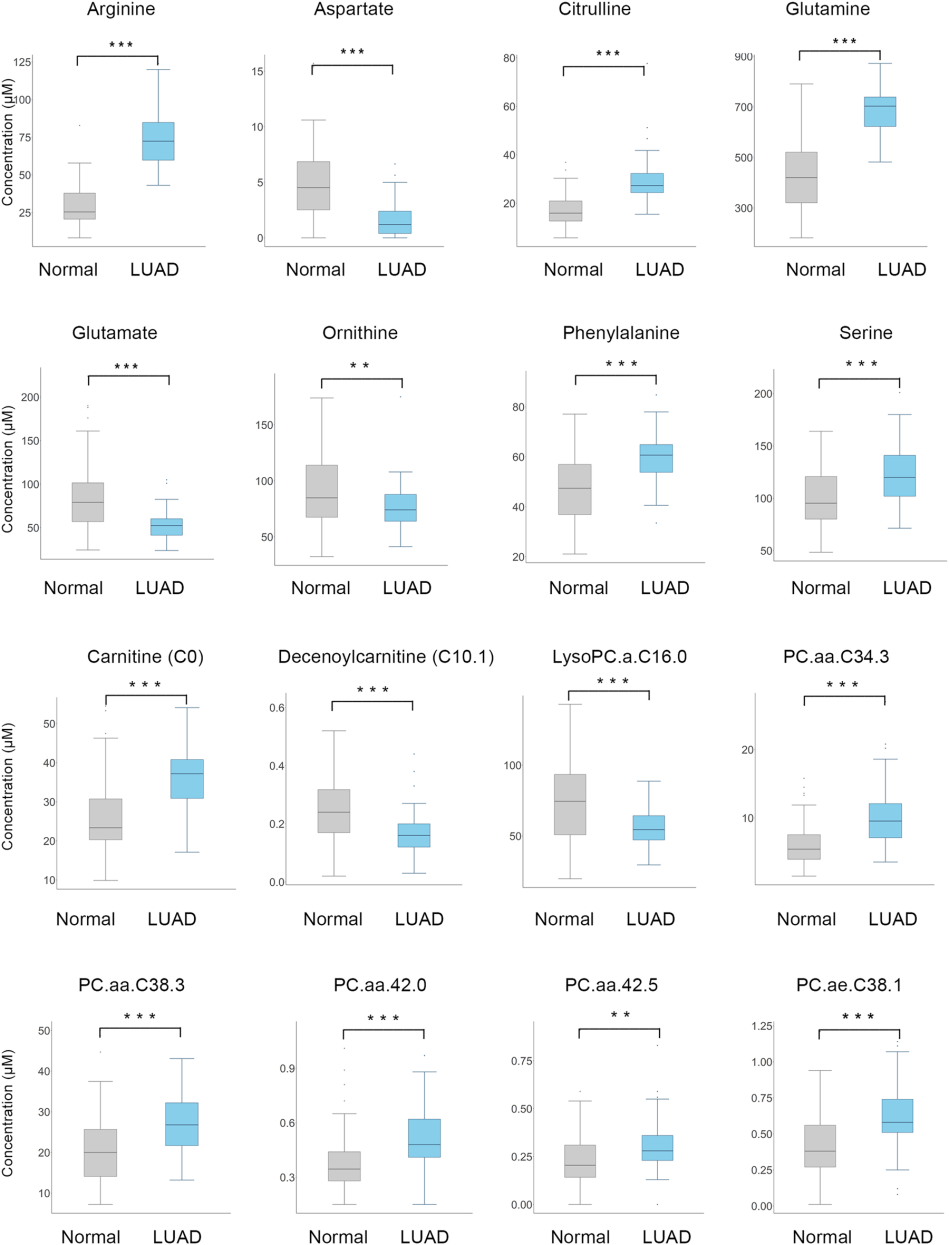
Measured metabolite concentrations after LASSO analysis. ***p < 0.001, **0.001 ≤ *p* < 0.01.

Among the 188 targeted metabolites, 121 including acyl carnitines, amino acids, biogenic amines, glycerophospholipids, and sphingolipids were measured and detected. The mean and standard deviation of these metabolites are listed in Fig. 1 (Supplementary Table 3). Most of the metabolites showed statistical significance, which implies a metabolic difference between the healthy control and LUAD patients. Among the 64 LUAD patients, 44 were diagnosed with early LUAD, whereas 20 were diagnosed with advanced LUAD (Supplementary Table 4). Among the measured metabolites, PC and LPC were decreased in advanced LUAD compared to early LUAD. TNM stage and overall survival analysis revealed that early-stage LUAD patients, which included T1 or T2 status, showed 97.7% overall survival. On the contrary, advanced LUAD, which included half of T2 and most of T3 and T4 status and stage 2 or greater, showed relatively unfavorable overall survival (Supplementary Table 5).

### Regression and analysis

As there were many variables and some showed collinearity, LASSO regression was performed, and independent variables related to LUAD diagnosis were investigated (Table 1). Optimal lambda parameter was screened in the LASSO model using the minimum criterion for tenfold cross validation. The left and right dashed lines indicate lambda and standard error of 1ambda values of the minimized mean square error (Supplementary Fig. 1A). LASSO regression penalizes variables and converges values of unimportant variables to zero, which was plotted in variable profiles (Supplementary Fig. 1B). LASSO regression analysis revealed that the tenfold cross-validated lambda was 0.0158. The regression analysis resulted in 18 of 121 variables that showed statistical significance, including C0, C10:1, arginine (Arg), aspartate (Asp), citrulline (Cit), glutamine (Gln), glutamate (Glu), ornithine (Orn), phenylalanine (Phe), serine (Ser), total DMA (dimethylarginine), lysophosphatidylcholine (lyso.PC) acyl C16:0 (lyso.PC.a.C:16), phosphatidylcholine acyl-alkyl (PC.aa) C34:3 (PC.aa.C34:4), PC.aa.C38:3, PC.aa.C40:5, PC.aa.C42:0, PC.aa.C42:5, and PC.aa.C38:1 (Table 1). To construct a diagnostic model, multivariate logistic regression analysis was performed using the backward stepwise method among these variables (Table 1). As some variables showed collinearity, the variable with the highest OR was eliminated. Finally, arginine, lyso.PC.a.C16:0, and PC.aa.C38:3 were included in the model.

**Table 1:** Predictive model screened by least absolute shrinkage and selection operator (LASSO) regression analysis followed by multivariate logistic regression analysis.

	LASSO regression coefficient	Multivariate logistic regression OR (odds ratio)	95% CI
C0	0.0014		
C10:1	− 0.8617		
Arg	0.0044	1.373	(1.083–1.740)
Asp	− 0.0222		
Cit	0.0029		
Gln	0.0007		
Glu	− 0.00003		
Orn	− 0.0011		
Phe	0.0018		
Ser	0.001		
total.DMA	0.0847		
lysoPC.a.C16:0	− 0.005	0.749	(0.602–0.931)
PC.aa.C34:3	0.0044		
PC.aa.C38:3	0.0039	1.509	(1.010–2.254)
PC.aa.C40:5	0.0135		
PC.aa.C42:0	0.0359		
PC.aa.C42:5	0.0299		
PC.ae.C38:1	0.0022		

Only variables with non zero coefficients are shown for LASSO regression analysis.

C0, carnithine; C10:1, acycarnithine C10:1; LysoPC.a.C16.0, lysophosphatidylcholine-acyl (LysoPC.a) C16:0; PC.aa, diacyl phosphatidylcholine; PC.ae, acyl-alkyl phosphatidylcholine.

### ROC curve analysis

ROC curve analysis was performed for all variables in LUAD diagnosis. Area under the receiver operation curve (AUROC), cut off values, sensitivity, specificity, positive predictive value (ppv), and negative predictive value (npv) were calculated (Supplementary Table 6). Amino acids showed higher AUROC followed by acylamines and PC for diagnosis of LUAD. LPCs showed relatively lower AUROC compared to other metabolites. Arg, Gln, Cit, and Asn showed the highest AUROCs of 0.981, 0.942, 0.915, and 0.854, respectively. For metabolites selected by regression analysis, arginine, lyso.PC.a.C16:0, and PC.aa.C38:3 resulted in AUROCs of 0.981, 0.697, and 0.715, respectively (Supplementary Fig. 2).

### Network analysis

**Figure 2:**
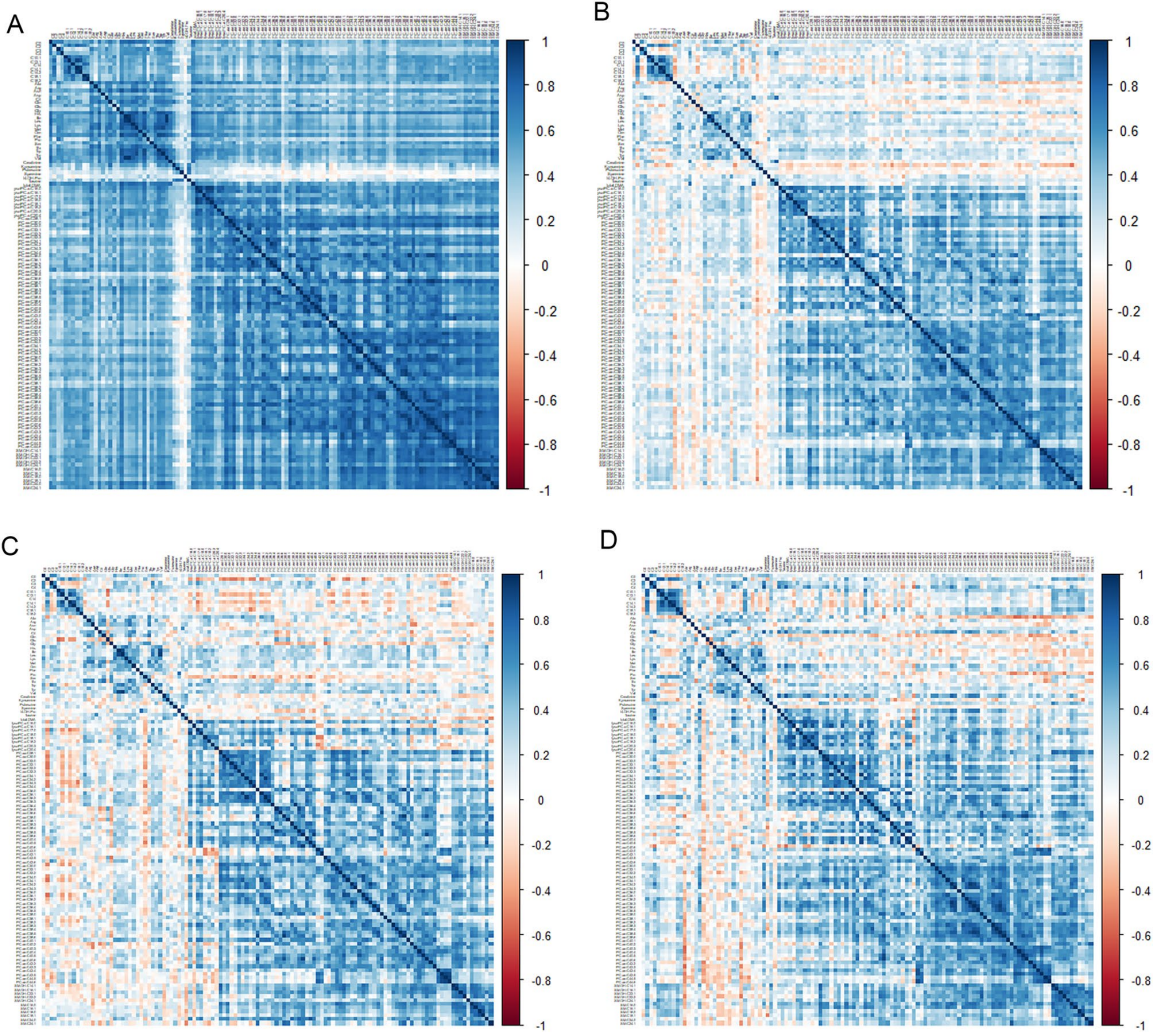
Correlation matrix of the healthy control group (**A**) and the LUAD group (**B**). The healthy controls showed correlations among tested metabolites compared to the LUAD group. Correlation matrix of early LUAD (stage I) (**C**) and advanced LUAD (stage II–IV) (**D**).

**Figure 3:**
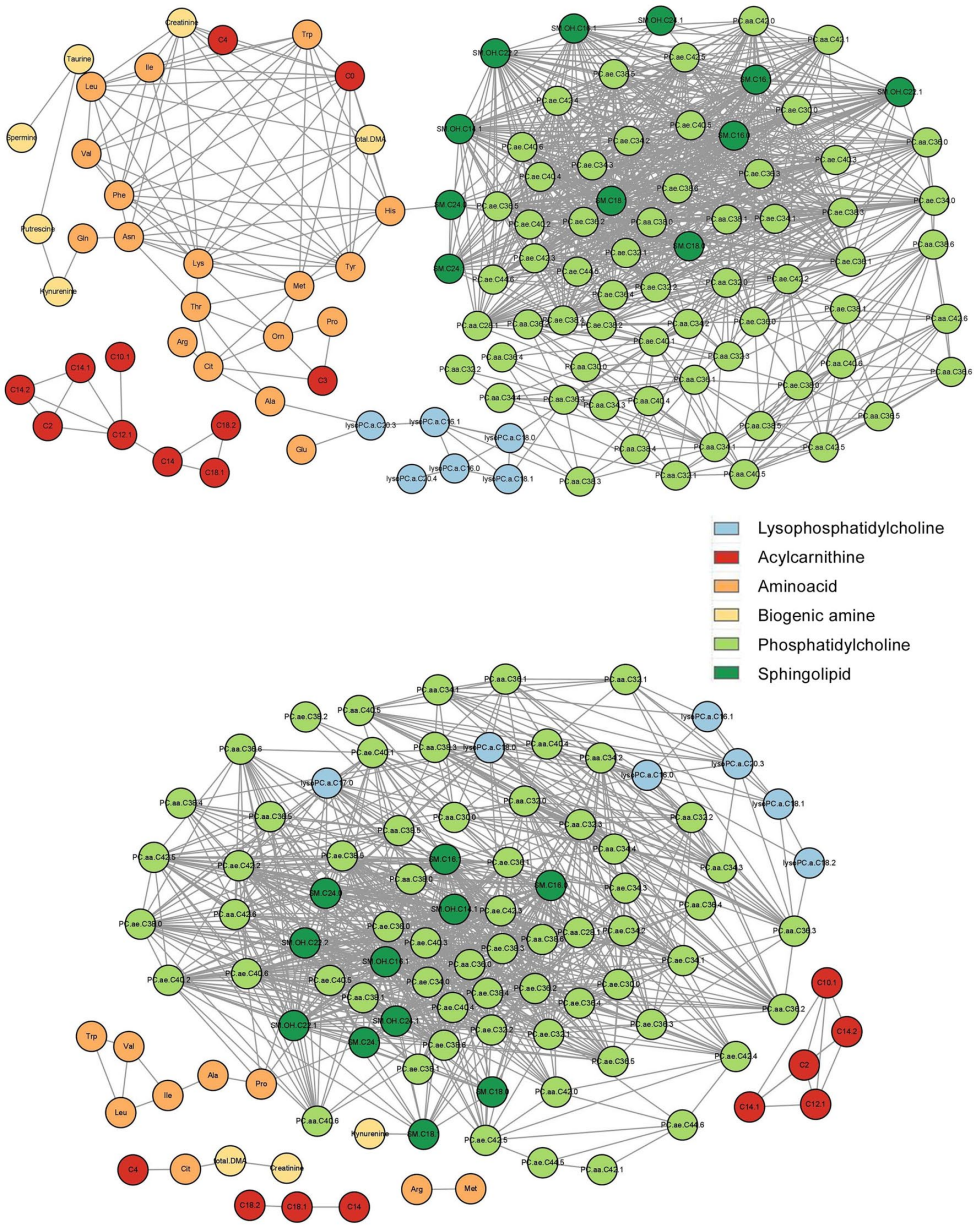
Metabolic network constructed based on the correlation matrix of healthy control (**A**) and LUAD (**B**) groups using peripheral blood.

An unweighted, undirected network was constructed based on the Pearson’s correlation matrix from the healthy control and LUAD groups (Fig. 2). Among them, a correlation coefficient greater than or equal to 0.8 was selected. Network construction was performed for the healthy controls and resulted in parameters related to nodes and links (Fig. 3A,B). The hub node was PC.ae.C34.1 with 41 degrees, followed by SM.C16.1 with 40 degrees and PC.ae.C38.3 with 39 degrees (Supplementary Tables 7, 8). For LUAD, the hub node was PC.ae.38.3 with 43 degrees, followed by PC.ae.C36.1, PC.ae.C42.3, and PC.ae.C42.2 all with 40 degrees (Supplementary Tables 9, 10, Fig. 3B). Network topologic parameters revealed slightly more nodes and links in the healthy control group compared to the LUAD group (Table 2). Clustering coefficient and network density were increased in the LUAD network, which implies correlation between molecules. However, network heterogeneity was lower, which denotes a smaller variance of degree in the cancer network. Diameter or shortest path of the LUAD network was shorter than that of the healthy control, which indicates that metabolites are more correlated. Average degree was higher in the LUAD network, which implies correlated metabolites.

**Table 2:** Network topologic parameters from healthy control (n = 78) and LUAD (n = 64) groups.

	Healthy normal (n = 78)	LUAD (n = 64)	Early stage (n = 27)	Advanced stage (n = 27)
Number of nodes, N	114	101	98	102
Number of links, L	932	898	550	427
Clustering coefficient, <*C*>	0.634	0.669	0.566	0.548
Network density	0.165	0.236	0.154	0.094
Network heterogeneity	0.702	0.565	0.546	0.573
Connected components	2	5	5	2
Network diameter	11	9	11	9
Network centralization	0.229	0.27	0.163	0.157
Shortest path	3.445	2.477	3.143	3.27
Average degree, <*k*>	17.377	20.276	12.786	8.723
Assortativity	− 0.5819	− 0.6566	0.0959	0.0579
Modularity	0.9824	0.9828	0.0545	0.0646
Node with highest degree (hub)	PC.ae.C34.1	PC.ae.C38.3	PC.ae.C38.3 PC.ae.C42.3	PC.ae.C38.6

Network topologic parameters from randomly selected cases (n = 27) among early-stage (n = 52) and advanced-stage (n = 27) LUAD samples.

**Figure 4:**
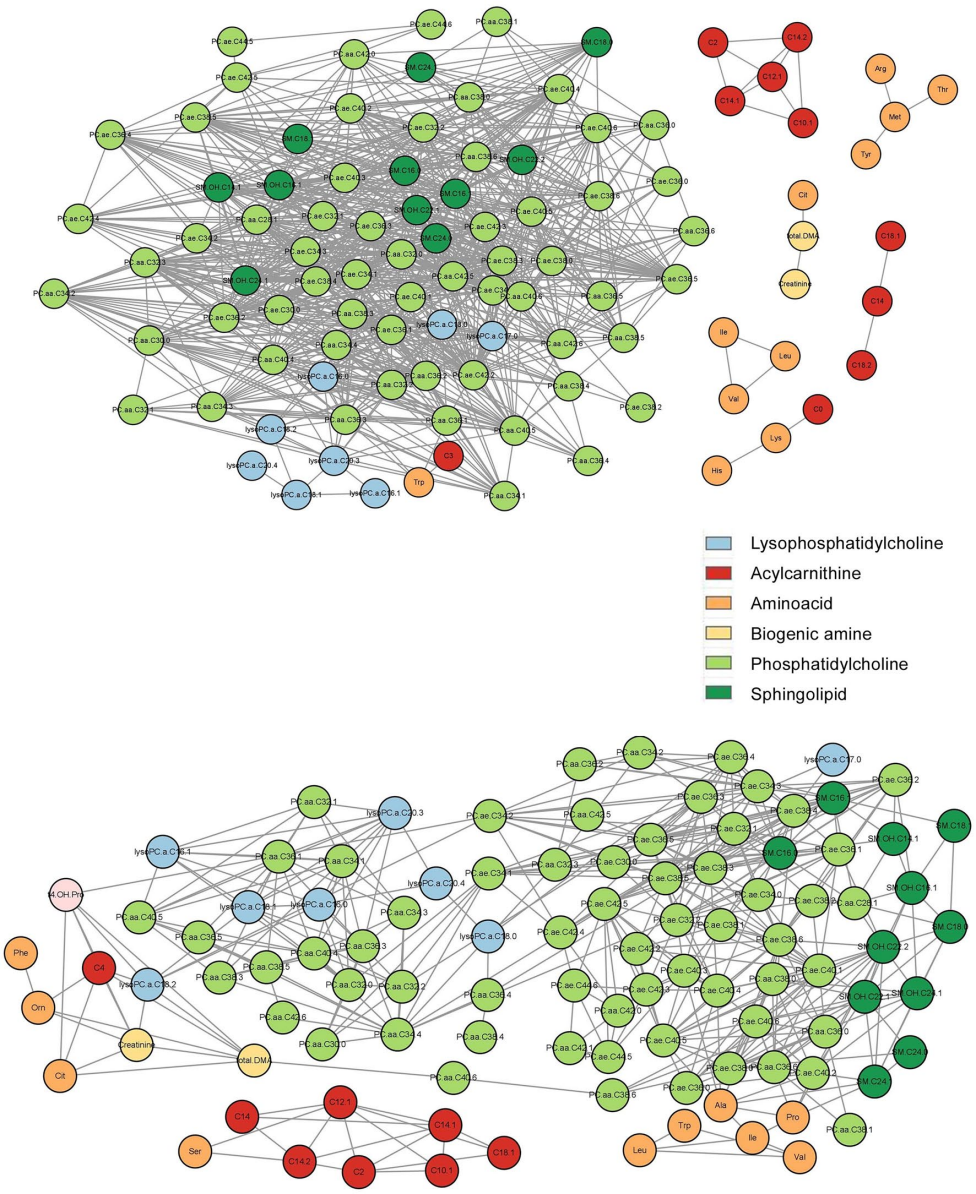
Metabolic network constructed based on the correlation matrix of early LUAD stage (I) (**A**) and advanced LUAD (stage II–IV) (**B**) groups using peripheral blood.

Early and advanced LUAD also were analyzed. For early LUAD, the hub nodes were PC.ae.C38.3 and PC.ae.C42.3 with 45 degrees (Supplementary Tables 11, 12). For advanced LUAD, the hub node was PC.ae.C38.6 with 23 degrees, followed by PC.ae.C38.5 and PC.ae.C32.1 with 21 degrees (Supplementary Tables 13, 14). Network parameters revealed decreased clustering coefficient, network density, and average degree in advanced-stage LUAD (Fig. 4A,B). However, network heterogeneity and shortest path were increased in advanced-stage LUAD, which implies a weaker correlation between molecules than in early-stage LUAD (Table 2).

### Public data analysis

**Figure 5:**
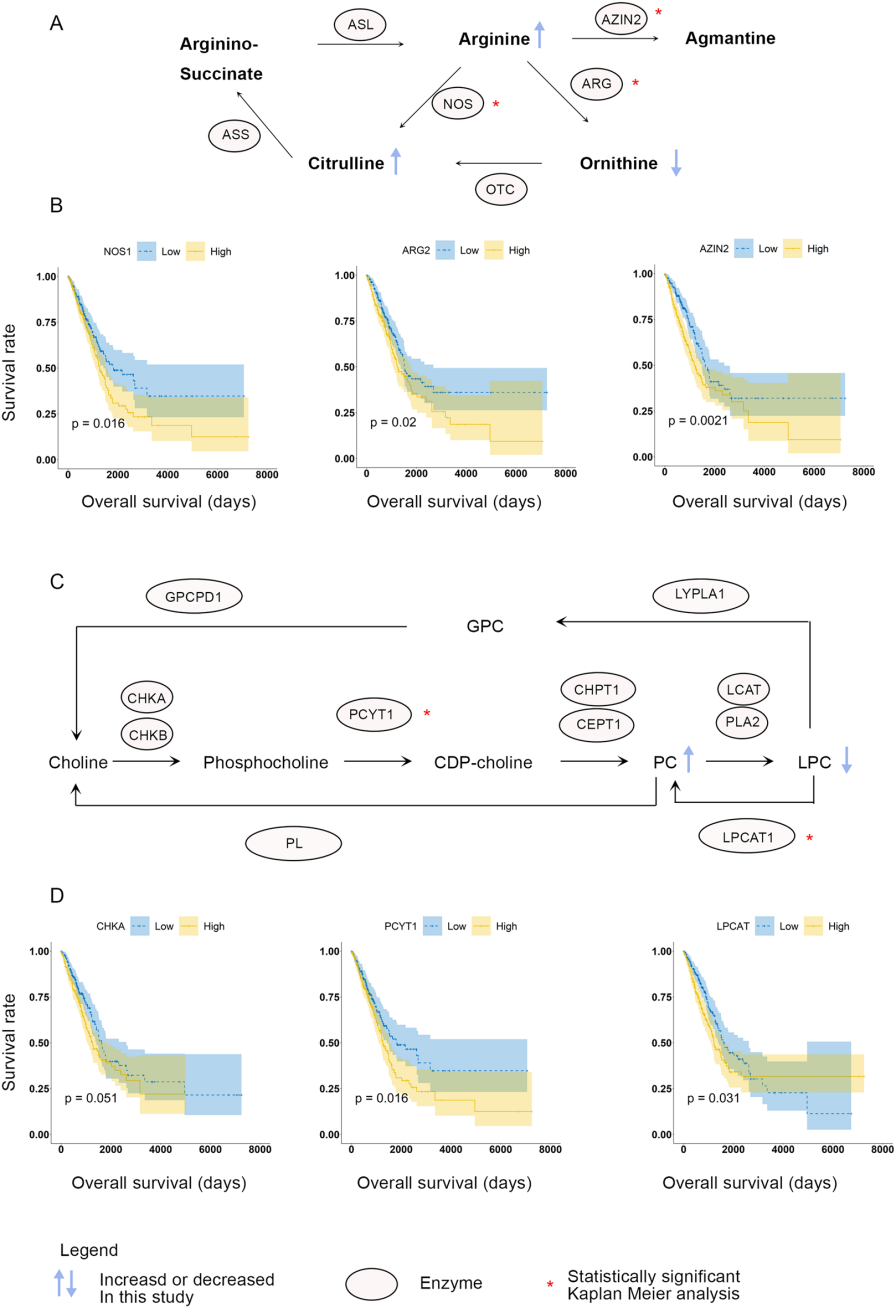
Arginine and phospholipid biosynthesis pathways with overlaid study data. Arginine pathways are plotted (**A**). The PC and LPC pathway is plotted (**B**). Kaplan Meier analysis of GCD TCGA LUAD data was downloaded from Xena Browser.

From Xena Browser, 877 samples from the GDC TCGA LUAD were downloaded. Among them, 597 primary tumor samples were selected, of which 73 were deleted due to null data. A total of 511 samples with survival data was selected; after deletion of 11 duplicate samples, 500 unique patient samples were analyzed via Kaplan Meier analysis. Arginine and the PC and LPC pathways were plotted with modifications^28–30^, and concentration data from this study were interpolated. Arginine, citrulline, and PC levels were increased in cancer patients, whereas lysoPC was decreased. Gene expression was also selected for Kaplan Meier analysis (Fig. 5), and the results showed that *AZIN2, NOS1,* and *ARG* that encode enzymes related to arginine biosynthesis were significantly upregulated in the LUAD group. Low gene expression was related with favorable overall survival rate. Arginine and citrulline were increased and ornithine was decreased in LUAD in this study. *NOS, ARG,* and *AZIN2* are expected to be decreased in LUAD, which would increase arginine level. For the PC and LPC pathways, *LPCAT* and *PCYT1* were significantly upregulated in the Kaplan Meier analysis (Supplementary Fig. 3).

## Discussion

Treatment of lung cancer could benefit from early diagnosis followed by surgical and pharmaceutical treatment. Early diagnosis has been achieved using low-dose computed tomography (CT) and was shown to decrease mortality by 20% compared to a control group^3,4^. Screening by low-dose CT is the only approved method for early diagnosis, but this can be a burden to the patient undergoing radiological testing. Thus, diagnosis of cancer using blood biomarkers might be more suitable.

Metabolic profiles could provide valuable information for early LUAD diagnosis. Metabolic shifts are noted in cancer cells and are a part of the metabolic adaptation required to meet high demand for energy production required for proliferation in cancer cells^44^. Most of the metabolic profiles showed differences between healthy controls and LUAD samples. Among the tested 188 metabolites, three were selected for early LUAD diagnosis, arginine, Lyso.PC.a.C16:0, and PC.aa.C38:3.

Arginine is required for biological systems including cell growth and immune response modulation. Arginine deprivation is related to caspase-dependent apoptosis, caspase-independent apoptosis, caspase-independent autophagic cell death, and necroptosis in various tumors^6^. As regulatory T cell or myeloid-derived suppressor cells suppress the immune response and metabolize arginine through arginase-I, arginine supplementation boosted chemotherapy efficacy^45,46^. As arginine is a precursor of nitric oxide (NO), NO could have anti-tumor effects due to p53 accumulation, which could be a possible mechanism in LUAD^45,46^. In this study, arginine was increased in the plasma of LUAD patients. Increased arginine might trigger the formation of NO that could result in vascular proliferation and tumor growth^47,48^. As increased NO expression is related with unfavorable prognosis, increased arginine could also be related to unfavorable prognosis, although this requires further study.

Unexpectedly, Lyso.PC.a.C16:0 was decreased in LUAD samples. LPC is decreased in cells due to the activity of LPCAT, which is an LPC acyltransferase that catalyzes the conversion of LPC to PC. Previously, LPCAT has been reported to be increased in cancer patients; therefore, LPC might be decreased in cancer patients^49–51^. In this study, except for one LPC or lysoPC.a.C28:1, every LPC was decreased in LUAD samples. Furthermore, Lyso.PC.a.C16:0 was included in the prognostic model, implying that LPC could be a crucial factor affecting cancer pathobiology^49–51^. Normally, LPCs are components of surfactants made by alveolar type II cells in the lung, and decreased LPC in lung cancer patients implies either consumption by cancer cells or conversion by LPCAT. PCs are used for membrane construction, and PC metabolism is reprogrammed for proliferation in cancer cells^30,52^. In this study, most of the measured PCs were increased in LUAD patients, and PC.aa.C38:3 was increased in the diagnostic model.

Network analysis can analyze complex or multi-dimensional data without losing or omitting many data points^19,25,53^. Network heterogeneity reflects the degree distribution of nodes, and increased heterogeneity implies the presence of a hub node. Biological genetic networks tend to be heterogeneous due to the presence of a hub gene that is related to many other genes. In this study, heterogeneity was lower in LUAD compared to the normal healthy control group, indicating decreased presence of a hub node. The small world property involves shorter paths between nodes and small parameter diameter. Shorter paths between nodes implies a fast response against external stimuli and adaptation to the external environment^51,53^, as observed in LUAD. Assortativity indicates relationships between hub and hub nodes, whereas dissortativity implies relationship between hub and non-hub nodes. In this study, there was no significant difference between the groups in assortativity. Modularity implies functional aspects of a network and was similar between the compared groups.

In conclusion, the metabolic profiles of healthy control and LUAD patients were compared. Diagnosis of LUAD was achieved using arginine, Lyso.PC.a.C16:0, and PC.aa.C38:3 biomarkers. Network analysis revealed that heterogeneity, diameter, and shortest path were decreased in LUAD. On the contrary, these parameters were increased in advanced-stage compared to early-stage LUAD. Clustering coefficient, network density, and average degree were increased in LUAD compared to the normal healthy controls, whereas these topologic parameters were decreased in advanced LUAD compared to early LUAD. Further studies are required to verify these results with larger samples and other histologic types of lung cancer.

## Supplementary Information

Supplementary Information.

## Data Availability

All the available data generated or analyzed during this study are included in this published article and its supplementary information files.
