# Supplementary material for: Metabolic profiles of lung adenocarcinoma via peripheral blood and diagnostic model construction
Source: Sci Rep. 2023 May 5;13:7304. doi: 10.1038/s41598-023-34575-0 (PMC10163250; doi:10.1038/s41598-023-34575-0)
Supplement: Supplementary file 1 — Supplementary Information. [file 41598_2023_34575_MOESM1_ESM.docx]

Supplementary Materials

Metabolic profiles of lung adenocarcinoma via peripheral blood and diagnostic model construction

Kyung Soo Kim^1^, Seok Whan Moon^1^, Mi Hyung Moon^1^, Kwan Yong Hyun^1^, Seung Joon Kim^2^, Young Koon Kim^2^, Kwang Youl Kim^3*^, Dong Wook Jekarl^4,5*^, Eun-Jee Oh^4,5^, Yonggoo Kim^4^

Supplementary Figure 1. Least absolute shrinkage and selection operator (LASSO) analysis was performed. Cross validation results of lambda and lambda.se (dotted line) to select lowest mean square error (A). Change of estimated coefficients along with log lambda value that decreases the coefficient (B).

**B**


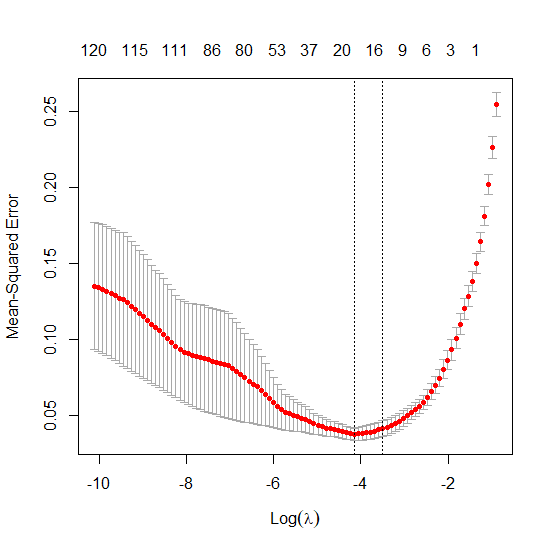


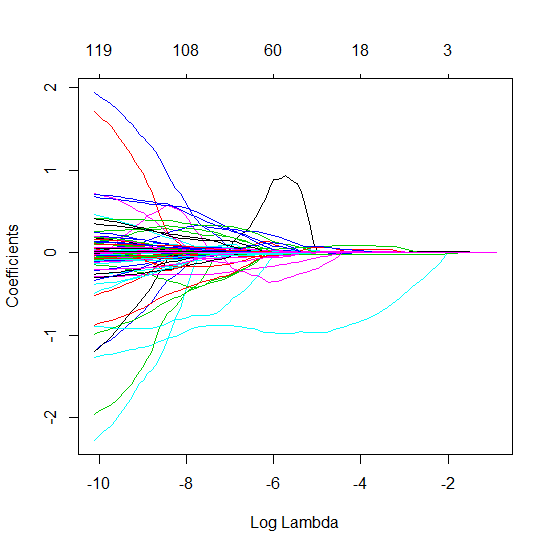


Supplementary Figure 2. ROC curves for arginine, lyso.PC.a.16.0, and PC.aa.C38.3.


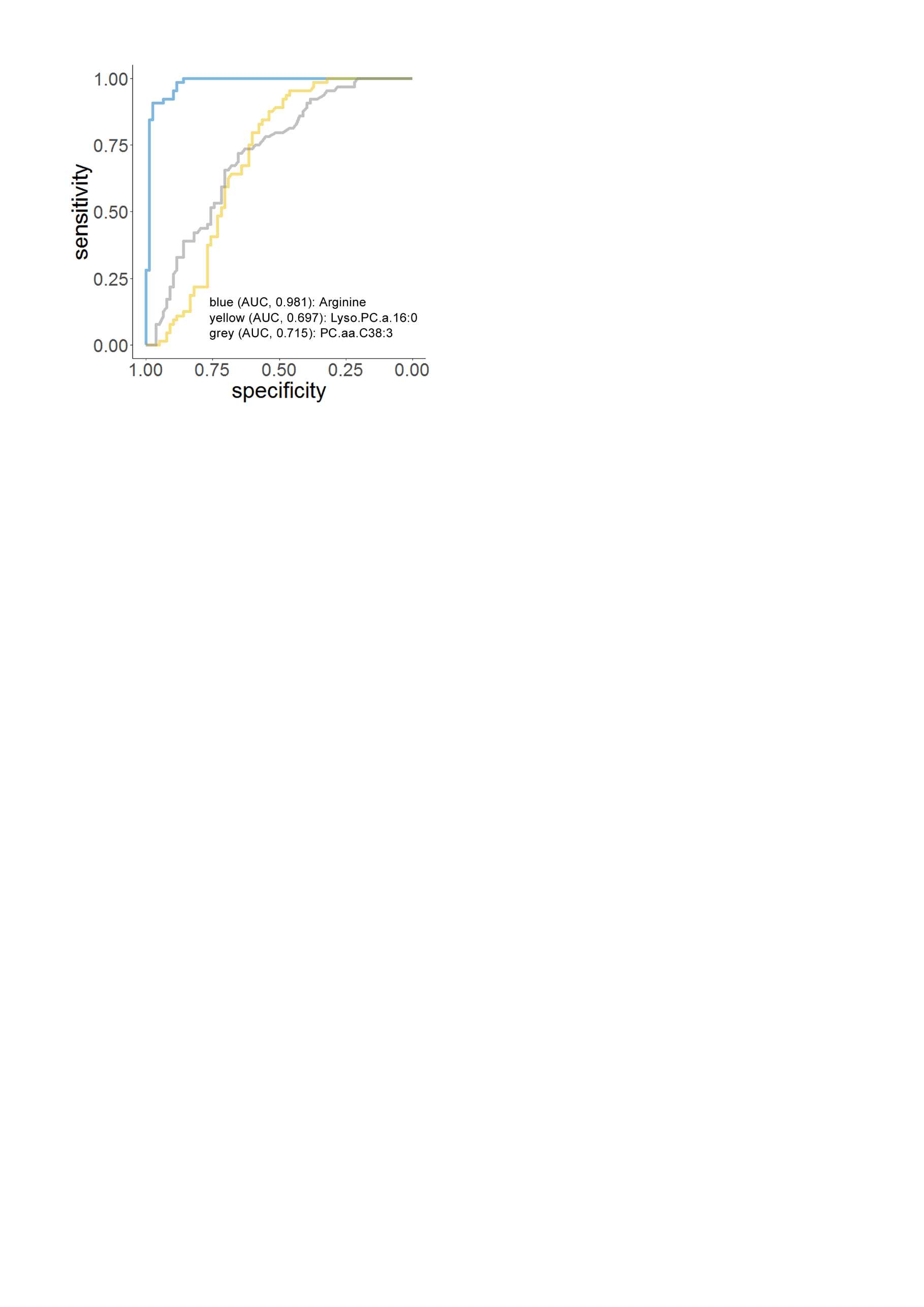


Supplementary Figure 3. Kaplan Meier analysis of 500 lung adenocarcinoma patients from the GDC TCGA LUAD dataset. Genes related with arginine synthesis (A) and PC and LPC (B) were plotted.


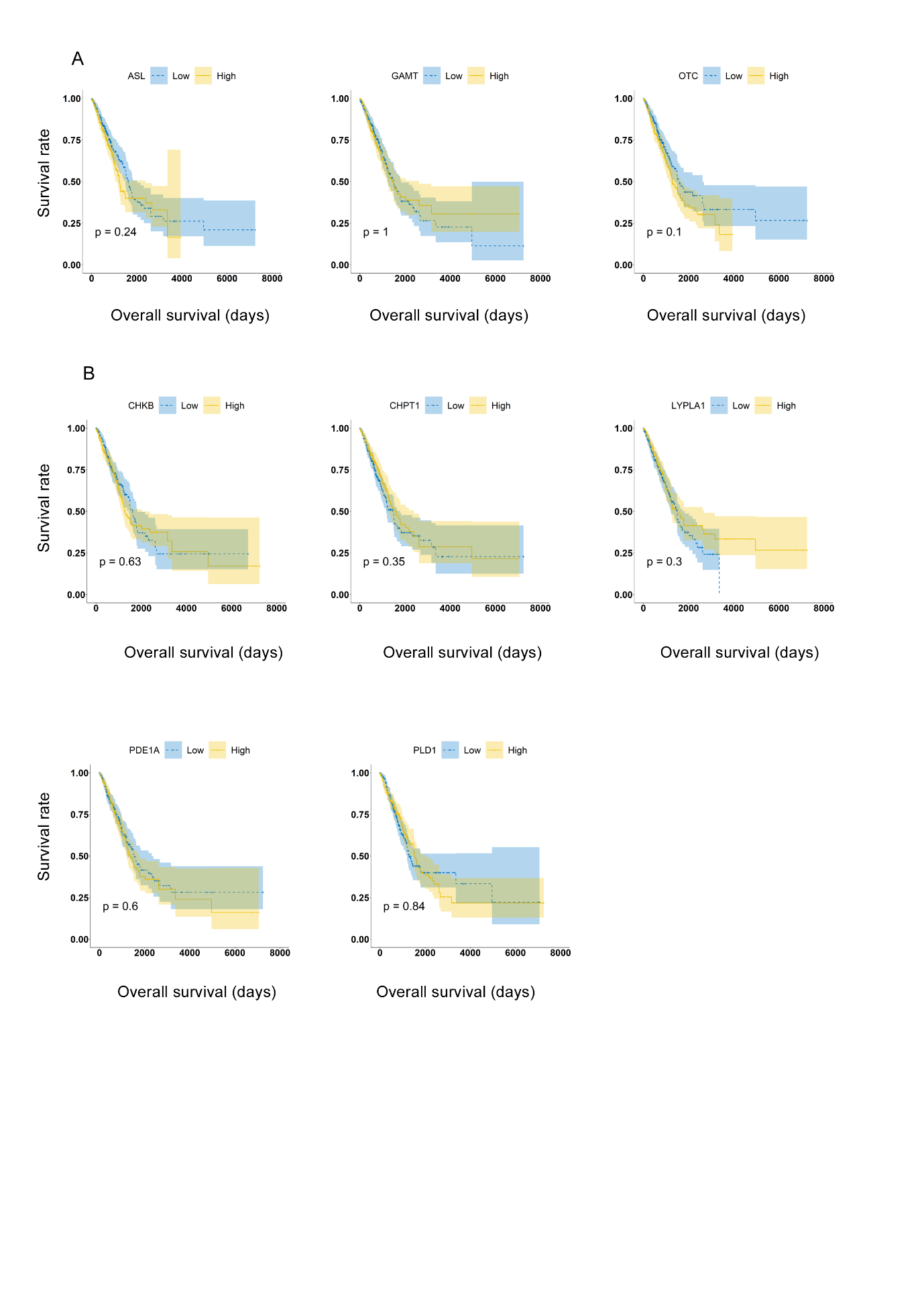


Supplementary Table 1. TNM stages from 79 lung adenocarcinoma patients.

| TNM staging system | n |
| --- | --- |
| TNM |  |
| T1 / T2 | 41 / 23 |
| T1a / T1b | 22 / 19 |
| T2a / T2b | 19 / 4 |
| T3 / T4 | 7 / 8 |
| N0 / N1 | 66 / 5 |
| N2 / N3 | 7 / 1 |
| M0 / M1 | 74 / 5 |
| TNM stage |  |
| stage I / stage II | 53 / 12 |
| stage III / stage IV | 9 / 6 |

Supplementary Table 2. Limit of detection, lower limit of quantitation, upper limit of quantitation for analytes.

| Analyte | BioID | Analytes in full term | LOD  (µM) | LLOQ  (µM) | LUOQ  (µM) |
| --- | --- | --- | --- | --- | --- |
| C0 | HMDB0000062 | Carnitine | 3.94 | 5 | 120 |
| C2 | HMDB0000201 | Acetylcarnitine | 0.753 | 0.4 | 35 |
| C3 | HMDB0000824 | Propionylcarnitine | 0.108 | 0.4 | 15 |
| C4 | HMDB0000736 | Butyrylcarnitine/Isobutyrylcarnitine | 0.038 | 0.4 | 12 |
| C10:1 | HMDB0013205 | Decenoylcarnitine | 0.063 | 0 | 0 |
| C12:1 | HMDB0013326 | Dodecenoylcarnitine | 0.06 | 0 | 0 |
| C14 | HMDB0005066 | Tetradecanoylcarnitine | 0.03 | 0.4 | 6 |
| C14:1 | HMDB0002014 | Tetradecenoylcarnitine | 0.037 | 0 | 0 |
| C14:2 | HMDB0013331 | Tetradecadienoylcarnitine | 0.012 | 0 | 0 |
| C18:1 | HMDB0005065 | Octadecenoylcarnitine | 0.092 | 0 | 0 |
| C18:2 | HMDB0006461 | Octadecadienoylcarnitine | 0.04 | 0 | 0 |
| Ala | HMDB0000161 | Alanine | 1 | 20 | 1600 |
| Arg | HMDB0000517 | Arginine | 0.456 | 5 | 400 |
| Asn | HMDB0000168 | Asparagine | 1.5 | 5 | 400 |
| Asp | HMDB0000191 | Aspartate | 1.5 | 5 | 400 |
| Cit | HMDB0000904 | Citrulline | 1 | 5 | 400 |
| Gln | HMDB0000641 | Glutamine | 1.5 | 20 | 1600 |
| Glu | HMDB0000148 | Glutamate | 2 | 10 | 800 |
| Gly | HMDB0000123 | Glycine | 3.99 | 25 | 2000 |
| His | HMDB0000177 | Histidine | 0.6 | 5 | 400 |
| Ile | HMDB0000172 | Isoleucine | 0.5 | 5 | 400 |
| Leu | HMDB0000687 | Leucine | 1.5 | 50 | 400 |
| Lys | HMDB0000182 | Lysine | 0.5 | 10 | 800 |
| Met | HMDB0000696 | Methionine | 4.5 | 5 | 400 |
| Orn | HMDB0000214 | Ornithine | 1.54 | 5 | 400 |
| Phe | HMDB0000159 | Phenylalanine | 0.678 | 5 | 400 |
| Pro | HMDB0000162 | Proline | 1.13 | 10 | 800 |
| Ser | HMDB0000187 | Serine | 4.23 | 5 | 400 |
| Thr | HMDB0000167 | Threonine | 1.01 | 5 | 400 |
| Trp | HMDB0000929 | Tryptophan | 0.5 | 5 | 400 |
| Tyr | HMDB0000158 | Tyrosine | 0.5 | 5 | 400 |
| Val | HMDB0000883 | Valine | 3.51 | 10 | 800 |
| Creatinine | HMDB0000562 | Creatinine | 2.04 | 10 | 800 |
| Kynurenine | HMDB0000684 | Kynurenine | 0.146 | 1 | 80 |
| Putrescine | HMDB0001414 | Putrescine | 0.02 | 0.1 | 8 |
| Spermine | HMDB0001256 | Spermine | 0.08 | 0.25 | 20 |
| t4-OH-Pro | HMDB0000725 | trans-4-Hydroxyproline | 0.1 | 10 | 80 |
| Taurine | HMDB0000251 | Taurine | 0.8 | 2.5 | 200 |
| total DMA | HMDB0000087 |  | 0.1 | 1.25 | 100 |
| lysoPC a C16:0 | HMDB0010382 | Lysophosphatidylcholine with acyl residue C16:0 | 0.12 | 0 | 0 |
| lysoPC a C16:1 | HMDB0010383 | Lysophosphatidylcholine with acyl residue C16:1 | 0.07 | 0 | 0 |
| lysoPC a C17:0 | HMDB0012108 | Lysophosphatidylcholine with acyl residue C17:0 | 0.05 | 0 | 0 |
| lysoPC a C18:0 | HMDB0010384 | Lysophosphatidylcholine with acyl residue C18:0 | 0.05 | 0 | 0 |
| lysoPC a C18:1 | HMDB0002815 | Lysophosphatidylcholine with acyl residue C18:1 | 0.1 | 0 | 0 |
| lysoPC a C18:2 | HMDB0010386 | Lysophosphatidylcholine with acyl residue C16:2 | 0.1 | 0 | 0 |
| lysoPC a C20:3 | HMDB0010393 | Lysophosphatidylcholine with acyl residue C20:3 | 0.2 | 0 | 0 |
| lysoPC a C20:4 | HMDB0010395 | Lysophosphatidylcholine with acyl residue C20:4:0 | 0.02 | 0 | 0 |
| PC aa C28:1 | HMDB0029221 | Phosphatidylcholine with diacyl residue C28:1 | 0.04 | 0 | 0 |
| PC aa C30:0 | HMDB0007869 | Phosphatidylcholine with diacyl residue C30:0 | 0.2 | 0 | 0 |
| PC aa C32:0 | HMDB0000564 | Phosphatidylcholine with diacyl residue C32:0 | 0.04 | 0 | 0 |
| PC aa C32:1 | HMDB0007872 | Phosphatidylcholine with diacyl residue C32:1 | 0.06 | 0 | 0 |
| PC aa C32:2 | HMDB0007874 | Phosphatidylcholine with diacyl residue C32:2 | 0.03 | 0 | 0 |
| PC aa C32:3 | HMDB0007876 | Phosphatidylcholine with diacyl residue C32:3 | 0.008 | 0 | 0 |
| PC aa C34:1 | HMDB0007971 | Phosphatidylcholine with diacyl residue C34:1 | 0.06 | 0 | 0 |
| PC aa C34:2 | HMDB0007973 | Phosphatidylcholine with diacyl residue C34:2 | 0.1 | 0 | 0 |
| PC aa C34:3 | HMDB0007974 | Phosphatidylcholine with diacyl residue C34:3 | 0.01 | 0 | 0 |
| PC aa C34:4 | HMDB0007883 | Phosphatidylcholine with diacyl residue C34:4 | 0.006 | 0 | 0 |
| PC aa C36:0 | HMDB0007886 | Phosphatidylcholine with diacyl residue C36:0 | 0.2 | 0 | 0 |
| PC aa C36:1 | HMDB0007978 | Phosphatidylcholine with diacyl residue C36:1 | 0.03 | 0 | 0 |
| PC aa C36:2 | HMDB0007979 | Phosphatidylcholine with diacyl residue C36:2 | 0.15 | 0 | 0 |
| PC aa C36:3 | HMDB0007980 | Phosphatidylcholine with diacyl residue C36:3 | 0.04 | 0 | 0 |
| PC aa C36:4 | HMDB0007982 | Phosphatidylcholine with diacyl residue C36:4 | 0.04 | 0 | 0 |
| PC aa C36:5 | HMDB0007984 | Phosphatidylcholine with diacyl residue C36:5 | 0.01 | 0 | 0 |
| PC aa C36:6 | HMDB0007892 | Phosphatidylcholine with diacyl residue C36:6 | 0.015 | 0 | 0 |
| PC aa C38:0 | HMDB0007893 | Phosphatidylcholine with diacyl residue C38:0 | 0.2 | 0 | 0 |
| PC aa C38:1 | HMDB0007894 | Phosphatidylcholine with diacyl residue C38:1 | 0.08 | 0 | 0 |
| PC aa C38:3 | HMDB0008020 | Phosphatidylcholine with diacyl residue C38:3 | 0.04 | 0 | 0 |
| PC aa C38:4 | HMDB0007988 | Phosphatidylcholine with diacyl residue C38:4 | 0.03 | 0 | 0 |
| PC aa C38:5 | HMDB0007989 | Phosphatidylcholine with diacyl residue C38:5 | 0.015 | 0 | 0 |
| PC aa C38:6 | HMDB0007991 | Phosphatidylcholine with diacyl residue C38:6 | 0.02 | 0 | 0 |
| PC aa C40:4 | HMDB0008054 | Phosphatidylcholine with diacyl residue C40:4 | 0.01 | 0 | 0 |
| PC aa C40:5 | HMDB0008055 | Phosphatidylcholine with diacyl residue C40:5 | 0.04 | 0 | 0 |
| PC aa C40:6 | HMDB0008057 | Phosphatidylcholine with diacyl residue C40:6 | 1.2 | 0 | 0 |
| PC aa C42:0 | HMDB0008058 | Phosphatidylcholine with diacyl residue C42:0 | 0.083 | 0 | 0 |
| PC aa C42:1 | HMDB0008059 | Phosphatidylcholine with diacyl residue C42:1 | 0.008 | 0 | 0 |
| PC aa C42:5 | HMDB0008287 | Phosphatidylcholine with diacyl residue C42:5 | 0.05 | 0 | 0 |
| PC aa C42:6 | HMDB0008288 | Phosphatidylcholine with diacyl residue C42:6 | 0.115 | 0 | 0 |
| PC ae C30:0 | HMDB0013341 | Phosphatidylcholine with acyl-alkyl residue C30:0 | 0.15 | 0 | 0 |
| PC ae C32:1 | HMDB0007896 | Phosphatidylcholine with acyl-alkyl residue C32:1 | 0.009 | 0 | 0 |
| PC ae C32:2 | HMDB0013411 | Phosphatidylcholine with acyl-alkyl residue C32:2 | 0.346 | 0 | 0 |
| PC ae C34:0 | HMDB0013405 | Phosphatidylcholine with acyl-alkyl residue C34:0 | 0.017 | 0 | 0 |
| PC ae C34:1 | HMDB0013426 | Phosphatidylcholine with acyl-alkyl residue C34:1 | 0.012 | 0 | 0 |
| PC ae C34:2 | HMDB0011151 | Phosphatidylcholine with acyl-alkyl residue C34:2 | 0.01 | 0 | 0 |
| PC ae C34:3 | HMDB0013413 | Phosphatidylcholine with acyl-alkyl residue C34:3 | 0.015 | 0 | 0 |
| PC ae C36:0 | HMDB0013406 | Phosphatidylcholine with acyl-alkyl residue C36:0 | 0.12 | 0 | 0 |
| PC ae C36:1 | HMDB0013414 | Phosphatidylcholine with acyl-alkyl residue C36:1 | 0.408 | 0 | 0 |
| PC ae C36:2 | HMDB0011243 | Phosphatidylcholine with acyl-alkyl residue C36:2 | 0.01 | 0 | 0 |
| PC ae C36:3 | HMDB0013425 | Phosphatidylcholine with acyl-alkyl residue C36:3 | 0.007 | 0 | 0 |
| PC ae C36:4 | HMDB0013407 | Phosphatidylcholine with acyl-alkyl residue C36:4 | 0.013 | 0 | 0 |
| PC ae C36:5 | HMDB0011220 | Phosphatidylcholine with acyl-alkyl residue C36:5 | 0.012 | 0 | 0 |
| PC ae C38:0 | HMDB0013408 | Phosphatidylcholine with acyl-alkyl residue C38:0 | 0.224 | 0 | 0 |
| PC ae C38:1 | HMDB0013408 | Phosphatidylcholine with acyl-alkyl residue C38:1 | 0.015 | 0 | 0 |
| PC ae C38:2 | HMDB0013416 | Phosphatidylcholine with acyl-alkyl residue C38:2 | 0.018 | 0 | 0 |
| PC ae C38:3 | HMDB0013431 | Phosphatidylcholine with acyl-alkyl residue C38:3 | 0.01 | 0 | 0 |
| PC ae C38:4 | HMDB0013420 | Phosphatidylcholine with acyl-alkyl residue C38:4 | 0.015 | 0 | 0 |
| PC ae C38:5 | HMDB0011253 | Phosphatidylcholine with acyl-alkyl residue C38:5 | 0.01 | 0 | 0 |
| PC ae C38:6 | HMDB0013409 | Phosphatidylcholine with acyl-alkyl residue C38:6 | 0.03 | 0 | 0 |
| PC ae C40:1 | HMDB0013433 | Phosphatidylcholine with acyl-alkyl residue C40:1 | 0.06 | 0 | 0 |
| PC ae C40:2 | HMDB0013437 | Phosphatidylcholine with acyl-alkyl residue C40:2 | 0.01 | 0 | 0 |
| PC ae C40:3 | HMDB0013445 | Phosphatidylcholine with acyl-alkyl residue C40:3 | 0.015 | 0 | 0 |
| PC ae C40:4 | HMDB0013442 | Phosphatidylcholine with acyl-alkyl residue C40:4 | 0.1 | 0 | 0 |
| PC ae C40:5 | HMDB0013444 | Phosphatidylcholine with acyl-alkyl residue C40:5 | 0.006 | 0 | 0 |
| PC ae C40:6 | HMDB0013422 | Phosphatidylcholine with acyl-alkyl residue C40:6 | 0.025 | 0 | 0 |
| PC ae C42:2 | HMDB0013438 | Phosphatidylcholine with acyl-alkyl residue C42:2 | 0.006 | 0 | 0 |
| PC ae C42:3 | HMDB0013458 | Phosphatidylcholine with acyl-alkyl residue C42:3 | 0.006 | 0 | 0 |
| PC ae C42:4 | HMDB0013448 | Phosphatidylcholine with acyl-alkyl residue C42:4 | 0.3 | 0 | 0 |
| PC ae C42:5 | HMDB0013451 | Phosphatidylcholine with acyl-alkyl residue C42:5 | 0.902 | 0 | 0 |
| PC ae C44:5 | HMDB0013456 | Phosphatidylcholine with acyl-alkyl residue C44:5 | 0.02 | 0 | 0 |
| PC ae C44:6 | HMDB0013450 | Phosphatidylcholine with acyl-alkyl residue C55:6 | 0.352 | 0 | 0 |
| SM (OH) C14:1 | HMDB0013462 | Hydroxysphingomyelin with acyl residue sum C14:1 | 0.376 | 0 | 0 |
| SM (OH) C16:1 | HMDB0013463 | Hydroxysphingomyelin with acyl residue sum C16:1 | 0.012 | 0 | 0 |
| SM (OH) C22:1 | HMDB0013466 | Hydroxysphingomyelin with acyl residue sum C22:1 | 0.015 | 0 | 0 |
| SM (OH) C22:2 | HMDB0013467 | Hydroxysphingomyelin with acyl residue sum C22:2 | 0.01 | 0 | 0 |
| SM (OH) C24:1 | HMDB0013469 | Hydroxysphingomyelin with acyl residue sum C24:1 | 0.01 | 0 | 0 |
| SM C16:0 | HMDB0010169 | Sphingomyelin with acyl residue sum C16:0 | 0.03 | 0 | 0 |
| SM C16:1 | HMDB0029216 | Sphingomyelin with acyl residue sum C16:1 | 0.01 | 0 | 0 |
| SM C18:0 | HMDB0001348 | Sphingomyelin with acyl residue sum C18:0 | 0.07 | 0 | 0 |
| SM C18:1 | HMDB0012100 | Sphingomyelin with acyl residue sum C18:1 | 0.01 | 0 | 0 |
| SM C24:0 | HMDB0011697 | Sphingomyelin with acyl residue sum C24:0 | 0.13 | 0 | 0 |
| SM C24:1 | HMDB0012107 | Sphingomyelin with acyl residue sum C24:1 | 0.035 | 0 | 0 |
| H1 | HMDB0000122 | Hexoses | 15.5 | 200 | 30000 |

Supplementary Table 3. Concentration of measured analytes for control and lung adenocarcinoma group

|  | BioID | Normal Healthy | Lung Adenocarcinoma | P-value |
| --- | --- | --- | --- | --- |
|  |  | (n=78) | (n=65) |  |
| sex |  |  |  | NS |
| Female |  | 50 (64.1%) | 37 (56.9%) |  |
| Male |  | 28 (35.9%) | 27 (43.1%) |  |
| Age |  | 41.6 ± 14.3 | 64.9 ± 10.4 | <0.001 |
| C0 | HMDB0000062 | 25.3 ± 8.9 | 36.5 ± 7.4 | <0.001 |
| C2 | HMDB0000201 | 6.6 ± 3.6 | 6.2 ± 2.1 | NS |
| C3 | HMDB0000824 | 0.2 ± 0.1 | 0.3 ± 0.1 | <0.001 |
| C4 | HMDB0000736 | 0.1 ± 0.0 | 0.2 ± 0.1 | <0.001 |
| C10:1 | HMDB0013205 | 0.3 ± 0.1 | 0.2 ± 0.1 | <0.001 |
| C12:1 | HMDB0013326 | 0.1 ± 0.0 | 0.1 ± 0.0 | 0.001 |
| C14 | HMDB0005066 | 0.0 ± 0.0 | 0.0 ± 0.0 | 0.019 |
| C14:1 | HMDB0002014 | 0.1 ± 0.1 | 0.1 ± 0.0 | 0.003 |
| C14:2 | HMDB0013331 | 0.1 ± 0.0 | 0.0 ± 0.0 | 0.003 |
| C18:1 | HMDB0005065 | 0.2 ± 0.1 | 0.1 ± 0.0 | <0.001 |
| C18:2 | HMDB0006461 | 0.1 ± 0.0 | 0.1 ± 0.0 | <0.001 |
| Alanine | HMDB0000161 | 318.3 ± 109.1 | 372.0 ± 92.2 | 0.002 |
| Arginine | HMDB0000517 | 29.6 ± 13.4 | 73.9 ± 17.1 | <0.001 |
| Asparagine | HMDB0000168 | 31.4 ± 9.1 | 43.9 ± 8.8 | <0.001 |
| Aspartate | HMDB0000191 | 5.0 ± 3.3 | 1.7 ± 1.6 | <0.001 |
| Citrulline | HMDB0000904 | 16.8 ± 6.0 | 29.2 ± 9.0 | <0.001 |
| Glutamine | HMDB0000641 | 421.1 ± 139.3 | 692.7 ± 96.2 | <0.001 |
| Glutamate | HMDB0000148 | 84.3 ± 38.0 | 53.0 ± 18.1 | <0.001 |
| Glycine | HMDB0000123 | 187.2 ± 56.6 | 256.7 ± 75.6 | <0.001 |
| Histidine | HMDB0000177 | 64.4 ± 17.8 | 81.3 ± 10.7 | <0.001 |
| Isoleucine | HMDB0000172 | 58.7 ± 19.8 | 72.0 ± 13.2 | <0.001 |
| Leucine | HMDB0000687 | 126.1 ± 45.3 | 149.4 ± 27.5 | <0.001 |
| Lysine | HMDB0000182 | 200.6 ± 67.0 | 259.3 ± 40.0 | <0.001 |
| Methionine | HMDB0000696 | 17.8 ± 4.9 | 24.6 ± 4.6 | <0.001 |
| Ornithine | HMDB0000214 | 90.7 ± 32.5 | 76.8 ± 19.5 | 0.02 |
| Phenylalanine | HMDB0000159 | 47.3 ± 13.0 | 60.2 ± 10.1 | <0.001 |
| Proline | HMDB0000162 | 105.6 ± 52.1 | 142.8 ± 39.3 | <0.001 |
| Serine | HMDB0000187 | 101.0 ± 29.5 | 123.7 ± 31.6 | <0.001 |
| Threonine | HMDB0000167 | 85.7 ± 28.8 | 119.8 ± 23.5 | <0.001 |
| Tryptophan | HMDB0000929 | 39.3 ± 12.5 | 42.4 ± 8.9 | NS |
| Tyrosine | HMDB0000158 | 44.9 ± 12.5 | 60.9 ± 12.8 | <0.001 |
| Valine | HMDB0000883 | 186.8 ± 60.7 | 229.1 ± 34.4 | <0.001 |
| Creatinine | HMDB0000562 | 46.1 ± 16.9 | 66.5 ± 66.6 | 0.002 |
| Kynurenine | HMDB0000684 | 2.0 ± 1.2 | 2.4 ± 0.9 | 0.002 |
| Putrescine | HMDB0001414 | 0.1 ± 0.0 | 0.1 ± 0.0 | 0.041 |
| Spermine | HMDB0001256 | 0.3 ± 0.2 | 0.2 ± 0.1 | <0.001 |
| t4-OH-Pro | HMDB0000725 | 6.4 ± 5.2 | 9.8 ± 7.6 | <0.001 |
| Taurine | HMDB0000251 | 86.4 ± 66.2 | 63.6 ± 28.8 | 0.02 |
| total DMA | HMDB0000087 | 0.4 ± 0.3 | 0.8 ± 0.4 | <0.001 |
| lysoPC a C16:0 | HMDB0010382 | 74.5 ± 29.2 | 56.2 ± 13.6 | <0.001 |
| lysoPC a C16:1 | HMDB0010383 | 1.8 ± 0.8 | 1.7 ± 0.5 | 0.196 |
| lysoPC a C17:0 | HMDB0012108 | 1.4 ± 0.6 | 1.2 ± 0.3 | <0.001 |
| lysoPC a C18:0 | HMDB0010384 | 23.9 ± 9.6 | 18.5 ± 5.6 | <0.001 |
| lysoPC a C18:1 | HMDB0002815 | 10.6 ± 4.2 | 10.0 ± 2.6 | NS |
| lysoPC a C18:2 | HMDB0010386 | 14.2 ± 6.2 | 14.5 ± 4.5 | NS |
| lysoPC a C20:3 | HMDB0010393 | 1.0 ± 0.5 | 1.0 ± 0.4 | NS |
| lysoPC a C20:4 | HMDB0010395 | 4.1 ± 1.8 | 3.3 ± 1.2 | 0.001 |
| lysoPC a C28:1 | HMDB0029221 | 1.4 ± 0.5 | 1.6 ± 0.5 | 0.01 |
| PC aa C30:0 | HMDB0007869 | 1.6 ± 0.6 | 2.2 ± 0.8 | <0.001 |
| PC aa C32:0 | HMDB0000564 | 7.0 ± 2.7 | 9.1 ± 2.5 | <0.001 |
| PC aa C32:1 | HMDB0007872 | 5.0 ± 2.8 | 8.4 ± 3.7 | <0.001 |
| PC aa C32:2 | HMDB0007874 | 1.3 ± 0.9 | 2.0 ± 0.9 | <0.001 |
| PC aa C32:3 | HMDB0007876 | 0.2 ± 0.1 | 0.2 ± 0.1 | <0.001 |
| PC aa C34:1 | HMDB0007971 | 100.3 ± 44.4 | 124.6 ± 31.6 | <0.001 |
| PC aa C34:2 | HMDB0007973 | 173.0 ± 63.1 | 201.0 ± 56.9 | 0.006 |
| PC aa C34:3 | HMDB0007974 | 6.3 ± 3.7 | 10.3 ± 4.2 | <0.001 |
| PC aa C34:4 | HMDB0007883 | 0.6 ± 0.3 | 0.8 ± 0.3 | <0.001 |
| PC aa C36:0 | HMDB0007886 | 2.7 ± 1.3 | 3.4 ± 1.2 | <0.001 |
| PC aa C36:1 | HMDB0007978 | 24.6 ± 10.9 | 31.5 ± 9.6 | <0.001 |
| PC aa C36:2 | HMDB0007979 | 118.6 ± 48.2 | 141.1 ± 40.4 | 0.003 |
| PC aa C36:3 | HMDB0007980 | 52.3 ± 22.2 | 62.7 ± 17.2 | 0.002 |
| PC aa C36:4 | HMDB0007982 | 90.2 ± 40.1 | 94.6 ± 24.5 | NS |
| PC aa C36:5 | HMDB0007984 | 21.4 ± 19.0 | 31.4 ± 16.2 | 0.001 |
| PC aa C36:6 | HMDB0007892 | 0.7 ± 0.4 | 1.0 ± 0.4 | <0.001 |
| PC aa C38:0 | HMDB0007893 | 2.3 ± 1.0 | 2.9 ± 0.9 | 0.001 |
| PC aa C38:1 | HMDB0007894 | 0.8 ± 0.4 | 1.0 ± 0.4 | 0.001 |
| PC aa C38:3 | HMDB0008020 | 20.9 ± 9.1 | 26.8 ± 7.3 | <0.001 |
| PC aa C38:4 | HMDB0007988 | 57.8 ± 25.9 | 64.2 ± 18.6 | NS |
| PC aa C38:5 | HMDB0007989 | 31.7 ± 17.8 | 41.2 ± 13.8 | <0.001 |
| PC aa C38:6 | HMDB0007991 | 67.8 ± 35.9 | 85.5 ± 28.3 | 0.002 |
| PC aa C40:4 | HMDB0008054 | 1.5 ± 0.7 | 1.7 ± 0.5 | 0.014 |
| PC aa C40:5 | HMDB0008055 | 5.9 ± 3.8 | 8.1 ± 3.0 | <0.001 |
| PC aa C40:6 | HMDB0008057 | 25.8 ± 14.9 | 36.9 ± 12.2 | <0.001 |
| PC aa C42:0 | HMDB0008058 | 0.4 ± 0.2 | 0.5 ± 0.2 | <0.001 |
| PC aa C42:1 | HMDB0008059 | 0.2 ± 0.1 | 0.2 ± 0.1 | <0.001 |
| PC aa C42:5 | HMDB0008287 | 0.2 ± 0.1 | 0.3 ± 0.1 | 0.002 |
| PC aa C42:6 | HMDB0008288 | 0.4 ± 0.2 | 0.5 ± 0.2 | <0.001 |
| PC ae C30:0 | HMDB0013341 | 0.2 ± 0.1 | 0.2 ± 0.1 | 0.014 |
| PC ae C32:1 | HMDB0007896 | 1.5 ± 0.6 | 1.8 ± 0.5 | 0.003 |
| PC ae C32:2 | HMDB0013411 | 0.4 ± 0.1 | 0.4 ± 0.1 | 0.003 |
| PC ae C34:0 | HMDB0013405 | 0.8 ± 0.3 | 1.0 ± 0.3 | <0.001 |
| PC ae C34:1 | HMDB0013426 | 3.9 ± 1.4 | 4.6 ± 1.1 | 0.002 |
| PC ae C34:2 | HMDB0011151 | 5.8 ± 2.4 | 6.0 ± 1.9 | 0.43 |
| PC ae C34:3 | HMDB0013413 | 4.7 ± 2.0 | 4.6 ± 1.5 | NS |
| PC ae C36:0 | HMDB0013406 | 0.6 ± 0.3 | 0.9 ± 0.3 | <0.001 |
| PC ae C36:1 | HMDB0013414 | 3.8 ± 1.3 | 4.3 ± 1.0 | 0.006 |
| PC ae C36:2 | HMDB0011243 | 6.5 ± 2.4 | 7.3 ± 2.1 | 0.023 |
| PC ae C36:3 | HMDB0013425 | 3.8 ± 1.6 | 4.0 ± 1.1 | NS |
| PC ae C36:4 | HMDB0013407 | 10.1 ± 4.3 | 9.7 ± 3.1 | NS |
| PC ae C36:5 | HMDB0011220 | 6.9 ± 2.8 | 7.5 ± 2.3 | NS |
| PC ae C38:0 | HMDB0013408 | 1.6 ± 0.8 | 2.0 ± 0.7 | <0.001 |
| PC ae C38:1 | HMDB0013408 | 0.4 ± 0.2 | 0.6 ± 0.2 | <0.001 |
| PC ae C38:2 | HMDB0013416 | 0.8 ± 0.4 | 1.1 ± 0.4 | <0.001 |
| PC ae C38:3 | HMDB0013431 | 1.7 ± 0.6 | 2.0 ± 0.5 | 0.017 |
| PC ae C38:4 | HMDB0013420 | 6.7 ± 2.5 | 6.4 ± 1.7 | NS |
| PC ae C38:5 | HMDB0011253 | 9.1 ± 3.6 | 9.4 ± 2.4 | NS |
| PC ae C38:6 | HMDB0013409 | 4.9 ± 2.1 | 5.8 ± 1.9 | 0.009 |
| PC ae C40:1 | HMDB0013433 | 0.8 ± 0.4 | 0.9 ± 0.3 | NS |
| PC ae C40:2 | HMDB0013437 | 0.9 ± 0.3 | 1.0 ± 0.3 | 0.001 |
| PC ae C40:3 | HMDB0013445 | 0.5 ± 0.2 | 0.5 ± 0.1 | NS |
| PC ae C40:4 | HMDB0013442 | 1.1 ± 0.4 | 1.2 ± 0.3 | NS |
| PC ae C40:5 | HMDB0013444 | 1.9 ± 0.7 | 2.2 ± 0.6 | 0.009 |
| PC ae C40:6 | HMDB0013422 | 3.3 ± 1.4 | 4.0 ± 1.2 | 0.001 |
| PC ae C42:2 | HMDB0013438 | 0.3 ± 0.1 | 0.4 ± 0.1 | <0.001 |
| PC ae C42:3 | HMDB0013458 | 0.4 ± 0.2 | 0.5 ± 0.1 | NS |
| PC ae C42:4 | HMDB0013448 | 0.5 ± 0.2 | 0.5 ± 0.2 | NS |
| PC ae C42:5 | HMDB0013451 | 1.2 ± 0.4 | 1.2 ± 0.3 | NS |
| PC ae C44:5 | HMDB0013456 | 0.9 ± 0.4 | 0.9 ± 0.3 | NS |
| PC ae C44:6 | HMDB0013450 | 0.7 ± 0.3 | 0.8 ± 0.2 | 0.027 |
| SM (OH) C14:1 | HMDB0013462 | 3.2 ± 1.1 | 3.6 ± 1.1 | 0.033 |
| SM (OH) C16:1 | HMDB0013463 | 2.4 ± 0.8 | 2.6 ± 0.7 | NS |
| SM (OH) C22:1 | HMDB0013466 | 7.6 ± 2.5 | 8.3 ± 2.1 | NS |
| SM (OH) C22:2 | HMDB0013467 | 6.6 ± 2.3 | 7.4 ± 1.8 | 0.017 |
| SM (OH) C24:1 | HMDB0013469 | 0.9 ± 0.3 | 1.0 ± 0.4 | 0.004 |
| SM C16:0 | HMDB0010169 | 76.7 ± 23.1 | 91.2 ± 17.4 | <0.001 |
| SM C16:1 | HMDB0029216 | 10.1 ± 3.1 | 11.7 ± 2.5 | 0.001 |
| SM C18:0 | HMDB0001348 | 18.2 ± 5.8 | 19.2 ± 4.4 | NS |
| SM C18:1 | HMDB0012100 | 9.0 ± 2.9 | 9.4 ± 2.5 | NS |
| SM C24:0 | HMDB0011697 | 13.0 ± 4.2 | 15.0 ± 3.5 | 0.002 |
| SM C24:1 | HMDB0012107 | 36.6 ± 12.4 | 44.0 ± 9.8 | <0.001 |
| H1 | HMDB0000122 | 1718.5 ± 967.1 | 4981.8 ± 817.2 | <0.001 |

| a, acyl residue; aa, diacyl residue; ae, acyl-alkyl, residue |  |
| --- | --- |
| DMA, dimethylamine; t4-OH-Pro, trans-4-hydrox-proline; H1, hexoses (including glucose) | |
| LysoPC, lysophosphatidylcholines; PC, phosphatidylcholines; SM, sphinogomyelins | |

Supplementary Table 4. Concentration of measured analytes for early and advanced lung adenocarcinoma group

|  | BioID | Early LUAD | Advanced LUAD | P-value |
| --- | --- | --- | --- | --- |
|  |  | (n=44) | (n=20) |  |
| sex |  |  |  | 0.027 |
| Female |  | 30 (68.2%) | 7 (35.0%) |  |
| Male |  | 14 (31.8%) | 13 (65.0%) |  |
| Age |  | 65.1 ± 9.5 | 64.5 ± 12.5 | NS |
| C0 | HMDB0000062 | 36.4 ± 7.4 | 36.0 ± 7.3 | NS |
| C2 | HMDB0000201 | 6.5 ± 2.1 | 5.3 ± 1.8 | NS |
| C3 | HMDB0000824 | 0.3 ± 0.1 | 0.3 ± 0.1 | NS |
| C4 | HMDB0000736 | 0.1 ± 0.0 | 0.2 ± 0.1 | NS |
| WBC | HMDB0013205 | 0.2 ± 0.1 | 0.1 ± 0.1 | NS |
| C12:1 | HMDB0013326 | 0.1 ± 0.0 | 0.1 ± 0.0 | NS |
| C14 | HMDB0005066 | 0.0 ± 0.0 | 0.0 ± 0.0 | NS |
| C14:1 | HMDB0002014 | 0.1 ± 0.0 | 0.1 ± 0.1 | NS |
| C14:2 | HMDB0013331 | 0.0 ± 0.0 | 0.0 ± 0.0 | NS |
| C18:1 | HMDB0005065 | 0.1 ± 0.0 | 0.1 ± 0.0 | NS |
| C18:2 | HMDB0006461 | 0.1 ± 0.0 | 0.1 ± 0.0 | NS |
| Alanine | HMDB0000161 | 363.9 ± 86.2 | 386.1 ± 105.7 | NS |
| Arginine | HMDB0000517 | 74.6 ± 18.1 | 72.0 ± 15.5 | NS |
| Asparagine | HMDB0000168 | 44.4 ± 9.3 | 43.2 ± 7.8 | NS |
| Aspartate | HMDB0000191 | 1.5 ± 1.6 | 2.1 ± 1.5 | NS |
| Citrulline | HMDB0000904 | 30.1 ± 9.4 | 27.2 ± 7.9 | NS |
| Glutamine | HMDB0000641 | 690.5 ± 88.9 | 701.1 ± 113.9 | NS |
| Glutamate | HMDB0000148 | 51.7 ± 18.1 | 55.8 ± 18.8 | NS |
| Glycine | HMDB0000123 | 264.2 ± 79.6 | 241.9 ± 66.8 | NS |
| Histidine | HMDB0000177 | 81.9 ± 11.5 | 79.5 ± 9.0 | NS |
| Isoleucine | HMDB0000172 | 69.6 ± 11.2 | 76.9 ± 16.1 | NS |
| Leucine | HMDB0000687 | 147.5 ± 26.5 | 153.2 ± 30.6 | NS |
| Lysine | HMDB0000182 | 257.9 ± 43.5 | 263.8 ± 32.5 | NS |
| Methionine | HMDB0000696 | 24.5 ± 4.8 | 24.7 ± 4.5 | NS |
| Ornithine | HMDB0000214 | 77.6 ± 20.4 | 75.4 ± 18.4 | NS |
| Phenylalanine | HMDB0000159 | 60.0 ± 10.8 | 60.7 ± 8.7 | NS |
| Proline | HMDB0000162 | 138.1 ± 37.8 | 151.6 ± 42.2 | NS |
| Serine | HMDB0000187 | 128.8 ± 32.6 | 112.9 ± 27.7 | NS |
| Threonine | HMDB0000167 | 122.2 ± 21.9 | 112.5 ± 24.8 | NS |
| Tryptophan | HMDB0000929 | 41.9 ± 8.7 | 42.9 ± 9.1 | NS |
| Tyrosine | HMDB0000158 | 59.9 ± 12.8 | 62.8 ± 13.2 | NS |
| Valine | HMDB0000883 | 226.9 ± 30.3 | 234.6 ± 43.1 | NS |
| Creatinine | HMDB0000562 | 69.9 ± 80.7 | 58.8 ± 10.6 | NS |
| Kynurenine | HMDB0000684 | 2.3 ± 0.9 | 2.7 ± 0.9 | NS |
| Putrescine | HMDB0001414 | 0.1 ± 0.0 | 0.1 ± 0.0 | NS |
| Spermine | HMDB0001256 | 0.2 ± 0.0 | 0.2 ± 0.1 | NS |
| t4-OH-Pro | HMDB0000725 | 9.6 ± 8.4 | 10.1 ± 5.8 | NS |
| Taurine | HMDB0000251 | 66.8 ± 30.9 | 57.6 ± 23.7 | NS |
| total DMA | HMDB0000087 | 0.8 ± 0.5 | 0.7 ± 0.2 | NS |
| lysoPC a C16:0 | HMDB0010382 | 57.1 ± 13.8 | 52.7 ± 11.2 | NS |
| lysoPC a C16:1 | HMDB0010383 | 1.7 ± 0.5 | 1.6 ± 0.4 | NS |
| lysoPC a C17:0 | HMDB0012108 | 1.2 ± 0.3 | 1.0 ± 0.2 | 0.014 |
| lysoPC a C18:0 | HMDB0010384 | 19.6 ± 6.0 | 15.8 ± 3.5 | 0.002 |
| lysoPC a C18:1 | HMDB0002815 | 10.1 ± 2.7 | 9.3 ± 2.0 | NS |
| lysoPC a C18:2 | HMDB0010386 | 14.9 ± 4.6 | 13.2 ± 3.2 | NS |
| lysoPC a C20:3 | HMDB0010393 | 1.0 ± 0.4 | 1.0 ± 0.4 | NS |
| lysoPC a C20:4 | HMDB0010395 | 3.3 ± 1.2 | 3.0 ± 0.9 | NS |
| lysoPC a C28:1 | HMDB0029221 | 1.7 ± 0.5 | 1.4 ± 0.3 | 0.002 |
| PC aa C30:0 | HMDB0007869 | 2.2 ± 0.9 | 2.0 ± 0.5 | NS |
| PC aa C32:0 | HMDB0000564 | 9.4 ± 2.8 | 8.3 ± 1.4 | NS |
| PC aa C32:1 | HMDB0007872 | 8.5 ± 3.6 | 7.9 ± 3.8 | NS |
| PC aa C32:2 | HMDB0007874 | 2.2 ± 1.0 | 1.7 ± 0.5 | 0.018 |
| PC aa C32:3 | HMDB0007876 | 0.3 ± 0.1 | 0.2 ± 0.1 | NS |
| PC aa C34:1 | HMDB0007971 | 125.5 ± 30.4 | 119.8 ± 33.1 | NS |
| PC aa C34:2 | HMDB0007973 | 207.6 ± 60.8 | 181.4 ± 38.2 | 0.041 |
| PC aa C34:3 | HMDB0007974 | 10.7 ± 4.6 | 9.1 ± 3.3 | NS |
| PC aa C34:4 | HMDB0007883 | 0.8 ± 0.3 | 0.7 ± 0.2 | NS |
| PC aa C36:0 | HMDB0007886 | 3.5 ± 1.2 | 3.3 ± 1.2 | NS |
| PC aa C36:1 | HMDB0007978 | 32.3 ± 9.7 | 29.1 ± 9.1 | NS |
| PC aa C36:2 | HMDB0007979 | 147.4 ± 43.0 | 124.4 ± 27.7 | 0.013 |
| PC aa C36:3 | HMDB0007980 | 63.4 ± 17.7 | 59.4 ± 14.8 | NS |
| PC aa C36:4 | HMDB0007982 | 96.7 ± 23.4 | 87.0 ± 22.7 | NS |
| PC aa C36:5 | HMDB0007984 | 35.0 ± 17.7 | 23.3 ± 9.1 | 0.001 |
| PC aa C36:6 | HMDB0007892 | 1.1 ± 0.4 | 0.9 ± 0.3 | 0.024 |
| PC aa C38:0 | HMDB0007893 | 3.0 ± 0.9 | 2.6 ± 0.9 | NS |
| PC aa C38:1 | HMDB0007894 | 1.1 ± 0.4 | 0.9 ± 0.3 | NS |
| PC aa C38:3 | HMDB0008020 | 27.4 ± 7.1 | 25.1 ± 7.7 | NS |
| PC aa C38:4 | HMDB0007988 | 66.7 ± 17.9 | 57.2 ± 18.1 | NS |
| PC aa C38:5 | HMDB0007989 | 43.6 ± 14.7 | 35.4 ± 10.1 | 0.027 |
| PC aa C38:6 | HMDB0007991 | 91.3 ± 28.8 | 72.0 ± 23.3 | 0.011 |
| PC aa C40:4 | HMDB0008054 | 1.7 ± 0.5 | 1.7 ± 0.6 | NS |
| PC aa C40:5 | HMDB0008055 | 8.1 ± 3.0 | 7.9 ± 3.1 | NS |
| PC aa C40:6 | HMDB0008057 | 39.2 ± 12.8 | 31.6 ± 9.2 | 0.02 |
| PC aa C42:0 | HMDB0008058 | 0.5 ± 0.2 | 0.4 ± 0.2 | 0.016 |
| PC aa C42:1 | HMDB0008059 | 0.2 ± 0.1 | 0.2 ± 0.1 | NS |
| PC aa C42:5 | HMDB0008287 | 0.3 ± 0.1 | 0.3 ± 0.1 | 0.002 |
| PC aa C42:6 | HMDB0008288 | 0.6 ± 0.2 | 0.5 ± 0.1 | 0.003 |
| PC ae C30:0 | HMDB0013341 | 0.2 ± 0.1 | 0.2 ± 0.0 | NS |
| PC ae C32:1 | HMDB0007896 | 1.8 ± 0.5 | 1.7 ± 0.4 | NS |
| PC ae C32:2 | HMDB0013411 | 0.5 ± 0.1 | 0.4 ± 0.1 | NS |
| PC ae C34:0 | HMDB0013405 | 1.0 ± 0.3 | 0.9 ± 0.2 | NS |
| PC ae C34:1 | HMDB0013426 | 4.7 ± 1.2 | 4.3 ± 0.8 | NS |
| PC ae C34:2 | HMDB0011151 | 6.3 ± 2.1 | 5.4 ± 1.1 | 0.023 |
| PC ae C34:3 | HMDB0013413 | 4.7 ± 1.6 | 4.2 ± 1.2 | NS |
| PC ae C36:0 | HMDB0013406 | 0.9 ± 0.3 | 0.8 ± 0.3 | NS |
| PC ae C36:1 | HMDB0013414 | 4.5 ± 1.1 | 4.0 ± 0.8 | NS |
| PC ae C36:2 | HMDB0011243 | 7.8 ± 2.2 | 6.4 ± 1.4 | 0.005 |
| PC ae C36:3 | HMDB0013425 | 4.1 ± 1.2 | 3.7 ± 0.8 | NS |
| PC ae C36:4 | HMDB0013407 | 9.9 ± 3.2 | 9.1 ± 2.5 | NS |
| PC ae C36:5 | HMDB0011220 | 7.6 ± 2.3 | 7.1 ± 2.5 | NS |
| PC ae C38:0 | HMDB0013408 | 2.2 ± 0.7 | 1.7 ± 0.5 | 0.006 |
| PC ae C38:1 | HMDB0013408 | 0.6 ± 0.2 | 0.6 ± 0.2 | NS |
| PC ae C38:2 | HMDB0013416 | 1.1 ± 0.4 | 1.0 ± 0.3 | NS |
| PC ae C38:3 | HMDB0013431 | 2.0 ± 0.5 | 1.8 ± 0.4 | 0.044 |
| PC ae C38:4 | HMDB0013420 | 6.6 ± 1.7 | 5.9 ± 1.7 | NS |
| PC ae C38:5 | HMDB0011253 | 9.7 ± 2.4 | 8.6 ± 2.3 | 0.096 |
| PC ae C38:6 | HMDB0013409 | 6.1 ± 1.9 | 5.1 ± 1.6 | NS |
| PC ae C40:1 | HMDB0013433 | 1.0 ± 0.3 | 0.8 ± 0.2 | 0.083 |
| PC ae C40:2 | HMDB0013437 | 1.1 ± 0.3 | 0.9 ± 0.2 | 0.002 |
| PC ae C40:3 | HMDB0013445 | 0.5 ± 0.1 | 0.5 ± 0.1 | NS |
| PC ae C40:4 | HMDB0013442 | 1.2 ± 0.3 | 1.2 ± 0.3 | NS |
| PC ae C40:5 | HMDB0013444 | 2.2 ± 0.6 | 2.1 ± 0.6 | NS |
| PC ae C40:6 | HMDB0013422 | 4.2 ± 1.2 | 3.6 ± 1.1 | 0.046 |
| PC ae C42:2 | HMDB0013438 | 0.4 ± 0.1 | 0.4 ± 0.1 | NS |
| PC ae C42:3 | HMDB0013458 | 0.5 ± 0.1 | 0.4 ± 0.1 | NS |
| PC ae C42:4 | HMDB0013448 | 0.5 ± 0.2 | 0.4 ± 0.1 | NS |
| PC ae C42:5 | HMDB0013451 | 1.3 ± 0.3 | 1.2 ± 0.3 | NS |
| PC ae C44:5 | HMDB0013456 | 0.9 ± 0.3 | 0.9 ± 0.4 | NS |
| PC ae C44:6 | HMDB0013450 | 0.8 ± 0.2 | 0.8 ± 0.3 | NS |
| SM (OH) C14:1 | HMDB0013462 | 3.9 ± 1.1 | 3.1 ± 0.7 | 0.001 |
| SM (OH) C16:1 | HMDB0013463 | 2.8 ± 0.7 | 2.3 ± 0.6 | 0.003 |
| SM (OH) C22:1 | HMDB0013466 | 9.0 ± 2.0 | 7.0 ± 1.7 | <0.001 |
| SM (OH) C22:2 | HMDB0013467 | 8.0 ± 1.6 | 6.2 ± 1.5 | <0.001 |
| SM (OH) C24:1 | HMDB0013469 | 1.1 ± 0.4 | 0.8 ± 0.2 | 0.002 |
| SM C16:0 | HMDB0010169 | 95.4 ± 18.4 | 82.0 ± 10.9 | 0.001 |
| SM C16:1 | HMDB0029216 | 12.3 ± 2.5 | 10.5 ± 2.0 | 0.006 |
| SM C18:0 | HMDB0001348 | 20.3 ± 4.3 | 17.0 ± 4.0 | 0.006 |
| SM C18:1 | HMDB0012100 | 9.9 ± 2.5 | 8.3 ± 2.4 | 0.013 |
| SM C24:0 | HMDB0011697 | 15.7 ± 3.4 | 13.2 ± 2.9 | 0.006 |
| SM C24:1 | HMDB0012107 | 46.5 ± 10.1 | 38.3 ± 6.6 | 0.001 |
| H1 | HMDB0000122 | 1718.5 ± 967.1 | 4981.8 ± 817.2 | <0.001 |

| a, acyl residue; aa, diacyl residue; ae, acyl-alkyl, residue |  |  |
| --- | --- | --- |
| DMA, dimethylamine; t4-OH-Pro, trans-4-hydrox-proline; H1, hexoses (including glucose) | | |
| LysoPC, lysophosphatidylcholines; PC, phosphatidylcholines; SM, sphinogomyelins | | |

Supplementary Table 5. Concentration of measured analytes for early and advanced lung adenocarcinoma group

|  | Early LUAD | Advanced LUAD | P-value |
| --- | --- | --- | --- |
|  | (n=44) | (n=20) |  |
| T stage |  |  | <0.001 |
| T1 | 34 (77.3%) | 1 ( 5.0%) |  |
| T2 | 10 (22.7%) | 8 (40.0%) |  |
| T3 | 0 ( 0.0%) | 4 (20.0%) |  |
| T4 | 0 ( 0.0%) | 7 (35.0%) |  |
| N stage |  |  | <0.001 |
| N0 | 43 (97.7%) | 11 (55.0%) |  |
| N1-3 | 1 ( 2.3%) | 9 (45.0%) |  |
| M stage |  |  | 0.003 |
| M0 | 44 (100.0%) | 15 (75.0%) |  |
| M1 | 0 ( 0.0%) | 5 (25.0%) |  |
| TNM stage |  |  | <0.001 |
| Stage1 | 44 (100.0%) | 0 ( 0.0%) |  |
| Stage2 | 0 ( 0.0%) | 8 (40.0%) |  |
| Stage3 | 0 ( 0.0%) | 6 (30.0%) |  |
| Stage4 | 0 ( 0.0%) | 6 (30.0%) |  |
| Overall survival |  |  | 0.004 |
| Alive | 43 (97.7%) | 14 (70.0%) |  |
| Dead | 1 ( 2.3%) | 6 (30.0%) |  |
| Overall Survival days | 2114.4 ± 188.5 | 1796.0 ± 582 | 0.02 |

Supplementary Table 6. Receiver operation curve, sensitivity, specificity, positive predictive value, negative predictive value for diagnosis of lung adenocarcinoma

| Serial | Metabolite | ROC | 95% CI | cut-off value  (uMol) | Sensitivity | Specificity | PPV | NPV |
| --- | --- | --- | --- | --- | --- | --- | --- | --- |
| 1 | C0 | 0.843 | 0.7754-0.9103 | 28.5 | 90.6 | 71.8 | 72.5 | 90.3 |
| 2 | C2 | 0.520 | 0.4242-0.6149 | 6.32 | 60.9 | 50.0 | 50.0 | 60.9 |
| 3 | C3 | 0.752 | 0.6713-0.8317 | 0.22 | 78.1 | 65.4 | 64.9 | 78.5 |
| 4 | C4 | 0.753 | 0.6744-0.8312 | 0.11 | 75.0 | 62.8 | 62.3 | 75.4 |
| 5 | C10.1 | 0.753 | 0.6733-0.8331 | 0.2 | 70.3 | 70.5 | 66.2 | 74.3 |
| 6 | C12.1 | 0.668 | 0.579-0.7568 | 0.07 | 68.8 | 61.5 | 59.5 | 70.6 |
| 7 | C14 | 0.618 | 0.5299-0.7063 | 0.04 | 60.9 | 57.7 | 54.2 | 64.3 |
| 8 | C14.1 | 0.636 | 0.5452-0.7275 | 0.14 | 82.8 | 42.3 | 54.1 | 75.0 |
| 9 | C14.2 | 0.650 | 0.5599-0.74 | 0.06 | 73.4 | 53.8 | 56.6 | 71.2 |
| 10 | C18.1 | 0.751 | 0.6729-0.8299 | 0.14 | 82.8 | 56.4 | 60.9 | 80.0 |
| 11 | C18.2 | 0.769 | 0.6932-0.8442 | 0.09 | 96.9 | 46.2 | 59.6 | 94.7 |
| 12 | Ala | 0.648 | 0.5577-0.738 | 302 | 81.3 | 51.3 | 57.8 | 76.9 |
| 13 | Arg | 0.981 | 0.9608-1 | 54.6 | 90.6 | 97.4 | 96.7 | 92.7 |
| 14 | Asn | 0.854 | 0.7929-0.9154 | 37.1 | 85.9 | 75.6 | 74.3 | 86.8 |
| 15 | Asp | 0.814 | 0.7436-0.885 | 3.16 | 85.9 | 69.2 | 69.6 | 85.7 |
| 16 | Cit | 0.915 | 0.8696-0.9613 | 23.3 | 81.3 | 88.5 | 85.2 | 85.2 |
| 17 | Gln | 0.942 | 0.9055-0.9791 | 537 | 93.8 | 83.3 | 82.2 | 94.2 |
| 18 | Glu | 0.786 | 0.7114-0.8601 | 65.1 | 81.3 | 66.7 | 66.7 | 81.3 |
| 19 | Gly | 0.801 | 0.7296-0.8726 | 212 | 71.9 | 78.2 | 73.0 | 77.2 |
| 20 | His | 0.788 | 0.712-0.865 | 70.3 | 85.9 | 67.9 | 68.8 | 85.5 |
| 21 | Ile | 0.719 | 0.6346-0.8037 | 57 | 90.6 | 55.1 | 62.4 | 87.8 |
| 22 | Leu | 0.690 | 0.6015-0.7775 | 130 | 79.7 | 60.3 | 62.2 | 78.3 |
| 23 | Lys | 0.771 | 0.6917-0.8496 | 210 | 89.1 | 61.5 | 65.5 | 87.3 |
| 24 | Met | 0.837 | 0.7717-0.9021 | 18.9 | 96.9 | 62.8 | 68.1 | 96.1 |
| 25 | Orn | 0.622 | 0.529-0.7142 | 104 | 96.9 | 33.3 | 54.4 | 92.9 |
| 26 | Phe | 0.779 | 0.7032-0.8551 | 58.2 | 67.2 | 80.8 | 74.1 | 75.0 |
| 27 | Pro | 0.797 | 0.7233-0.8715 | 104 | 89.1 | 61.5 | 65.5 | 87.3 |
| 28 | Ser | 0.700 | 0.6142-0.785 | 98.5 | 81.3 | 55.1 | 59.8 | 78.2 |
| 29 | Thr | 0.809 | 0.7397-0.8791 | 99.9 | 79.7 | 73.1 | 70.8 | 81.4 |
| 30 | Trp | 0.589 | 0.4942-0.6829 | 36.9 | 76.6 | 47.4 | 54.4 | 71.2 |
| 31 | Tyr | 0.818 | 0.7485-0.8869 | 51.1 | 81.3 | 73.1 | 71.2 | 82.6 |
| 32 | Val | 0.744 | 0.6611-0.8261 | 215 | 71.9 | 74.4 | 69.7 | 76.3 |
| 33 | Creatinine | 0.736 | 0.6546-0.8184 | 45.1 | 85.9 | 57.7 | 62.5 | 83.3 |
| 34 | Kynurenine | 0.638 | 0.5468-0.7284 | 1.19 | 96.9 | 35.9 | 55.4 | 93.3 |
| 35 | Putrescine | 0.572 | 0.4776-0.6668 | 0.08 | 56.3 | 61.5 | 54.5 | 63.2 |
| 36 | Spermine | 0.714 | 0.6311-0.7974 | 0.29 | 93.8 | 41.0 | 56.6 | 88.9 |
| 37 | t4.OH.Pro | 0.750 | 0.6694-0.8302 | 5.88 | 76.6 | 66.7 | 65.3 | 77.6 |
| 38 | Taurine | 0.525 | 0.4285-0.6214 | 115 | 95.3 | 28.2 | 52.1 | 88.0 |
| 39 | total.DMA | 0.847 | 0.7814-0.9117 | 0.58 | 78.1 | 80.8 | 76.9 | 81.8 |
| 40 | lysoPC.a.C16.0 | 0.697 | 0.6081-0.7851 | 78.7 | 95.3 | 46.2 | 59.2 | 92.3 |
| 41 | lysoPC.a.C16.1 | 0.533 | 0.4372-0.6283 | 2.6 | 96.9 | 23.1 | 50.8 | 90.0 |
| 42 | lysoPC.a.C17.0 | 0.632 | 0.5395-0.7241 | 1.61 | 93.8 | 38.5 | 55.6 | 88.2 |
| 43 | lysoPC.a.C18.0 | 0.679 | 0.5903-0.7679 | 21.3 | 75.0 | 62.8 | 62.3 | 75.4 |
| 44 | lysoPC.a.C18.1 | 0.550 | 0.4539-0.6456 | 11.6 | 79.7 | 42.3 | 53.1 | 71.7 |
| 45 | lysoPC.a.C18.2 | 0.538 | 0.4426-0.6331 | 14.5 | 54.7 | 57.7 | 51.5 | 60.8 |
| 46 | lysoPC.a.C20.3 | 0.551 | 0.4558-0.646 | 0.74 | 75.0 | 42.3 | 51.6 | 67.3 |
| 47 | lysoPC.a.C20.4 | 0.652 | 0.5616-0.7421 | 3.86 | 75.0 | 53.8 | 57.1 | 72.4 |
| 48 | PC.aa.C28.1 | 0.626 | 0.5341-0.7183 | 1.2 | 82.8 | 42.3 | 54.1 | 75.0 |
| 49 | PC.aa.C30.0 | 0.734 | 0.6516-0.8156 | 1.62 | 78.1 | 60.3 | 61.7 | 77.0 |
| 50 | PC.aa.C32.0 | 0.747 | 0.6653-0.8283 | 7.92 | 65.6 | 78.2 | 71.2 | 73.5 |
| 51 | PC.aa.C32.1 | 0.800 | 0.7279-0.8716 | 5.92 | 73.4 | 74.4 | 70.1 | 77.3 |
| 52 | PC.aa.C32.2 | 0.750 | 0.6699-0.8301 | 1.06 | 87.5 | 53.8 | 60.9 | 84.0 |
| 53 | PC.aa.C32.3 | 0.714 | 0.6308-0.7977 | 0.17 | 82.8 | 52.6 | 58.9 | 78.8 |
| 54 | PC.aa.C34.1 | 0.725 | 0.642-0.8089 | 96.9 | 84.4 | 57.7 | 62.1 | 81.8 |
| 55 | PC.aa.C34.2 | 0.641 | 0.5504-0.7319 | 185 | 60.9 | 64.1 | 58.2 | 66.7 |
| 56 | PC.aa.C34.3 | 0.826 | 0.7581-0.8948 | 6.48 | 90.6 | 66.7 | 69.0 | 89.7 |
| 57 | PC.aa.C34.4 | 0.705 | 0.62-0.7899 | 0.53 | 79.7 | 55.1 | 59.3 | 76.8 |
| 58 | PC.aa.C36.0 | 0.702 | 0.6156-0.7891 | 2.56 | 79.7 | 60.3 | 62.2 | 78.3 |
| 59 | PC.aa.C36.1 | 0.718 | 0.6342-0.8011 | 24.6 | 75.0 | 61.5 | 61.5 | 75.0 |
| 60 | PC.aa.C36.2 | 0.659 | 0.5698-0.7485 | 106 | 78.1 | 48.7 | 55.6 | 73.1 |
| 61 | PC.aa.C36.3 | 0.666 | 0.578-0.7547 | 40.1 | 95.3 | 35.9 | 55.0 | 90.3 |
| 62 | PC.aa.C36.4 | 0.582 | 0.4877-0.6759 | 76.7 | 78.1 | 42.3 | 52.6 | 70.2 |
| 63 | PC.aa.C36.5 | 0.716 | 0.6306-0.8019 | 19 | 81.3 | 64.1 | 65.0 | 80.6 |
| 64 | PC.aa.C36.6 | 0.724 | 0.6406-0.8073 | 0.53 | 92.2 | 48.7 | 59.6 | 88.4 |
| 65 | PC.aa.C38.0 | 0.687 | 0.6001-0.7741 | 2.26 | 71.9 | 59.0 | 59.0 | 71.9 |
| 66 | PC.aa.C38.1 | 0.660 | 0.5686-0.7513 | 0.9 | 59.4 | 71.8 | 63.3 | 68.3 |
| 67 | PC.aa.C38.3 | 0.715 | 0.6314-0.7993 | 22.2 | 71.9 | 65.4 | 63.0 | 73.9 |
| 68 | PC.aa.C38.4 | 0.622 | 0.5292-0.714 | 58.4 | 65.6 | 59.0 | 56.8 | 67.6 |
| 69 | PC.aa.C38.5 | 0.713 | 0.6285-0.7983 | 25.5 | 93.8 | 48.7 | 60.0 | 90.5 |
| 70 | PC.aa.C38.6 | 0.692 | 0.6055-0.7787 | 55.2 | 87.5 | 48.7 | 58.3 | 82.6 |
| 71 | PC.aa.C40.4 | 0.652 | 0.5621-0.7424 | 1.18 | 90.6 | 42.3 | 56.3 | 84.6 |
| 72 | PC.aa.C40.5 | 0.733 | 0.6502-0.8165 | 6.72 | 64.1 | 75.6 | 68.3 | 72.0 |
| 73 | PC.aa.C40.6 | 0.745 | 0.6636-0.8259 | 24.2 | 85.9 | 60.3 | 64.0 | 83.9 |
| 74 | PC.aa.C42.0 | 0.726 | 0.6398-0.8114 | 0.39 | 76.6 | 67.9 | 66.2 | 77.9 |
| 75 | PC.aa.C42.1 | 0.737 | 0.6545-0.8203 | 0.19 | 79.7 | 62.8 | 63.8 | 79.0 |
| 76 | PC.aa.C42.5 | 0.689 | 0.6012-0.7762 | 0.18 | 89.1 | 46.2 | 57.6 | 83.7 |
| 77 | PC.aa.C42.6 | 0.753 | 0.6721-0.8335 | 0.44 | 67.2 | 76.9 | 70.5 | 74.1 |
| 78 | PC.ae.C30.0 | 0.659 | 0.5671-0.7504 | 0.17 | 56.3 | 76.9 | 66.7 | 68.2 |
| 79 | PC.ae.C32.1 | 0.675 | 0.5851-0.7643 | 1.58 | 68.8 | 65.4 | 62.0 | 71.8 |
| 80 | PC.ae.C32.2 | 0.700 | 0.6123-0.787 | 0.41 | 68.8 | 70.5 | 65.7 | 73.3 |
| 81 | PC.ae.C34.0 | 0.717 | 0.6336-0.8001 | 0.84 | 65.6 | 66.7 | 61.8 | 70.3 |
| 82 | PC.ae.C34.1 | 0.673 | 0.5839-0.7625 | 3.89 | 78.1 | 57.7 | 60.2 | 76.3 |
| 83 | PC.ae.C34.2 | 0.569 | 0.4736-0.6634 | 4.69 | 81.3 | 39.7 | 52.5 | 72.1 |
| 84 | PC.ae.C34.3 | 0.505 | 0.3994-0.591 | 5.3 | 81.3 | 32.1 | 49.5 | 67.6 |
| 85 | PC.ae.C36.0 | 0.730 | 0.6475-0.8124 | 0.56 | 87.5 | 51.3 | 59.6 | 83.3 |
| 86 | PC.ae.C36.1 | 0.652 | 0.5609-0.7424 | 3.31 | 87.5 | 47.4 | 57.7 | 82.2 |
| 87 | PC.ae.C36.2 | 0.626 | 0.5336-0.718 | 7.01 | 59.4 | 67.9 | 60.3 | 67.1 |
| 88 | PC.ae.C36.3 | 0.572 | 0.4772-0.6672 | 3.15 | 79.7 | 43.6 | 53.7 | 72.3 |
| 89 | PC.ae.C36.4 | 0.517 | 0.4219-0.6126 | 13.7 | 89.1 | 20.5 | 47.9 | 69.6 |
| 90 | PC.ae.C36.5 | 0.578 | 0.484-0.6724 | 7.28 | 48.4 | 67.9 | 55.4 | 61.6 |
| 91 | PC.ae.C38.0 | 0.707 | 0.6217-0.7914 | 1.41 | 81.3 | 56.4 | 60.5 | 78.6 |
| 92 | PC.ae.C38.1 | 0.740 | 0.6572-0.8238 | 0.46 | 81.3 | 65.4 | 65.8 | 81.0 |
| 93 | PC.ae.C38.2 | 0.720 | 0.6358-0.8045 | 0.89 | 73.4 | 67.9 | 65.3 | 75.7 |
| 94 | PC.ae.C38.3 | 0.631 | 0.5397-0.7231 | 1.79 | 67.2 | 59.0 | 57.3 | 68.7 |
| 95 | PC.ae.C38.4 | 0.491 | 0.4127-0.6048 | 8.33 | 68.8 | 44.9 | 50.6 | 63.6 |
| 96 | PC.ae.C38.5 | 0.559 | 0.4645-0.6542 | 7.53 | 78.1 | 38.5 | 51.0 | 68.2 |
| 97 | PC.ae.C38.6 | 0.653 | 0.562-0.7433 | 5.37 | 59.4 | 69.2 | 61.3 | 67.5 |
| 98 | PC.ae.C40.1 | 0.619 | 0.5263-0.7109 | 0.9 | 51.6 | 70.5 | 58.9 | 64.0 |
| 99 | PC.ae.C40.2 | 0.669 | 0.5808-0.758 | 0.95 | 59.4 | 67.9 | 60.3 | 67.1 |
| 100 | PC.ae.C40.3 | 0.597 | 0.5033-0.6898 | 0.51 | 57.8 | 60.3 | 54.4 | 63.5 |
| 101 | PC.ae.C40.4 | 0.594 | 0.5004-0.6881 | 0.93 | 85.9 | 35.9 | 52.4 | 75.7 |
| 102 | PC.ae.C40.5 | 0.659 | 0.5689-0.7484 | 1.69 | 84.4 | 41.0 | 54.0 | 76.2 |
| 103 | PC.ae.C40.6 | 0.689 | 0.6006-0.7764 | 3.59 | 64.1 | 71.8 | 65.1 | 70.9 |
| 104 | PC.ae.C42.2 | 0.723 | 0.6376-0.8075 | 0.28 | 79.7 | 57.7 | 60.7 | 77.6 |
| 105 | PC.ae.C42.3 | 0.621 | 0.5291-0.7137 | 0.33 | 90.6 | 32.1 | 52.3 | 80.6 |
| 106 | PC.ae.C42.4 | 0.548 | 0.4525-0.6429 | 0.29 | 92.2 | 29.5 | 51.8 | 82.1 |
| 107 | PC.ae.C42.5 | 0.588 | 0.4936-0.6827 | 1.15 | 65.6 | 57.7 | 56.0 | 67.2 |
| 108 | PC.ae.C44.5 | 0.513 | 0.3904-0.5835 | 0.66 | 81.3 | 32.1 | 49.5 | 67.6 |
| 109 | PC.ae.C44.6 | 0.631 | 0.5389-0.7225 | 0.57 | 92.2 | 34.6 | 53.6 | 84.4 |
| 110 | SM.OH.C14.1 | 0.615 | 0.5219-0.7076 | 3.34 | 62.5 | 64.1 | 58.8 | 67.6 |
| 111 | SM.OH.C16.1 | 0.600 | 0.5063-0.6934 | 3.17 | 28.1 | 88.5 | 66.7 | 60.0 |
| 112 | SM.OH.C22.1 | 0.603 | 0.5091-0.6966 | 7.66 | 64.1 | 59.0 | 56.2 | 66.7 |
| 113 | SM.OH.C22.2 | 0.645 | 0.553-0.7373 | 6.89 | 65.6 | 69.2 | 63.6 | 71.1 |
| 114 | SM.OH.C24.1 | 0.630 | 0.5378-0.722 | 1.07 | 40.6 | 82.1 | 65.0 | 62.7 |
| 115 | SM.C16.0 | 0.702 | 0.6161-0.7873 | 71.5 | 92.2 | 43.6 | 57.3 | 87.2 |
| 116 | SM.C16.1 | 0.665 | 0.5768-0.7538 | 8.82 | 90.6 | 38.5 | 54.7 | 83.3 |
| 117 | SM.C18.0 | 0.559 | 0.4642-0.654 | 13.5 | 95.3 | 23.1 | 50.4 | 85.7 |
| 118 | SM.C18.1 | 0.556 | 0.4605-0.6509 | 7.17 | 78.1 | 38.5 | 51.0 | 68.2 |
| 119 | SM.C24.0 | 0.643 | 0.5534-0.7335 | 12.4 | 76.6 | 50.0 | 55.7 | 72.2 |
| 120 | SM.C24.1 | 0.698 | 0.6125-0.784 | 37.8 | 76.6 | 57.7 | 59.8 | 75.0 |

Supplementary Table 7. Network parameters for healthy control group

| Average Shortest  PathLength | Betweenness Centrality | Closeness Centrality | Clustering Coefficient | Degree | Eccentricity | Metabolite | Neighborhood Connectivity | Number Of  Undirected Edges |
| --- | --- | --- | --- | --- | --- | --- | --- | --- |
| 2.99047619 | 0.00098404 | 0.3343949 | 0.8421053 | 19 | 9 | PC.ae.C34.2 | 30.57894737 | 19 |
| 3.00952381 | 0.000868982 | 0.33227848 | 0.8496732 | 18 | 9 | PC.ae.C36.3 | 30.27777778 | 18 |
| 2.685714286 | 0.007834392 | 0.37234043 | 0.7396825 | 36 | 9 | PC.aa.C38.0 | 31.69444444 | 36 |
| 2.723809524 | 0.004390768 | 0.36713287 | 0.7903226 | 32 | 9 | PC.ae.C38.6 | 32.4375 | 32 |
| 2.704761905 | 0.005708477 | 0.36971831 | 0.7664884 | 34 | 9 | PC.ae.C40.5 | 32.08823529 | 34 |
| 2.895238095 | 0.001181036 | 0.34539474 | 0.8275862 | 29 | 9 | PC.ae.C38.4 | 32.10344828 | 29 |
| 2.857142857 | 0.001741275 | 0.35 | 0.7992424 | 33 | 9 | PC.ae.C38.5 | 31.84848485 | 33 |
| 2.742857143 | 0.003832552 | 0.36458333 | 0.7954023 | 30 | 9 | PC.ae.C40.6 | 32.26666667 | 30 |
| 4.552380952 | 0.000142377 | 0.21966527 | 0.8611111 | 9 | 7 | Leu | 10.44444444 | 9 |
| 4.561904762 | 4.90581E-05 | 0.21920668 | 0.9285714 | 8 | 7 | Val | 10.875 | 8 |
| 4.561904762 | 0.000725482 | 0.21920668 | 0.7857143 | 8 | 7 | Ile | 10 | 8 |
| 3 | 0.001421456 | 0.33333333 | 0.8362573 | 19 | 9 | PC.ae.C34.3 | 29.78947368 | 19 |
| 1.857142857 | 0 | 0.53846154 | 1 | 3 | 3 | C14.1 | 3.666666667 | 3 |
| 1.857142857 | 0 | 0.53846154 | 1 | 3 | 3 | C14.2 | 3.666666667 | 3 |
| 2.79047619 | 0.005927092 | 0.35836177 | 0.7878788 | 22 | 9 | PC.aa.C36.0 | 30.72727273 | 22 |
| 3.257142857 | 0.001666866 | 0.30701754 | 0.8666667 | 6 | 10 | PC.aa.C36.4 | 16.16666667 | 6 |
| 3.247619048 | 0.001770651 | 0.30791789 | 0.8095238 | 7 | 10 | PC.aa.C38.4 | 16.14285714 | 7 |
| 2.752380952 | 0.0050621 | 0.3633218 | 0.7312312 | 37 | 9 | SM.OH.C14.1 | 31 | 37 |
| 2.8 | 0.003267104 | 0.35714286 | 0.7825312 | 34 | 9 | SM.OH.C16.1 | 31.88235294 | 34 |
| 2.542857143 | 0.028206161 | 0.39325843 | 0.71266 | 38 | 8 | SM.C16.0 | 30.34210526 | 38 |
| 2.419047619 | 0.044293848 | 0.41338583 | 0.6692308 | 40 | 8 | SM.C16.1 | 29.925 | 40 |
| 3.142857143 | 0.01319926 | 0.31818182 | 0.6 | 16 | 10 | PC.aa.C38.6 | 17.4375 | 16 |
| 3.304761905 | 0.002552187 | 0.30259366 | 0.8030303 | 12 | 10 | PC.aa.C40.6 | 15.16666667 | 12 |
| 2.714285714 | 0.006368237 | 0.36842105 | 0.7282282 | 37 | 9 | PC.ae.C36.5 | 31.32432432 | 37 |
| 2.80952381 | 0.003463607 | 0.3559322 | 0.7507937 | 36 | 9 | PC.ae.C40.3 | 31.05555556 | 36 |
| 3.180952381 | 0.009579423 | 0.31437126 | 0.6923077 | 14 | 10 | PC.aa.C38.5 | 14.71428571 | 14 |
| 2.80952381 | 0.018378757 | 0.3559322 | 0.5714286 | 14 | 9 | PC.aa.C34.2 | 24.5 | 14 |
| 2.819047619 | 0.014216289 | 0.35472973 | 0.530303 | 12 | 9 | PC.aa.C36.2 | 18.08333333 | 12 |
| 2.676190476 | 0.007618424 | 0.37366548 | 0.7537538 | 37 | 9 | PC.ae.C40.4 | 31.97297297 | 37 |
| 2.733333333 | 0.007329349 | 0.36585366 | 0.7344029 | 34 | 9 | PC.ae.C40.2 | 31.08823529 | 34 |
| 2.828571429 | 0.003191095 | 0.35353535 | 0.752381 | 36 | 9 | PC.ae.C42.5 | 31 | 36 |
| 2.847619048 | 0.001822652 | 0.35117057 | 0.7932264 | 34 | 9 | PC.ae.C32.1 | 31.91176471 | 34 |
| 2.619047619 | 0.01425624 | 0.38181818 | 0.6512195 | 41 | 9 | PC.ae.C34.1 | 30 | 41 |
| 3.4 | 0.001904451 | 0.29411765 | 0.8727273 | 11 | 10 | PC.aa.C42.6 | 15 | 11 |
| 3.095238095 | 0.018382036 | 0.32307692 | 0.4117647 | 18 | 10 | PC.aa.C34.1 | 12.77777778 | 18 |
| 3.076190476 | 0.015579858 | 0.3250774 | 0.4485294 | 17 | 10 | PC.aa.C36.1 | 14.35294118 | 17 |
| 3.2 | 0.00619064 | 0.3125 | 0.7307692 | 13 | 10 | PC.aa.C42.5 | 14.38461538 | 13 |
| 2.895238095 | 0.00176877 | 0.34539474 | 0.7980296 | 29 | 9 | PC.ae.C42.4 | 30.86206897 | 29 |
| 3.152380952 | 0.010235967 | 0.31722054 | 0.6333333 | 16 | 10 | PC.ae.C38.0 | 17.125 | 16 |
| 2.704761905 | 0.013724848 | 0.36971831 | 0.8184615 | 26 | 8 | SM.C18.0 | 31.5 | 26 |
| 2.838095238 | 0.001446945 | 0.35234899 | 0.8295455 | 33 | 9 | SM.C18.1 | 32.6969697 | 33 |
| 2.533333333 | 0.028737923 | 0.39473684 | 0.7333333 | 36 | 8 | SM.OH.C22.2 | 30.86111111 | 36 |
| 3.295238095 | 0.006857502 | 0.30346821 | 0.6373626 | 14 | 10 | PC.aa.C40.5 | 13.64285714 | 14 |
| 2.761904762 | 0.018558358 | 0.36206897 | 0.6892308 | 26 | 9 | PC.ae.C34.0 | 29.92307692 | 26 |
| 2.819047619 | 0.022853804 | 0.35472973 | 0.5842105 | 20 | 9 | PC.ae.C36.0 | 24.7 | 20 |
| 3.038095238 | 0.000529401 | 0.32915361 | 0.8684211 | 20 | 9 | PC.ae.C36.4 | 30.55 | 20 |
| 3.019047619 | 0.020317678 | 0.33123028 | 0.5 | 12 | 10 | PC.aa.C40.4 | 15.83333333 | 12 |
| 2.780952381 | 0.002461162 | 0.35958904 | 0.8338462 | 26 | 9 | PC.aa.C42.0 | 33.11538462 | 26 |
| 3.79047619 | 0.007889124 | 0.2638191 | 0.7777778 | 10 | 7 | Trp | 10.5 | 10 |
| 3.771428571 | 0.008173951 | 0.26515152 | 0.7424242 | 12 | 7 | Tyr | 10.08333333 | 12 |
| 3.771428571 | 0.008173951 | 0.26515152 | 0.7424242 | 12 | 7 | Phe | 10.08333333 | 12 |
| 2.876190476 | 0.010095546 | 0.34768212 | 0.8380952 | 21 | 8 | SM.C24.1 | 31.23809524 | 21 |
| 2.666666667 | 0.010519545 | 0.375 | 0.6770982 | 38 | 9 | PC.ae.C32.2 | 30.60526316 | 38 |
| 3.057142857 | 0.000638586 | 0.3271028 | 0.8586957 | 24 | 9 | PC.ae.C44.6 | 31.45833333 | 24 |
| 2.80952381 | 0.008703377 | 0.3559322 | 0.7010753 | 31 | 9 | PC.ae.C36.2 | 30 | 31 |
| 3.057142857 | 0.001601736 | 0.3271028 | 0.8092308 | 26 | 9 | PC.aa.C42.1 | 31.5 | 26 |
| 2.619047619 | 0.063440003 | 0.38181818 | 0.5132275 | 28 | 9 | PC.ae.C42.2 | 25.92857143 | 28 |
| 2.285714286 | 0 | 0.4375 | 1 | 2 | 3 | C18.1 | 2.5 | 2 |
| 2.285714286 | 0 | 0.4375 | 1 | 2 | 3 | C18.2 | 2.5 | 2 |
| 2.4 | 0.068553307 | 0.41666667 | 0.7142857 | 28 | 8 | PC.aa.C28.1 | 31.57142857 | 28 |
| 2.333333333 | 0.071643 | 0.42857143 | 0.6730159 | 36 | 8 | PC.ae.C36.1 | 30.77777778 | 36 |
| 2.304761905 | 0.092321246 | 0.4338843 | 0.6072874 | 39 | 8 | PC.ae.C38.3 | 29.51282051 | 39 |
| 3.066666667 | 0.313934276 | 0.32608696 | 0.6888889 | 10 | 6 | His | 10.9 | 10 |
| 2.561904762 | 0.075513756 | 0.39033457 | 0.421371 | 32 | 9 | PC.aa.C32.0 | 24.15625 | 32 |
| 2.542857143 | 0.038814615 | 0.39325843 | 0.6651652 | 37 | 9 | PC.ae.C42.3 | 30.94594595 | 37 |
| 2.552380952 | 0.018610105 | 0.39179104 | 0.7741935 | 32 | 8 | SM.OH.C22.1 | 31.71875 | 32 |
| 2.952380952 | 0.001529641 | 0.33870968 | 0.8 | 6 | 8 | SM.OH.C24.1 | 30.66666667 | 6 |
| 2.638095238 | 0.105677725 | 0.37906137 | 0.347619 | 21 | 9 | PC.ae.C40.1 | 17.9047619 | 21 |
| 1.285714286 | 0.714285714 | 0.77777778 | 0.3 | 5 | 2 | C12.1 | 2.6 | 5 |
| 2.59047619 | 0.323304823 | 0.38602941 | 0.6 | 11 | 7 | SM.C24.0 | 28.36363636 | 11 |
| 3.647619048 | 0.043684583 | 0.27415144 | 0.5824176 | 14 | 6 | Lys | 9.357142857 | 14 |
| 3.285714286 | 0.00010427 | 0.30434783 | 0.8939394 | 12 | 9 | PC.ae.C44.5 | 29.25 | 12 |
| 3.952380952 | 0 | 0.25301205 | 1 | 9 | 11 | PC.aa.C36.6 | 13.22222222 | 9 |
| 3.20952381 | 0.007270829 | 0.3115727 | 0.6923077 | 14 | 10 | PC.ae.C38.1 | 16.92857143 | 14 |
| 3.819047619 | 0.016272242 | 0.26184539 | 0.6666667 | 12 | 7 | C0 | 9.5 | 12 |
| 3.952380952 | 0 | 0.25301205 | 1 | 9 | 11 | PC.aa.C36.5 | 13.22222222 | 9 |
| 3.371428571 | 0.014636388 | 0.29661017 | 0.5714286 | 8 | 10 | PC.aa.C34.3 | 12 | 8 |
| 3.523809524 | 0.005676604 | 0.28378378 | 0.5714286 | 7 | 10 | PC.aa.C34.4 | 10.42857143 | 7 |
| 3.171428571 | 0.000219868 | 0.31531532 | 0.8888889 | 10 | 9 | PC.aa.C38.1 | 29.6 | 10 |
| 6.466666667 | 0.037728938 | 0.15463918 | 0 | 2 | 9 | Putrescine | 2 | 2 |
| 7.438095238 | 0.019047619 | 0.13444302 | 0 | 2 | 10 | Taurine | 1.5 | 2 |
| 1.857142857 | 0 | 0.53846154 | 1 | 3 | 3 | C2 | 3.666666667 | 3 |
| 3.666666667 | 0.099361645 | 0.27272727 | 0.5777778 | 10 | 6 | Asn | 9.4 | 10 |
| 3.676190476 | 0.057018214 | 0.27202073 | 0.5777778 | 10 | 6 | Met | 9.8 | 10 |
| 4.085714286 | 0.019047619 | 0.24475524 | 0.5 | 4 | 10 | lysoPC.a.C16.0 | 4 | 4 |
| 3.371428571 | 0.041536011 | 0.29661017 | 0.4 | 5 | 10 | lysoPC.a.C18.0 | 8.4 | 5 |
| 1.571428571 | 0.476190476 | 0.63636364 | 0.3333333 | 3 | 2 | C14 | 3 | 3 |
| 3.885714286 | 0.004220769 | 0.25735294 | 0.5 | 4 | 10 | lysoPC.a.C18.1 | 6.25 | 4 |
| 2.828571429 | 0.002038613 | 0.35353535 | 0.6818182 | 12 | 9 | PC.ae.C38.2 | 26.16666667 | 12 |
| 2.752380952 | 0.002883228 | 0.3633218 | 0.8266129 | 32 | 9 | PC.ae.C30.0 | 33.125 | 32 |
| 5.180952381 | 0.001007594 | 0.19301471 | 0.6666667 | 3 | 8 | C3 | 4.666666667 | 3 |
| 5.428571429 | 0 | 0.18421053 | 1 | 2 | 8 | Pro | 4.5 | 2 |
| 3.647619048 | 0.071440237 | 0.27415144 | 0.2666667 | 6 | 9 | lysoPC.a.C16.1 | 5.333333333 | 6 |
| 3.428571429 | 0.032123632 | 0.29166667 | 0.5 | 9 | 10 | PC.aa.C32.1 | 12.11111111 | 9 |
| 4.180952381 | 0.033306074 | 0.23917995 | 0.5333333 | 6 | 6 | Thr | 8.166666667 | 6 |
| 4.342857143 | 0 | 0.23026316 | 1 | 2 | 11 | PC.aa.C32.2 | 7.5 | 2 |
| 3.257142857 | 0.019366394 | 0.30701754 | 0.4444444 | 10 | 10 | PC.aa.C36.3 | 12 | 10 |
| 3.485714286 | 0.013505606 | 0.28688525 | 0.4285714 | 7 | 10 | PC.aa.C38.3 | 9.142857143 | 7 |
| 4.447619048 | 0.027773869 | 0.2248394 | 0.4666667 | 6 | 7 | Orn | 6.833333333 | 6 |
| 2.933333333 | 0.002445749 | 0.34090909 | 0.6515152 | 12 | 9 | PC.aa.C32.3 | 25.08333333 | 12 |
| 2.942857143 | 0.012107637 | 0.33980583 | 0.5277778 | 9 | 9 | PC.aa.C30.0 | 17.66666667 | 9 |
| 3.828571429 | 0.002229318 | 0.26119403 | 0.8928571 | 8 | 7 | total.DMA | 11.125 | 8 |
| 4.514285714 | 0.045119686 | 0.22151899 | 0.3333333 | 3 | 7 | Ala | 5 | 3 |
| 4.276190476 | 0.034676889 | 0.23385301 | 0.3333333 | 6 | 7 | Cit | 4.833333333 | 6 |
| 3.80952381 | 0.013748872 | 0.2625 | 0.6888889 | 10 | 7 | Creatinine | 9.8 | 10 |
| 5.076190476 | 0 | 0.19699812 | 0 | 1 | 11 | lysoPC.a.C20.4 | 4 | 1 |
| 5.514285714 | 0.056043956 | 0.18134715 | 0 | 2 | 8 | Kynurenine | 2 | 2 |
| 5.114285714 | 0 | 0.19553073 | 0 | 1 | 9 | Glu | 3 | 1 |
| 4.123809524 | 0.06290901 | 0.24249423 | 0 | 3 | 8 | lysoPC.a.C20.3 | 3.333333333 | 3 |
| 4.733333333 | 2.61643E-05 | 0.21126761 | 0.6666667 | 3 | 8 | C4 | 10 | 3 |
| 4.580952381 | 0.073992674 | 0.21829522 | 0 | 2 | 7 | Gln | 6 | 2 |
| 8.428571429 | 0 | 0.11864407 | 0 | 1 | 11 | Spermine | 2 | 1 |
| 2.142857143 | 0 | 0.46666667 | 0 | 1 | 3 | C10.1 | 5 | 1 |
| 5.266666667 | 0 | 0.18987342 | 0 | 1 | 8 | Arg | 6 | 1 |

Supplementary Table 8. Correlation coefficient of nodes from healthy control group

| Correlation  coefficient | Edge Betweenness | Analytes |
| --- | --- | --- |
| 0.989867051 | 2.163636364 | PC.ae.C34.2 (interacts with) PC.ae.C36.3 |
| 0.988579882 | 2.438596491 | PC.aa.C38.0 (interacts with) PC.ae.C38.6 |
| 0.98791637 | 2.205263158 | PC.ae.C38.6 (interacts with) PC.ae.C40.5 |
| 0.982601743 | 2.383128771 | PC.ae.C38.4 (interacts with) PC.ae.C38.5 |
| 0.982429039 | 2.399667261 | PC.ae.C40.5 (interacts with) PC.ae.C40.6 |
| 0.982091194 | 2.333333333 | Leu (interacts with) Val |
| 0.981751427 | 2.952380952 | Ile (interacts with) Val |
| 0.981279697 | 3.157086489 | PC.ae.C34.3 (interacts with) PC.ae.C36.3 |
| 0.981213842 | 2.653635515 | PC.aa.C38.0 (interacts with) PC.ae.C40.6 |
| 0.980828695 | 2.212885154 | PC.aa.C38.0 (interacts with) PC.ae.C40.5 |
| 0.979934919 | 2 | C14.1 (interacts with) C14.2 |
| 0.979396974 | 2.983926316 | PC.ae.C34.2 (interacts with) PC.ae.C34.3 |
| 0.979391882 | 4.421323588 | PC.aa.C36.0 (interacts with) PC.ae.C38.6 |
| 0.979295913 | 2.170909091 | PC.ae.C38.6 (interacts with) PC.ae.C40.6 |
| 0.978238405 | 2.4 | PC.aa.C36.4 (interacts with) PC.aa.C38.4 |
| 0.977753561 | 2.430588791 | SM.OH.C14.1 (interacts with) SM.OH.C16.1 |
| 0.977738006 | 3.137785413 | SM.C16.0 (interacts with) SM.C16.1 |
| 0.977191753 | 5.695121991 | PC.aa.C38.6 (interacts with) PC.aa.C40.6 |
| 0.973816115 | 3.352380952 | Ile (interacts with) Leu |
| 0.973149806 | 3.793904481 | PC.ae.C36.5 (interacts with) PC.ae.C38.5 |
| 0.971803382 | 9.243086976 | PC.ae.C40.3 (interacts with) SM.C16.1 |
| 0.971091275 | 5.399047619 | PC.aa.C38.5 (interacts with) PC.aa.C40.6 |
| 0.970710501 | 12.04292967 | PC.aa.C34.2 (interacts with) PC.aa.C36.2 |
| 0.970494346 | 5.277389014 | PC.ae.C38.5 (interacts with) PC.ae.C40.4 |
| 0.969658092 | 3.158489316 | PC.ae.C40.2 (interacts with) PC.ae.C40.6 |
| 0.969347674 | 4.983548907 | PC.ae.C40.4 (interacts with) PC.ae.C42.5 |
| 0.968901297 | 5.453952353 | PC.ae.C32.1 (interacts with) PC.ae.C34.1 |
| 0.966787229 | 9.717985807 | PC.aa.C38.5 (interacts with) PC.aa.C42.6 |
| 0.966584875 | 4.691650338 | PC.aa.C34.1 (interacts with) PC.aa.C36.1 |
| 0.966247195 | 2.28708134 | PC.ae.C32.1 (interacts with) PC.ae.C42.5 |
| 0.966194155 | 4.099391019 | PC.aa.C36.0 (interacts with) PC.ae.C40.6 |
| 0.966078915 | 10.1148112 | PC.aa.C42.5 (interacts with) PC.aa.C42.6 |
| 0.965663889 | 2.733676262 | PC.ae.C42.4 (interacts with) PC.ae.C42.5 |
| 0.964702134 | 9.168100717 | PC.aa.C42.6 (interacts with) PC.ae.C38.0 |
| 0.964154556 | 5.725540745 | PC.aa.C36.0 (interacts with) PC.aa.C38.0 |
| 0.963726783 | 9.367471805 | SM.C18.0 (interacts with) SM.C18.1 |
| 0.963446158 | 5.027902907 | PC.aa.C36.0 (interacts with) PC.ae.C40.5 |
| 0.963325236 | 3.091857248 | SM.OH.C22.2 (interacts with) SM.C16.0 |
| 0.9629078 | 5.300735249 | PC.aa.C38.5 (interacts with) PC.aa.C40.5 |
| 0.962814193 | 8.063492063 | PC.ae.C40.3 (interacts with) SM.C16.0 |
| 0.962582411 | 2.917985114 | PC.aa.C38.0 (interacts with) PC.ae.C40.2 |
| 0.962479562 | 6.071555387 | PC.aa.C38.5 (interacts with) PC.ae.C38.0 |
| 0.962158381 | 9.900451548 | SM.C16.1 (interacts with) SM.C18.1 |
| 0.961472511 | 8.55959596 | SM.C16.0 (interacts with) SM.C18.1 |
| 0.960753386 | 6.285637787 | PC.ae.C34.0 (interacts with) PC.ae.C36.0 |
| 0.960720302 | 2.396825397 | PC.aa.C38.5 (interacts with) PC.aa.C42.5 |
| 0.959128382 | 4.096029088 | SM.OH.C22.2 (interacts with) SM.C18.0 |
| 0.957750096 | 3.933501862 | PC.ae.C36.4 (interacts with) PC.ae.C38.4 |
| 0.957552353 | 8.873022939 | PC.ae.C42.5 (interacts with) SM.C16.0 |
| 0.956393853 | 2.959958795 | PC.ae.C36.5 (interacts with) PC.ae.C40.4 |
| 0.955935999 | 6.353896524 | PC.ae.C38.4 (interacts with) PC.ae.C40.4 |
| 0.95590239 | 9.427775456 | PC.aa.C34.1 (interacts with) PC.aa.C40.4 |
| 0.955766139 | 6.543272336 | PC.ae.C34.0 (interacts with) PC.ae.C38.6 |
| 0.955615386 | 2.935632173 | PC.ae.C40.2 (interacts with) PC.ae.C40.5 |
| 0.95472267 | 2.965672515 | PC.ae.C38.6 (interacts with) PC.ae.C40.2 |
| 0.954108928 | 2.473103708 | PC.aa.C42.0 (interacts with) PC.ae.C40.6 |
| 0.953665843 | 3.020757602 | PC.ae.C32.1 (interacts with) PC.ae.C42.4 |
| 0.95337067 | 3.680769618 | SM.C16.0 (interacts with) SM.C18.0 |
| 0.953007591 | 2.535714286 | Trp (interacts with) Tyr |
| 0.952927851 | 3.475513985 | PC.aa.C40.6 (interacts with) PC.aa.C42.6 |
| 0.952816912 | 2.823145871 | PC.ae.C40.3 (interacts with) PC.ae.C42.4 |
| 0.952508181 | 2.535714286 | Phe (interacts with) Trp |
| 0.952271027 | 2.950078445 | SM.C18.0 (interacts with) SM.C24.1 |
| 0.95181701 | 9.092142706 | SM.OH.C16.1 (interacts with) SM.OH.C22.2 |
| 0.950989252 | 3.214012799 | PC.aa.C38.0 (interacts with) PC.aa.C42.0 |
| 0.950985472 | 10.63505063 | PC.ae.C42.4 (interacts with) SM.C16.0 |
| 0.950530295 | 5.382486078 | PC.ae.C32.1 (interacts with) PC.ae.C32.2 |
| 0.949602951 | 4.197694704 | PC.ae.C42.5 (interacts with) PC.ae.C44.6 |
| 0.949333893 | 4.687120021 | PC.ae.C36.4 (interacts with) PC.ae.C38.5 |
| 0.949332221 | 8.740593226 | SM.OH.C22.2 (interacts with) SM.C18.1 |
| 0.94931297 | 10.19282127 | PC.ae.C34.1 (interacts with) SM.C16.1 |
| 0.949070331 | 8.663172999 | SM.OH.C14.1 (interacts with) SM.C16.0 |
| 0.948526323 | 5.620450766 | PC.ae.C34.1 (interacts with) PC.ae.C36.2 |
| 0.948479302 | 5.21672388 | PC.ae.C34.2 (interacts with) PC.ae.C36.2 |
| 0.948313663 | 4.548840522 | PC.aa.C42.1 (interacts with) PC.ae.C44.6 |
| 0.947942375 | 10.40938193 | PC.ae.C34.0 (interacts with) PC.ae.C42.2 |
| 0.947780997 | 2 | C18.1 (interacts with) C18.2 |
| 0.947541512 | 2 | Phe (interacts with) Tyr |
| 0.947465786 | 4.301694683 | PC.aa.C40.5 (interacts with) PC.aa.C42.5 |
| 0.947376647 | 2.748148714 | PC.ae.C42.5 (interacts with) SM.C18.1 |
| 0.94721316 | 5.117566271 | PC.aa.C40.6 (interacts with) PC.ae.C38.0 |
| 0.947115895 | 3.410654637 | PC.ae.C32.1 (interacts with) SM.OH.C14.1 |
| 0.946873469 | 10.11488373 | PC.ae.C32.1 (interacts with) SM.C16.0 |
| 0.946760202 | 4.821860332 | PC.aa.C28.1 (interacts with) PC.ae.C36.1 |
| 0.946737156 | 4.290117358 | PC.ae.C36.3 (interacts with) PC.ae.C36.4 |
| 0.946298638 | 11.50212349 | PC.ae.C34.1 (interacts with) PC.ae.C38.3 |
| 0.945942288 | 7.974940031 | PC.aa.C38.5 (interacts with) PC.aa.C38.6 |
| 0.945939141 | 9.000595968 | PC.ae.C34.1 (interacts with) PC.ae.C42.4 |
| 0.945375673 | 5.945031719 | PC.ae.C34.1 (interacts with) PC.ae.C42.5 |
| 0.945291872 | 15.4609632 | PC.ae.C44.6 (interacts with) SM.C16.0 |
| 0.94511989 | 2.252044818 | PC.ae.C40.3 (interacts with) PC.ae.C42.5 |
| 0.945007309 | 3.789149269 | PC.ae.C34.1 (interacts with) PC.ae.C40.4 |
| 0.944685088 | 4.286868769 | PC.ae.C36.5 (interacts with) PC.ae.C38.4 |
| 0.94460162 | 10.21252369 | PC.ae.C42.5 (interacts with) SM.C16.1 |
| 0.944490325 | 36.79602056 | PC.ae.C34.0 (interacts with) PC.ae.C36.1 |
| 0.944229082 | 2.357575758 | PC.ae.C38.5 (interacts with) PC.ae.C42.5 |
| 0.944069496 | 227.1283919 | His (interacts with) Phe |
| 0.944056928 | 3.324169942 | PC.aa.C36.0 (interacts with) PC.ae.C34.0 |
| 0.943440536 | 7.264540041 | PC.ae.C36.0 (interacts with) PC.ae.C42.2 |
| 0.942857243 | 5.585447368 | PC.ae.C34.1 (interacts with) PC.ae.C40.3 |
| 0.942844063 | 8.65227326 | SM.OH.C14.1 (interacts with) SM.OH.C22.2 |
| 0.942236456 | 6.142357754 | PC.ae.C34.1 (interacts with) PC.ae.C38.5 |
| 0.942137455 | 18.02106699 | PC.aa.C32.0 (interacts with) PC.ae.C42.3 |
| 0.941988807 | 5.795873016 | PC.aa.C40.6 (interacts with) PC.aa.C42.5 |
| 0.941697177 | 16.23454365 | SM.OH.C22.1 (interacts with) SM.OH.C24.1 |
| 0.941655081 | 6.445796541 | PC.aa.C36.0 (interacts with) PC.ae.C40.2 |
| 0.941431931 | 47.04748653 | PC.aa.C32.0 (interacts with) PC.ae.C40.1 |
| 0.940918977 | 7.080502593 | PC.aa.C38.4 (interacts with) PC.aa.C40.4 |
| 0.940288746 | 13.6538843 | PC.ae.C36.1 (interacts with) PC.ae.C38.6 |
| 0.939030825 | 12.01423928 | PC.ae.C34.1 (interacts with) SM.C16.0 |
| 0.939018462 | 7.679887737 | PC.aa.C36.0 (interacts with) PC.ae.C36.0 |
| 0.938939804 | 11.45438448 | PC.ae.C32.1 (interacts with) SM.C16.1 |
| 0.938853947 | 10 | C12.1 (interacts with) C14.1 |
| 0.938765597 | 10.7562575 | PC.aa.C32.0 (interacts with) PC.ae.C42.2 |
| 0.938680301 | 4.34799855 | SM.OH.C22.2 (interacts with) SM.C16.1 |
| 0.938643877 | 12.64651515 | PC.ae.C42.4 (interacts with) SM.C16.1 |
| 0.937727822 | 7.457858228 | PC.ae.C34.0 (interacts with) PC.ae.C40.5 |
| 0.937582211 | 6.316427655 | PC.ae.C34.0 (interacts with) PC.ae.C40.6 |
| 0.936988063 | 3.696575981 | PC.ae.C40.2 (interacts with) SM.OH.C16.1 |
| 0.936513874 | 5.266470123 | PC.ae.C34.1 (interacts with) SM.OH.C14.1 |
| 0.936242338 | 230.5106194 | SM.OH.C22.1 (interacts with) SM.C24.0 |
| 0.935842895 | 2.686580087 | PC.ae.C40.3 (interacts with) SM.C18.1 |
| 0.935650106 | 53.04935065 | Leu (interacts with) Lys |
| 0.934874592 | 67.35559335 | Ile (interacts with) Lys |
| 0.934549868 | 3.518819307 | PC.ae.C34.3 (interacts with) PC.ae.C36.4 |
| 0.934242072 | 4.740965058 | PC.ae.C36.1 (interacts with) PC.ae.C38.3 |
| 0.934235112 | 9.865525165 | PC.aa.C40.5 (interacts with) PC.aa.C40.6 |
| 0.934185151 | 4.820167537 | PC.ae.C32.1 (interacts with) PC.ae.C40.4 |
| 0.93404149 | 10.75712981 | PC.aa.C40.5 (interacts with) PC.ae.C38.0 |
| 0.933866363 | 3.126431246 | PC.ae.C42.4 (interacts with) PC.ae.C44.6 |
| 0.933779919 | 9.239790141 | PC.ae.C38.5 (interacts with) SM.C16.0 |
| 0.933496452 | 10.69878452 | PC.ae.C38.5 (interacts with) SM.C16.1 |
| 0.933285579 | 6.363745476 | PC.ae.C32.1 (interacts with) PC.ae.C36.2 |
| 0.932941885 | 5.213374048 | PC.ae.C44.5 (interacts with) PC.ae.C44.6 |
| 0.932928996 | 6.259627832 | SM.OH.C22.2 (interacts with) SM.C24.1 |
| 0.932651984 | 2.693430546 | PC.ae.C32.2 (interacts with) PC.ae.C34.1 |
| 0.932583812 | 9.77765498 | PC.ae.C40.4 (interacts with) SM.C16.1 |
| 0.932519496 | 9.166711176 | PC.ae.C40.3 (interacts with) SM.OH.C22.2 |
| 0.932331588 | 3.543461482 | PC.ae.C42.5 (interacts with) SM.OH.C14.1 |
| 0.932221794 | 2.664012799 | PC.aa.C42.0 (interacts with) PC.ae.C38.6 |
| 0.931575475 | 2.656232478 | PC.ae.C38.4 (interacts with) PC.ae.C42.4 |
| 0.931531791 | 2.922346132 | PC.aa.C42.0 (interacts with) PC.ae.C40.5 |
| 0.931453339 | 10.47090235 | PC.ae.C40.4 (interacts with) SM.C16.0 |
| 0.93134267 | 57.26867168 | Lys (interacts with) Val |
| 0.931108179 | 7.171429093 | PC.ae.C34.1 (interacts with) PC.ae.C38.4 |
| 0.931082134 | 6.968942428 | PC.aa.C42.5 (interacts with) PC.ae.C38.0 |
| 0.93104926 | 40.29336913 | PC.aa.C36.6 (interacts with) PC.ae.C38.0 |
| 0.930580169 | 16.94282491 | PC.ae.C44.6 (interacts with) SM.OH.C22.2 |
| 0.930489902 | 3.220886922 | SM.OH.C14.1 (interacts with) SM.C18.1 |
| 0.930249682 | 4.495421143 | PC.ae.C40.3 (interacts with) PC.ae.C44.6 |
| 0.929971626 | 3.866808088 | PC.ae.C40.4 (interacts with) SM.C18.1 |
| 0.929849658 | 27.86124255 | PC.ae.C38.6 (interacts with) PC.ae.C42.2 |
| 0.929649856 | 12.70386344 | PC.ae.C36.1 (interacts with) PC.ae.C40.5 |
| 0.929425939 | 2.649362537 | PC.ae.C38.5 (interacts with) PC.ae.C42.4 |
| 0.929225891 | 8.658409851 | PC.aa.C38.0 (interacts with) PC.ae.C34.0 |
| 0.929213024 | 12.09868243 | PC.aa.C38.0 (interacts with) PC.ae.C36.1 |
| 0.929055089 | 14.77585543 | PC.aa.C42.1 (interacts with) SM.OH.C22.2 |
| 0.928633613 | 8.937211871 | PC.aa.C38.6 (interacts with) PC.aa.C42.6 |
| 0.928187759 | 3.11298365 | SM.OH.C16.1 (interacts with) SM.C18.1 |
| 0.928065576 | 2.652525253 | PC.aa.C38.6 (interacts with) PC.ae.C38.0 |
| 0.927820351 | 4.775694527 | PC.ae.C40.3 (interacts with) PC.ae.C40.4 |
| 0.927741853 | 9.515909004 | SM.OH.C14.1 (interacts with) SM.C16.1 |
| 0.927594607 | 2.999288733 | PC.ae.C40.4 (interacts with) PC.ae.C40.5 |
| 0.927525139 | 29.1744402 | PC.ae.C36.0 (interacts with) PC.ae.C38.1 |
| 0.926861064 | 24.48225108 | C0 (interacts with) Leu |
| 0.92649351 | 11.13880811 | PC.aa.C36.5 (interacts with) PC.aa.C42.6 |
| 0.926150719 | 5.233646555 | PC.aa.C34.3 (interacts with) PC.aa.C34.4 |
| 0.925909002 | 5.119547069 | PC.aa.C42.6 (interacts with) PC.ae.C38.1 |
| 0.925641935 | 7.089848835 | SM.C16.1 (interacts with) SM.C18.0 |
| 0.925600198 | 10 | C12.1 (interacts with) C14.2 |
| 0.925315144 | 9.515690253 | PC.ae.C40.3 (interacts with) SM.C18.0 |
| 0.924656346 | 5.597566369 | PC.ae.C36.2 (interacts with) PC.ae.C36.3 |
| 0.924534375 | 2.423573925 | PC.ae.C38.5 (interacts with) PC.ae.C40.3 |
| 0.924314217 | 3.480461356 | SM.OH.C22.1 (interacts with) SM.OH.C22.2 |
| 0.924059756 | 4.220461773 | PC.aa.C38.0 (interacts with) PC.ae.C32.2 |
| 0.923646404 | 2.296172249 | PC.ae.C32.1 (interacts with) PC.ae.C38.5 |
| 0.923594186 | 11.03451509 | PC.aa.C36.0 (interacts with) PC.aa.C38.1 |
| 0.923556313 | 45.97070225 | PC.ae.C38.1 (interacts with) PC.ae.C42.2 |
| 0.923403333 | 18.67371548 | PC.ae.C36.0 (interacts with) PC.ae.C38.6 |
| 0.923277374 | 21.40260719 | PC.aa.C36.6 (interacts with) PC.aa.C38.5 |
| 0.922774591 | 9.97683996 | PC.ae.C42.5 (interacts with) SM.OH.C22.2 |
| 0.9227488 | 4.458647691 | PC.aa.C38.0 (interacts with) SM.OH.C16.1 |
| 0.922570906 | 11.13880811 | PC.aa.C36.6 (interacts with) PC.aa.C42.6 |
| 0.922438788 | 24.83191463 | PC.ae.C42.2 (interacts with) PC.ae.C42.3 |
| 0.922411045 | 416 | Putrescine (interacts with) Taurine |
| 0.922322812 | 8.083265855 | PC.ae.C36.2 (interacts with) SM.OH.C14.1 |
| 0.922189754 | 4.699306727 | PC.ae.C32.2 (interacts with) SM.OH.C14.1 |
| 0.922119489 | 2.801058063 | PC.ae.C38.4 (interacts with) PC.ae.C42.5 |
| 0.921882761 | 10 | C2 (interacts with) C12.1 |
| 0.921587762 | 9.745332004 | SM.OH.C16.1 (interacts with) SM.C16.0 |
| 0.921577271 | 8.076801524 | PC.ae.C40.4 (interacts with) PC.ae.C42.4 |
| 0.921516049 | 10.0771595 | PC.ae.C34.0 (interacts with) PC.ae.C40.2 |
| 0.921298859 | 21.40260719 | PC.aa.C36.5 (interacts with) PC.aa.C38.5 |
| 0.921226564 | 23.10986902 | PC.aa.C40.4 (interacts with) PC.ae.C40.1 |
| 0.920852781 | 3.547821196 | PC.ae.C42.4 (interacts with) SM.C18.1 |
| 0.919956118 | 3.691088822 | PC.aa.C42.0 (interacts with) PC.ae.C40.2 |
| 0.9197774 | 39.85555556 | Asn (interacts with) Met |
| 0.91965642 | 5.926353386 | PC.ae.C32.2 (interacts with) PC.ae.C42.5 |
| 0.919419244 | 9.708587888 | PC.ae.C38.4 (interacts with) SM.C16.0 |
| 0.919318447 | 2 | PC.aa.C36.5 (interacts with) PC.aa.C36.6 |
| 0.919111795 | 3.47965598 | PC.ae.C40.3 (interacts with) SM.OH.C14.1 |
| 0.918536277 | 2.67159369 | PC.ae.C32.1 (interacts with) SM.C18.1 |
| 0.918158145 | 15.90530741 | PC.ae.C36.1 (interacts with) PC.ae.C42.3 |
| 0.918095169 | 8.836146138 | PC.aa.C36.1 (interacts with) PC.aa.C40.4 |
| 0.917855706 | 111.9375775 | PC.ae.C36.1 (interacts with) PC.ae.C42.2 |
| 0.917823717 | 8.624392549 | PC.ae.C34.1 (interacts with) PC.ae.C42.3 |
| 0.917709445 | 4.385477554 | PC.ae.C34.2 (interacts with) PC.ae.C36.4 |
| 0.917599788 | 15.61061793 | PC.ae.C36.1 (interacts with) PC.ae.C40.6 |
| 0.917389534 | 2.539126158 | PC.ae.C32.1 (interacts with) PC.ae.C40.3 |
| 0.917210051 | 9.793025588 | PC.aa.C38.0 (interacts with) PC.ae.C42.3 |
| 0.91713581 | 251.616354 | lysoPC.a.C16.0 (interacts with) lysoPC.a.C18.0 |
| 0.91702681 | 3.778473143 | PC.ae.C34.1 (interacts with) PC.ae.C36.5 |
| 0.916985191 | 17.22690417 | PC.aa.C40.5 (interacts with) PC.aa.C42.6 |
| 0.916876309 | 12 | C14 (interacts with) C18.1 |
| 0.916864108 | 20.07311799 | Met (interacts with) Tyr |
| 0.915697503 | 3.936328775 | PC.ae.C40.2 (interacts with) SM.OH.C14.1 |
| 0.915607635 | 28.28721805 | C0 (interacts with) Val |
| 0.91528984 | 30.85830392 | lysoPC.a.C18.0 (interacts with) lysoPC.a.C18.1 |
| 0.914827176 | 4.507816876 | PC.aa.C38.0 (interacts with) SM.OH.C14.1 |
| 0.914430935 | 8.60009903 | PC.aa.C36.2 (interacts with) PC.ae.C38.2 |
| 0.914276771 | 2.756021883 | PC.aa.C38.0 (interacts with) PC.ae.C40.4 |
| 0.914255055 | 3.498969956 | PC.ae.C36.4 (interacts with) PC.ae.C42.4 |
| 0.91389471 | 9.470270897 | PC.aa.C38.6 (interacts with) PC.aa.C42.5 |
| 0.913740019 | 10.07721619 | PC.ae.C40.4 (interacts with) PC.ae.C42.3 |
| 0.913247602 | 4.42001255 | PC.ae.C32.2 (interacts with) PC.ae.C38.6 |
| 0.913063082 | 9.773054078 | PC.ae.C40.2 (interacts with) SM.OH.C22.2 |
| 0.912967239 | 15.90199154 | PC.ae.C38.3 (interacts with) PC.ae.C42.3 |
| 0.912899127 | 3.714620375 | PC.ae.C30.0 (interacts with) PC.ae.C32.2 |
| 0.912685178 | 42.14278888 | PC.aa.C34.1 (interacts with) PC.ae.C40.1 |
| 0.912122744 | 3.724952573 | PC.ae.C42.5 (interacts with) SM.OH.C16.1 |
| 0.911887689 | 7.519747297 | PC.ae.C34.3 (interacts with) PC.ae.C36.2 |
| 0.91183959 | 12.88635716 | PC.ae.C34.1 (interacts with) PC.ae.C36.1 |
| 0.911485001 | 6.165591846 | SM.C16.0 (interacts with) SM.C24.1 |
| 0.911446633 | 11.28443633 | PC.ae.C38.4 (interacts with) SM.C16.1 |
| 0.911443326 | 18.51756244 | Met (interacts with) Trp |
| 0.911423226 | 3.712681476 | PC.ae.C40.4 (interacts with) SM.OH.C14.1 |
| 0.91102651 | 4.699924242 | PC.ae.C44.6 (interacts with) SM.C18.1 |
| 0.910917893 | 4.500383998 | PC.aa.C42.1 (interacts with) PC.ae.C42.5 |
| 0.910848449 | 5.746949736 | PC.ae.C34.1 (interacts with) SM.C18.1 |
| 0.910733253 | 2.867371672 | PC.ae.C38.4 (interacts with) PC.ae.C40.3 |
| 0.910607942 | 11.99071887 | PC.aa.C38.1 (interacts with) PC.ae.C40.6 |
| 0.910432732 | 31.37277295 | PC.ae.C40.5 (interacts with) PC.ae.C42.2 |
| 0.910354879 | 9.452689398 | PC.ae.C40.5 (interacts with) PC.ae.C42.3 |
| 0.910306913 | 30.19442213 | PC.aa.C36.1 (interacts with) PC.ae.C40.1 |
| 0.910253254 | 8.087026684 | SM.OH.C14.1 (interacts with) SM.OH.C22.1 |
| 0.909962545 | 13.00292781 | C3 (interacts with) Pro |
| 0.90983713 | 2.766431068 | PC.ae.C38.5 (interacts with) SM.C18.1 |
| 0.909764073 | 135.6000481 | lysoPC.a.C16.0 (interacts with) lysoPC.a.C16.1 |
| 0.909603794 | 13.15598869 | PC.ae.C32.2 (interacts with) SM.C16.1 |
| 0.909486909 | 9.173642104 | PC.ae.C40.2 (interacts with) SM.OH.C22.1 |
| 0.909300199 | 65.01485023 | PC.aa.C32.1 (interacts with) PC.aa.C36.1 |
| 0.909285052 | 20.46532788 | PC.ae.C44.6 (interacts with) SM.C16.1 |
| 0.908926128 | 12.92266795 | PC.ae.C36.1 (interacts with) PC.ae.C40.2 |
| 0.908901239 | 9.853236789 | PC.ae.C40.4 (interacts with) SM.OH.C22.2 |
| 0.908724502 | 4.368337648 | PC.ae.C34.3 (interacts with) PC.ae.C42.4 |
| 0.908701698 | 85.90442152 | Met (interacts with) Thr |
| 0.908359314 | 5.173824158 | PC.ae.C34.2 (interacts with) PC.ae.C42.4 |
| 0.908222419 | 7.803174603 | Lys (interacts with) Met |
| 0.908075507 | 9.09868595 | PC.ae.C32.2 (interacts with) PC.ae.C42.3 |
| 0.908057503 | 7.18821084 | PC.ae.C36.2 (interacts with) PC.ae.C42.4 |
| 0.908021964 | 5.176307076 | PC.ae.C40.6 (interacts with) SM.OH.C16.1 |
| 0.907926839 | 13.93324978 | PC.ae.C44.6 (interacts with) SM.C18.0 |
| 0.907352805 | 9.34605363 | PC.ae.C38.6 (interacts with) PC.ae.C42.3 |
| 0.906776331 | 27.55227629 | PC.ae.C40.6 (interacts with) PC.ae.C42.2 |
| 0.906636278 | 81.71571083 | PC.aa.C32.0 (interacts with) PC.ae.C36.1 |
| 0.906617401 | 10.60437797 | SM.OH.C16.1 (interacts with) SM.C18.0 |
| 0.906505191 | 3.786622657 | PC.ae.C36.5 (interacts with) PC.ae.C42.5 |
| 0.90649561 | 58.83926693 | PC.aa.C32.2 (interacts with) PC.aa.C34.4 |
| 0.905644503 | 13.82878788 | PC.aa.C34.1 (interacts with) PC.aa.C38.4 |
| 0.90563278 | 5.556345597 | PC.ae.C36.3 (interacts with) PC.ae.C42.4 |
| 0.905466266 | 3.473701418 | PC.ae.C30.0 (interacts with) SM.OH.C14.1 |
| 0.905401585 | 8.418059071 | PC.aa.C36.4 (interacts with) PC.aa.C40.4 |
| 0.905354192 | 21.91944966 | PC.ae.C36.0 (interacts with) PC.ae.C40.5 |
| 0.90520503 | 6.503504648 | PC.ae.C32.2 (interacts with) PC.ae.C36.2 |
| 0.905096994 | 36.72603928 | PC.aa.C38.0 (interacts with) PC.ae.C42.2 |
| 0.905048786 | 2.72450304 | PC.ae.C32.1 (interacts with) PC.ae.C38.4 |
| 0.90501124 | 20.07554478 | PC.ae.C38.3 (interacts with) PC.ae.C40.3 |
| 0.90448862 | 5.097433488 | PC.aa.C42.0 (interacts with) PC.ae.C32.2 |
| 0.904363531 | 67.6082551 | PC.aa.C28.1 (interacts with) PC.aa.C32.0 |
| 0.903763665 | 24.05630897 | PC.ae.C42.3 (interacts with) SM.C16.1 |
| 0.903561114 | 15.00011338 | C0 (interacts with) Lys |
| 0.902771906 | 45.57832036 | PC.aa.C36.2 (interacts with) PC.aa.C36.3 |
| 0.902698611 | 142.5482478 | PC.ae.C40.1 (interacts with) PC.ae.C42.3 |
| 0.902629836 | 3.696369003 | PC.ae.C32.1 (interacts with) PC.ae.C36.5 |
| 0.902370578 | 10.15774117 | PC.ae.C42.5 (interacts with) SM.C18.0 |
| 0.901921957 | 15.67765971 | PC.aa.C42.1 (interacts with) SM.C16.0 |
| 0.901632357 | 45.5354924 | PC.aa.C36.3 (interacts with) PC.aa.C38.3 |
| 0.901221481 | 42.29578703 | PC.aa.C32.1 (interacts with) PC.aa.C34.1 |
| 0.90119096 | 4.972897805 | PC.aa.C42.1 (interacts with) PC.ae.C40.3 |
| 0.90116719 | 3.276886049 | PC.ae.C38.6 (interacts with) PC.ae.C40.4 |
| 0.901128666 | 41.74347716 | PC.ae.C38.0 (interacts with) PC.ae.C42.2 |
| 0.90105982 | 3.573963909 | PC.ae.C32.1 (interacts with) SM.OH.C16.1 |
| 0.901022851 | 8.510375055 | SM.OH.C16.1 (interacts with) SM.OH.C22.1 |
| 0.900744057 | 227.6641062 | His (interacts with) Trp |
| 0.900675071 | 16.12658495 | PC.ae.C36.2 (interacts with) PC.ae.C38.3 |
| 0.900545589 | 227.1283919 | His (interacts with) Tyr |
| 0.900329005 | 931.8327264 | Asn (interacts with) His |
| 0.899946118 | 4.690925529 | PC.ae.C40.5 (interacts with) SM.OH.C16.1 |
| 0.899842093 | 4.690060653 | SM.OH.C22.1 (interacts with) SM.C18.0 |
| 0.899718757 | 14.0436252 | PC.aa.C36.1 (interacts with) PC.aa.C40.5 |
| 0.899544181 | 4.335059637 | PC.ae.C32.2 (interacts with) PC.ae.C40.5 |
| 0.899277707 | 9.373864674 | PC.ae.C36.5 (interacts with) SM.C16.0 |
| 0.8990166 | 14.22361059 | PC.ae.C40.4 (interacts with) SM.C18.0 |
| 0.898887815 | 6.627258151 | PC.aa.C28.1 (interacts with) PC.ae.C38.3 |
| 0.898834137 | 16.45416525 | PC.aa.C28.1 (interacts with) PC.ae.C42.3 |
| 0.898648305 | 3.463791379 | PC.ae.C32.2 (interacts with) PC.ae.C40.4 |
| 0.898570059 | 3.781517514 | PC.ae.C40.4 (interacts with) SM.OH.C16.1 |
| 0.898398774 | 25.50257518 | PC.aa.C38.6 (interacts with) PC.ae.C36.0 |
| 0.898396137 | 2 | C2 (interacts with) C14.1 |
| 0.898117364 | 5.604763083 | PC.aa.C42.0 (interacts with) SM.OH.C14.1 |
| 0.897919324 | 36.44587724 | PC.aa.C38.6 (interacts with) PC.ae.C42.2 |
| 0.897522654 | 30.18684747 | PC.aa.C36.0 (interacts with) PC.ae.C36.1 |
| 0.897454199 | 14.40103328 | PC.ae.C36.2 (interacts with) SM.C16.1 |
| 0.897262562 | 3.835003871 | SM.OH.C22.1 (interacts with) SM.C16.0 |
| 0.897148981 | 26.32094204 | PC.aa.C42.0 (interacts with) PC.ae.C42.2 |
| 0.897092488 | 6.429909863 | PC.aa.C42.0 (interacts with) PC.ae.C34.0 |
| 0.897020392 | 22.87921873 | PC.ae.C38.3 (interacts with) PC.ae.C38.4 |
| 0.896970202 | 14.02289309 | PC.ae.C38.3 (interacts with) PC.ae.C40.4 |
| 0.896968081 | 12.81702039 | PC.ae.C34.1 (interacts with) PC.ae.C34.2 |
| 0.896808746 | 17.45028826 | PC.ae.C36.0 (interacts with) PC.ae.C40.6 |
| 0.89678026 | 202.9506404 | lysoPC.a.C16.1 (interacts with) lysoPC.a.C18.0 |
| 0.896725924 | 13.91999886 | PC.aa.C38.1 (interacts with) PC.ae.C40.2 |
| 0.896656786 | 7.048021222 | PC.ae.C40.5 (interacts with) PC.ae.C42.5 |
| 0.89661938 | 6.725916544 | PC.aa.C38.0 (interacts with) PC.ae.C42.5 |
| 0.896580268 | 14.06680684 | PC.aa.C34.1 (interacts with) PC.aa.C40.5 |
| 0.896426707 | 12.36783894 | PC.ae.C36.1 (interacts with) PC.ae.C40.4 |
| 0.896278181 | 3.413636364 | PC.ae.C38.0 (interacts with) PC.ae.C38.1 |
| 0.895941519 | 56.55302869 | PC.aa.C32.1 (interacts with) PC.aa.C40.5 |
| 0.895814363 | 5.029858721 | PC.ae.C42.4 (interacts with) SM.OH.C14.1 |
| 0.895755231 | 8.20253031 | PC.ae.C38.3 (interacts with) SM.C16.1 |
| 0.895698677 | 4.146716312 | PC.aa.C38.0 (interacts with) PC.ae.C36.5 |
| 0.895668168 | 15.02835003 | PC.aa.C36.0 (interacts with) PC.ae.C42.2 |
| 0.895061343 | 4.634670501 | PC.ae.C30.0 (interacts with) PC.ae.C34.1 |
| 0.895019002 | 3.220077299 | PC.ae.C38.4 (interacts with) SM.C18.1 |
| 0.894990837 | 151.1607331 | PC.aa.C32.2 (interacts with) PC.aa.C34.3 |
| 0.894990315 | 4.762219173 | PC.ae.C36.5 (interacts with) PC.ae.C38.6 |
| 0.894845808 | 3.235925967 | PC.aa.C38.0 (interacts with) PC.ae.C30.0 |
| 0.894820134 | 3.079390928 | PC.ae.C30.0 (interacts with) PC.ae.C38.6 |
| 0.894285028 | 6.075677162 | PC.ae.C32.1 (interacts with) PC.ae.C34.2 |
| 0.894228855 | 17.99784819 | PC.aa.C36.5 (interacts with) PC.aa.C42.5 |
| 0.893997252 | 15.60557884 | PC.ae.C36.2 (interacts with) SM.C16.0 |
| 0.893048303 | 42.87697884 | PC.aa.C32.1 (interacts with) PC.aa.C34.4 |
| 0.892964349 | 141.7432844 | PC.aa.C32.0 (interacts with) PC.aa.C34.1 |
| 0.892738666 | 12.75856794 | PC.aa.C36.5 (interacts with) PC.aa.C40.6 |
| 0.892687627 | 28.7835979 | lysoPC.a.C16.0 (interacts with) lysoPC.a.C18.1 |
| 0.892140407 | 9.330356496 | PC.ae.C36.1 (interacts with) SM.OH.C22.1 |
| 0.891693476 | 8.924501244 | PC.ae.C36.5 (interacts with) SM.C16.1 |
| 0.891650085 | 13.74149794 | PC.ae.C36.1 (interacts with) PC.ae.C36.5 |
| 0.891647696 | 4.484776044 | PC.ae.C32.1 (interacts with) PC.ae.C44.6 |
| 0.891556069 | 32.83214286 | C0 (interacts with) Ile |
| 0.891364184 | 3.742850231 | PC.ae.C40.2 (interacts with) PC.ae.C40.4 |
| 0.890994544 | 4.940550818 | PC.ae.C38.6 (interacts with) SM.OH.C16.1 |
| 0.890854706 | 5.349407886 | PC.ae.C40.6 (interacts with) SM.OH.C14.1 |
| 0.89085333 | 4.433106705 | PC.ae.C36.5 (interacts with) PC.ae.C40.5 |
| 0.890428411 | 3.282128364 | PC.ae.C30.0 (interacts with) PC.ae.C40.6 |
| 0.890256064 | 6.617180148 | PC.ae.C36.2 (interacts with) PC.ae.C40.3 |
| 0.890222033 | 10.29267045 | PC.aa.C38.0 (interacts with) SM.OH.C22.2 |
| 0.890071531 | 4.302086518 | PC.aa.C36.0 (interacts with) PC.aa.C42.0 |
| 0.889915113 | 11.62458655 | SM.C18.1 (interacts with) SM.C24.1 |
| 0.889790018 | 19.78186813 | PC.aa.C34.1 (interacts with) PC.aa.C36.4 |
| 0.889744168 | 27.00792186 | PC.ae.C42.3 (interacts with) PC.ae.C42.5 |
| 0.889446463 | 8.066135548 | PC.aa.C42.5 (interacts with) PC.ae.C38.1 |
| 0.888897951 | 10.41959539 | PC.ae.C38.5 (interacts with) SM.OH.C22.2 |
| 0.888621824 | 3.319819156 | PC.ae.C30.0 (interacts with) PC.ae.C40.2 |
| 0.888614737 | 10.50926854 | SM.OH.C14.1 (interacts with) SM.C18.0 |
| 0.888573431 | 22.17783053 | PC.ae.C38.3 (interacts with) PC.ae.C38.5 |
| 0.888320771 | 37.03321223 | Orn (interacts with) Thr |
| 0.888045107 | 18.96821604 | PC.ae.C36.4 (interacts with) SM.C16.0 |
| 0.887930554 | 10.24862692 | PC.ae.C40.6 (interacts with) PC.ae.C42.3 |
| 0.887700559 | 72.46841403 | PC.aa.C32.0 (interacts with) PC.aa.C40.4 |
| 0.887679656 | 5.114877636 | PC.aa.C42.0 (interacts with) SM.OH.C16.1 |
| 0.887602196 | 5.60488556 | PC.ae.C32.2 (interacts with) PC.ae.C40.3 |
| 0.887406253 | 10.07342745 | PC.ae.C38.5 (interacts with) SM.C18.0 |
| 0.887359666 | 26.94585541 | PC.aa.C34.3 (interacts with) PC.aa.C36.1 |
| 0.886898659 | 6.42473992 | PC.aa.C38.0 (interacts with) PC.ae.C32.1 |
| 0.886849473 | 3.5770281 | PC.ae.C38.5 (interacts with) SM.OH.C14.1 |
| 0.886847563 | 15.90899916 | PC.aa.C38.6 (interacts with) PC.aa.C40.5 |
| 0.886231297 | 6.887187276 | SM.OH.C22.1 (interacts with) SM.C24.1 |
| 0.886176405 | 4.660411907 | PC.ae.C30.0 (interacts with) PC.ae.C40.3 |
| 0.886003695 | 10.49958936 | PC.ae.C36.4 (interacts with) PC.ae.C36.5 |
| 0.88599174 | 15.17031458 | PC.aa.C28.1 (interacts with) PC.ae.C38.6 |
| 0.885894105 | 8.693519223 | PC.aa.C32.3 (interacts with) PC.aa.C34.2 |
| 0.885496256 | 5.511551848 | PC.ae.C32.2 (interacts with) SM.C18.1 |
| 0.885441491 | 7.201703147 | PC.aa.C42.0 (interacts with) PC.ae.C32.1 |
| 0.885429513 | 3.232909688 | PC.ae.C30.0 (interacts with) PC.ae.C40.5 |
| 0.885269484 | 4.768066743 | PC.ae.C40.5 (interacts with) SM.OH.C14.1 |
| 0.885216369 | 4.95232972 | PC.aa.C38.0 (interacts with) PC.ae.C34.1 |
| 0.885122847 | 21.22657632 | PC.aa.C42.1 (interacts with) SM.C16.1 |
| 0.884874714 | 5.501745144 | PC.ae.C32.2 (interacts with) SM.OH.C16.1 |
| 0.884860499 | 5.051025365 | PC.ae.C38.6 (interacts with) SM.OH.C14.1 |
| 0.884566533 | 59.68697148 | PC.ae.C40.1 (interacts with) PC.ae.C42.2 |
| 0.884426193 | 7.178773569 | PC.aa.C38.5 (interacts with) PC.ae.C38.1 |
| 0.884364019 | 10.44966744 | PC.aa.C42.0 (interacts with) PC.ae.C42.3 |
| 0.883988941 | 26.52399352 | PC.aa.C38.0 (interacts with) PC.ae.C36.0 |
| 0.883540007 | 5.369354584 | PC.ae.C36.3 (interacts with) PC.ae.C38.4 |
| 0.883368867 | 6.11026946 | PC.ae.C32.2 (interacts with) PC.ae.C38.5 |
| 0.883328487 | 13.12812874 | PC.ae.C38.3 (interacts with) SM.OH.C22.1 |
| 0.883018507 | 5.199138875 | PC.aa.C38.0 (interacts with) SM.C18.1 |
| 0.882986111 | 8.134971695 | Lys (interacts with) Tyr |
| 0.882982829 | 11.21870075 | PC.ae.C32.1 (interacts with) SM.OH.C22.2 |
| 0.8828552 | 4.942012934 | SM.OH.C22.1 (interacts with) SM.C16.1 |
| 0.882804413 | 5.168881187 | PC.ae.C36.4 (interacts with) PC.ae.C40.3 |
| 0.882459217 | 75.82248805 | PC.ae.C34.0 (interacts with) PC.ae.C38.1 |
| 0.882402418 | 21.77891037 | PC.aa.C36.6 (interacts with) PC.aa.C40.5 |
| 0.882237663 | 24.33103415 | PC.ae.C32.1 (interacts with) PC.ae.C42.3 |
| 0.882148542 | 18.71667787 | PC.aa.C28.1 (interacts with) PC.ae.C30.0 |
| 0.882023384 | 18.29051476 | PC.aa.C36.2 (interacts with) PC.ae.C40.1 |
| 0.881901295 | 12.75856794 | PC.aa.C36.6 (interacts with) PC.aa.C40.6 |
| 0.881780708 | 4.29260148 | PC.ae.C30.0 (interacts with) PC.ae.C32.1 |
| 0.881732774 | 99.58075832 | PC.aa.C28.1 (interacts with) PC.ae.C42.2 |
| 0.881724151 | 10.92784292 | SM.OH.C16.1 (interacts with) SM.C16.1 |
| 0.881644243 | 14.94113067 | PC.ae.C36.1 (interacts with) SM.OH.C14.1 |
| 0.881603915 | 31.3 | Asn (interacts with) Lys |
| 0.881569186 | 10.94128555 | PC.ae.C42.4 (interacts with) SM.C18.0 |
| 0.881407499 | 14.22688064 | PC.ae.C32.2 (interacts with) SM.C16.0 |
| 0.881385503 | 7.178464518 | PC.ae.C44.6 (interacts with) SM.OH.C14.1 |
| 0.881320947 | 18.4249553 | PC.aa.C32.3 (interacts with) PC.aa.C36.2 |
| 0.881231744 | 43.24179238 | PC.aa.C32.1 (interacts with) PC.aa.C34.3 |
| 0.881203663 | 8.134971695 | Lys (interacts with) Phe |
| 0.881112593 | 12 | C14 (interacts with) C18.2 |
| 0.880906955 | 6.28329409 | PC.ae.C34.1 (interacts with) SM.OH.C16.1 |
| 0.880343537 | 9.046190166 | SM.OH.C22.1 (interacts with) SM.C18.1 |
| 0.880320286 | 8.670685981 | Lys (interacts with) Trp |
| 0.880269465 | 24.36830503 | Asn (interacts with) Tyr |
| 0.880123476 | 4.06811665 | PC.aa.C38.6 (interacts with) PC.ae.C38.1 |
| 0.879881654 | 3.317396782 | PC.ae.C36.5 (interacts with) SM.OH.C14.1 |
| 0.8798597 | 6.005521406 | PC.ae.C32.1 (interacts with) PC.ae.C34.3 |
| 0.879848313 | 4.913154026 | PC.ae.C32.2 (interacts with) PC.ae.C40.6 |
| 0.879811983 | 3.962828299 | PC.ae.C40.3 (interacts with) SM.OH.C16.1 |
| 0.879782499 | 55.08183444 | PC.aa.C34.2 (interacts with) PC.ae.C38.3 |
| 0.879754524 | 13.69292749 | PC.ae.C34.1 (interacts with) PC.ae.C36.3 |
| 0.879639619 | 3.56898827 | PC.ae.C32.2 (interacts with) PC.ae.C36.5 |
| 0.879565346 | 22.55613722 | PC.ae.C36.4 (interacts with) SM.C16.1 |
| 0.879344428 | 36.87630843 | PC.ae.C38.2 (interacts with) PC.ae.C40.1 |
| 0.879104792 | 6.146428294 | PC.ae.C36.2 (interacts with) PC.ae.C38.4 |
| 0.878944376 | 5.567822053 | PC.aa.C32.3 (interacts with) PC.ae.C38.2 |
| 0.878797297 | 24.72788465 | PC.ae.C40.3 (interacts with) PC.ae.C42.3 |
| 0.878717553 | 74.37009193 | SM.OH.C24.1 (interacts with) SM.C24.0 |
| 0.878695717 | 14.56360374 | PC.aa.C38.1 (interacts with) PC.ae.C40.5 |
| 0.878440895 | 82.53954374 | PC.aa.C32.0 (interacts with) PC.ae.C38.3 |
| 0.878379733 | 9.909783236 | PC.ae.C40.4 (interacts with) SM.OH.C22.1 |
| 0.878069996 | 9.339652095 | PC.ae.C42.4 (interacts with) PC.ae.C44.5 |
| 0.878062012 | 16.45237237 | PC.ae.C34.1 (interacts with) PC.ae.C36.4 |
| 0.87791302 | 13.08703402 | PC.ae.C34.1 (interacts with) SM.OH.C22.2 |
| 0.877261308 | 52.70733377 | PC.ae.C38.0 (interacts with) PC.ae.C40.1 |
| 0.877206154 | 13.36999601 | PC.ae.C40.4 (interacts with) PC.ae.C44.6 |
| 0.876535873 | 7.924809595 | PC.aa.C42.0 (interacts with) PC.ae.C42.5 |
| 0.876522827 | 98.41616243 | PC.ae.C36.0 (interacts with) PC.ae.C36.1 |
| 0.876165073 | 40.20691141 | PC.aa.C30.0 (interacts with) PC.aa.C32.0 |
| 0.875789397 | 12.31001864 | PC.aa.C32.3 (interacts with) PC.ae.C36.2 |
| 0.875567342 | 3.553046348 | PC.ae.C40.4 (interacts with) PC.ae.C40.6 |
| 0.87533686 | 4.271978165 | PC.ae.C38.4 (interacts with) SM.OH.C14.1 |
| 0.875163643 | 10.08424996 | PC.ae.C38.4 (interacts with) SM.C18.0 |
| 0.875070584 | 108.3001441 | PC.aa.C38.3 (interacts with) PC.aa.C40.4 |
| 0.875057671 | 71.75835555 | lysoPC.a.C16.1 (interacts with) lysoPC.a.C18.1 |
| 0.874848026 | 16.76577588 | PC.aa.C28.1 (interacts with) PC.ae.C40.6 |
| 0.874357115 | 6.399877089 | PC.ae.C36.2 (interacts with) PC.ae.C38.5 |
| 0.874134649 | 661.7796306 | His (interacts with) Met |
| 0.874105539 | 6.445342468 | PC.aa.C34.2 (interacts with) PC.ae.C38.2 |
| 0.874064917 | 6.294680288 | PC.ae.C36.3 (interacts with) PC.ae.C38.5 |
| 0.873961691 | 17.64528714 | PC.aa.C28.1 (interacts with) PC.ae.C40.2 |
| 0.873745152 | 14.02757371 | PC.ae.C40.6 (interacts with) SM.OH.C22.2 |
| 0.873604402 | 4.399289461 | PC.aa.C42.1 (interacts with) SM.C18.1 |
| 0.87352859 | 9.113503204 | PC.ae.C32.2 (interacts with) PC.ae.C42.4 |
| 0.873299683 | 3.403097834 | PC.aa.C42.0 (interacts with) PC.ae.C30.0 |
| 0.873128131 | 17.19416448 | SM.C16.1 (interacts with) SM.C24.1 |
| 0.873079587 | 21.56802066 | PC.ae.C40.4 (interacts with) SM.C24.1 |
| 0.872852554 | 4.793390563 | PC.ae.C34.3 (interacts with) PC.ae.C38.4 |
| 0.872600376 | 12.61899774 | PC.ae.C42.4 (interacts with) SM.OH.C22.2 |
| 0.87255589 | 7.083271479 | PC.ae.C36.2 (interacts with) PC.ae.C42.5 |
| 0.872487451 | 13.38353227 | PC.aa.C38.1 (interacts with) PC.ae.C38.6 |
| 0.872380303 | 14.56298425 | PC.aa.C28.1 (interacts with) PC.aa.C38.0 |
| 0.872286861 | 12.99469456 | PC.ae.C34.1 (interacts with) SM.OH.C22.1 |
| 0.872077659 | 3.358344679 | PC.ae.C30.0 (interacts with) SM.OH.C16.1 |
| 0.871724251 | 15.00574449 | PC.ae.C32.2 (interacts with) PC.ae.C36.1 |
| 0.871694376 | 14.6556139 | PC.aa.C28.1 (interacts with) PC.ae.C40.5 |
| 0.871642792 | 9.982291999 | PC.ae.C40.3 (interacts with) SM.OH.C22.1 |
| 0.871326135 | 6.756790535 | PC.ae.C44.6 (interacts with) SM.OH.C16.1 |
| 0.87109649 | 4.236585879 | PC.aa.C40.6 (interacts with) PC.ae.C38.1 |
| 0.870563024 | 21.86802394 | PC.ae.C42.3 (interacts with) SM.C18.1 |
| 0.870510924 | 35.28196988 | PC.aa.C28.1 (interacts with) PC.ae.C34.0 |
| 0.870457997 | 14.49228961 | PC.aa.C42.1 (interacts with) SM.C18.0 |
| 0.870165133 | 9.899239689 | PC.ae.C36.5 (interacts with) SM.OH.C22.2 |
| 0.869965325 | 18.32515151 | PC.ae.C38.3 (interacts with) SM.OH.C14.1 |
| 0.869898641 | 4.747074886 | PC.ae.C30.0 (interacts with) PC.ae.C42.5 |
| 0.869876379 | 10.43774515 | PC.aa.C38.0 (interacts with) SM.OH.C22.1 |
| 0.869826322 | 4.05379007 | PC.ae.C38.5 (interacts with) PC.ae.C44.6 |
| 0.86981314 | 107.4748627 | PC.aa.C42.6 (interacts with) PC.ae.C42.2 |
| 0.86969078 | 17.99784819 | PC.aa.C36.6 (interacts with) PC.aa.C42.5 |
| 0.869257064 | 20.07311799 | Met (interacts with) Phe |
| 0.869154875 | 162.5462842 | SM.C24.0 (interacts with) SM.C24.1 |
| 0.869135437 | 3.91166553 | PC.ae.C36.5 (interacts with) SM.OH.C16.1 |
| 0.868818043 | 25.86424631 | PC.ae.C38.5 (interacts with) PC.ae.C42.3 |
| 0.868766033 | 3.650093894 | PC.ae.C36.5 (interacts with) SM.C18.1 |
| 0.86876551 | 15.03563513 | PC.ae.C30.0 (interacts with) PC.ae.C36.1 |
| 0.868764981 | 5.89040448 | PC.aa.C42.1 (interacts with) SM.OH.C14.1 |
| 0.868634549 | 5.118660833 | PC.aa.C42.1 (interacts with) SM.OH.C16.1 |
| 0.868348869 | 5.604634997 | PC.ae.C34.3 (interacts with) PC.ae.C38.5 |
| 0.868210279 | 58.08065712 | PC.aa.C42.6 (interacts with) PC.ae.C36.0 |
| 0.867999213 | 14.24686999 | PC.ae.C44.6 (interacts with) SM.C24.1 |
| 0.867652404 | 22.81022522 | PC.aa.C32.0 (interacts with) PC.ae.C34.0 |
| 0.867437817 | 5.174488531 | PC.ae.C34.1 (interacts with) PC.ae.C40.5 |
| 0.867354275 | 5.732699195 | PC.ae.C32.2 (interacts with) PC.ae.C40.2 |
| 0.867331542 | 11.294293 | PC.ae.C40.5 (interacts with) SM.OH.C22.2 |
| 0.86718574 | 16.13442214 | PC.ae.C36.4 (interacts with) PC.ae.C40.4 |
| 0.867104351 | 33.07866191 | PC.ae.C34.3 (interacts with) SM.C16.0 |
| 0.867057459 | 5.757159199 | PC.ae.C34.2 (interacts with) PC.ae.C38.5 |
| 0.866857689 | 16.50700765 | PC.aa.C38.0 (interacts with) PC.aa.C38.1 |
| 0.86669074 | 13.03218366 | PC.aa.C28.1 (interacts with) SM.C16.1 |
| 0.866680855 | 26.04989497 | PC.ae.C44.5 (interacts with) SM.C16.0 |
| 0.866308719 | 175.7852065 | His (interacts with) total.DMA |
| 0.866273381 | 15.56136741 | PC.aa.C28.1 (interacts with) SM.OH.C22.1 |
| 0.866169884 | 22.42678634 | PC.ae.C32.1 (interacts with) PC.ae.C38.3 |
| 0.86615064 | 7.424752738 | PC.ae.C38.6 (interacts with) PC.ae.C42.5 |
| 0.865925792 | 11.26244864 | PC.aa.C38.0 (interacts with) SM.C16.1 |
| 0.865490296 | 4.944795319 | PC.ae.C34.2 (interacts with) PC.ae.C38.4 |
| 0.865397693 | 2.791241793 | PC.ae.C30.0 (interacts with) PC.ae.C40.4 |
| 0.865359064 | 90.76669946 | PC.aa.C32.0 (interacts with) PC.aa.C36.1 |
| 0.864841501 | 5.487914724 | PC.ae.C34.1 (interacts with) PC.ae.C38.6 |
| 0.864727741 | 3.419431137 | PC.ae.C36.5 (interacts with) PC.ae.C40.3 |
| 0.864602794 | 11.28193999 | PC.ae.C38.4 (interacts with) SM.OH.C22.2 |
| 0.864335727 | 12.55294946 | PC.ae.C30.0 (interacts with) SM.C16.1 |
| 0.864157656 | 221.4901397 | PC.aa.C28.1 (interacts with) PC.ae.C40.1 |
| 0.864151806 | 30.02055138 | Phe (interacts with) Val |
| 0.863995607 | 165.1065841 | PC.aa.C34.2 (interacts with) PC.aa.C36.3 |
| 0.863926612 | 14.24011551 | PC.ae.C36.5 (interacts with) PC.ae.C38.3 |
| 0.863210863 | 28.29041229 | PC.aa.C40.4 (interacts with) PC.aa.C40.5 |
| 0.863163946 | 21.45456337 | PC.aa.C34.2 (interacts with) PC.ae.C36.2 |
| 0.862995486 | 2 | C2 (interacts with) C14.2 |
| 0.862885681 | 24.36830503 | Asn (interacts with) Phe |
| 0.862547896 | 6.666891353 | PC.ae.C32.1 (interacts with) PC.ae.C36.3 |
| 0.862547492 | 3.731145928 | PC.ae.C38.5 (interacts with) SM.OH.C16.1 |
| 0.862519343 | 7.116451976 | PC.aa.C38.0 (interacts with) PC.ae.C38.5 |
| 0.862298276 | 16.41831299 | PC.ae.C40.2 (interacts with) PC.ae.C42.3 |
| 0.862027156 | 14.52933844 | PC.aa.C34.3 (interacts with) PC.aa.C36.3 |
| 0.862023002 | 17.270294 | PC.ae.C34.1 (interacts with) PC.ae.C34.3 |
| 0.862002397 | 29.47105227 | PC.ae.C38.3 (interacts with) PC.ae.C42.4 |
| 0.86194416 | 226.3116647 | Ala (interacts with) Cit |
| 0.861724228 | 14.28838866 | PC.ae.C30.0 (interacts with) PC.ae.C42.3 |
| 0.86157465 | 54.85036331 | PC.ae.C32.2 (interacts with) PC.ae.C42.2 |
| 0.861408831 | 230.7378984 | Asn (interacts with) Thr |
| 0.861366052 | 58.62495749 | PC.aa.C40.6 (interacts with) PC.ae.C36.0 |
| 0.861314812 | 15.86083328 | PC.aa.C28.1 (interacts with) PC.ae.C34.1 |
| 0.861089683 | 5.209702278 | PC.ae.C40.2 (interacts with) SM.C18.1 |
| 0.861043676 | 9.899807034 | PC.ae.C36.5 (interacts with) SM.OH.C22.1 |
| 0.860989557 | 4.813492063 | C0 (interacts with) Creatinine |
| 0.860691373 | 11.25629731 | PC.ae.C30.0 (interacts with) SM.OH.C22.2 |
| 0.860522621 | 5.258486385 | PC.ae.C36.5 (interacts with) PC.ae.C40.2 |
| 0.860429288 | 18.97191159 | PC.ae.C34.1 (interacts with) SM.C18.0 |
| 0.860185596 | 210 | lysoPC.a.C16.0 (interacts with) lysoPC.a.C20.4 |
| 0.859676626 | 25.81558442 | Leu (interacts with) Phe |
| 0.859399851 | 7.813539299 | PC.ae.C40.6 (interacts with) PC.ae.C42.5 |
| 0.859320159 | 6.488220096 | PC.ae.C32.1 (interacts with) PC.ae.C40.5 |
| 0.858586159 | 31.38548765 | PC.aa.C34.2 (interacts with) PC.ae.C36.3 |
| 0.858466414 | 14.52826212 | PC.aa.C30.0 (interacts with) PC.ae.C38.2 |
| 0.858396559 | 5.442405725 | PC.ae.C40.5 (interacts with) SM.C18.1 |
| 0.858314609 | 25.81050454 | PC.ae.C34.2 (interacts with) SM.C16.1 |
| 0.858265081 | 7.402842369 | PC.ae.C38.5 (interacts with) PC.ae.C40.5 |
| 0.85822333 | 40.29336913 | PC.aa.C36.5 (interacts with) PC.ae.C38.0 |
| 0.857954173 | 30.31574104 | PC.ae.C36.0 (interacts with) PC.ae.C38.0 |
| 0.857859545 | 9.505081245 | PC.aa.C42.1 (interacts with) PC.ae.C40.6 |
| 0.857430818 | 6.80164629 | PC.ae.C32.1 (interacts with) PC.ae.C38.6 |
| 0.857324887 | 5.423768333 | PC.ae.C36.5 (interacts with) PC.ae.C42.4 |
| 0.857221007 | 37.94651485 | PC.aa.C32.0 (interacts with) PC.ae.C38.6 |
| 0.85719071 | 5.084225103 | PC.ae.C36.4 (interacts with) PC.ae.C42.5 |
| 0.856994707 | 6.740973962 | PC.ae.C40.2 (interacts with) PC.ae.C42.5 |
| 0.85698762 | 17.00365717 | PC.ae.C36.1 (interacts with) SM.OH.C16.1 |
| 0.856636711 | 29.93721805 | Trp (interacts with) Val |
| 0.856334031 | 9.593334892 | PC.ae.C36.2 (interacts with) PC.ae.C36.4 |
| 0.856316967 | 45.60775316 | PC.ae.C40.2 (interacts with) PC.ae.C42.2 |
| 0.856045388 | 11.97660405 | PC.ae.C36.5 (interacts with) PC.ae.C42.3 |
| 0.855884204 | 5.11219713 | PC.ae.C32.1 (interacts with) PC.ae.C36.4 |
| 0.855875776 | 11.96717915 | PC.ae.C40.3 (interacts with) SM.C24.1 |
| 0.85574334 | 28.90056867 | PC.ae.C36.0 (interacts with) PC.ae.C40.2 |
| 0.85570092 | 12.30916622 | PC.ae.C42.5 (interacts with) SM.C24.1 |
| 0.855658177 | 31.88832391 | PC.aa.C34.2 (interacts with) PC.ae.C34.2 |
| 0.855609428 | 12.14179868 | PC.aa.C38.0 (interacts with) SM.C16.0 |
| 0.855561396 | 13.79777796 | PC.ae.C32.2 (interacts with) PC.ae.C34.2 |
| 0.855259526 | 16.66009301 | PC.ae.C34.1 (interacts with) PC.ae.C44.6 |
| 0.854924999 | 21.37110872 | PC.ae.C36.1 (interacts with) PC.ae.C38.5 |
| 0.854825259 | 6.504795052 | PC.ae.C34.2 (interacts with) PC.ae.C40.3 |
| 0.85477515 | 21.83407172 | PC.aa.C30.0 (interacts with) PC.ae.C40.1 |
| 0.854723877 | 17.71759864 | PC.aa.C42.0 (interacts with) PC.ae.C36.1 |
| 0.854383358 | 13.9952263 | PC.ae.C38.3 (interacts with) SM.C16.0 |
| 0.854185752 | 21.04602413 | PC.ae.C42.3 (interacts with) SM.OH.C14.1 |
| 0.854116387 | 3.796825397 | Phe (interacts with) total.DMA |
| 0.854015604 | 12.27819365 | PC.ae.C34.0 (interacts with) PC.ae.C42.3 |
| 0.853666073 | 35.16487339 | PC.ae.C34.3 (interacts with) SM.C16.1 |
| 0.853665586 | 104.3281359 | PC.aa.C40.6 (interacts with) PC.ae.C42.2 |
| 0.853662459 | 44.65091082 | PC.aa.C34.4 (interacts with) PC.aa.C36.1 |
| 0.853455343 | 154.845485 | PC.aa.C36.2 (interacts with) PC.ae.C38.3 |
| 0.852751004 | 7.045364956 | PC.ae.C36.2 (interacts with) SM.C18.1 |
| 0.852373987 | 196.9970722 | Orn (interacts with) Pro |
| 0.852365989 | 14.66904363 | PC.aa.C42.1 (interacts with) SM.C24.1 |
| 0.851905623 | 14.650902 | PC.ae.C40.3 (interacts with) PC.ae.C44.5 |
| 0.851537873 | 15.11682131 | PC.ae.C32.2 (interacts with) PC.ae.C38.3 |
| 0.851511967 | 48.60512638 | PC.aa.C32.0 (interacts with) PC.ae.C34.1 |
| 0.851357852 | 12.11060105 | PC.ae.C30.0 (interacts with) SM.C16.0 |
| 0.851066263 | 24.64079182 | PC.ae.C34.2 (interacts with) SM.C16.0 |
| 0.850918592 | 25.86320346 | Leu (interacts with) total.DMA |
| 0.850809485 | 27.60314685 | PC.aa.C30.0 (interacts with) PC.aa.C32.3 |
| 0.850764515 | 11.16904175 | PC.ae.C38.4 (interacts with) SM.OH.C22.1 |
| 0.850586814 | 9.574642739 | PC.aa.C38.0 (interacts with) PC.aa.C42.1 |
| 0.850477404 | 12.55481596 | PC.ae.C36.5 (interacts with) SM.C18.0 |
| 0.850412897 | 8.908552089 | PC.ae.C36.1 (interacts with) SM.C16.1 |
| 0.850272441 | 11.68682075 | PC.ae.C40.2 (interacts with) SM.C16.0 |
| 0.850201836 | 6.296825397 | C0 (interacts with) total.DMA |
| 0.85017143 | 83.27223112 | PC.aa.C38.5 (interacts with) PC.ae.C36.0 |
| 0.85006316 | 12.53345879 | PC.ae.C38.6 (interacts with) SM.OH.C22.2 |
| 0.849939993 | 26.64205147 | PC.aa.C34.4 (interacts with) PC.aa.C36.3 |
| 0.849677079 | 9.010625032 | PC.ae.C36.2 (interacts with) SM.OH.C16.1 |
| 0.849641325 | 29.53570467 | PC.aa.C28.1 (interacts with) PC.aa.C36.0 |
| 0.849575379 | 7.111882259 | PC.ae.C36.3 (interacts with) PC.ae.C40.3 |
| 0.849382633 | 9.571870246 | PC.aa.C42.1 (interacts with) PC.ae.C40.5 |
| 0.849323112 | 10.73047746 | PC.ae.C38.5 (interacts with) SM.OH.C22.1 |
| 0.84930886 | 17.9242431 | PC.ae.C30.0 (interacts with) PC.ae.C38.3 |
| 0.849143447 | 4.166666667 | C0 (interacts with) Phe |
| 0.848996225 | 14.05598173 | PC.ae.C40.6 (interacts with) SM.OH.C22.1 |
| 0.84895486 | 3.769839936 | PC.aa.C42.0 (interacts with) PC.ae.C40.4 |
| 0.848954446 | 23.02615253 | PC.ae.C38.3 (interacts with) PC.ae.C42.5 |
| 0.848843106 | 41.84747444 | PC.aa.C32.3 (interacts with) PC.ae.C38.3 |
| 0.848713091 | 4.787465338 | PC.aa.C42.1 (interacts with) PC.ae.C32.1 |
| 0.848545647 | 32.57210845 | PC.ae.C40.2 (interacts with) SM.OH.C24.1 |
| 0.848459866 | 618 | Kynurenine (interacts with) Putrescine |
| 0.848459481 | 42.35511484 | PC.aa.C38.3 (interacts with) PC.aa.C38.4 |
| 0.848340149 | 8.27052172 | PC.ae.C36.2 (interacts with) PC.ae.C40.4 |
| 0.848283424 | 61.92084734 | PC.aa.C32.0 (interacts with) PC.aa.C38.6 |
| 0.847992775 | 26.3512987 | Leu (interacts with) Trp |
| 0.847940847 | 6.334495765 | PC.aa.C42.0 (interacts with) PC.ae.C34.1 |
| 0.847928277 | 12.16630908 | PC.ae.C38.5 (interacts with) SM.C24.1 |
| 0.847822908 | 15.41244423 | PC.aa.C32.0 (interacts with) PC.ae.C38.2 |
| 0.847700424 | 7.731954837 | PC.ae.C38.5 (interacts with) PC.ae.C38.6 |
| 0.847444493 | 11.09633595 | PC.aa.C36.0 (interacts with) SM.OH.C16.1 |
| 0.847407763 | 14.47414678 | PC.ae.C32.2 (interacts with) SM.OH.C22.2 |
| 0.8472642 | 64.43963923 | PC.aa.C38.6 (interacts with) PC.ae.C34.0 |
| 0.847234576 | 8.647478546 | PC.aa.C42.1 (interacts with) PC.ae.C40.4 |
| 0.847076666 | 24.79030043 | PC.aa.C32.0 (interacts with) PC.ae.C36.0 |
| 0.84702235 | 10.46089776 | PC.aa.C36.0 (interacts with) PC.ae.C32.2 |
| 0.846954619 | 5.618683127 | PC.aa.C42.1 (interacts with) PC.ae.C42.4 |
| 0.846766294 | 46.21574078 | PC.aa.C38.4 (interacts with) PC.ae.C40.1 |
| 0.846623773 | 67.07069632 | PC.ae.C38.2 (interacts with) PC.ae.C38.3 |
| 0.846583246 | 26.5422609 | PC.ae.C36.3 (interacts with) SM.C16.1 |
| 0.84642154 | 6.234904403 | PC.ae.C36.4 (interacts with) PC.ae.C44.5 |
| 0.846317666 | 11.39960197 | PC.ae.C32.1 (interacts with) SM.C18.0 |
| 0.846286952 | 17.11608215 | PC.aa.C36.1 (interacts with) PC.ae.C38.0 |
| 0.846253737 | 12.57950537 | PC.aa.C36.0 (interacts with) PC.ae.C36.5 |
| 0.845712798 | 35.27138973 | PC.ae.C42.3 (interacts with) SM.C16.0 |
| 0.845523502 | 317.2692112 | PC.ae.C38.3 (interacts with) PC.ae.C40.1 |
| 0.845419999 | 5.145353592 | PC.ae.C42.4 (interacts with) SM.OH.C16.1 |
| 0.845416221 | 26.07868142 | PC.aa.C28.1 (interacts with) PC.ae.C40.3 |
| 0.845322379 | 4.400780552 | PC.ae.C38.4 (interacts with) SM.OH.C16.1 |
| 0.845158981 | 50.34429726 | PC.aa.C36.5 (interacts with) PC.aa.C38.6 |
| 0.84487553 | 32.95850103 | PC.aa.C34.2 (interacts with) PC.ae.C34.1 |
| 0.844705954 | 30.83834947 | PC.aa.C36.1 (interacts with) PC.aa.C36.2 |
| 0.844658779 | 17.7383083 | PC.ae.C44.5 (interacts with) SM.C18.0 |
| 0.844555184 | 12.12230911 | PC.ae.C40.2 (interacts with) SM.C16.1 |
| 0.84444295 | 25.32754354 | PC.ae.C36.3 (interacts with) SM.C16.0 |
| 0.844269926 | 24.22539683 | Cit (interacts with) Thr |
| 0.844247272 | 4.001658341 | PC.ae.C38.4 (interacts with) PC.ae.C44.6 |
| 0.844137264 | 16.74693498 | Lys (interacts with) total.DMA |
| 0.844054701 | 24.90401931 | Asn (interacts with) Trp |
| 0.843861782 | 61.77482442 | PC.aa.C34.2 (interacts with) PC.ae.C40.1 |
| 0.843818061 | 12.57020015 | PC.ae.C38.6 (interacts with) SM.OH.C22.1 |
| 0.843724157 | 3.98015873 | Phe (interacts with) Creatinine |
| 0.843533737 | 19.81436267 | PC.aa.C30.0 (interacts with) PC.aa.C36.1 |
| 0.843387268 | 16.45763774 | PC.aa.C42.0 (interacts with) PC.ae.C36.0 |
| 0.843360859 | 113.0399993 | PC.aa.C38.5 (interacts with) PC.ae.C42.2 |
| 0.843330806 | 36.39315205 | PC.ae.C42.3 (interacts with) PC.ae.C42.4 |
| 0.843317804 | 195.9083732 | SM.C18.0 (interacts with) SM.C24.0 |
| 0.843009149 | 6.5452596 | PC.aa.C42.1 (interacts with) PC.ae.C30.0 |
| 0.842932216 | 6.397244147 | PC.ae.C32.1 (interacts with) PC.ae.C40.2 |
| 0.84273976 | 13.58954652 | PC.aa.C32.3 (interacts with) PC.ae.C34.2 |
| 0.842536504 | 7.919341627 | PC.aa.C42.1 (interacts with) PC.ae.C40.2 |
| 0.842308556 | 16.94299439 | PC.aa.C36.1 (interacts with) PC.aa.C42.5 |
| 0.842252743 | 5.99616334 | PC.ae.C40.6 (interacts with) SM.C18.1 |
| 0.842053443 | 17.87695192 | PC.ae.C36.5 (interacts with) SM.C24.1 |
| 0.84189962 | 12.40237178 | PC.ae.C40.5 (interacts with) SM.C16.1 |
| 0.841838418 | 727.2575508 | PC.ae.C38.3 (interacts with) SM.C24.0 |
| 0.841802082 | 296.0726567 | PC.ae.C36.1 (interacts with) PC.ae.C40.1 |
| 0.841765745 | 14.90066961 | PC.ae.C40.2 (interacts with) SM.C18.0 |
| 0.841528209 | 21.97327216 | PC.aa.C28.1 (interacts with) SM.OH.C14.1 |
| 0.841225925 | 170.7813311 | lysoPC.a.C18.1 (interacts with) PC.aa.C36.3 |
| 0.840942209 | 6.752591136 | PC.ae.C34.1 (interacts with) PC.ae.C40.2 |
| 0.840836452 | 45.15268803 | PC.aa.C32.0 (interacts with) PC.aa.C38.0 |
| 0.840583291 | 6.046806973 | PC.ae.C34.3 (interacts with) PC.ae.C40.3 |
| 0.840371644 | 11.97589763 | PC.aa.C42.1 (interacts with) PC.ae.C32.2 |
| 0.840180584 | 6.270845058 | PC.ae.C36.2 (interacts with) PC.ae.C36.5 |
| 0.840034073 | 21.15391267 | PC.ae.C32.1 (interacts with) PC.ae.C36.1 |
| 0.840024735 | 19.48044196 | PC.ae.C38.3 (interacts with) SM.C18.1 |
| 0.839979118 | 11.9803236 | PC.aa.C36.0 (interacts with) PC.ae.C42.3 |
| 0.83979306 | 40.90605522 | PC.aa.C32.0 (interacts with) PC.ae.C40.5 |
| 0.839739763 | 20.50534234 | PC.aa.C36.3 (interacts with) PC.aa.C40.4 |
| 0.839626611 | 210 | Glu (interacts with) lysoPC.a.C20.3 |
| 0.839599039 | 11.3925423 | PC.ae.C40.5 (interacts with) SM.OH.C22.1 |
| 0.839540716 | 15.85590004 | PC.ae.C38.2 (interacts with) PC.ae.C42.3 |
| 0.83943383 | 30.02055138 | Tyr (interacts with) Val |
| 0.839066861 | 20.61827425 | PC.ae.C40.2 (interacts with) SM.C24.1 |
| 0.838980363 | 14.06926755 | PC.ae.C32.2 (interacts with) PC.ae.C34.0 |
| 0.838940393 | 17.58097585 | PC.aa.C28.1 (interacts with) PC.ae.C32.2 |
| 0.838071729 | 6.149464865 | PC.aa.C42.0 (interacts with) SM.C18.1 |
| 0.8380083 | 12.63767467 | SM.OH.C16.1 (interacts with) SM.C24.1 |
| 0.837968864 | 788.571832 | lysoPC.a.C16.1 (interacts with) lysoPC.a.C20.3 |
| 0.837472252 | 122.2967265 | PC.aa.C32.0 (interacts with) PC.aa.C38.4 |
| 0.837431513 | 6.172733031 | PC.ae.C34.1 (interacts with) PC.ae.C40.6 |
| 0.836893388 | 11.54725263 | PC.ae.C32.1 (interacts with) SM.OH.C22.1 |
| 0.83663771 | 6.515871709 | PC.aa.C38.0 (interacts with) PC.ae.C40.3 |
| 0.836560429 | 383.7413348 | Ala (interacts with) Thr |
| 0.836426094 | 15.21398687 | PC.aa.C28.1 (interacts with) PC.ae.C40.4 |
| 0.835818476 | 18.68872216 | PC.aa.C32.3 (interacts with) PC.ae.C34.1 |
| 0.835706945 | 68.13180143 | PC.aa.C32.0 (interacts with) PC.ae.C38.0 |
| 0.835685496 | 11.26689824 | PC.ae.C30.0 (interacts with) SM.OH.C22.1 |
| 0.835430776 | 5.720003042 | PC.ae.C38.6 (interacts with) SM.C18.1 |
| 0.835275806 | 18.12313785 | PC.ae.C36.2 (interacts with) PC.ae.C38.2 |
| 0.835267795 | 19.3514831 | PC.ae.C36.1 (interacts with) PC.ae.C40.3 |
| 0.835176934 | 78.530409 | PC.aa.C32.0 (interacts with) PC.ae.C38.1 |
| 0.834881651 | 10.79346184 | PC.ae.C42.5 (interacts with) SM.OH.C22.1 |
| 0.834218654 | 30.35645281 | PC.aa.C36.1 (interacts with) PC.aa.C36.3 |
| 0.833939829 | 6.749916466 | PC.aa.C36.0 (interacts with) PC.ae.C30.0 |
| 0.833774944 | 9.487539575 | PC.aa.C42.0 (interacts with) PC.aa.C42.1 |
| 0.833731919 | 8.431436966 | PC.ae.C30.0 (interacts with) PC.ae.C36.2 |
| 0.83357518 | 6.837976387 | PC.ae.C40.3 (interacts with) PC.ae.C40.5 |
| 0.833572014 | 4.854614638 | PC.ae.C30.0 (interacts with) PC.ae.C38.5 |
| 0.832974674 | 13.72102474 | PC.ae.C38.6 (interacts with) SM.C16.1 |
| 0.832941087 | 7.197934167 | PC.ae.C32.2 (interacts with) PC.ae.C38.4 |
| 0.832847988 | 6.652120529 | PC.ae.C40.2 (interacts with) PC.ae.C40.3 |
| 0.832846023 | 31.56972016 | Cit (interacts with) Orn |
| 0.832458249 | 14.23413459 | PC.ae.C36.2 (interacts with) PC.ae.C42.3 |
| 0.832194539 | 25.81558442 | Leu (interacts with) Tyr |
| 0.832107402 | 20.35300297 | PC.ae.C36.1 (interacts with) PC.ae.C36.2 |
| 0.832078496 | 6.636030493 | PC.ae.C34.2 (interacts with) PC.ae.C42.5 |
| 0.832027565 | 6.063328769 | PC.ae.C36.4 (interacts with) SM.C18.1 |
| 0.831954222 | 125.9219303 | PC.aa.C40.5 (interacts with) PC.ae.C40.1 |
| 0.831790653 | 5.035714286 | C0 (interacts with) Trp |
| 0.831650017 | 7.180909042 | PC.ae.C32.1 (interacts with) PC.ae.C40.6 |
| 0.831031138 | 31.33848049 | PC.ae.C42.3 (interacts with) SM.OH.C22.1 |
| 0.830898376 | 21.70944742 | PC.ae.C36.1 (interacts with) PC.ae.C42.5 |
| 0.830885658 | 16.44989011 | PC.aa.C42.0 (interacts with) SM.OH.C22.2 |
| 0.830817947 | 99.33333333 | C0 (interacts with) C4 |
| 0.830603869 | 13.97322245 | PC.aa.C40.5 (interacts with) PC.ae.C38.1 |
| 0.830517909 | 21.02876501 | PC.aa.C34.1 (interacts with) PC.aa.C38.5 |
| 0.830244799 | 6.005521406 | PC.ae.C34.3 (interacts with) PC.ae.C42.5 |
| 0.829946308 | 5.151005734 | PC.ae.C36.5 (interacts with) PC.ae.C40.6 |
| 0.829820412 | 13.19753378 | PC.ae.C40.5 (interacts with) SM.C16.0 |
| 0.829520571 | 24.48991363 | PC.ae.C38.2 (interacts with) PC.ae.C42.2 |
| 0.82927096 | 13.35044884 | PC.ae.C42.5 (interacts with) PC.ae.C44.5 |
| 0.829245133 | 8.34503493 | PC.aa.C36.0 (interacts with) PC.ae.C40.4 |
| 0.829238264 | 21.77891037 | PC.aa.C36.5 (interacts with) PC.aa.C40.5 |
| 0.828567711 | 71.43047011 | PC.aa.C32.1 (interacts with) PC.aa.C42.5 |
| 0.828494342 | 15.62466079 | PC.ae.C32.2 (interacts with) PC.ae.C44.6 |
| 0.828357693 | 16.90543182 | PC.aa.C38.0 (interacts with) SM.C18.0 |
| 0.828223848 | 51.63171418 | PC.aa.C32.0 (interacts with) PC.ae.C40.4 |
| 0.828160071 | 8.644248123 | PC.aa.C42.1 (interacts with) PC.ae.C44.5 |
| 0.828059959 | 22.68394618 | PC.ae.C36.1 (interacts with) PC.ae.C38.4 |
| 0.828009686 | 11.05080213 | PC.ae.C30.0 (interacts with) PC.ae.C34.0 |
| 0.827777864 | 31.26533137 | PC.aa.C32.0 (interacts with) PC.aa.C34.2 |
| 0.827427098 | 54.28040776 | PC.ae.C36.4 (interacts with) PC.ae.C38.3 |
| 0.827220093 | 3.98015873 | Tyr (interacts with) Creatinine |
| 0.827147063 | 15.24345096 | PC.aa.C38.0 (interacts with) PC.ae.C38.3 |
| 0.827055243 | 29.16471576 | PC.aa.C34.1 (interacts with) PC.aa.C36.3 |
| 0.82699294 | 34.19205453 | PC.aa.C32.0 (interacts with) PC.aa.C36.2 |
| 0.8268489 | 26.95700637 | PC.ae.C38.4 (interacts with) PC.ae.C42.3 |
| 0.826681209 | 45.22904752 | PC.aa.C32.0 (interacts with) PC.ae.C32.2 |
| 0.826485789 | 17.57775417 | PC.ae.C36.4 (interacts with) SM.C18.0 |
| 0.826448506 | 67.625463 | PC.aa.C34.1 (interacts with) PC.aa.C38.3 |
| 0.826277686 | 17.48789647 | PC.ae.C36.1 (interacts with) SM.C18.1 |
| 0.825643759 | 17.53903377 | PC.ae.C38.3 (interacts with) PC.ae.C38.6 |
| 0.82562856 | 3.816035753 | PC.ae.C30.0 (interacts with) PC.ae.C36.5 |
| 0.825513858 | 45.74366163 | PC.ae.C30.0 (interacts with) PC.ae.C42.2 |
| 0.825460391 | 8.162047227 | PC.aa.C30.0 (interacts with) PC.aa.C36.2 |
| 0.82524859 | 18.1270489 | lysoPC.a.C18.0 (interacts with) PC.aa.C38.3 |
| 0.825137562 | 15.34825265 | PC.aa.C42.1 (interacts with) PC.ae.C34.1 |
| 0.825048901 | 18.22284679 | PC.ae.C36.2 (interacts with) SM.OH.C22.2 |
| 0.824849729 | 11.93843511 | PC.ae.C36.1 (interacts with) SM.OH.C22.2 |
| 0.82474861 | 22.57635242 | PC.ae.C34.0 (interacts with) SM.OH.C14.1 |
| 0.824688864 | 84.76241121 | PC.aa.C36.3 (interacts with) PC.ae.C40.1 |
| 0.824638273 | 3.333333333 | Creatinine (interacts with) total.DMA |
| 0.824514776 | 28.98037758 | SM.OH.C14.1 (interacts with) SM.OH.C24.1 |
| 0.824419475 | 816 | Gln (interacts with) Kynurenine |
| 0.82425182 | 20.28359496 | PC.ae.C32.2 (interacts with) PC.ae.C38.2 |
| 0.824214722 | 23.73510422 | PC.aa.C34.2 (interacts with) PC.ae.C42.3 |
| 0.823931165 | 20.89708757 | PC.ae.C34.0 (interacts with) SM.OH.C16.1 |
| 0.823850509 | 16.31241932 | PC.ae.C38.3 (interacts with) PC.ae.C40.5 |
| 0.823777335 | 20.519195 | Lys (interacts with) Creatinine |
| 0.823694891 | 194.1325031 | PC.aa.C28.1 (interacts with) PC.aa.C30.0 |
| 0.823542281 | 172.2298376 | PC.aa.C34.3 (interacts with) PC.ae.C40.1 |
| 0.82329972 | 181.1902375 | lysoPC.a.C16.1 (interacts with) PC.aa.C38.3 |
| 0.823263876 | 17.83993432 | PC.aa.C36.1 (interacts with) PC.aa.C38.5 |
| 0.823254601 | 106.3570657 | C3 (interacts with) Orn |
| 0.823098968 | 276.6985017 | His (interacts with) Creatinine |
| 0.822987069 | 41.82892436 | PC.aa.C36.4 (interacts with) PC.aa.C38.3 |
| 0.822961651 | 12.12665245 | PC.ae.C38.4 (interacts with) SM.C24.1 |
| 0.822864667 | 19.69462353 | PC.aa.C34.1 (interacts with) PC.aa.C42.5 |
| 0.822836252 | 89.41077622 | PC.aa.C34.3 (interacts with) PC.aa.C36.2 |
| 0.82265667 | 241.6995635 | PC.aa.C40.4 (interacts with) PC.ae.C42.3 |
| 0.822586885 | 31.05088947 | PC.ae.C44.5 (interacts with) SM.OH.C22.2 |
| 0.822399631 | 22.74103446 | PC.aa.C34.1 (interacts with) PC.ae.C38.0 |
| 0.822395331 | 26.9067368 | PC.aa.C34.1 (interacts with) PC.aa.C34.3 |
| 0.822133552 | 7.202622544 | PC.ae.C30.0 (interacts with) PC.ae.C42.4 |
| 0.821653664 | 3.522339866 | PC.ae.C36.4 (interacts with) PC.ae.C44.6 |
| 0.821577151 | 12.39001908 | PC.ae.C34.0 (interacts with) PC.ae.C40.4 |
| 0.821484101 | 36.14642857 | Ile (interacts with) Trp |
| 0.821317647 | 16.87846639 | PC.aa.C28.1 (interacts with) PC.ae.C36.5 |
| 0.821301456 | 14.49414141 | PC.aa.C36.1 (interacts with) PC.aa.C38.4 |
| 0.821173405 | 15.50705885 | PC.ae.C40.6 (interacts with) SM.C16.1 |
| 0.820835258 | 23.06915721 | Asn (interacts with) total.DMA |
| 0.820791662 | 210 | Spermine (interacts with) Taurine |
| 0.820477041 | 585.3609476 | Ala (interacts with) lysoPC.a.C20.3 |
| 0.82046763 | 39.74920618 | PC.ae.C36.3 (interacts with) PC.ae.C38.3 |
| 0.8203598 | 34.18262336 | PC.ae.C42.3 (interacts with) SM.OH.C22.2 |
| 0.820168209 | 6.970837667 | PC.ae.C38.5 (interacts with) PC.ae.C40.2 |
| 0.820152502 | 38.9207989 | PC.ae.C34.2 (interacts with) PC.ae.C38.3 |
| 0.820067098 | 129.4525676 | PC.ae.C38.3 (interacts with) PC.ae.C42.2 |
| 0.819953992 | 18.31339899 | PC.aa.C28.1 (interacts with) SM.OH.C22.2 |
| 0.819869627 | 4.166666667 | C0 (interacts with) Tyr |
| 0.81983478 | 12.94366962 | PC.ae.C36.1 (interacts with) SM.C16.0 |
| 0.819831572 | 12.51958087 | PC.aa.C38.1 (interacts with) PC.aa.C42.1 |
| 0.819778281 | 3.660317984 | PC.ae.C30.0 (interacts with) SM.C18.1 |
| 0.819721611 | 17.52984959 | PC.ae.C36.2 (interacts with) SM.OH.C22.1 |
| 0.819652571 | 453.1448618 | His (interacts with) Lys |
| 0.819392046 | 18.65413167 | PC.ae.C34.0 (interacts with) PC.ae.C36.5 |
| 0.818896137 | 305.7346052 | C0 (interacts with) His |
| 0.8178924 | 79.64546099 | PC.aa.C34.2 (interacts with) SM.C16.1 |
| 0.817760119 | 17.76459021 | PC.aa.C32.3 (interacts with) PC.ae.C32.2 |
| 0.817698072 | 9.65908952 | PC.aa.C42.1 (interacts with) PC.ae.C38.6 |
| 0.81767624 | 14 | C10.1 (interacts with) C12.1 |
| 0.817586317 | 203.4438567 | Met (interacts with) Orn |
| 0.817452409 | 10.16029341 | PC.ae.C34.3 (interacts with) PC.ae.C44.5 |
| 0.817378342 | 321.390312 | SM.C16.0 (interacts with) SM.C24.0 |
| 0.816991658 | 101.0301209 | C4 (interacts with) Creatinine |
| 0.816959005 | 10.79087978 | PC.ae.C34.2 (interacts with) SM.OH.C14.1 |
| 0.816879291 | 10.53739843 | PC.ae.C30.0 (interacts with) PC.ae.C44.6 |
| 0.816744274 | 143.668362 | PC.aa.C32.0 (interacts with) SM.C16.1 |
| 0.816732541 | 28.38552653 | PC.ae.C36.0 (interacts with) PC.ae.C42.3 |
| 0.816600143 | 50.34429726 | PC.aa.C36.6 (interacts with) PC.aa.C38.6 |
| 0.816308231 | 13.330359 | PC.aa.C32.3 (interacts with) PC.ae.C36.3 |
| 0.81629192 | 12.46456935 | PC.aa.C36.0 (interacts with) SM.OH.C14.1 |
| 0.816247078 | 117.6090663 | PC.aa.C30.0 (interacts with) PC.aa.C34.4 |
| 0.816053899 | 48.84069988 | PC.aa.C36.4 (interacts with) PC.ae.C40.1 |
| 0.815898116 | 641.7413137 | PC.aa.C28.1 (interacts with) SM.C24.0 |
| 0.815429309 | 66.72933871 | PC.ae.C36.1 (interacts with) SM.OH.C24.1 |
| 0.815416279 | 17.57225367 | PC.aa.C42.0 (interacts with) SM.C16.1 |
| 0.815057486 | 210 | Arg (interacts with) Cit |
| 0.814892673 | 39.36208748 | PC.aa.C32.0 (interacts with) PC.ae.C40.6 |
| 0.814882069 | 362.5906157 | Cit (interacts with) Met |
| 0.814590681 | 4.905578803 | PC.aa.C42.1 (interacts with) PC.ae.C38.5 |
| 0.814128645 | 17.72280529 | PC.ae.C40.5 (interacts with) SM.C18.0 |
| 0.814112218 | 3.796825397 | Tyr (interacts with) total.DMA |
| 0.814024501 | 5.151121039 | PC.ae.C34.3 (interacts with) PC.ae.C44.6 |
| 0.813981591 | 607.3688037 | PC.ae.C36.1 (interacts with) SM.C24.0 |
| 0.81394884 | 24.52089071 | SM.OH.C16.1 (interacts with) SM.OH.C24.1 |
| 0.813891843 | 14.51618674 | PC.ae.C38.6 (interacts with) SM.C16.0 |
| 0.813191351 | 175.7623965 | Lys (interacts with) Thr |
| 0.813136434 | 54.01565922 | PC.ae.C44.5 (interacts with) SM.C16.1 |
| 0.813076195 | 112.6458621 | C3 (interacts with) Cit |
| 0.812842484 | 1010 | Asn (interacts with) Gln |
| 0.812538315 | 13.01570693 | SM.OH.C14.1 (interacts with) SM.C24.1 |
| 0.812491557 | 30.54042503 | PC.aa.C30.0 (interacts with) PC.aa.C34.1 |
| 0.812412268 | 18.34990508 | PC.ae.C32.2 (interacts with) PC.ae.C34.3 |
| 0.812170363 | 21.26979398 | PC.ae.C34.1 (interacts with) PC.ae.C38.2 |
| 0.812045969 | 320.5610592 | SM.OH.C22.2 (interacts with) SM.C24.0 |
| 0.812025111 | 7.255022463 | PC.ae.C36.3 (interacts with) PC.ae.C42.5 |
| 0.811919497 | 38.52412483 | PC.aa.C32.0 (interacts with) PC.aa.C32.3 |
| 0.811891129 | 48.42742498 | PC.ae.C34.0 (interacts with) SM.OH.C22.1 |
| 0.811808465 | 19.91515066 | PC.aa.C28.1 (interacts with) SM.C16.0 |
| 0.811687583 | 154.0252484 | PC.aa.C42.5 (interacts with) PC.ae.C42.2 |
| 0.811660205 | 16.16333196 | PC.ae.C40.6 (interacts with) SM.C16.0 |
| 0.810959114 | 39.6223696 | Asn (interacts with) Creatinine |
| 0.810484399 | 32.59442551 | PC.aa.C38.1 (interacts with) PC.ae.C36.0 |
| 0.810141673 | 241.1803646 | Lys (interacts with) Orn |
| 0.810072602 | 18.28139143 | PC.aa.C42.0 (interacts with) SM.C16.0 |
| 0.810060054 | 15.8286883 | PC.ae.C44.5 (interacts with) SM.C24.1 |
| 0.810058438 | 409.8947493 | SM.C16.1 (interacts with) SM.C24.0 |
| 0.809874717 | 88.81433356 | PC.aa.C28.1 (interacts with) PC.ae.C36.0 |
| 0.809757334 | 36.49880952 | Ile (interacts with) Tyr |
| 0.809697348 | 47.070889 | PC.aa.C32.3 (interacts with) PC.ae.C36.1 |
| 0.809218401 | 35.23673469 | C0 (interacts with) Met |
| 0.809157014 | 43.91960094 | PC.ae.C34.0 (interacts with) PC.ae.C38.3 |
| 0.809035465 | 22.80691011 | PC.aa.C36.2 (interacts with) PC.aa.C40.4 |
| 0.808845124 | 7.603494464 | PC.ae.C40.3 (interacts with) PC.ae.C40.6 |
| 0.808640842 | 7.214707903 | PC.ae.C38.6 (interacts with) PC.ae.C40.3 |
| 0.808511282 | 38.1251015 | PC.aa.C34.1 (interacts with) PC.aa.C34.4 |
| 0.808062929 | 32.28559181 | PC.aa.C36.5 (interacts with) PC.ae.C38.1 |
| 0.807926675 | 97.18858354 | PC.aa.C32.1 (interacts with) PC.aa.C38.5 |
| 0.807882302 | 69.20146352 | PC.aa.C36.0 (interacts with) PC.aa.C38.6 |
| 0.807744747 | 62.61863847 | PC.ae.C34.1 (interacts with) PC.ae.C42.2 |
| 0.807583854 | 40.20721258 | PC.aa.C36.0 (interacts with) SM.OH.C22.2 |
| 0.807394938 | 29.97373683 | PC.aa.C34.1 (interacts with) PC.aa.C38.6 |
| 0.80733426 | 70.80516263 | PC.aa.C38.1 (interacts with) SM.OH.C22.2 |
| 0.807253156 | 26.0465368 | Leu (interacts with) Creatinine |
| 0.807111474 | 15.25126598 | PC.ae.C38.3 (interacts with) SM.OH.C22.2 |
| 0.806851672 | 32.28559181 | PC.aa.C36.6 (interacts with) PC.ae.C38.1 |
| 0.806790195 | 3579.428168 | His (interacts with) SM.C24.0 |
| 0.806531142 | 10.73948737 | PC.ae.C34.3 (interacts with) PC.ae.C36.5 |
| 0.806313781 | 125.1347959 | PC.aa.C32.0 (interacts with) PC.aa.C36.4 |
| 0.80613375 | 30.25150376 | Val (interacts with) Creatinine |
| 0.806024759 | 36.49880952 | Ile (interacts with) Phe |
| 0.805981473 | 16.8538765 | PC.ae.C34.0 (interacts with) PC.ae.C34.1 |
| 0.805976953 | 390.1836675 | lysoPC.a.C16.1 (interacts with) PC.aa.C32.1 |
| 0.805973562 | 17.48336188 | PC.aa.C38.1 (interacts with) PC.ae.C34.0 |
| 0.805044012 | 38.81241088 | PC.aa.C32.0 (interacts with) PC.aa.C42.0 |
| 0.80501187 | 15.90540436 | PC.aa.C42.0 (interacts with) SM.OH.C22.1 |
| 0.804870729 | 12.55356955 | PC.ae.C42.4 (interacts with) SM.C24.1 |
| 0.804387398 | 9.393753224 | PC.ae.C34.2 (interacts with) PC.ae.C36.5 |
| 0.804263419 | 14.97042713 | PC.ae.C32.2 (interacts with) PC.ae.C36.3 |
| 0.804243089 | 9.427421824 | PC.ae.C36.5 (interacts with) PC.ae.C44.6 |
| 0.803895426 | 10.51776948 | PC.ae.C36.3 (interacts with) PC.ae.C36.5 |
| 0.803824757 | 25.27509669 | PC.aa.C38.0 (interacts with) SM.C24.1 |
| 0.803709131 | 76.0618571 | PC.ae.C34.0 (interacts with) PC.ae.C38.0 |
| 0.803546462 | 89.04796124 | PC.aa.C36.1 (interacts with) PC.ae.C42.2 |
| 0.803121176 | 62.47281314 | PC.aa.C32.0 (interacts with) PC.ae.C36.5 |
| 0.803096346 | 8.903989672 | PC.aa.C38.0 (interacts with) PC.ae.C38.4 |
| 0.803095409 | 49.91423798 | PC.aa.C34.2 (interacts with) PC.ae.C34.3 |
| 0.803047685 | 23.19100052 | PC.aa.C28.1 (interacts with) SM.C18.1 |
| 0.802883991 | 19.29012792 | PC.ae.C38.3 (interacts with) PC.ae.C40.2 |
| 0.802421043 | 37.68430747 | PC.aa.C34.1 (interacts with) PC.aa.C40.6 |
| 0.802344437 | 11.62031343 | PC.aa.C38.0 (interacts with) PC.ae.C36.2 |
| 0.802332256 | 77.29130488 | PC.aa.C36.2 (interacts with) PC.ae.C36.2 |
| 0.801750491 | 613.5941297 | lysoPC.a.C18.0 (interacts with) PC.ae.C40.1 |
| 0.801672334 | 55.39190871 | PC.aa.C38.6 (interacts with) PC.ae.C40.1 |
| 0.801659188 | 23.06199943 | PC.ae.C42.3 (interacts with) SM.OH.C16.1 |
| 0.801640005 | 9.190380064 | PC.ae.C38.4 (interacts with) PC.ae.C40.5 |
| 0.801519184 | 21.37271181 | PC.ae.C38.3 (interacts with) SM.OH.C16.1 |
| 0.801328937 | 30 | C12.1 (interacts with) C14 |
| 0.801232798 | 13.07636992 | PC.ae.C42.4 (interacts with) SM.OH.C22.1 |
| 0.801103449 | 102.7949565 | PC.aa.C32.1 (interacts with) PC.aa.C40.4 |
| 0.801044677 | 15.84727885 | PC.ae.C36.2 (interacts with) PC.ae.C44.6 |
| 0.80102916 | 11.21194105 | PC.ae.C34.3 (interacts with) SM.OH.C14.1 |
| 0.800865488 | 28.66965485 | PC.aa.C36.1 (interacts with) PC.ae.C38.1 |
| 0.800814632 | 39.66156498 | PC.ae.C38.3 (interacts with) SM.C18.0 |
| 0.800673536 | 51.36146984 | PC.ae.C40.4 (interacts with) PC.ae.C42.2 |
| 0.800069649 | 10.2079743 | C4 (interacts with) Ile |
| 0.800049429 | 29.55091272 | PC.aa.C32.0 (interacts with) PC.aa.C36.0 |

Supplementary Table 9. Network parameters for lung adenocarcinoma group

| Average Shortest  PathLength | Betweenness Centrality | Closeness Centrality | Clustering Coefficient | Degree | Eccentricity | Metabolite | Neighborhood Connectivity | Number Of  Undirected Edges |
| --- | --- | --- | --- | --- | --- | --- | --- | --- |
| 1.895348837 | 0.012956853 | 0.527607362 | 0.680926916 | 34 | 6 | SM.OH.C14.1 | 30.82352941 | 34 |
| 2 | 0.008508598 | 0.5 | 0.717741935 | 32 | 6 | SM.OH.C16.1 | 31.25 | 32 |
| 2.151162791 | 0.022217724 | 0.464864865 | 0.646666667 | 25 | 5 | PC.aa.C38.0 | 26.96 | 25 |
| 2.058139535 | 0.034042959 | 0.485875706 | 0.622988506 | 30 | 5 | PC.ae.C40.6 | 27.2 | 30 |
| 1 | 0 | 1 | 1 | 4 | 1 | C12.1 | 4 | 4 |
| 1 | 0 | 1 | 1 | 4 | 1 | C14.1 | 4 | 4 |
| 2.302325581 | 0.000894647 | 0.434343434 | 0.791666667 | 16 | 7 | PC.ae.C34.2 | 26.75 | 16 |
| 2.139534884 | 0.014915028 | 0.467391304 | 0.689473684 | 20 | 7 | PC.ae.C36.3 | 26.45 | 20 |
| 2.174418605 | 0.003831015 | 0.459893048 | 0.726315789 | 20 | 7 | PC.ae.C34.3 | 27.1 | 20 |
| 2.244186047 | 0.006379387 | 0.445595855 | 0.683823529 | 17 | 7 | PC.aa.C34.2 | 25.47058824 | 17 |
| 2.139534884 | 0.011872294 | 0.467391304 | 0.58008658 | 22 | 7 | PC.aa.C36.2 | 24.45454545 | 22 |
| 1 | 0 | 1 | 1 | 4 | 1 | C14.2 | 4 | 4 |
| 3.023255814 | 0.007221767 | 0.330769231 | 0.714285714 | 14 | 9 | PC.aa.C34.1 | 14.14285714 | 14 |
| 2.662790698 | 0.003627582 | 0.375545852 | 0.791208791 | 14 | 8 | PC.aa.C36.1 | 16.64285714 | 14 |
| 2.058139535 | 0.013037194 | 0.485875706 | 0.632575758 | 33 | 6 | PC.ae.C38.6 | 28.45454545 | 33 |
| 2.023255814 | 0.038398332 | 0.494252874 | 0.627586207 | 30 | 5 | PC.ae.C40.2 | 27.46666667 | 30 |
| 1.988372093 | 0.053530015 | 0.502923977 | 0.664772727 | 33 | 5 | SM.OH.C22.2 | 29.60606061 | 33 |
| 2.465116279 | 8.81954E-05 | 0.405660377 | 0.954545455 | 12 | 7 | PC.aa.C36.5 | 28 | 12 |
| 2.151162791 | 0.025257808 | 0.464864865 | 0.602339181 | 19 | 7 | PC.aa.C38.5 | 25.36842105 | 19 |
| 2.127906977 | 0.002333962 | 0.469945355 | 0.798418972 | 23 | 7 | PC.ae.C36.5 | 32.95652174 | 23 |
| 1.930232558 | 0.014356293 | 0.518072289 | 0.65530303 | 33 | 6 | PC.ae.C38.5 | 30.3030303 | 33 |
| 1.813953488 | 0.033236433 | 0.551282051 | 0.60455192 | 38 | 6 | SM.C16.0 | 29.5 | 38 |
| 1.88372093 | 0.016265198 | 0.530864198 | 0.636507937 | 36 | 6 | SM.C16.1 | 29.94444444 | 36 |
| 2.151162791 | 0.006082229 | 0.464864865 | 0.704615385 | 26 | 6 | PC.aa.C38.6 | 28.19230769 | 26 |
| 2.372093023 | 0.00129896 | 0.421568627 | 0.819047619 | 15 | 6 | PC.aa.C40.6 | 27.46666667 | 15 |
| 1.825581395 | 0.022595565 | 0.547770701 | 0.596491228 | 39 | 6 | PC.ae.C34.0 | 29.76923077 | 39 |
| 1.848837209 | 0.048408157 | 0.540880503 | 0.508974359 | 40 | 7 | PC.ae.C36.1 | 28.225 | 40 |
| 2.348837209 | 0.004127877 | 0.425742574 | 0.791208791 | 14 | 7 | PC.aa.C42.0 | 29.64285714 | 14 |
| 2.418604651 | 0.024557602 | 0.413461538 | 0.692307692 | 14 | 7 | PC.ae.C42.5 | 26.71428571 | 14 |
| 3.197674419 | 0.001737642 | 0.312727273 | 0.75 | 9 | 9 | PC.aa.C32.1 | 13.22222222 | 9 |
| 1.755813953 | 0.035941003 | 0.569536424 | 0.518272425 | 43 | 6 | PC.ae.C38.3 | 27.88372093 | 43 |
| 2.034883721 | 0.008558355 | 0.491428571 | 0.722943723 | 22 | 6 | PC.aa.C42.6 | 29.27272727 | 22 |
| 1.837209302 | 0.028406204 | 0.544303797 | 0.563300142 | 38 | 6 | PC.ae.C38.4 | 28.34210526 | 38 |
| 2.372093023 | 0.000421785 | 0.421568627 | 0.89010989 | 14 | 6 | SM.C18.0 | 30 | 14 |
| 2.313953488 | 0.023825493 | 0.432160804 | 0.725274725 | 14 | 6 | SM.C18.1 | 28.71428571 | 14 |
| 2 | 0.030279845 | 0.5 | 0.692473118 | 31 | 6 | PC.ae.C40.3 | 30.77419355 | 31 |
| 2 | 0.012863968 | 0.5 | 0.665322581 | 32 | 6 | PC.ae.C40.4 | 30.09375 | 32 |
| 1.872093023 | 0.024114067 | 0.534161491 | 0.602495544 | 34 | 6 | PC.aa.C28.1 | 30.05882353 | 34 |
| 1.895348837 | 0.0185663 | 0.527607362 | 0.599190283 | 39 | 6 | SM.OH.C22.1 | 28.51282051 | 39 |
| 1.941860465 | 0.012325076 | 0.51497006 | 0.657142857 | 35 | 6 | SM.OH.C24.1 | 30.2 | 35 |
| 2.406976744 | 0.00869496 | 0.415458937 | 0.723809524 | 15 | 8 | PC.aa.C30.0 | 20.86666667 | 15 |
| 2 | 0.052451231 | 0.5 | 0.476923077 | 26 | 7 | PC.aa.C32.0 | 24.26923077 | 26 |
| 3.372093023 | 0.001349974 | 0.296551724 | 0.666666667 | 3 | 8 | PC.ae.C44.5 | 7.333333333 | 3 |
| 2.744186047 | 0.023929662 | 0.36440678 | 0.533333333 | 6 | 7 | PC.ae.C44.6 | 13.33333333 | 6 |
| 2.546511628 | 0.009476091 | 0.392694064 | 0.669117647 | 17 | 8 | PC.aa.C34.3 | 17.41176471 | 17 |
| 2.441860465 | 0.055132386 | 0.40952381 | 0.533333333 | 21 | 8 | PC.aa.C36.3 | 16.66666667 | 21 |
| 1.965116279 | 0.04311026 | 0.50887574 | 0.53968254 | 28 | 7 | PC.ae.C34.1 | 27.17857143 | 28 |
| 2.26744186 | 0.007614431 | 0.441025641 | 0.484848485 | 12 | 7 | PC.aa.C36.4 | 25.5 | 12 |
| 2.26744186 | 0.004420227 | 0.441025641 | 0.651515152 | 12 | 7 | PC.aa.C38.4 | 30 | 12 |
| 2.476744186 | 0.00999237 | 0.403755869 | 0.640522876 | 18 | 8 | PC.aa.C32.2 | 19.38888889 | 18 |
| 2.534883721 | 0.011179896 | 0.394495413 | 0.683333333 | 16 | 8 | PC.aa.C40.4 | 16.9375 | 16 |
| 2.534883721 | 0.014739471 | 0.394495413 | 0.704761905 | 15 | 8 | PC.aa.C40.5 | 16.73333333 | 15 |
| 2.034883721 | 0.01313314 | 0.491428571 | 0.643076923 | 26 | 7 | PC.ae.C30.0 | 28.80769231 | 26 |
| 1.930232558 | 0.012348529 | 0.518072289 | 0.643678161 | 30 | 6 | SM.C24.0 | 29.7 | 30 |
| 1.965116279 | 0.027747795 | 0.50887574 | 0.580952381 | 36 | 6 | PC.ae.C40.5 | 27.91666667 | 36 |
| 1 | 0 | 1 | 1 | 4 | 1 | C2 | 4 | 4 |
| 2.325581395 | 0.011567885 | 0.43 | 0.639705882 | 17 | 8 | PC.aa.C38.3 | 19.82352941 | 17 |
| 2.209302326 | 0.00314478 | 0.452631579 | 0.680952381 | 21 | 7 | PC.ae.C36.4 | 27.61904762 | 21 |
| 2.372093023 | 0.006139583 | 0.421568627 | 0.747252747 | 14 | 5 | SM.C24.1 | 25.78571429 | 14 |
| 2.069767442 | 0.009154335 | 0.483146067 | 0.675213675 | 27 | 6 | PC.ae.C38.0 | 29.07407407 | 27 |
| 1.930232558 | 0.011149402 | 0.518072289 | 0.643939394 | 33 | 6 | PC.ae.C36.2 | 30.18181818 | 33 |
| 3.73255814 | 3.03998E-05 | 0.267912773 | 0.9 | 5 | 9 | lysoPC.a.C16.1 | 11 | 5 |
| 3.348837209 | 0.000934877 | 0.298611111 | 0.714285714 | 7 | 9 | lysoPC.a.C18.1 | 11 | 7 |
| 5.546511628 | 0.011491108 | 0.180293501 | 0.666666667 | 3 | 8 | Leu | 2.666666667 | 3 |
| 5.546511628 | 0.011491108 | 0.180293501 | 0.666666667 | 3 | 8 | Val | 2.666666667 | 3 |
| 2.11627907 | 0.004709761 | 0.472527473 | 0.710526316 | 20 | 7 | lysoPC.a.C17.0 | 30.85 | 20 |
| 2.127906977 | 0.044317143 | 0.469945355 | 0.447619048 | 21 | 7 | lysoPC.a.C18.0 | 21.85714286 | 21 |
| 1.825581395 | 0.037221414 | 0.547770701 | 0.573076923 | 40 | 6 | PC.ae.C42.3 | 28.975 | 40 |
| 2.26744186 | 0.005958049 | 0.441025641 | 0.712418301 | 18 | 6 | PC.aa.C36.6 | 26.05555556 | 18 |
| 3.186046512 | 0.001768042 | 0.313868613 | 0.777777778 | 10 | 9 | lysoPC.a.C20.3 | 13.3 | 10 |
| 2.639534884 | 0.000278011 | 0.378854626 | 0.822222222 | 10 | 6 | PC.aa.C36.0 | 23.1 | 10 |
| 2.674418605 | 0.000164803 | 0.373913043 | 0.861111111 | 9 | 6 | PC.aa.C38.1 | 22.77777778 | 9 |
| 1.976744186 | 0.008731307 | 0.505882353 | 0.703703704 | 28 | 6 | PC.aa.C42.5 | 30.71428571 | 28 |
| 2.011627907 | 0.031283539 | 0.497109827 | 0.515669516 | 27 | 7 | PC.aa.C32.3 | 24.92592593 | 27 |
| 1.941860465 | 0.012577834 | 0.51497006 | 0.685483871 | 32 | 6 | PC.ae.C32.2 | 30.84375 | 32 |
| 2.337209302 | 0.014926481 | 0.427860697 | 0.596491228 | 19 | 8 | PC.aa.C34.4 | 19.94736842 | 19 |
| 1.790697674 | 0.033912251 | 0.558441558 | 0.573076923 | 40 | 6 | PC.ae.C42.2 | 29.25 | 40 |
| 2.802325581 | 0.020315567 | 0.356846473 | 0.628571429 | 15 | 8 | lysoPC.a.C16.0 | 14.13333333 | 15 |
| 2.5 | 0.000373966 | 0.4 | 0.888888889 | 9 | 6 | PC.ae.C36.0 | 27.22222222 | 9 |
| 2.023255814 | 0.008787203 | 0.494252874 | 0.68226601 | 29 | 7 | PC.ae.C32.1 | 29.68965517 | 29 |
| 2.220930233 | 0.0183125 | 0.45026178 | 0.633333333 | 16 | 7 | PC.ae.C42.4 | 25.125 | 16 |
| 2.093023256 | 0.012057909 | 0.477777778 | 0.595238095 | 21 | 7 | PC.ae.C40.1 | 27.57142857 | 21 |
| 1 | 0 | 1 | 1 | 4 | 1 | C10.1 | 4 | 4 |
| 4.593023256 | 0.068125855 | 0.217721519 | 0.333333333 | 3 | 7 | Ile | 2.666666667 | 3 |
| 1.5 | 0 | 0.666666667 | 0 | 1 | 2 | C14 | 2 | 1 |
| 1 | 1 | 1 | 0 | 2 | 1 | C18.1 | 1 | 2 |
| 3.395348837 | 0 | 0.294520548 | 1 | 3 | 9 | lysoPC.a.C18.2 | 14.33333333 | 3 |
| 1.333333333 | 0.666666667 | 0.75 | 0 | 2 | 2 | Cit | 1.5 | 2 |
| 1.333333333 | 0.666666667 | 0.75 | 0 | 2 | 2 | total.DMA | 1.5 | 2 |
| 2 | 0 | 0.5 | 0 | 1 | 3 | Creatinine | 2 | 1 |
| 2.546511628 | 0 | 0.392694064 | 1 | 3 | 7 | PC.ae.C38.2 | 29.33333333 | 3 |
| 1 | 0 | 1 | 0 | 1 | 1 | Arg | 1 | 1 |
| 1 | 0 | 1 | 0 | 1 | 1 | Met | 1 | 1 |
| 6.523255814 | 0 | 0.153297683 | 1 | 2 | 9 | Trp | 3 | 2 |
| 2.453488372 | 2.73598E-05 | 0.407582938 | 0.952380952 | 7 | 7 | PC.ae.C38.1 | 35.85714286 | 7 |
| 1.5 | 0 | 0.666666667 | 0 | 1 | 2 | C18.2 | 2 | 1 |
| 2 | 0 | 0.5 | 0 | 1 | 3 | C4 | 2 | 1 |
| 3.674418605 | 0.089740082 | 0.272151899 | 0 | 2 | 6 | Ala | 4.5 | 2 |
| 3.720930233 | 0 | 0.26875 | 1 | 2 | 8 | PC.aa.C42.1 | 4.5 | 2 |
| 2.779069767 | 0.110807114 | 0.359832636 | 0.666666667 | 6 | 5 | Pro | 22.33333333 | 6 |
| 3.302325581 | 0 | 0.302816901 | 0 | 1 | 7 | Kynurenine | 14 | 1 |

Supplementary Table 10. Correlation coefficient of nodes from lung adenocarcinoma group

| correlation Coefficient | Edge Betweenness | name |
| --- | --- | --- |
| 0.98143377 | 3.144370748 | SM.OH.C14.1 (interacts with) SM.OH.C16.1 |
| 0.981104988 | 2.81988686 | PC.aa.C38.0 (interacts with) PC.ae.C40.6 |
| 0.980071388 | 2 | C12.1 (interacts with) C14.1 |
| 0.979718058 | 5.111311612 | PC.ae.C34.2 (interacts with) PC.ae.C36.3 |
| 0.97961588 | 3.12042199 | PC.ae.C34.3 (interacts with) PC.ae.C36.3 |
| 0.978433615 | 3.467255158 | PC.aa.C34.2 (interacts with) PC.aa.C36.2 |
| 0.976955083 | 2 | C14.1 (interacts with) C14.2 |
| 0.974691139 | 5.858708677 | PC.aa.C34.1 (interacts with) PC.aa.C36.1 |
| 0.970869338 | 3.267572699 | PC.ae.C34.2 (interacts with) PC.ae.C34.3 |
| 0.970600532 | 7.042236184 | PC.aa.C38.0 (interacts with) PC.ae.C38.6 |
| 0.970455685 | 3.60855037 | PC.ae.C40.2 (interacts with) SM.OH.C22.2 |
| 0.970241337 | 2 | C12.1 (interacts with) C14.2 |
| 0.96860051 | 22.04778634 | PC.aa.C36.5 (interacts with) PC.aa.C38.5 |
| 0.966575537 | 4.61688837 | PC.ae.C36.5 (interacts with) PC.ae.C38.5 |
| 0.965626926 | 3.942099196 | SM.C16.0 (interacts with) SM.C16.1 |
| 0.964329319 | 5.575651457 | PC.aa.C38.6 (interacts with) PC.aa.C40.6 |
| 0.963989468 | 8.10236641 | PC.ae.C34.0 (interacts with) PC.ae.C36.1 |
| 0.962654626 | 4.11822356 | PC.aa.C42.0 (interacts with) PC.ae.C42.5 |
| 0.961648606 | 4.506600212 | PC.aa.C32.1 (interacts with) PC.aa.C34.1 |
| 0.960257628 | 5.028586281 | PC.ae.C36.1 (interacts with) PC.ae.C38.3 |
| 0.959948762 | 8.277717366 | PC.aa.C38.5 (interacts with) PC.aa.C42.6 |
| 0.959877959 | 6.941840262 | SM.OH.C16.1 (interacts with) SM.OH.C22.2 |
| 0.956761252 | 4.787991535 | PC.ae.C38.3 (interacts with) PC.ae.C38.4 |
| 0.956500165 | 6.200448745 | SM.C18.0 (interacts with) SM.C18.1 |
| 0.955910588 | 2.906097712 | SM.OH.C14.1 (interacts with) SM.C16.0 |
| 0.955028481 | 4.138095238 | PC.ae.C40.3 (interacts with) PC.ae.C40.4 |
| 0.954647768 | 5.49933122 | PC.aa.C28.1 (interacts with) SM.OH.C14.1 |
| 0.953990636 | 4.348279767 | SM.OH.C22.1 (interacts with) SM.OH.C24.1 |
| 0.952925203 | 4.493243535 | PC.ae.C38.4 (interacts with) PC.ae.C38.5 |
| 0.952800504 | 16.89481668 | PC.aa.C30.0 (interacts with) PC.aa.C32.0 |
| 0.95224192 | 2.852526942 | PC.ae.C40.2 (interacts with) PC.ae.C40.6 |
| 0.950743138 | 18.79414308 | PC.ae.C44.5 (interacts with) PC.ae.C44.6 |
| 0.95061569 | 5.820159991 | PC.ae.C38.6 (interacts with) PC.ae.C40.6 |
| 0.949928697 | 4.140358451 | PC.aa.C34.3 (interacts with) PC.aa.C36.3 |
| 0.948681763 | 5.255233583 | PC.aa.C32.0 (interacts with) PC.ae.C34.1 |
| 0.948511468 | 4.278960426 | SM.OH.C16.1 (interacts with) SM.C16.0 |
| 0.948444212 | 3.431006859 | SM.OH.C14.1 (interacts with) SM.C16.1 |
| 0.948433845 | 12.20808004 | PC.aa.C36.4 (interacts with) PC.aa.C38.4 |
| 0.947909912 | 5.05645333 | PC.aa.C32.2 (interacts with) PC.aa.C34.3 |
| 0.94724942 | 4.705957588 | PC.aa.C40.4 (interacts with) PC.aa.C40.5 |
| 0.946941166 | 8.803685775 | SM.OH.C22.1 (interacts with) SM.OH.C22.2 |
| 0.94674014 | 4.948660098 | PC.ae.C30.0 (interacts with) PC.ae.C34.1 |
| 0.94663347 | 5.289735088 | SM.OH.C22.1 (interacts with) SM.C24.0 |
| 0.946011799 | 5.530752112 | PC.ae.C40.4 (interacts with) PC.ae.C40.5 |
| 0.945727353 | 4.250733381 | SM.OH.C16.1 (interacts with) SM.C16.1 |
| 0.945466021 | 2 | C2 (interacts with) C14.1 |
| 0.945393851 | 5.801965573 | PC.aa.C38.3 (interacts with) PC.aa.C40.4 |
| 0.945365186 | 4.045534911 | PC.ae.C34.2 (interacts with) PC.ae.C36.4 |
| 0.944524494 | 3.94331877 | PC.ae.C40.6 (interacts with) SM.OH.C22.2 |
| 0.94449471 | 7.117035065 | PC.ae.C40.2 (interacts with) SM.C24.1 |
| 0.944213081 | 3.971730564 | PC.ae.C38.6 (interacts with) PC.ae.C40.5 |
| 0.943828067 | 4.723463643 | PC.aa.C38.6 (interacts with) PC.ae.C38.0 |
| 0.943810699 | 4.191394652 | PC.ae.C36.2 (interacts with) PC.ae.C38.3 |
| 0.943449772 | 5.857443205 | PC.aa.C38.3 (interacts with) PC.aa.C40.5 |
| 0.943433941 | 3.91817165 | PC.aa.C36.1 (interacts with) PC.aa.C40.5 |
| 0.943365905 | 8.297147665 | PC.aa.C36.5 (interacts with) PC.aa.C42.6 |
| 0.94330167 | 6.4820572 | PC.aa.C32.2 (interacts with) PC.aa.C36.3 |
| 0.942961844 | 4.442279602 | SM.OH.C16.1 (interacts with) SM.OH.C22.1 |
| 0.942812481 | 8.096011963 | PC.aa.C28.1 (interacts with) PC.ae.C36.1 |
| 0.942153519 | 6.278395407 | lysoPC.a.C16.1 (interacts with) lysoPC.a.C18.1 |
| 0.941722572 | 2 | Leu (interacts with) Val |
| 0.940078551 | 5.762853649 | PC.aa.C36.3 (interacts with) PC.aa.C40.4 |
| 0.938939161 | 8.238446738 | PC.aa.C38.6 (interacts with) PC.ae.C40.6 |
| 0.938541532 | 16.58892623 | lysoPC.a.C17.0 (interacts with) lysoPC.a.C18.0 |
| 0.938310978 | 8.075989728 | PC.ae.C40.5 (interacts with) PC.ae.C42.3 |
| 0.938239638 | 4.916403927 | PC.aa.C36.6 (interacts with) PC.ae.C38.0 |
| 0.938151434 | 3.344269077 | PC.aa.C36.1 (interacts with) PC.aa.C40.4 |
| 0.937840842 | 12.33965039 | lysoPC.a.C16.1 (interacts with) lysoPC.a.C20.3 |
| 0.937826938 | 2.965698274 | PC.aa.C38.0 (interacts with) PC.ae.C40.2 |
| 0.936894972 | 2.341836735 | PC.aa.C36.0 (interacts with) PC.aa.C38.1 |
| 0.936639259 | 8.175276147 | PC.ae.C36.4 (interacts with) PC.ae.C38.4 |
| 0.936597059 | 3.925795345 | PC.aa.C42.5 (interacts with) PC.aa.C42.6 |
| 0.935674094 | 5.870543952 | PC.aa.C28.1 (interacts with) SM.C16.1 |
| 0.935530542 | 5.135890169 | PC.aa.C32.3 (interacts with) PC.aa.C36.2 |
| 0.935263795 | 9.865511481 | PC.ae.C40.5 (interacts with) PC.ae.C40.6 |
| 0.934875528 | 3.688379879 | PC.ae.C34.0 (interacts with) PC.ae.C42.3 |
| 0.934375622 | 8.73638566 | SM.OH.C16.1 (interacts with) SM.C18.0 |
| 0.934122376 | 3.973978691 | PC.ae.C32.2 (interacts with) PC.ae.C40.3 |
| 0.934010528 | 3.560273081 | PC.aa.C30.0 (interacts with) PC.aa.C32.2 |
| 0.933616248 | 8.221245573 | PC.ae.C40.2 (interacts with) SM.OH.C16.1 |
| 0.932584323 | 5.029181984 | lysoPC.a.C18.1 (interacts with) lysoPC.a.C20.3 |
| 0.932541865 | 5.078615493 | SM.OH.C22.1 (interacts with) SM.C16.1 |
| 0.931886971 | 5.666491599 | PC.aa.C28.1 (interacts with) SM.C16.0 |
| 0.931831163 | 8.312234309 | PC.ae.C40.4 (interacts with) PC.ae.C42.3 |
| 0.931174593 | 5.014858785 | PC.aa.C38.0 (interacts with) SM.OH.C22.2 |
| 0.930952645 | 6.205844802 | PC.aa.C32.2 (interacts with) PC.aa.C34.4 |
| 0.930474544 | 2 | C2 (interacts with) C12.1 |
| 0.930227228 | 21.18325758 | SM.C16.1 (interacts with) SM.C18.1 |
| 0.930170645 | 5.162580605 | PC.aa.C28.1 (interacts with) PC.ae.C38.3 |
| 0.930050081 | 15.74347645 | PC.ae.C40.3 (interacts with) PC.ae.C42.5 |
| 0.930023729 | 6.970789617 | PC.aa.C36.1 (interacts with) PC.aa.C36.3 |
| 0.929378004 | 9.85412487 | PC.ae.C34.1 (interacts with) PC.ae.C36.1 |
| 0.929220753 | 2.588546334 | SM.OH.C24.1 (interacts with) SM.C16.1 |
| 0.928925322 | 6.071907655 | PC.ae.C42.2 (interacts with) PC.ae.C42.3 |
| 0.928779306 | 10.20904034 | PC.ae.C36.4 (interacts with) PC.ae.C38.5 |
| 0.927963741 | 6.559738747 | PC.aa.C40.6 (interacts with) PC.ae.C38.0 |
| 0.927660611 | 4.834724765 | PC.ae.C38.5 (interacts with) SM.C16.0 |
| 0.927457929 | 11.22559447 | PC.ae.C38.0 (interacts with) PC.ae.C40.6 |
| 0.92682232 | 12.82933321 | lysoPC.a.C16.0 (interacts with) PC.aa.C36.3 |
| 0.926501632 | 6.151268306 | PC.aa.C34.2 (interacts with) PC.ae.C34.3 |
| 0.926264207 | 3.414759998 | PC.ae.C34.0 (interacts with) PC.ae.C42.2 |
| 0.926098299 | 6.743646612 | PC.ae.C36.2 (interacts with) SM.OH.C14.1 |
| 0.925899624 | 3.580816298 | PC.ae.C32.2 (interacts with) PC.ae.C40.4 |
| 0.925817801 | 21.50542348 | PC.ae.C36.0 (interacts with) PC.ae.C40.5 |
| 0.925687041 | 7.406540995 | PC.ae.C30.0 (interacts with) PC.ae.C36.1 |
| 0.925683482 | 3.872260017 | PC.aa.C42.5 (interacts with) SM.C24.0 |
| 0.925385006 | 14.94568994 | PC.ae.C34.1 (interacts with) PC.ae.C38.3 |
| 0.925304197 | 5.924009184 | PC.ae.C34.0 (interacts with) PC.ae.C38.3 |
| 0.92489636 | 6.977797067 | PC.aa.C34.3 (interacts with) PC.aa.C34.4 |
| 0.924489364 | 4.8871985 | PC.ae.C38.0 (interacts with) SM.OH.C22.1 |
| 0.924225262 | 5.3056803 | PC.ae.C42.2 (interacts with) SM.OH.C22.1 |
| 0.923583788 | 5.485142157 | PC.ae.C36.4 (interacts with) PC.ae.C36.5 |
| 0.923503271 | 3.870135702 | SM.OH.C16.1 (interacts with) SM.OH.C24.1 |
| 0.923380635 | 11.67989776 | PC.aa.C38.0 (interacts with) PC.ae.C40.5 |
| 0.923000508 | 9.822463016 | PC.ae.C38.5 (interacts with) PC.ae.C38.6 |
| 0.922544006 | 6.786968606 | PC.aa.C36.2 (interacts with) PC.ae.C34.3 |
| 0.922462311 | 7.654468671 | PC.ae.C36.3 (interacts with) PC.ae.C36.4 |
| 0.921921963 | 8.014030542 | PC.aa.C28.1 (interacts with) SM.OH.C22.1 |
| 0.921840674 | 4.887671398 | PC.ae.C34.3 (interacts with) PC.ae.C36.4 |
| 0.921815098 | 8.362797858 | PC.ae.C38.3 (interacts with) PC.ae.C42.3 |
| 0.921711355 | 2.507936508 | PC.ae.C32.2 (interacts with) PC.ae.C38.5 |
| 0.921707824 | 4.019355659 | lysoPC.a.C20.3 (interacts with) PC.aa.C34.1 |
| 0.921560122 | 7.868868812 | PC.aa.C28.1 (interacts with) SM.OH.C16.1 |
| 0.92129979 | 6.128655764 | PC.ae.C38.3 (interacts with) PC.ae.C42.2 |
| 0.921148318 | 4.617745808 | PC.aa.C42.6 (interacts with) PC.ae.C38.0 |
| 0.920748445 | 16.39273507 | SM.OH.C16.1 (interacts with) SM.C18.1 |
| 0.920641737 | 13.84046093 | PC.aa.C36.0 (interacts with) PC.aa.C38.0 |
| 0.920600761 | 5.438860948 | SM.OH.C14.1 (interacts with) SM.OH.C22.1 |
| 0.920217632 | 22.81230528 | PC.ae.C42.5 (interacts with) PC.ae.C44.6 |
| 0.919830016 | 6.834277436 | PC.ae.C38.6 (interacts with) SM.OH.C22.2 |
| 0.919796894 | 9.55011928 | SM.OH.C22.2 (interacts with) SM.C18.0 |
| 0.91975751 | 4.64207709 | PC.ae.C32.2 (interacts with) SM.C16.0 |
| 0.919729943 | 7.59172487 | PC.aa.C38.6 (interacts with) SM.OH.C22.2 |
| 0.919688354 | 3.714260695 | PC.aa.C34.3 (interacts with) PC.aa.C36.1 |
| 0.919580241 | 11.29606117 | PC.ae.C40.2 (interacts with) SM.OH.C22.1 |
| 0.919546592 | 12.01901971 | PC.aa.C34.1 (interacts with) PC.aa.C40.4 |
| 0.919194013 | 4.959305276 | PC.aa.C38.6 (interacts with) SM.OH.C22.1 |
| 0.918887319 | 6.613402957 | PC.ae.C32.1 (interacts with) PC.ae.C42.4 |
| 0.918776642 | 22.94823835 | PC.ae.C40.4 (interacts with) PC.ae.C42.5 |
| 0.918597421 | 13.44069848 | SM.OH.C22.2 (interacts with) SM.C16.1 |
| 0.918594888 | 4.14412651 | PC.ae.C38.5 (interacts with) PC.ae.C40.4 |
| 0.918308099 | 5.106228463 | PC.ae.C38.4 (interacts with) SM.C16.1 |
| 0.918171832 | 18.63891066 | PC.aa.C34.1 (interacts with) PC.aa.C36.3 |
| 0.917938393 | 5.142613201 | PC.ae.C32.1 (interacts with) PC.ae.C32.2 |
| 0.917875959 | 6.308514914 | PC.ae.C40.3 (interacts with) SM.C16.0 |
| 0.917646027 | 4.83269579 | SM.OH.C24.1 (interacts with) SM.C16.0 |
| 0.917573844 | 5.926896156 | PC.ae.C38.4 (interacts with) SM.OH.C24.1 |
| 0.917445508 | 3.712468672 | PC.aa.C42.6 (interacts with) SM.C24.0 |
| 0.917154094 | 2 | C2 (interacts with) C14.2 |
| 0.91679238 | 4.731711285 | PC.aa.C42.5 (interacts with) SM.OH.C22.1 |
| 0.916770054 | 6.880926726 | PC.aa.C42.6 (interacts with) PC.ae.C34.0 |
| 0.916707113 | 5.358420647 | PC.ae.C30.0 (interacts with) PC.ae.C32.1 |
| 0.916675221 | 4.486290947 | PC.ae.C38.5 (interacts with) SM.C16.1 |
| 0.916575433 | 4.053341383 | PC.aa.C28.1 (interacts with) PC.ae.C36.2 |
| 0.916506812 | 6.622283081 | PC.ae.C38.6 (interacts with) PC.ae.C40.2 |
| 0.916090341 | 10.68158147 | PC.aa.C36.5 (interacts with) PC.aa.C42.5 |
| 0.916077262 | 7.792155528 | PC.ae.C32.1 (interacts with) SM.C16.0 |
| 0.91531006 | 4.402971674 | PC.ae.C42.2 (interacts with) SM.C24.0 |
| 0.915179524 | 7.092203203 | PC.aa.C36.6 (interacts with) PC.aa.C42.6 |
| 0.915084921 | 5.403921021 | PC.ae.C38.4 (interacts with) SM.C16.0 |
| 0.915042525 | 4.579552412 | PC.ae.C36.2 (interacts with) PC.ae.C38.4 |
| 0.914817511 | 11.92776188 | PC.ae.C40.1 (interacts with) PC.ae.C42.3 |
| 0.914737036 | 6.671240836 | PC.ae.C36.1 (interacts with) PC.ae.C36.2 |
| 0.914537847 | 8.887720457 | PC.aa.C36.1 (interacts with) PC.aa.C38.3 |
| 0.914235426 | 6.617499703 | SM.OH.C22.1 (interacts with) SM.C16.0 |
| 0.914022576 | 14.85106645 | PC.ae.C32.1 (interacts with) PC.ae.C34.1 |
| 0.913978347 | 10.66611699 | SM.OH.C22.2 (interacts with) SM.C24.1 |
| 0.913140105 | 5.391301047 | lysoPC.a.C16.0 (interacts with) PC.aa.C36.1 |
| 0.912979202 | 11.68843311 | SM.OH.C14.1 (interacts with) SM.OH.C22.2 |
| 0.912570493 | 5.271947329 | PC.aa.C28.1 (interacts with) PC.ae.C34.0 |
| 0.912015419 | 15.83914586 | PC.aa.C32.3 (interacts with) PC.aa.C34.4 |
| 0.911407713 | 9.129844574 | PC.ae.C34.3 (interacts with) PC.ae.C36.2 |
| 0.91138593 | 5.036769661 | PC.aa.C34.2 (interacts with) PC.ae.C36.3 |
| 0.910839075 | 4.736334471 | PC.aa.C30.0 (interacts with) PC.aa.C34.4 |
| 0.910627492 | 27.38831119 | PC.aa.C30.0 (interacts with) PC.ae.C34.1 |
| 0.910612003 | 4.792927994 | PC.aa.C42.5 (interacts with) PC.ae.C42.2 |
| 0.909729131 | 15.6795014 | PC.aa.C34.1 (interacts with) PC.aa.C40.5 |
| 0.909513861 | 9.980397148 | PC.aa.C38.0 (interacts with) PC.aa.C38.6 |
| 0.909305978 | 16.67019637 | PC.aa.C36.0 (interacts with) PC.ae.C40.6 |
| 0.908756497 | 5.251438796 | PC.ae.C34.0 (interacts with) SM.OH.C22.1 |
| 0.908534047 | 7.410162376 | PC.ae.C38.3 (interacts with) PC.ae.C38.5 |
| 0.907597615 | 23.74629985 | PC.aa.C32.2 (interacts with) PC.aa.C32.3 |
| 0.907391606 | 10.14929607 | PC.ae.C32.2 (interacts with) PC.ae.C38.6 |
| 0.907352314 | 5.203593575 | PC.aa.C38.0 (interacts with) SM.C24.1 |
| 0.90732308 | 4.110116699 | SM.OH.C14.1 (interacts with) SM.OH.C24.1 |
| 0.907155469 | 2 | C10.1 (interacts with) C12.1 |
| 0.906660708 | 10.44374972 | PC.aa.C32.0 (interacts with) PC.ae.C30.0 |
| 0.906572335 | 7.449677992 | PC.aa.C32.3 (interacts with) PC.aa.C34.2 |
| 0.906036362 | 10.75914217 | PC.ae.C38.0 (interacts with) SM.OH.C22.2 |
| 0.905921543 | 7.389946992 | PC.ae.C38.3 (interacts with) SM.C16.1 |
| 0.905633359 | 7.949191656 | PC.aa.C34.4 (interacts with) PC.aa.C40.4 |
| 0.905527409 | 10.64531096 | PC.aa.C32.3 (interacts with) PC.ae.C36.1 |
| 0.90543026 | 14.13187003 | PC.aa.C32.1 (interacts with) PC.aa.C36.1 |
| 0.905034521 | 5.742232603 | PC.aa.C36.2 (interacts with) PC.ae.C36.3 |
| 0.904753792 | 6.788403451 | PC.ae.C32.1 (interacts with) PC.ae.C38.3 |
| 0.90457353 | 9.816641492 | PC.aa.C28.1 (interacts with) PC.ae.C30.0 |
| 0.904448647 | 5.956569956 | PC.ae.C36.5 (interacts with) PC.ae.C38.4 |
| 0.904232044 | 5.798614593 | PC.aa.C36.5 (interacts with) PC.aa.C36.6 |
| 0.904130556 | 7.210621901 | PC.ae.C32.1 (interacts with) PC.ae.C34.2 |
| 0.904099595 | 9.051288968 | PC.ae.C36.1 (interacts with) PC.ae.C42.2 |
| 0.90400337 | 7.540278143 | PC.aa.C42.6 (interacts with) PC.ae.C42.2 |
| 0.903939305 | 11.17846365 | PC.aa.C34.4 (interacts with) PC.aa.C36.3 |
| 0.903088013 | 14.56910995 | PC.ae.C38.0 (interacts with) PC.ae.C40.1 |
| 0.902888108 | 5.063713916 | PC.ae.C38.6 (interacts with) SM.OH.C22.1 |
| 0.902842259 | 12.71483969 | PC.ae.C36.1 (interacts with) PC.ae.C42.3 |
| 0.902771933 | 3.487617802 | PC.aa.C34.4 (interacts with) PC.aa.C38.3 |
| 0.902633254 | 5.142299467 | PC.ae.C38.0 (interacts with) PC.ae.C38.6 |
| 0.902436006 | 8.443022325 | PC.aa.C38.6 (interacts with) PC.ae.C40.2 |
| 0.902395717 | 8.261572627 | PC.aa.C40.6 (interacts with) PC.aa.C42.6 |
| 0.902139909 | 9.008487313 | PC.ae.C38.3 (interacts with) SM.OH.C14.1 |
| 0.901946823 | 11.8692511 | PC.ae.C38.3 (interacts with) SM.OH.C22.1 |
| 0.901844825 | 8.668816437 | PC.ae.C30.0 (interacts with) PC.ae.C38.3 |
| 0.901750406 | 19.08722717 | PC.aa.C36.2 (interacts with) PC.ae.C36.2 |
| 0.901650334 | 6.677013351 | PC.ae.C40.6 (interacts with) SM.C24.1 |
| 0.901395796 | 5.19664941 | PC.ae.C32.1 (interacts with) PC.ae.C36.4 |
| 0.901317512 | 6.89379459 | PC.ae.C42.3 (interacts with) SM.OH.C22.1 |
| 0.901263686 | 7.417704705 | lysoPC.a.C16.0 (interacts with) lysoPC.a.C20.3 |
| 0.900845933 | 6.19748121 | PC.ae.C42.2 (interacts with) SM.OH.C24.1 |
| 0.900679113 | 9.49143044 | lysoPC.a.C16.0 (interacts with) PC.aa.C40.4 |
| 0.900503267 | 7.268428793 | lysoPC.a.C17.0 (interacts with) PC.ae.C36.1 |
| 0.900490487 | 5.390150493 | PC.ae.C38.4 (interacts with) SM.OH.C14.1 |
| 0.90048865 | 8.255141416 | PC.ae.C38.3 (interacts with) SM.OH.C24.1 |
| 0.900394788 | 4.749144215 | PC.ae.C32.1 (interacts with) PC.ae.C38.4 |
| 0.900182805 | 12.56187261 | lysoPC.a.C16.1 (interacts with) PC.aa.C32.1 |
| 0.899629316 | 8.865320668 | lysoPC.a.C16.0 (interacts with) PC.aa.C34.3 |
| 0.899575607 | 8.22732896 | PC.ae.C38.3 (interacts with) SM.C16.0 |
| 0.89907341 | 4.372845802 | PC.aa.C42.5 (interacts with) PC.ae.C34.0 |
| 0.898826198 | 21.69022909 | PC.aa.C28.1 (interacts with) PC.ae.C34.1 |
| 0.898357395 | 10.02671948 | PC.aa.C32.3 (interacts with) PC.ae.C34.3 |
| 0.898244862 | 15.32275079 | PC.ae.C30.0 (interacts with) PC.ae.C34.0 |
| 0.898221155 | 2.222222222 | lysoPC.a.C20.3 (interacts with) PC.aa.C32.1 |
| 0.898134058 | 5.222272499 | PC.aa.C38.6 (interacts with) PC.ae.C38.6 |
| 0.897638251 | 27.96664991 | SM.OH.C22.2 (interacts with) SM.C18.1 |
| 0.897543718 | 9.010632919 | PC.ae.C32.2 (interacts with) PC.ae.C42.3 |
| 0.897527255 | 7.734108582 | PC.aa.C42.6 (interacts with) PC.ae.C40.1 |
| 0.897232977 | 12.83400982 | PC.aa.C38.5 (interacts with) PC.aa.C42.5 |
| 0.897019772 | 13.79274727 | PC.aa.C42.0 (interacts with) PC.ae.C32.1 |
| 0.896588778 | 13.47722883 | PC.ae.C36.2 (interacts with) PC.ae.C36.3 |
| 0.896358295 | 16.02940732 | PC.aa.C42.6 (interacts with) PC.ae.C36.1 |
| 0.896209498 | 17.17917231 | SM.OH.C22.2 (interacts with) SM.C16.0 |
| 0.896144198 | 8.941915144 | PC.aa.C38.5 (interacts with) SM.C24.0 |
| 0.895707343 | 5.786766766 | PC.ae.C32.1 (interacts with) PC.ae.C38.5 |
| 0.895680558 | 6.986247293 | PC.ae.C38.4 (interacts with) PC.ae.C40.4 |
| 0.895481369 | 14.27146021 | SM.C16.1 (interacts with) SM.C18.0 |
| 0.895408017 | 6.323962763 | PC.aa.C28.1 (interacts with) SM.OH.C24.1 |
| 0.894831632 | 13.01942512 | PC.ae.C36.5 (interacts with) PC.ae.C38.6 |
| 0.894319163 | 7.713019856 | PC.ae.C36.2 (interacts with) PC.ae.C42.3 |
| 0.894105525 | 252 | Ile (interacts with) Leu |
| 0.894035885 | 8.274563255 | PC.ae.C32.2 (interacts with) PC.ae.C40.5 |
| 0.89398906 | 7.129011272 | SM.OH.C24.1 (interacts with) SM.C24.0 |
| 0.893825963 | 20.93794089 | PC.aa.C36.0 (interacts with) PC.ae.C38.6 |
| 0.89366521 | 18.63736442 | PC.ae.C36.3 (interacts with) PC.ae.C38.3 |
| 0.893616126 | 21.9452184 | SM.C16.0 (interacts with) SM.C18.0 |
| 0.893601956 | 6.152026743 | PC.ae.C34.0 (interacts with) PC.ae.C36.2 |
| 0.892896485 | 9.715708173 | PC.aa.C34.2 (interacts with) PC.ae.C34.2 |
| 0.892716185 | 10.19316641 | lysoPC.a.C17.0 (interacts with) PC.ae.C38.3 |
| 0.892647806 | 8.412174141 | PC.ae.C34.0 (interacts with) PC.ae.C40.5 |
| 0.892597874 | 22.49588995 | PC.ae.C36.1 (interacts with) SM.OH.C22.1 |
| 0.892470041 | 20.55920308 | PC.ae.C40.3 (interacts with) PC.ae.C42.3 |
| 0.892374965 | 9.020201617 | PC.ae.C40.2 (interacts with) SM.C18.0 |
| 0.892173347 | 4.294974989 | PC.ae.C32.2 (interacts with) PC.ae.C36.5 |
| 0.892054395 | 4.862270158 | PC.ae.C38.5 (interacts with) PC.ae.C40.3 |
| 0.892037126 | 4.913935586 | PC.ae.C38.5 (interacts with) SM.OH.C24.1 |
| 0.892034401 | 3.977533578 | lysoPC.a.C17.0 (interacts with) PC.ae.C40.1 |
| 0.892013861 | 7.143087569 | PC.ae.C36.2 (interacts with) SM.OH.C24.1 |
| 0.892007726 | 5.816407006 | PC.aa.C28.1 (interacts with) PC.ae.C42.2 |
| 0.891973963 | 10.64920841 | PC.ae.C40.2 (interacts with) PC.ae.C40.5 |
| 0.891969657 | 6.381119586 | PC.ae.C42.2 (interacts with) SM.C16.0 |
| 0.891890092 | 4 | C14 (interacts with) C18.1 |
| 0.891731654 | 5.606603691 | PC.ae.C42.2 (interacts with) SM.C16.1 |
| 0.891493566 | 3.823325084 | lysoPC.a.C16.0 (interacts with) PC.aa.C34.1 |
| 0.891058533 | 10.25602734 | PC.ae.C34.0 (interacts with) PC.ae.C40.1 |
| 0.890970022 | 7.614902809 | PC.ae.C38.5 (interacts with) PC.ae.C42.2 |
| 0.89088662 | 7.2809748 | PC.aa.C42.0 (interacts with) PC.ae.C40.3 |
| 0.89071399 | 10.94403512 | PC.ae.C40.3 (interacts with) PC.ae.C40.5 |
| 0.890445433 | 11.62717739 | PC.ae.C40.6 (interacts with) SM.OH.C22.1 |
| 0.890353028 | 14.01969073 | PC.aa.C38.0 (interacts with) PC.ae.C38.0 |
| 0.890187244 | 5.333333333 | lysoPC.a.C18.1 (interacts with) lysoPC.a.C18.2 |
| 0.890154203 | 8.994169189 | PC.aa.C32.3 (interacts with) PC.ae.C36.3 |
| 0.890056641 | 32.49186051 | PC.aa.C42.0 (interacts with) PC.ae.C44.6 |
| 0.889651926 | 2 | C10.1 (interacts with) C14.2 |
| 0.889546387 | 4.713460854 | PC.ae.C34.0 (interacts with) SM.C24.0 |
| 0.889440458 | 55.01212408 | PC.aa.C32.3 (interacts with) PC.aa.C34.3 |
| 0.888874577 | 17.49452561 | PC.ae.C36.1 (interacts with) SM.OH.C14.1 |
| 0.888398269 | 11.31053342 | SM.OH.C22.2 (interacts with) SM.OH.C24.1 |
| 0.888377007 | 7.734716609 | PC.ae.C38.0 (interacts with) PC.ae.C42.3 |
| 0.888357745 | 7.790970551 | PC.ae.C38.4 (interacts with) PC.ae.C42.2 |
| 0.888295381 | 13.29286764 | lysoPC.a.C20.3 (interacts with) PC.aa.C36.1 |
| 0.888110708 | 19.33066119 | PC.aa.C38.5 (interacts with) PC.ae.C42.2 |
| 0.888081049 | 10.45103974 | PC.ae.C36.2 (interacts with) SM.OH.C22.1 |
| 0.88785028 | 7.319057561 | PC.aa.C28.1 (interacts with) SM.C24.0 |
| 0.887750142 | 3.954615936 | PC.ae.C32.2 (interacts with) SM.OH.C14.1 |
| 0.887647537 | 7.601626104 | PC.aa.C42.6 (interacts with) SM.OH.C22.1 |
| 0.887419036 | 154.2029003 | lysoPC.a.C16.0 (interacts with) lysoPC.a.C18.0 |
| 0.887386509 | 17.31317776 | PC.aa.C42.0 (interacts with) SM.C16.0 |
| 0.887375126 | 10.75834466 | PC.ae.C34.2 (interacts with) PC.ae.C42.4 |
| 0.887199569 | 8.298603236 | PC.aa.C32.3 (interacts with) PC.ae.C30.0 |
| 0.88714357 | 4.363983328 | PC.aa.C36.6 (interacts with) PC.aa.C40.6 |
| 0.886856856 | 8 | Cit (interacts with) total.DMA |
| 0.8866775 | 11.11743802 | lysoPC.a.C16.0 (interacts with) PC.aa.C40.5 |
| 0.88637591 | 9.315819974 | PC.ae.C36.2 (interacts with) SM.OH.C16.1 |
| 0.886144217 | 8.871916888 | PC.aa.C42.6 (interacts with) PC.ae.C42.3 |
| 0.885997403 | 10.60881473 | PC.ae.C36.1 (interacts with) PC.ae.C38.4 |
| 0.885927883 | 4.990386942 | PC.ae.C40.4 (interacts with) SM.C16.0 |
| 0.885704389 | 5.129649042 | PC.ae.C32.2 (interacts with) SM.C16.1 |
| 0.885513272 | 10.40724862 | PC.ae.C38.6 (interacts with) PC.ae.C42.3 |
| 0.885403787 | 14.02202826 | PC.ae.C34.3 (interacts with) PC.ae.C38.3 |
| 0.88536082 | 8.696879227 | PC.ae.C36.1 (interacts with) PC.ae.C40.1 |
| 0.885152314 | 38.66285798 | PC.aa.C36.6 (interacts with) PC.aa.C38.5 |
| 0.884755838 | 252 | Ile (interacts with) Val |
| 0.884517818 | 5.785726311 | PC.ae.C34.0 (interacts with) SM.OH.C14.1 |
| 0.884450871 | 42.38129175 | PC.aa.C30.0 (interacts with) PC.ae.C30.0 |
| 0.884196946 | 5.370583442 | PC.aa.C28.1 (interacts with) PC.ae.C38.4 |
| 0.884087207 | 7.537040287 | SM.C18.0 (interacts with) SM.C24.1 |
| 0.883925294 | 10.08398291 | lysoPC.a.C18.0 (interacts with) PC.aa.C36.2 |
| 0.883853135 | 5.892625628 | PC.aa.C32.3 (interacts with) PC.ae.C34.1 |
| 0.883734017 | 16.7118784 | PC.aa.C32.3 (interacts with) PC.ae.C36.2 |
| 0.883729251 | 5.618982563 | PC.ae.C34.1 (interacts with) PC.ae.C36.3 |
| 0.883673882 | 10.75359169 | PC.ae.C38.3 (interacts with) PC.ae.C40.4 |
| 0.883355824 | 14.14072191 | SM.OH.C22.1 (interacts with) SM.C18.0 |
| 0.883321031 | 32.49007718 | PC.aa.C32.0 (interacts with) PC.ae.C32.1 |
| 0.883259166 | 13.63806777 | SM.OH.C14.1 (interacts with) SM.C18.0 |
| 0.882933555 | 20.56842455 | PC.aa.C30.0 (interacts with) PC.aa.C32.3 |
| 0.882874627 | 8.515858779 | PC.ae.C38.0 (interacts with) PC.ae.C40.5 |
| 0.882636651 | 5.725582538 | PC.ae.C40.3 (interacts with) SM.OH.C14.1 |
| 0.882564902 | 14.32136632 | PC.ae.C34.3 (interacts with) PC.ae.C38.4 |
| 0.88252168 | 9.694713553 | PC.aa.C32.2 (interacts with) PC.aa.C36.1 |
| 0.881807461 | 9.193235983 | PC.ae.C38.5 (interacts with) PC.ae.C42.3 |
| 0.881720537 | 16.92568161 | PC.aa.C32.0 (interacts with) PC.ae.C36.1 |
| 0.881646465 | 18.22355013 | PC.aa.C32.3 (interacts with) PC.ae.C38.3 |
| 0.88154114 | 7.840138193 | PC.ae.C32.2 (interacts with) PC.ae.C38.3 |
| 0.881210887 | 10.28151081 | PC.ae.C38.6 (interacts with) SM.C16.0 |
| 0.880831119 | 8.167320546 | PC.ae.C38.5 (interacts with) PC.ae.C40.5 |
| 0.880632805 | 52.79084388 | lysoPC.a.C20.3 (interacts with) PC.aa.C36.3 |
| 0.880545023 | 5.923037764 | PC.aa.C30.0 (interacts with) PC.aa.C40.4 |
| 0.880544894 | 7.245983435 | PC.ae.C40.3 (interacts with) SM.OH.C16.1 |
| 0.88049741 | 9.234431942 | PC.ae.C40.6 (interacts with) SM.OH.C16.1 |
| 0.880459813 | 6.68029427 | PC.ae.C36.2 (interacts with) SM.C16.1 |
| 0.880345696 | 28.72789277 | PC.aa.C38.5 (interacts with) PC.aa.C40.6 |
| 0.880156052 | 39.88262178 | PC.ae.C34.1 (interacts with) PC.ae.C42.4 |
| 0.880108514 | 10.26786159 | PC.ae.C36.1 (interacts with) SM.C24.0 |
| 0.880090683 | 8.197197981 | PC.ae.C38.4 (interacts with) SM.OH.C16.1 |
| 0.879976083 | 10.93782601 | PC.ae.C40.5 (interacts with) PC.ae.C42.2 |
| 0.879857315 | 10.76890542 | PC.ae.C34.2 (interacts with) PC.ae.C36.2 |
| 0.879749812 | 3.66677322 | PC.aa.C34.3 (interacts with) PC.aa.C40.4 |
| 0.879415752 | 9.494801474 | lysoPC.a.C17.0 (interacts with) PC.ae.C34.0 |
| 0.87924121 | 30.95623691 | lysoPC.a.C16.0 (interacts with) PC.aa.C34.4 |
| 0.879201228 | 12.77728043 | PC.ae.C38.6 (interacts with) PC.ae.C42.2 |
| 0.878717259 | 33.39247765 | SM.C16.0 (interacts with) SM.C18.1 |
| 0.878622861 | 10.23482902 | PC.aa.C36.3 (interacts with) PC.aa.C38.3 |
| 0.878552499 | 8.704280426 | lysoPC.a.C18.0 (interacts with) PC.aa.C38.3 |
| 0.878402148 | 10.99542994 | PC.ae.C32.1 (interacts with) PC.ae.C36.3 |
| 0.878355134 | 21.92901108 | SM.OH.C14.1 (interacts with) SM.C18.1 |
| 0.87821572 | 9.080454893 | PC.aa.C36.5 (interacts with) PC.ae.C38.0 |
| 0.878134419 | 11.71387092 | PC.aa.C34.4 (interacts with) PC.aa.C36.1 |
| 0.87788154 | 5.354411522 | PC.ae.C30.0 (interacts with) PC.ae.C36.3 |
| 0.877849925 | 12.29502238 | PC.ae.C36.4 (interacts with) PC.ae.C38.3 |
| 0.877842312 | 4.591099799 | PC.ae.C32.1 (interacts with) PC.ae.C36.5 |
| 0.877828937 | 15.89325981 | PC.ae.C38.3 (interacts with) PC.ae.C40.3 |
| 0.877800877 | 7.929995809 | PC.ae.C32.1 (interacts with) PC.ae.C40.4 |
| 0.877634843 | 4.128217648 | PC.ae.C38.5 (interacts with) SM.OH.C14.1 |
| 0.877551169 | 13.79138774 | PC.aa.C42.5 (interacts with) PC.ae.C36.1 |
| 0.877532538 | 2 | C2 (interacts with) C10.1 |
| 0.877361821 | 8.402924739 | PC.ae.C38.6 (interacts with) SM.C16.1 |
| 0.877315509 | 10.80002242 | PC.aa.C42.0 (interacts with) PC.ae.C40.4 |
| 0.877124806 | 6.56105004 | PC.ae.C32.1 (interacts with) PC.ae.C34.3 |
| 0.876942071 | 4.500544051 | PC.aa.C36.5 (interacts with) PC.aa.C40.6 |
| 0.876863222 | 5.414057146 | PC.ae.C38.0 (interacts with) SM.C24.0 |
| 0.876630591 | 11.8719062 | PC.ae.C32.1 (interacts with) PC.ae.C42.2 |
| 0.876607088 | 39.12728034 | PC.ae.C42.5 (interacts with) SM.C16.0 |
| 0.876579032 | 6.837588275 | PC.aa.C32.2 (interacts with) PC.aa.C40.4 |
| 0.876521079 | 14.01156497 | PC.ae.C40.2 (interacts with) SM.OH.C14.1 |
| 0.876519453 | 22.26167821 | PC.ae.C32.2 (interacts with) PC.ae.C42.5 |
| 0.876408119 | 5.488300516 | PC.ae.C38.6 (interacts with) SM.OH.C16.1 |
| 0.875895708 | 8.058652391 | PC.ae.C36.5 (interacts with) PC.ae.C38.3 |
| 0.875314516 | 30.36263632 | PC.aa.C34.3 (interacts with) PC.aa.C36.2 |
| 0.87517722 | 4.148188314 | PC.aa.C36.0 (interacts with) PC.ae.C36.0 |
| 0.874815809 | 10.02363688 | PC.ae.C36.5 (interacts with) PC.ae.C42.2 |
| 0.874779354 | 6.000562806 | PC.ae.C40.5 (interacts with) SM.OH.C22.1 |
| 0.874586232 | 8.32827972 | PC.ae.C38.4 (interacts with) PC.ae.C40.3 |
| 0.874504146 | 12.22343683 | PC.ae.C38.0 (interacts with) PC.ae.C40.2 |
| 0.874342557 | 7.891133196 | PC.ae.C38.6 (interacts with) PC.ae.C40.4 |
| 0.874330055 | 8.338130875 | lysoPC.a.C18.0 (interacts with) PC.aa.C34.4 |
| 0.874235812 | 10.8772558 | PC.aa.C34.1 (interacts with) PC.aa.C34.3 |
| 0.874232016 | 6.735645683 | PC.ae.C42.3 (interacts with) SM.C24.0 |
| 0.873890505 | 23.79151875 | PC.ae.C36.3 (interacts with) PC.ae.C38.4 |
| 0.873448265 | 6.68658888 | PC.aa.C28.1 (interacts with) PC.ae.C32.1 |
| 0.873046158 | 10.51838753 | PC.ae.C38.4 (interacts with) SM.OH.C22.1 |
| 0.873017492 | 9.093526278 | PC.ae.C38.3 (interacts with) SM.C24.0 |
| 0.872731495 | 13.19819493 | PC.aa.C40.6 (interacts with) SM.OH.C22.1 |
| 0.872701547 | 10.38721622 | PC.aa.C42.0 (interacts with) PC.ae.C32.2 |
| 0.872462979 | 7.542858865 | PC.aa.C28.1 (interacts with) PC.aa.C42.5 |
| 0.872433749 | 5.069429562 | PC.ae.C38.5 (interacts with) SM.OH.C16.1 |
| 0.87236614 | 7.718815321 | PC.ae.C38.5 (interacts with) SM.OH.C22.1 |
| 0.872073797 | 12.89824614 | PC.aa.C40.6 (interacts with) PC.ae.C40.6 |
| 0.872008498 | 7.340911103 | PC.aa.C28.1 (interacts with) PC.ae.C42.3 |
| 0.871155319 | 17.03753746 | PC.ae.C34.2 (interacts with) PC.ae.C38.4 |
| 0.871154953 | 9.26607884 | PC.aa.C34.4 (interacts with) PC.aa.C40.5 |
| 0.870876042 | 12.71032535 | lysoPC.a.C18.0 (interacts with) PC.aa.C36.4 |
| 0.870764874 | 5.015921555 | PC.ae.C32.2 (interacts with) PC.ae.C36.2 |
| 0.870726372 | 6.302706695 | PC.ae.C30.0 (interacts with) PC.ae.C36.2 |
| 0.870719867 | 8.114655578 | PC.aa.C30.0 (interacts with) PC.aa.C36.1 |
| 0.870507128 | 9.55113701 | PC.ae.C40.4 (interacts with) PC.ae.C42.2 |
| 0.870326688 | 21.15346076 | PC.ae.C40.2 (interacts with) SM.C16.0 |
| 0.87024648 | 7.333024084 | PC.ae.C32.2 (interacts with) PC.ae.C34.0 |
| 0.870145939 | 6 | Creatinine (interacts with) total.DMA |
| 0.870091669 | 6.801398971 | lysoPC.a.C17.0 (interacts with) PC.aa.C42.6 |
| 0.870051193 | 10.52957569 | PC.ae.C40.5 (interacts with) SM.OH.C22.2 |
| 0.870017614 | 13.65468615 | PC.aa.C36.5 (interacts with) SM.C24.0 |
| 0.869761807 | 7.264415477 | PC.ae.C32.1 (interacts with) SM.OH.C14.1 |
| 0.869337335 | 5.558350364 | PC.aa.C30.0 (interacts with) PC.aa.C34.3 |
| 0.869227302 | 12.8544168 | PC.ae.C38.6 (interacts with) SM.C24.1 |
| 0.869146691 | 8.315981644 | lysoPC.a.C17.0 (interacts with) PC.ae.C36.2 |
| 0.86902243 | 22.79589979 | PC.aa.C36.2 (interacts with) PC.ae.C38.3 |
| 0.869021474 | 25.50549766 | PC.aa.C38.5 (interacts with) PC.ae.C38.0 |
| 0.869007292 | 24.67731055 | PC.aa.C36.6 (interacts with) PC.ae.C40.1 |
| 0.86869611 | 5.194180835 | PC.ae.C32.2 (interacts with) SM.OH.C16.1 |
| 0.868203005 | 4.068100261 | PC.ae.C30.0 (interacts with) PC.ae.C34.3 |
| 0.868174464 | 24.09193378 | PC.ae.C34.1 (interacts with) PC.ae.C38.4 |
| 0.868080708 | 10.26677732 | PC.ae.C32.1 (interacts with) PC.ae.C34.0 |
| 0.868076378 | 6.222577544 | PC.ae.C42.2 (interacts with) SM.OH.C14.1 |
| 0.868043857 | 12.96397949 | PC.ae.C38.3 (interacts with) SM.OH.C16.1 |
| 0.867924704 | 4.101486145 | PC.aa.C42.5 (interacts with) PC.ae.C38.0 |
| 0.867884178 | 7.329044345 | PC.ae.C32.1 (interacts with) PC.ae.C40.3 |
| 0.867427125 | 32.46589917 | PC.ae.C32.1 (interacts with) PC.ae.C42.5 |
| 0.867288233 | 9.429384682 | PC.aa.C36.3 (interacts with) PC.aa.C40.5 |
| 0.867243722 | 10.54456641 | lysoPC.a.C17.0 (interacts with) PC.ae.C42.2 |
| 0.867120432 | 7.435149345 | PC.ae.C40.1 (interacts with) SM.C24.0 |
| 0.866943518 | 17.79145787 | SM.OH.C24.1 (interacts with) SM.C18.1 |
| 0.866872467 | 37.15143053 | PC.aa.C36.0 (interacts with) PC.ae.C40.5 |
| 0.86674964 | 31.45261871 | PC.aa.C36.4 (interacts with) PC.ae.C38.4 |
| 0.866691022 | 10.05536752 | PC.aa.C42.0 (interacts with) PC.ae.C42.4 |
| 0.866676262 | 23.52600161 | PC.aa.C34.2 (interacts with) PC.aa.C34.3 |
| 0.866647278 | 11.44108266 | PC.aa.C36.2 (interacts with) PC.ae.C34.2 |
| 0.866588235 | 43.72976021 | PC.aa.C28.1 (interacts with) PC.aa.C32.0 |
| 0.866185624 | 7.030491395 | PC.ae.C34.0 (interacts with) PC.ae.C38.0 |
| 0.866063068 | 14.21171138 | PC.ae.C40.2 (interacts with) SM.OH.C24.1 |
| 0.865980374 | 25.12264066 | SM.OH.C22.1 (interacts with) SM.C18.1 |
| 0.86585926 | 6.136230847 | PC.ae.C36.5 (interacts with) PC.ae.C40.4 |
| 0.865692698 | 27.00726076 | PC.aa.C36.5 (interacts with) PC.ae.C42.2 |
| 0.865685535 | 26.13821295 | lysoPC.a.C16.0 (interacts with) PC.aa.C32.2 |
| 0.865414686 | 7.02731454 | PC.ae.C36.2 (interacts with) PC.ae.C42.2 |
| 0.865360205 | 7.906807936 | PC.aa.C30.0 (interacts with) PC.aa.C36.3 |
| 0.865185566 | 21.3248731 | lysoPC.a.C16.0 (interacts with) lysoPC.a.C18.1 |
| 0.865127735 | 13.49744441 | PC.aa.C38.4 (interacts with) SM.OH.C24.1 |
| 0.864858099 | 9.833296286 | PC.aa.C38.0 (interacts with) SM.OH.C16.1 |
| 0.864366623 | 5.539988325 | PC.ae.C36.2 (interacts with) PC.ae.C38.5 |
| 0.864355206 | 7.391932284 | PC.ae.C36.2 (interacts with) SM.C16.0 |
| 0.86432002 | 8.514356498 | PC.ae.C32.1 (interacts with) SM.C16.1 |
| 0.864165381 | 5.52520137 | PC.aa.C38.6 (interacts with) PC.aa.C42.5 |
| 0.864131844 | 17.84485963 | PC.aa.C38.4 (interacts with) PC.ae.C38.4 |
| 0.864021503 | 15.94025603 | PC.ae.C36.1 (interacts with) SM.C16.1 |
| 0.863942244 | 7.561117131 | PC.ae.C34.0 (interacts with) PC.ae.C40.4 |
| 0.863918345 | 23.19873592 | PC.aa.C38.0 (interacts with) PC.ae.C32.2 |
| 0.863669041 | 9.309605963 | PC.ae.C42.3 (interacts with) SM.C16.0 |
| 0.863658744 | 32.22054756 | PC.aa.C34.2 (interacts with) PC.aa.C36.3 |
| 0.863655392 | 25.49105281 | PC.ae.C38.2 (interacts with) PC.ae.C40.1 |
| 0.863632055 | 8.145393933 | PC.aa.C38.6 (interacts with) PC.aa.C42.6 |
| 0.863623852 | 2 | Arg (interacts with) Met |
| 0.863118149 | 8.471756607 | PC.ae.C34.0 (interacts with) PC.ae.C36.5 |
| 0.863067333 | 6.508661897 | PC.ae.C30.0 (interacts with) PC.ae.C34.2 |
| 0.863050184 | 19.38352706 | PC.aa.C38.1 (interacts with) PC.ae.C40.6 |
| 0.862999647 | 23.10556711 | PC.ae.C36.3 (interacts with) PC.ae.C42.4 |
| 0.862871008 | 14.6471677 | PC.aa.C38.0 (interacts with) SM.OH.C22.1 |
| 0.862853257 | 4.624159333 | PC.ae.C32.2 (interacts with) PC.ae.C38.4 |
| 0.862732834 | 19.87713034 | PC.ae.C34.2 (interacts with) PC.ae.C38.3 |
| 0.862574537 | 15.48907371 | PC.aa.C38.0 (interacts with) PC.aa.C38.1 |
| 0.862351407 | 10.24738493 | PC.ae.C38.4 (interacts with) PC.ae.C42.3 |
| 0.862035581 | 10.11084147 | PC.ae.C34.0 (interacts with) PC.ae.C38.6 |
| 0.861978352 | 6.682556673 | PC.aa.C38.6 (interacts with) SM.OH.C16.1 |
| 0.861929522 | 8.203488906 | PC.ae.C36.5 (interacts with) SM.C16.0 |
| 0.861878598 | 30.23556587 | PC.ae.C34.0 (interacts with) PC.ae.C34.1 |
| 0.861794337 | 6.726674601 | PC.ae.C34.1 (interacts with) PC.ae.C34.3 |
| 0.861692553 | 8.040732144 | PC.ae.C42.3 (interacts with) SM.OH.C14.1 |
| 0.861622168 | 27.90691373 | PC.aa.C28.1 (interacts with) PC.aa.C32.3 |
| 0.861617842 | 2 | C10.1 (interacts with) C14.1 |
| 0.861583826 | 4.692931244 | PC.ae.C32.1 (interacts with) PC.ae.C36.2 |
| 0.861143162 | 8.655301051 | PC.aa.C32.0 (interacts with) PC.aa.C32.3 |
| 0.861053071 | 32.95701723 | lysoPC.a.C20.3 (interacts with) PC.aa.C40.4 |
| 0.861026464 | 41.572289 | lysoPC.a.C16.1 (interacts with) PC.aa.C34.1 |
| 0.860976832 | 11.65093505 | PC.aa.C42.5 (interacts with) PC.ae.C38.3 |
| 0.860920983 | 8.243865751 | PC.ae.C36.4 (interacts with) PC.ae.C42.4 |
| 0.860515693 | 20.68221738 | PC.ae.C42.4 (interacts with) PC.ae.C42.5 |
| 0.860430823 | 22.70982319 | PC.aa.C34.2 (interacts with) PC.ae.C36.2 |
| 0.860323784 | 8.722232609 | PC.aa.C42.5 (interacts with) SM.C16.0 |
| 0.859708021 | 14.42777389 | PC.ae.C36.0 (interacts with) PC.ae.C40.6 |
| 0.859562824 | 13.78511966 | PC.ae.C34.1 (interacts with) PC.ae.C34.2 |
| 0.858785504 | 6.100246691 | PC.ae.C34.0 (interacts with) SM.C16.0 |
| 0.858740495 | 10.37314274 | PC.aa.C32.2 (interacts with) PC.aa.C34.2 |
| 0.858590981 | 11.03158944 | PC.ae.C36.2 (interacts with) PC.ae.C40.1 |
| 0.857988059 | 7.080524955 | PC.ae.C34.0 (interacts with) SM.OH.C16.1 |
| 0.857980539 | 86 | Trp (interacts with) Val |
| 0.857857258 | 14.44983641 | PC.aa.C38.4 (interacts with) SM.C16.1 |
| 0.857427869 | 14.9200683 | PC.aa.C40.6 (interacts with) SM.C24.0 |
| 0.857198813 | 8.684468922 | PC.ae.C40.3 (interacts with) SM.C16.1 |
| 0.857125583 | 9.531457002 | PC.ae.C32.1 (interacts with) PC.ae.C36.1 |
| 0.856756261 | 8.493390282 | PC.ae.C42.3 (interacts with) SM.OH.C24.1 |
| 0.856658047 | 24.49104063 | lysoPC.a.C18.0 (interacts with) PC.ae.C40.1 |
| 0.856554262 | 47.4110058 | PC.aa.C32.0 (interacts with) PC.aa.C40.4 |
| 0.856419386 | 10.10234528 | PC.ae.C34.3 (interacts with) PC.ae.C36.5 |
| 0.856141244 | 9.500953967 | PC.aa.C40.6 (interacts with) PC.aa.C42.5 |
| 0.855806936 | 21.67099467 | PC.ae.C38.1 (interacts with) PC.ae.C42.3 |
| 0.855592399 | 32.09406462 | PC.aa.C32.2 (interacts with) PC.aa.C34.1 |
| 0.855579587 | 8.341699712 | PC.ae.C36.2 (interacts with) PC.ae.C40.4 |
| 0.855332002 | 7.274036249 | PC.ae.C34.0 (interacts with) PC.ae.C38.5 |
| 0.855286932 | 7.131140001 | PC.ae.C36.5 (interacts with) SM.C16.1 |
| 0.855204082 | 10.37236449 | PC.ae.C36.5 (interacts with) PC.ae.C42.3 |
| 0.855178274 | 25.43310051 | lysoPC.a.C16.0 (interacts with) PC.aa.C38.3 |
| 0.855059369 | 27.6469075 | PC.aa.C32.0 (interacts with) PC.ae.C38.3 |
| 0.855013871 | 26.57749227 | PC.ae.C40.6 (interacts with) PC.ae.C42.3 |
| 0.854793423 | 38.40587433 | PC.aa.C34.1 (interacts with) PC.aa.C38.3 |
| 0.854344927 | 8.75016512 | PC.aa.C38.6 (interacts with) SM.C24.0 |
| 0.854143288 | 8.209824115 | PC.aa.C38.6 (interacts with) SM.C18.0 |
| 0.85406315 | 13.34711406 | PC.aa.C38.6 (interacts with) SM.C24.1 |
| 0.853924525 | 8.439652916 | PC.ae.C42.2 (interacts with) SM.OH.C16.1 |
| 0.853806939 | 28.1525557 | PC.ae.C40.2 (interacts with) PC.ae.C40.3 |
| 0.853769678 | 13.73168006 | PC.ae.C38.3 (interacts with) PC.ae.C40.1 |
| 0.853623954 | 8.480571926 | PC.ae.C38.0 (interacts with) PC.ae.C42.2 |
| 0.853516986 | 7.56602366 | PC.aa.C42.5 (interacts with) PC.ae.C42.3 |
| 0.853442458 | 25.0643837 | PC.ae.C38.5 (interacts with) PC.ae.C42.5 |
| 0.853393721 | 7.889307269 | PC.ae.C34.0 (interacts with) PC.ae.C38.4 |
| 0.853343847 | 33.1717448 | PC.aa.C28.1 (interacts with) SM.OH.C22.2 |
| 0.853319505 | 7.975113659 | PC.aa.C30.0 (interacts with) PC.aa.C40.5 |
| 0.853007149 | 16.84988367 | PC.ae.C40.2 (interacts with) SM.C16.1 |
| 0.852890443 | 13.32527996 | PC.aa.C32.3 (interacts with) PC.ae.C40.1 |
| 0.852766379 | 6.067733127 | PC.ae.C34.0 (interacts with) SM.C16.1 |
| 0.852703799 | 4.254381842 | PC.aa.C30.0 (interacts with) PC.aa.C38.3 |
| 0.85237727 | 20.50215991 | PC.aa.C32.0 (interacts with) PC.aa.C32.2 |
| 0.852223004 | 13.05624409 | PC.ae.C30.0 (interacts with) PC.ae.C42.4 |
| 0.85183367 | 15.48062541 | PC.aa.C36.2 (interacts with) PC.ae.C36.1 |
| 0.851751625 | 8.609338954 | PC.ae.C42.3 (interacts with) SM.C16.1 |
| 0.851700564 | 33.13728304 | PC.aa.C28.1 (interacts with) PC.aa.C36.2 |
| 0.851691715 | 14.82215142 | PC.aa.C40.6 (interacts with) SM.OH.C22.2 |
| 0.851539487 | 18.25472542 | PC.aa.C38.5 (interacts with) PC.ae.C34.0 |
| 0.851465452 | 50.33379051 | lysoPC.a.C18.0 (interacts with) PC.aa.C38.4 |
| 0.851291014 | 4.917332551 | PC.ae.C40.4 (interacts with) SM.OH.C16.1 |
| 0.851073131 | 34.87778818 | PC.aa.C32.3 (interacts with) PC.ae.C34.0 |
| 0.851057273 | 11.98430546 | lysoPC.a.C18.0 (interacts with) PC.aa.C32.3 |
| 0.850904109 | 7.429611428 | PC.ae.C38.6 (interacts with) SM.OH.C24.1 |
| 0.850420249 | 7.091210086 | PC.aa.C42.5 (interacts with) PC.ae.C38.6 |
| 0.850318404 | 42.45654525 | PC.aa.C36.2 (interacts with) PC.aa.C36.3 |
| 0.850250969 | 18.68683671 | PC.aa.C42.6 (interacts with) PC.ae.C38.3 |
| 0.850174769 | 17.20719147 | lysoPC.a.C17.0 (interacts with) SM.OH.C22.1 |
| 0.850136345 | 4.315618522 | PC.ae.C40.4 (interacts with) SM.OH.C14.1 |
| 0.850134057 | 23.16062451 | PC.aa.C38.0 (interacts with) PC.ae.C38.5 |
| 0.850125475 | 18.22986 | PC.aa.C36.4 (interacts with) PC.aa.C38.3 |
| 0.850037698 | 7.303089804 | PC.aa.C34.2 (interacts with) PC.ae.C34.1 |
| 0.849817984 | 17.14424782 | PC.ae.C36.1 (interacts with) SM.C16.0 |
| 0.849811769 | 32.03407775 | PC.aa.C30.0 (interacts with) PC.aa.C34.1 |
| 0.849608058 | 15.82217472 | PC.ae.C36.4 (interacts with) SM.C16.0 |
| 0.84939831 | 6.550381581 | PC.aa.C28.1 (interacts with) PC.ae.C32.2 |
| 0.849288865 | 13.37275749 | PC.aa.C32.2 (interacts with) PC.aa.C36.2 |
| 0.849224029 | 8.914903179 | PC.ae.C42.3 (interacts with) SM.OH.C16.1 |
| 0.849202876 | 11.18028561 | PC.ae.C40.3 (interacts with) SM.OH.C24.1 |
| 0.849196064 | 22.46751906 | PC.ae.C42.3 (interacts with) SM.OH.C22.2 |
| 0.849082049 | 6.555201946 | PC.aa.C42.5 (interacts with) SM.OH.C24.1 |
| 0.848486145 | 20.12358411 | PC.ae.C40.1 (interacts with) SM.OH.C22.1 |
| 0.848476207 | 6.517603561 | PC.aa.C34.3 (interacts with) PC.aa.C38.3 |
| 0.848269235 | 13.68361861 | lysoPC.a.C17.0 (interacts with) PC.ae.C42.3 |
| 0.848154599 | 7.668436888 | SM.C16.1 (interacts with) SM.C24.0 |
| 0.848044613 | 10.6264023 | PC.aa.C36.5 (interacts with) PC.aa.C38.6 |
| 0.84799941 | 50.53826287 | PC.aa.C32.0 (interacts with) PC.ae.C42.2 |
| 0.847751378 | 25.67808461 | PC.ae.C40.3 (interacts with) SM.OH.C22.2 |
| 0.847422205 | 9.657462757 | PC.aa.C38.6 (interacts with) SM.C16.1 |
| 0.847358485 | 5.887367268 | lysoPC.a.C17.0 (interacts with) SM.C24.0 |
| 0.847132096 | 30.17568697 | PC.ae.C36.1 (interacts with) SM.OH.C16.1 |
| 0.847084125 | 16.30238078 | PC.ae.C38.6 (interacts with) PC.ae.C40.3 |
| 0.847083068 | 12.22582035 | PC.ae.C36.5 (interacts with) PC.ae.C40.5 |
| 0.84708189 | 8.328089127 | PC.ae.C32.2 (interacts with) PC.ae.C42.2 |
| 0.846960976 | 11.21326992 | PC.aa.C42.0 (interacts with) PC.ae.C38.5 |
| 0.846661376 | 8.420294947 | PC.ae.C34.2 (interacts with) PC.ae.C36.5 |
| 0.84666032 | 19.67235207 | PC.ae.C30.0 (interacts with) PC.ae.C42.3 |
| 0.846525438 | 15.08158757 | PC.aa.C36.6 (interacts with) PC.ae.C40.6 |
| 0.846515344 | 8.407430239 | PC.ae.C40.4 (interacts with) SM.OH.C22.1 |
| 0.846418103 | 8.682844332 | PC.ae.C32.2 (interacts with) SM.OH.C22.1 |
| 0.846006487 | 16.18134282 | PC.aa.C36.0 (interacts with) PC.aa.C36.6 |
| 0.845876 | 5.11016347 | PC.aa.C34.3 (interacts with) PC.aa.C40.5 |
| 0.845545498 | 8.313063672 | PC.aa.C38.1 (interacts with) SM.C24.1 |
| 0.845167423 | 16.97326976 | PC.ae.C32.2 (interacts with) SM.OH.C22.2 |
| 0.845076899 | 14.1634766 | PC.aa.C28.1 (interacts with) PC.ae.C40.3 |
| 0.845057561 | 15.30331156 | PC.ae.C34.1 (interacts with) PC.ae.C36.2 |
| 0.845052715 | 7.548373987 | PC.ae.C38.6 (interacts with) SM.OH.C14.1 |
| 0.845006368 | 12.64204904 | PC.ae.C32.1 (interacts with) PC.ae.C42.3 |
| 0.844996976 | 5.88061082 | PC.ae.C40.4 (interacts with) SM.OH.C24.1 |
| 0.844982099 | 13.18755671 | PC.ae.C40.1 (interacts with) PC.ae.C42.2 |
| 0.844959711 | 31.73544805 | PC.aa.C32.2 (interacts with) PC.ae.C34.1 |
| 0.844718464 | 88.44289961 | PC.aa.C30.0 (interacts with) PC.ae.C36.1 |
| 0.844687825 | 6.179312407 | PC.aa.C36.2 (interacts with) PC.ae.C34.1 |
| 0.844636124 | 51.77435517 | PC.aa.C32.0 (interacts with) PC.ae.C34.0 |
| 0.844508392 | 11.72173557 | SM.OH.C24.1 (interacts with) SM.C18.0 |
| 0.844131795 | 25.38361528 | PC.aa.C36.5 (interacts with) PC.ae.C34.0 |
| 0.844048857 | 16.639177 | PC.ae.C38.5 (interacts with) SM.OH.C22.2 |
| 0.843567269 | 11.96074818 | PC.aa.C38.0 (interacts with) PC.ae.C36.0 |
| 0.843546747 | 17.0340497 | PC.ae.C38.4 (interacts with) PC.ae.C42.4 |
| 0.843507179 | 19.08646272 | PC.aa.C36.5 (interacts with) SM.OH.C22.1 |
| 0.843465804 | 12.04611892 | PC.aa.C38.6 (interacts with) PC.ae.C42.2 |
| 0.8433622 | 17.43459995 | PC.aa.C38.6 (interacts with) SM.C18.1 |
| 0.842843338 | 7.174630131 | PC.ae.C36.5 (interacts with) SM.OH.C24.1 |
| 0.84280173 | 16.38470205 | PC.aa.C38.0 (interacts with) PC.ae.C40.4 |
| 0.842694404 | 15.92252016 | PC.aa.C32.0 (interacts with) PC.aa.C34.4 |
| 0.842348966 | 4.109309285 | PC.aa.C38.1 (interacts with) PC.ae.C36.0 |
| 0.842338059 | 31.74358639 | PC.aa.C38.5 (interacts with) SM.OH.C22.1 |
| 0.842097163 | 9.24275104 | PC.ae.C32.2 (interacts with) PC.ae.C36.4 |
| 0.841714907 | 33.70014832 | PC.aa.C28.1 (interacts with) SM.C18.0 |
| 0.841281167 | 15.67005611 | PC.aa.C42.6 (interacts with) PC.ae.C40.5 |
| 0.84107236 | 6.454156558 | PC.aa.C36.6 (interacts with) PC.aa.C38.6 |
| 0.84092247 | 7.231263998 | PC.aa.C42.5 (interacts with) SM.C16.1 |
| 0.840834017 | 8.360686026 | PC.aa.C38.6 (interacts with) SM.OH.C24.1 |
| 0.840831117 | 19.02465663 | PC.ae.C36.1 (interacts with) SM.OH.C24.1 |
| 0.840347103 | 7.34417448 | lysoPC.a.C17.0 (interacts with) PC.aa.C38.5 |
| 0.840333092 | 28.46318545 | lysoPC.a.C18.0 (interacts with) PC.aa.C34.3 |
| 0.840308525 | 12.40147774 | PC.ae.C36.2 (interacts with) PC.ae.C40.3 |
| 0.84027129 | 13.56185017 | PC.ae.C36.4 (interacts with) SM.C16.1 |
| 0.840140389 | 16.77719437 | PC.aa.C32.0 (interacts with) PC.aa.C38.3 |
| 0.840066503 | 79.16793436 | PC.aa.C32.3 (interacts with) PC.aa.C36.3 |
| 0.840059493 | 15.36227507 | PC.ae.C36.0 (interacts with) PC.ae.C38.6 |
| 0.839943512 | 31.61986839 | lysoPC.a.C18.0 (interacts with) PC.ae.C36.1 |
| 0.839673817 | 22.18572786 | PC.ae.C32.2 (interacts with) PC.ae.C40.6 |
| 0.839611879 | 9.12972649 | PC.aa.C38.5 (interacts with) PC.ae.C40.1 |
| 0.839502821 | 51.42416995 | PC.ae.C34.1 (interacts with) SM.C16.0 |
| 0.839353737 | 18.28123849 | SM.OH.C16.1 (interacts with) SM.C24.1 |
| 0.839228316 | 22.70693007 | PC.ae.C36.3 (interacts with) PC.ae.C36.5 |
| 0.839197742 | 31.38665359 | PC.ae.C38.1 (interacts with) PC.ae.C38.3 |
| 0.839015467 | 72.88118501 | PC.aa.C34.4 (interacts with) PC.ae.C36.1 |
| 0.838991202 | 13.99882258 | PC.ae.C34.1 (interacts with) PC.ae.C36.4 |
| 0.838800875 | 61.27607218 | PC.aa.C32.0 (interacts with) PC.aa.C40.5 |
| 0.838757679 | 8.767217268 | PC.aa.C38.6 (interacts with) PC.ae.C40.5 |
| 0.838672322 | 21.31324349 | PC.ae.C34.2 (interacts with) PC.ae.C38.5 |
| 0.83836697 | 6.980658322 | PC.aa.C42.5 (interacts with) SM.OH.C16.1 |
| 0.838149645 | 14.45700964 | lysoPC.a.C17.0 (interacts with) PC.ae.C38.0 |
| 0.838104782 | 33.35896597 | PC.aa.C36.0 (interacts with) PC.ae.C38.0 |
| 0.838057251 | 15.86830954 | PC.ae.C40.4 (interacts with) PC.ae.C40.6 |
| 0.837994111 | 10.58458721 | SM.C16.0 (interacts with) SM.C24.0 |
| 0.837781594 | 17.68949226 | PC.ae.C30.0 (interacts with) SM.OH.C14.1 |
| 0.837738783 | 16.80199671 | PC.ae.C40.3 (interacts with) SM.OH.C22.1 |
| 0.837312865 | 7.793964827 | PC.ae.C38.0 (interacts with) SM.OH.C24.1 |
| 0.837107203 | 6.233112478 | PC.ae.C34.0 (interacts with) SM.OH.C24.1 |
| 0.836762319 | 8.493116524 | PC.ae.C38.0 (interacts with) SM.OH.C16.1 |
| 0.836556116 | 56.59991851 | lysoPC.a.C18.0 (interacts with) PC.ae.C38.3 |
| 0.836115775 | 4 | C18.1 (interacts with) C18.2 |
| 0.835160153 | 18.34467204 | PC.aa.C38.4 (interacts with) SM.OH.C22.1 |
| 0.83515269 | 28.79183767 | PC.ae.C36.0 (interacts with) PC.ae.C40.4 |
| 0.835044484 | 17.78056537 | PC.ae.C30.0 (interacts with) PC.ae.C42.2 |
| 0.834857976 | 18.48746338 | PC.aa.C36.6 (interacts with) PC.ae.C34.0 |
| 0.834634891 | 7.156032523 | PC.ae.C36.2 (interacts with) PC.ae.C36.4 |
| 0.834569408 | 5.03871346 | PC.ae.C40.5 (interacts with) SM.OH.C16.1 |
| 0.834078664 | 161.0741705 | PC.ae.C42.5 (interacts with) PC.ae.C44.5 |
| 0.83377251 | 22.99511068 | lysoPC.a.C18.0 (interacts with) PC.aa.C38.5 |
| 0.833625806 | 51.84829809 | PC.ae.C34.1 (interacts with) SM.OH.C14.1 |
| 0.833397234 | 6.779975643 | PC.aa.C28.1 (interacts with) PC.ae.C38.5 |
| 0.833395676 | 15.38109984 | PC.ae.C40.2 (interacts with) PC.ae.C40.4 |
| 0.833348544 | 14.09189928 | PC.ae.C34.3 (interacts with) PC.ae.C36.1 |
| 0.833240814 | 16.81161611 | PC.ae.C36.1 (interacts with) PC.ae.C36.3 |
| 0.833230675 | 36.73025696 | PC.ae.C40.2 (interacts with) SM.C18.1 |
| 0.832936937 | 9.818325009 | PC.aa.C36.2 (interacts with) PC.aa.C36.4 |
| 0.83281225 | 16.25111542 | PC.aa.C36.6 (interacts with) PC.ae.C40.5 |
| 0.832791284 | 11.165842 | PC.aa.C28.1 (interacts with) PC.aa.C42.6 |
| 0.832780265 | 28.94863797 | lysoPC.a.C16.0 (interacts with) lysoPC.a.C18.2 |
| 0.832627982 | 37.76057151 | PC.aa.C36.2 (interacts with) PC.ae.C38.4 |
| 0.832428527 | 8.20377251 | PC.ae.C40.5 (interacts with) SM.C16.0 |
| 0.832176814 | 7.496895325 | PC.aa.C42.5 (interacts with) SM.OH.C14.1 |
| 0.832106331 | 54.41897498 | PC.aa.C32.1 (interacts with) PC.aa.C36.3 |
| 0.832105934 | 29.75491069 | PC.ae.C38.3 (interacts with) PC.ae.C42.4 |
| 0.832065109 | 33.63615188 | PC.aa.C38.0 (interacts with) PC.ae.C42.3 |
| 0.8319698 | 9.258253594 | PC.aa.C34.2 (interacts with) PC.ae.C30.0 |
| 0.831682341 | 10.8033845 | PC.aa.C34.4 (interacts with) PC.aa.C36.2 |
| 0.831680002 | 5.671099495 | PC.ae.C40.4 (interacts with) SM.C16.1 |
| 0.8315102 | 4.752563641 | PC.ae.C36.2 (interacts with) PC.ae.C36.5 |
| 0.831368296 | 11.06272807 | PC.ae.C36.5 (interacts with) SM.OH.C22.1 |
| 0.83094308 | 19.2314414 | PC.ae.C32.2 (interacts with) PC.ae.C34.2 |
| 0.830873327 | 10.96424168 | PC.aa.C42.6 (interacts with) PC.ae.C38.6 |
| 0.830816347 | 20.9681037 | PC.aa.C38.5 (interacts with) PC.ae.C36.1 |
| 0.830765195 | 15.04521557 | PC.ae.C40.3 (interacts with) PC.ae.C42.4 |
| 0.830572403 | 12.16100443 | lysoPC.a.C17.0 (interacts with) PC.aa.C36.2 |
| 0.830414491 | 20.73902068 | PC.ae.C38.3 (interacts with) PC.ae.C40.5 |
| 0.830393234 | 25.80572003 | PC.aa.C36.4 (interacts with) PC.ae.C38.3 |
| 0.830003362 | 8.64003485 | PC.aa.C36.2 (interacts with) PC.ae.C30.0 |
| 0.829820403 | 42.06562275 | PC.aa.C36.4 (interacts with) PC.aa.C40.4 |
| 0.829713623 | 10.32654699 | PC.ae.C30.0 (interacts with) PC.ae.C38.4 |
| 0.829471366 | 26.53665798 | PC.ae.C40.2 (interacts with) PC.ae.C42.3 |
| 0.829224256 | 20.49675158 | PC.ae.C36.1 (interacts with) PC.ae.C38.5 |
| 0.829210534 | 16.58723311 | PC.aa.C32.3 (interacts with) PC.ae.C34.2 |
| 0.829197582 | 8.016092217 | PC.aa.C36.6 (interacts with) PC.ae.C38.6 |
| 0.829043149 | 49.11701587 | PC.aa.C34.4 (interacts with) PC.ae.C40.1 |
| 0.829030253 | 14.73667809 | PC.aa.C42.5 (interacts with) SM.OH.C22.2 |
| 0.828161715 | 13.81754656 | PC.ae.C30.0 (interacts with) PC.ae.C32.2 |
| 0.82807395 | 23.97711758 | PC.aa.C32.2 (interacts with) PC.ae.C36.3 |
| 0.827909469 | 40.19192002 | lysoPC.a.C20.3 (interacts with) PC.aa.C40.5 |
| 0.82775969 | 10.81716356 | lysoPC.a.C17.0 (interacts with) PC.aa.C32.3 |
| 0.827705144 | 29.90607873 | PC.ae.C42.2 (interacts with) SM.OH.C22.2 |
| 0.827544271 | 34.62030189 | PC.aa.C38.5 (interacts with) PC.aa.C38.6 |
| 0.827416051 | 17.29628152 | PC.aa.C34.4 (interacts with) PC.aa.C36.4 |
| 0.826900396 | 21.46458354 | PC.ae.C34.3 (interacts with) PC.ae.C38.5 |
| 0.826828031 | 28.47426869 | SM.OH.C22.1 (interacts with) SM.C24.1 |
| 0.826823835 | 8.029325346 | PC.aa.C32.0 (interacts with) PC.aa.C34.2 |
| 0.826707756 | 30.51940609 | PC.aa.C38.0 (interacts with) SM.C16.0 |
| 0.826448796 | 8.659357006 | PC.aa.C42.5 (interacts with) PC.ae.C40.5 |
| 0.826396331 | 5.615525007 | PC.ae.C32.2 (interacts with) SM.OH.C24.1 |
| 0.826107428 | 17.12486119 | PC.aa.C36.5 (interacts with) PC.ae.C38.6 |
| 0.826010984 | 22.54750301 | PC.ae.C34.0 (interacts with) SM.OH.C22.2 |
| 0.825740544 | 20.6612302 | PC.aa.C38.0 (interacts with) SM.C16.1 |
| 0.825616206 | 17.95955379 | PC.aa.C36.6 (interacts with) PC.ae.C42.3 |
| 0.825248765 | 31.48118444 | PC.aa.C34.2 (interacts with) PC.ae.C38.3 |
| 0.825209191 | 25.95646968 | PC.aa.C38.1 (interacts with) PC.ae.C38.6 |
| 0.825140081 | 6.622394898 | PC.aa.C32.2 (interacts with) PC.aa.C38.3 |
| 0.824954524 | 14.78392851 | lysoPC.a.C17.0 (interacts with) SM.OH.C24.1 |
| 0.824953069 | 25.02944656 | PC.aa.C36.6 (interacts with) PC.aa.C38.1 |
| 0.824930632 | 25.01613589 | lysoPC.a.C18.0 (interacts with) PC.aa.C40.5 |
| 0.824922905 | 19.40349404 | PC.ae.C30.0 (interacts with) SM.C16.0 |
| 0.824876344 | 6 | C4 (interacts with) Cit |
| 0.824673606 | 9.420442134 | PC.ae.C38.0 (interacts with) SM.C16.1 |
| 0.824431645 | 31.45351711 | PC.ae.C34.1 (interacts with) PC.ae.C42.2 |
| 0.824268182 | 14.73680441 | PC.aa.C40.6 (interacts with) PC.ae.C40.2 |
| 0.824146314 | 70.93165791 | lysoPC.a.C18.0 (interacts with) PC.aa.C42.6 |
| 0.824025709 | 14.11393801 | PC.ae.C40.4 (interacts with) SM.OH.C22.2 |
| 0.823933269 | 15.49930157 | PC.ae.C34.0 (interacts with) PC.ae.C40.3 |
| 0.823831847 | 15.52108462 | PC.ae.C40.3 (interacts with) PC.ae.C42.2 |
| 0.823693392 | 12.79240519 | PC.ae.C34.3 (interacts with) PC.ae.C42.4 |
| 0.823379163 | 50.61439964 | PC.ae.C38.4 (interacts with) PC.ae.C42.5 |
| 0.823374562 | 22.31766617 | PC.ae.C34.0 (interacts with) PC.ae.C38.1 |
| 0.823308978 | 30.93585699 | PC.aa.C38.4 (interacts with) SM.C18.1 |
| 0.823159356 | 21.04454386 | PC.ae.C32.2 (interacts with) PC.ae.C40.2 |
| 0.823137381 | 15.06033172 | PC.ae.C38.1 (interacts with) PC.ae.C40.1 |
| 0.823073889 | 10.78801457 | PC.aa.C38.4 (interacts with) SM.C24.0 |
| 0.823027993 | 15.74234959 | PC.aa.C38.4 (interacts with) PC.ae.C42.2 |
| 0.822948803 | 86.67791984 | PC.aa.C32.0 (interacts with) PC.aa.C36.1 |
| 0.822927547 | 17.86650277 | PC.aa.C42.5 (interacts with) PC.ae.C40.2 |
| 0.822881913 | 11.35953974 | lysoPC.a.C18.1 (interacts with) PC.aa.C34.1 |
| 0.822734484 | 176.7388438 | PC.ae.C40.3 (interacts with) PC.ae.C44.6 |
| 0.822649677 | 41.31224555 | PC.ae.C36.3 (interacts with) PC.ae.C38.5 |
| 0.822460765 | 10.26489241 | PC.aa.C40.6 (interacts with) PC.ae.C38.6 |
| 0.822281414 | 48.1974579 | PC.aa.C32.2 (interacts with) PC.ae.C30.0 |
| 0.82227173 | 137.7180287 | lysoPC.a.C18.2 (interacts with) PC.aa.C36.3 |
| 0.822231256 | 45.24749753 | PC.aa.C28.1 (interacts with) SM.C18.1 |
| 0.822181151 | 9.432781134 | lysoPC.a.C17.0 (interacts with) PC.aa.C28.1 |
| 0.821493311 | 57.14712195 | lysoPC.a.C18.0 (interacts with) SM.C24.0 |
| 0.821287698 | 13.92670378 | PC.ae.C36.4 (interacts with) PC.ae.C40.4 |
| 0.821091516 | 28.57078666 | PC.aa.C38.0 (interacts with) PC.ae.C40.3 |
| 0.820968861 | 15.32797718 | PC.ae.C40.6 (interacts with) SM.OH.C14.1 |
| 0.820923827 | 11.69117041 | PC.aa.C36.6 (interacts with) SM.OH.C22.1 |
| 0.820776437 | 53.26980914 | PC.aa.C38.3 (interacts with) PC.aa.C38.5 |
| 0.820768732 | 14.50659237 | PC.aa.C36.4 (interacts with) PC.ae.C36.4 |
| 0.820458755 | 47.7568015 | PC.ae.C34.1 (interacts with) SM.C16.1 |
| 0.820148439 | 5.584737539 | lysoPC.a.C18.1 (interacts with) PC.aa.C32.1 |
| 0.819984454 | 19.70207299 | PC.aa.C38.4 (interacts with) PC.ae.C38.3 |
| 0.819974474 | 29.01817042 | PC.ae.C40.3 (interacts with) PC.ae.C40.6 |
| 0.819875703 | 39.53417969 | PC.aa.C28.1 (interacts with) PC.aa.C34.2 |
| 0.819671127 | 8.771527554 | PC.ae.C36.2 (interacts with) SM.C24.0 |
| 0.819516596 | 84.76185117 | PC.aa.C40.4 (interacts with) PC.ae.C34.1 |
| 0.819487138 | 14.35765687 | PC.aa.C38.0 (interacts with) PC.aa.C40.6 |
| 0.819354448 | 33.12735979 | SM.OH.C22.2 (interacts with) SM.C24.0 |
| 0.819353542 | 23.38154342 | lysoPC.a.C16.0 (interacts with) PC.aa.C30.0 |
| 0.819312446 | 58.69776213 | PC.ae.C36.0 (interacts with) PC.ae.C42.3 |
| 0.819155003 | 6.925258147 | PC.aa.C32.0 (interacts with) PC.aa.C36.2 |
| 0.818885931 | 11.89851964 | PC.aa.C36.6 (interacts with) SM.C24.0 |
| 0.818716614 | 55.49691199 | PC.aa.C32.3 (interacts with) PC.ae.C42.3 |
| 0.818368948 | 17.93041198 | lysoPC.a.C17.0 (interacts with) PC.ae.C38.4 |
| 0.818292157 | 61.15659248 | SM.C16.0 (interacts with) SM.C24.1 |
| 0.818115991 | 18.24403154 | PC.ae.C40.6 (interacts with) SM.C16.1 |
| 0.817814327 | 54.33568519 | PC.ae.C40.5 (interacts with) PC.ae.C42.5 |
| 0.817776551 | 23.29913605 | PC.aa.C36.0 (interacts with) PC.ae.C40.2 |
| 0.817756548 | 27.21696825 | PC.ae.C36.1 (interacts with) PC.ae.C38.0 |
| 0.817726244 | 17.07512746 | PC.ae.C36.1 (interacts with) PC.ae.C36.5 |
| 0.817622727 | 28.89624024 | PC.ae.C34.0 (interacts with) PC.ae.C40.6 |
| 0.817468738 | 9.035334613 | PC.aa.C28.1 (interacts with) PC.ae.C40.4 |
| 0.817278533 | 28.74348075 | lysoPC.a.C18.0 (interacts with) PC.aa.C40.4 |
| 0.817151679 | 25.40924871 | PC.ae.C36.1 (interacts with) PC.ae.C40.4 |
| 0.817091602 | 10.98387914 | PC.aa.C32.2 (interacts with) PC.aa.C40.5 |
| 0.81695251 | 16.47599579 | PC.aa.C28.1 (interacts with) PC.ae.C34.3 |
| 0.816723137 | 98.87142133 | lysoPC.a.C18.0 (interacts with) PC.ae.C42.2 |
| 0.81668117 | 10.5200467 | PC.ae.C40.6 (interacts with) SM.C18.0 |
| 0.816504158 | 11.89047803 | PC.ae.C40.5 (interacts with) SM.C24.0 |
| 0.815480268 | 130.7578408 | lysoPC.a.C18.1 (interacts with) PC.aa.C36.3 |
| 0.815428767 | 26.3020667 | PC.aa.C38.1 (interacts with) PC.ae.C40.2 |
| 0.815404005 | 41.12200947 | PC.aa.C36.4 (interacts with) SM.C16.1 |
| 0.815203905 | 19.04200883 | PC.ae.C32.2 (interacts with) PC.ae.C36.1 |
| 0.815150662 | 26.1514376 | PC.aa.C34.4 (interacts with) PC.ae.C34.1 |
| 0.81511595 | 22.65957158 | PC.ae.C40.5 (interacts with) SM.C24.1 |
| 0.815111193 | 11.81908114 | PC.ae.C38.6 (interacts with) SM.C24.0 |
| 0.815098306 | 9.348440859 | PC.ae.C36.5 (interacts with) PC.ae.C40.3 |
| 0.8150127 | 28.85969309 | PC.aa.C38.5 (interacts with) PC.ae.C42.3 |
| 0.81488579 | 20.83318413 | PC.ae.C38.5 (interacts with) PC.ae.C40.2 |
| 0.814823332 | 34.00900766 | PC.aa.C32.1 (interacts with) PC.aa.C40.4 |
| 0.814770552 | 11.08814758 | PC.ae.C32.1 (interacts with) SM.OH.C16.1 |
| 0.814554066 | 22.87213147 | PC.ae.C40.6 (interacts with) SM.C16.0 |
| 0.814395744 | 9.527319314 | PC.aa.C32.0 (interacts with) PC.ae.C36.3 |
| 0.81434841 | 27.28933861 | PC.ae.C38.3 (interacts with) PC.ae.C38.6 |
| 0.81432166 | 31.45376511 | PC.ae.C40.1 (interacts with) PC.ae.C40.5 |
| 0.81415763 | 664 | Ala (interacts with) Ile |
| 0.814139824 | 11.86831355 | PC.aa.C42.1 (interacts with) PC.ae.C44.5 |
| 0.814090934 | 110.88282 | PC.ae.C42.4 (interacts with) PC.ae.C44.6 |
| 0.814047558 | 90.47659475 | PC.ae.C38.2 (interacts with) PC.ae.C42.3 |
| 0.814047532 | 86 | Leu (interacts with) Trp |
| 0.814013895 | 48.00182304 | PC.ae.C38.4 (interacts with) SM.C18.1 |
| 0.813704337 | 34.19093136 | PC.ae.C36.1 (interacts with) PC.ae.C38.1 |
| 0.813551635 | 63.77418121 | PC.aa.C32.0 (interacts with) PC.aa.C42.5 |
| 0.813510208 | 13.30776046 | PC.aa.C42.0 (interacts with) SM.OH.C14.1 |
| 0.813464463 | 78.60941543 | PC.aa.C32.0 (interacts with) PC.aa.C36.3 |
| 0.813242666 | 14.37995741 | PC.aa.C34.2 (interacts with) PC.ae.C36.4 |
| 0.813204672 | 19.57318728 | PC.ae.C38.4 (interacts with) PC.ae.C38.6 |
| 0.813163903 | 9.186852496 | SM.OH.C16.1 (interacts with) SM.C24.0 |
| 0.813160483 | 21.93950329 | PC.ae.C38.5 (interacts with) PC.ae.C40.6 |
| 0.813066397 | 41.40881637 | PC.ae.C36.2 (interacts with) SM.OH.C22.2 |
| 0.813025557 | 7.569547347 | PC.ae.C40.5 (interacts with) SM.OH.C24.1 |
| 0.812999458 | 79.71094026 | PC.aa.C38.3 (interacts with) PC.ae.C36.1 |
| 0.812864742 | 38.5201412 | PC.ae.C40.6 (interacts with) PC.ae.C42.2 |
| 0.812725626 | 160.1316865 | PC.aa.C42.1 (interacts with) PC.ae.C44.6 |
| 0.812568245 | 21.170278 | PC.ae.C38.1 (interacts with) PC.ae.C40.4 |
| 0.812485961 | 40.82649695 | PC.ae.C40.2 (interacts with) PC.ae.C42.2 |
| 0.812268439 | 10.18054668 | PC.aa.C42.5 (interacts with) PC.ae.C38.5 |
| 0.811639371 | 38.442649 | PC.aa.C42.0 (interacts with) PC.ae.C42.2 |
| 0.811592201 | 19.95862352 | PC.ae.C40.4 (interacts with) PC.ae.C42.4 |
| 0.811363835 | 8.621572811 | PC.aa.C36.6 (interacts with) PC.aa.C42.5 |
| 0.811246737 | 9.25634767 | lysoPC.a.C17.0 (interacts with) PC.aa.C42.5 |
| 0.811145031 | 45.87367703 | lysoPC.a.C18.0 (interacts with) PC.aa.C36.3 |
| 0.8111448 | 46.69371325 | PC.aa.C34.1 (interacts with) PC.aa.C34.4 |
| 0.810948729 | 17.58908135 | PC.aa.C42.5 (interacts with) PC.ae.C40.6 |
| 0.809959871 | 14.07903038 | PC.aa.C38.4 (interacts with) PC.aa.C38.5 |
| 0.809912776 | 19.61785724 | PC.ae.C38.0 (interacts with) PC.ae.C38.3 |
| 0.809907371 | 33.85837702 | PC.aa.C32.3 (interacts with) PC.ae.C38.4 |
| 0.809554824 | 99.9768323 | PC.aa.C36.3 (interacts with) PC.ae.C36.3 |
| 0.809456772 | 60.5678761 | PC.ae.C38.3 (interacts with) SM.OH.C22.2 |
| 0.809300529 | 19.29210135 | PC.aa.C36.2 (interacts with) PC.ae.C40.1 |
| 0.809164462 | 11.95210208 | PC.aa.C32.0 (interacts with) PC.ae.C34.3 |
| 0.809114117 | 26.60314448 | PC.ae.C38.1 (interacts with) PC.ae.C40.5 |
| 0.809113653 | 30.12170349 | PC.aa.C42.6 (interacts with) PC.ae.C40.6 |
| 0.809077191 | 28.30243761 | PC.aa.C40.6 (interacts with) PC.ae.C42.2 |
| 0.809026054 | 56.03235244 | PC.aa.C32.3 (interacts with) PC.ae.C38.2 |
| 0.80882408 | 102.9640635 | PC.aa.C32.0 (interacts with) SM.C16.0 |
| 0.808801739 | 32.50858453 | PC.aa.C32.2 (interacts with) PC.ae.C34.3 |
| 0.808773356 | 21.24321513 | PC.ae.C30.0 (interacts with) PC.ae.C40.4 |
| 0.808702318 | 26.97107392 | PC.ae.C42.4 (interacts with) SM.C16.0 |
| 0.808207022 | 15.42584706 | PC.ae.C40.6 (interacts with) SM.OH.C24.1 |
| 0.807992779 | 18.46405814 | PC.ae.C36.0 (interacts with) PC.ae.C40.2 |
| 0.807710078 | 14.64255823 | PC.aa.C36.2 (interacts with) PC.ae.C36.4 |
| 0.807625764 | 12.81994977 | PC.aa.C32.0 (interacts with) PC.aa.C36.4 |
| 0.807547027 | 6.892279904 | PC.ae.C36.5 (interacts with) SM.OH.C14.1 |
| 0.807481131 | 135.676555 | PC.aa.C38.5 (interacts with) PC.aa.C40.5 |
| 0.807348242 | 47.4846305 | PC.aa.C38.1 (interacts with) PC.ae.C40.5 |
| 0.807271025 | 17.40786967 | PC.aa.C38.0 (interacts with) SM.OH.C14.1 |
| 0.807105941 | 43.32412985 | lysoPC.a.C18.0 (interacts with) PC.aa.C36.1 |
| 0.806926185 | 18.69771619 | PC.aa.C38.4 (interacts with) PC.ae.C38.5 |
| 0.806490829 | 27.58801213 | lysoPC.a.C20.3 (interacts with) PC.aa.C34.3 |
| 0.806382764 | 13.57802808 | PC.aa.C28.1 (interacts with) PC.ae.C40.1 |
| 0.806355187 | 9.732812469 | PC.ae.C32.1 (interacts with) SM.OH.C24.1 |
| 0.806311662 | 6.309688747 | PC.ae.C40.5 (interacts with) SM.OH.C14.1 |
| 0.806270926 | 20.58297844 | PC.aa.C42.0 (interacts with) PC.ae.C38.4 |
| 0.806123773 | 28.68179576 | PC.aa.C32.1 (interacts with) PC.aa.C34.3 |
| 0.805830692 | 22.53288509 | PC.aa.C32.3 (interacts with) PC.ae.C32.1 |
| 0.805616095 | 41.2872504 | PC.aa.C32.1 (interacts with) PC.aa.C40.5 |
| 0.805481841 | 28.32907402 | PC.ae.C34.0 (interacts with) PC.ae.C40.2 |
| 0.805380614 | 74.89201623 | PC.aa.C32.0 (interacts with) PC.ae.C42.4 |
| 0.80537068 | 14.54904023 | PC.ae.C36.4 (interacts with) SM.OH.C24.1 |
| 0.805008511 | 36.32983904 | PC.ae.C36.1 (interacts with) PC.ae.C40.3 |
| 0.80499195 | 8.917680977 | SM.OH.C14.1 (interacts with) SM.C24.0 |
| 0.804848189 | 19.92245164 | PC.aa.C32.3 (interacts with) PC.aa.C38.3 |
| 0.804792827 | 130.4710581 | PC.aa.C36.3 (interacts with) PC.ae.C34.1 |
| 0.804756262 | 8.13502734 | PC.aa.C36.0 (interacts with) SM.C24.1 |
| 0.804705601 | 17.8066864 | PC.aa.C42.0 (interacts with) SM.C16.1 |
| 0.804652988 | 12.89324093 | lysoPC.a.C18.0 (interacts with) PC.aa.C34.2 |
| 0.804639264 | 45.28759046 | PC.aa.C36.4 (interacts with) SM.OH.C24.1 |
| 0.804612628 | 15.97109226 | PC.ae.C36.2 (interacts with) PC.ae.C38.0 |
| 0.804496229 | 45.25652943 | PC.ae.C36.1 (interacts with) PC.ae.C40.5 |
| 0.804473995 | 99.69223704 | lysoPC.a.C16.0 (interacts with) lysoPC.a.C16.1 |
| 0.804342113 | 15.69618415 | PC.ae.C38.4 (interacts with) PC.ae.C40.5 |
| 0.804195869 | 31.65602602 | PC.aa.C32.3 (interacts with) SM.C24.0 |
| 0.804166921 | 11.54040251 | PC.ae.C38.4 (interacts with) SM.C24.0 |
| 0.80411902 | 12.9187005 | PC.aa.C38.6 (interacts with) PC.ae.C42.3 |
| 0.80405644 | 40.70963514 | PC.ae.C38.4 (interacts with) SM.OH.C22.2 |
| 0.803367188 | 8.975078505 | PC.aa.C38.0 (interacts with) SM.C18.0 |
| 0.803331742 | 820 | Ala (interacts with) Pro |
| 0.803278273 | 21.73721455 | PC.aa.C34.2 (interacts with) PC.ae.C36.1 |
| 0.803178079 | 23.48332333 | PC.ae.C30.0 (interacts with) PC.ae.C40.3 |
| 0.803130051 | 39.10576641 | PC.ae.C32.2 (interacts with) PC.ae.C36.3 |
| 0.802980976 | 30.42614905 | PC.ae.C42.5 (interacts with) SM.OH.C16.1 |
| 0.802761974 | 19.55183517 | PC.ae.C32.2 (interacts with) PC.ae.C34.3 |
| 0.802761074 | 29.00500566 | PC.aa.C38.3 (interacts with) PC.ae.C34.1 |
| 0.802700484 | 49.71303564 | PC.ae.C34.1 (interacts with) PC.ae.C42.3 |
| 0.802250636 | 34.71089811 | lysoPC.a.C17.0 (interacts with) PC.aa.C34.4 |
| 0.802141736 | 16.93000129 | PC.aa.C38.0 (interacts with) SM.OH.C24.1 |
| 0.802117855 | 9.453689365 | PC.aa.C38.6 (interacts with) SM.OH.C14.1 |
| 0.80181651 | 47.52792251 | PC.aa.C28.1 (interacts with) PC.ae.C40.2 |
| 0.801469105 | 25.79310436 | PC.aa.C42.6 (interacts with) SM.OH.C22.2 |
| 0.801438912 | 56.42235166 | PC.aa.C32.0 (interacts with) PC.aa.C34.3 |
| 0.801297389 | 6.558632025 | PC.ae.C30.0 (interacts with) PC.ae.C36.4 |
| 0.801229456 | 11.28125314 | PC.aa.C38.6 (interacts with) PC.ae.C34.0 |
| 0.800963468 | 7.889972956 | PC.ae.C40.5 (interacts with) SM.C16.1 |
| 0.800811477 | 29.35803267 | PC.ae.C42.5 (interacts with) SM.OH.C14.1 |
| 0.80072448 | 17.92328257 | PC.aa.C36.6 (interacts with) SM.OH.C22.2 |
| 0.800629964 | 24.75663121 | PC.aa.C42.0 (interacts with) PC.ae.C40.5 |
| 0.800423755 | 13.90705447 | PC.aa.C38.6 (interacts with) SM.C16.0 |
| 0.800249514 | 13.4388994 | PC.ae.C36.4 (interacts with) PC.ae.C40.3 |
| -0.81113016 | 138.5238816 | Pro (interacts with) PC.aa.C38.0 |
| -0.821603135 | 358.1938972 | Pro (interacts with) SM.OH.C22.2 |
| -0.821917195 | 205.8362527 | Pro (interacts with) PC.ae.C40.6 |
| -0.826394092 | 218.1073597 | Pro (interacts with) PC.ae.C40.2 |
| -0.83366313 | 51.33860877 | Pro (interacts with) SM.C24.1 |
| -0.847293481 | 172 | Kynurenine (interacts with) SM.C18.1 |

Table 11. Network parameters for early adenocarcinoma group

| Average Shortest  PathLength | Betweenness Centrality | Closeness Centrality | Clustering Coefficient | Degree | Eccentricity | Metabolite | Neighborhood Connectivity | Number Of  Undirected Edges |
| --- | --- | --- | --- | --- | --- | --- | --- | --- |
| 2.3 | 0.005014173 | 0.43478261 | 0.72058824 | 17 | 6 | PC.aa.C38.0 | 25.88235294 | 17 |
| 1.9625 | 0.019682618 | 0.50955414 | 0.63793103 | 29 | 5 | PC.ae.C38.6 | 26.72413793 | 29 |
| 1.7625 | 0.012308069 | 0.56737589 | 0.67914439 | 34 | 5 | SM.OH.C14.1 | 31.35294118 | 34 |
| 1.7 | 0.024468617 | 0.58823529 | 0.62462462 | 37 | 5 | SM.OH.C16.1 | 30.24324324 | 37 |
| 2.15 | 0.003241673 | 0.46511628 | 0.81045752 | 18 | 4 | PC.aa.C34.1 | 25.27777778 | 18 |
| 2.1 | 0.014568166 | 0.47619048 | 0.73333333 | 21 | 4 | PC.aa.C36.1 | 24.14285714 | 21 |
| 2.025 | 0.008095621 | 0.49382716 | 0.71541502 | 23 | 4 | PC.aa.C34.2 | 27.60869565 | 23 |
| 1.825 | 0.017125251 | 0.54794521 | 0.6773399 | 29 | 4 | PC.aa.C36.2 | 28.06896552 | 29 |
| 2.0125 | 0.019114734 | 0.49689441 | 0.73333333 | 21 | 4 | lysoPC.a.C16.0 | 24.23809524 | 21 |
| 1.8625 | 0.016013155 | 0.53691275 | 0.63666667 | 25 | 4 | lysoPC.a.C18.0 | 25.96 | 25 |
| 1.775 | 0.012127925 | 0.56338028 | 0.67241379 | 29 | 4 | PC.ae.C34.3 | 31.5862069 | 29 |
| 1.8 | 0.010785235 | 0.55555556 | 0.66951567 | 27 | 4 | PC.ae.C36.3 | 30.51851852 | 27 |
| 1 | 0.055555556 | 1 | 0.83333333 | 4 | 1 | C12.1 | 3.5 | 4 |
| 1 | 0.055555556 | 1 | 0.83333333 | 4 | 1 | C14.1 | 3.5 | 4 |
| 1.8625 | 0.004112005 | 0.53691275 | 0.77230769 | 26 | 4 | PC.ae.C34.2 | 32.80769231 | 26 |
| 1.8125 | 0.009124218 | 0.55172414 | 0.67943548 | 32 | 5 | PC.ae.C36.5 | 30.78125 | 32 |
| 1.8375 | 0.015958275 | 0.54421769 | 0.68505747 | 30 | 5 | PC.ae.C38.5 | 29.46666667 | 30 |
| 1.5 | 0 | 0.66666667 | 0 | 1 | 2 | Creatinine | 2 | 1 |
| 1 | 1 | 1 | 0 | 2 | 1 | total.DMA | 1 | 2 |
| 2 | 0.01601641 | 0.5 | 0.65811966 | 27 | 5 | PC.ae.C40.6 | 27.2962963 | 27 |
| 1.975 | 0.007953382 | 0.50632911 | 0.68783069 | 28 | 5 | PC.ae.C40.2 | 28.5 | 28 |
| 1.975 | 0.008575758 | 0.50632911 | 0.67487685 | 29 | 5 | SM.OH.C22.2 | 28.24137931 | 29 |
| 2.1625 | 0.001648949 | 0.46242775 | 0.84558824 | 17 | 5 | PC.aa.C36.5 | 28.47058824 | 17 |
| 1.9375 | 0.004656437 | 0.51612903 | 0.74736842 | 20 | 4 | PC.aa.C38.5 | 29.35 | 20 |
| 2.25 | 3.67E-04 | 0.44444444 | 0.75 | 8 | 4 | PC.aa.C36.4 | 26.25 | 8 |
| 2.075 | 0.001673318 | 0.48192771 | 0.68181818 | 12 | 4 | PC.aa.C38.4 | 31.33333333 | 12 |
| 1.6 | 0.029361313 | 0.625 | 0.56146179 | 43 | 4 | PC.ae.C42.2 | 29.95348837 | 43 |
| 1.6125 | 0.029605912 | 0.62015504 | 0.57026713 | 42 | 4 | SM.OH.C22.1 | 30.9047619 | 42 |
| 2.075 | 0.007135879 | 0.48192771 | 0.71861472 | 22 | 4 | PC.aa.C34.3 | 24.22727273 | 22 |
| 1.925 | 0.037298163 | 0.51948052 | 0.63532764 | 27 | 4 | PC.aa.C36.3 | 24.62962963 | 27 |
| 1 | 0.055555556 | 1 | 0.83333333 | 4 | 1 | C14.2 | 3.5 | 4 |
| 1.825 | 0.017447002 | 0.54794521 | 0.63817664 | 27 | 4 | PC.aa.C38.3 | 27 | 27 |
| 1.7625 | 0.041479044 | 0.56737589 | 0.5655914 | 31 | 4 | PC.aa.C40.5 | 27.4516129 | 31 |
| 1.7125 | 0.0132262 | 0.58394161 | 0.64201681 | 35 | 4 | PC.aa.C28.1 | 32.25714286 | 35 |
| 1.725 | 0.018240057 | 0.57971014 | 0.64314516 | 32 | 4 | PC.ae.C30.0 | 31.96875 | 32 |
| 1.6875 | 0.021412007 | 0.59259259 | 0.59159159 | 37 | 4 | PC.aa.C32.0 | 31.27027027 | 37 |
| 1.6375 | 0.032171323 | 0.61068702 | 0.5802969 | 39 | 4 | PC.ae.C36.1 | 30.28205128 | 39 |
| 1.5375 | 0.061542584 | 0.6504065 | 0.52929293 | 45 | 4 | PC.ae.C38.3 | 30.35555556 | 45 |
| 1.9 | 0.016770808 | 0.52631579 | 0.63054187 | 29 | 4 | PC.aa.C40.4 | 25.68965517 | 29 |
| 1.7125 | 0.02137291 | 0.58394161 | 0.62698413 | 36 | 5 | SM.C16.0 | 30.11111111 | 36 |
| 1.575 | 0.035173486 | 0.63492063 | 0.55073996 | 44 | 4 | PC.ae.C34.0 | 30.40909091 | 44 |
| 2.1125 | 0.006997855 | 0.47337278 | 0.73160173 | 22 | 4 | PC.aa.C32.2 | 24.63636364 | 22 |
| 1.975 | 0.008814842 | 0.50632911 | 0.68660969 | 27 | 5 | PC.aa.C38.6 | 28.14814815 | 27 |
| 1.9375 | 0.00379615 | 0.51612903 | 0.77192982 | 19 | 4 | PC.aa.C40.6 | 30.52631579 | 19 |
| 1.7375 | 0.016244559 | 0.57553957 | 0.63361345 | 35 | 5 | SM.C16.1 | 30.4 | 35 |
| 1.6875 | 0.025050919 | 0.59259259 | 0.61008403 | 35 | 4 | PC.ae.C34.1 | 30.94285714 | 35 |
| 1.825 | 0.017723242 | 0.54794521 | 0.59259259 | 27 | 4 | PC.aa.C42.6 | 29.48148148 | 27 |
| 1.7375 | 0.012680161 | 0.57553957 | 0.67096774 | 31 | 4 | PC.ae.C36.2 | 32.35483871 | 31 |
| 1.85 | 0.010377782 | 0.54054054 | 0.65333333 | 25 | 4 | lysoPC.a.C17.0 | 31 | 25 |
| 1.5625 | 0.056861914 | 0.64 | 0.51010101 | 45 | 4 | PC.ae.C42.3 | 29.31111111 | 45 |
| 2.4625 | 0.032765151 | 0.40609137 | 0.65151515 | 12 | 6 | PC.ae.C42.5 | 23.66666667 | 12 |
| 3.225 | 5.84E-04 | 0.31007752 | 0.66666667 | 3 | 7 | PC.ae.C44.6 | 10.33333333 | 3 |
| 2.3 | 0.017653797 | 0.43478261 | 0.72058824 | 17 | 6 | PC.aa.C42.0 | 26.23529412 | 17 |
| 2.6125 | 9.27E-05 | 0.38277512 | 0.96428571 | 8 | 5 | PC.aa.C32.1 | 22.25 | 8 |
| 2.275 | 0.001621933 | 0.43956044 | 0.77777778 | 9 | 5 | PC.aa.C36.6 | 24 | 9 |
| 1.95 | 0.011960839 | 0.51282051 | 0.64492754 | 24 | 5 | PC.ae.C38.0 | 27.54166667 | 24 |
| 1.8625 | 0.01094657 | 0.53691275 | 0.72486772 | 28 | 5 | PC.ae.C32.1 | 31.03571429 | 28 |
| 1.975 | 0.003723462 | 0.50632911 | 0.8 | 20 | 5 | PC.ae.C36.4 | 31.2 | 20 |
| 1.8 | 0.01280788 | 0.55555556 | 0.68145161 | 32 | 5 | PC.ae.C38.4 | 31.8125 | 32 |
| 2.2 | 0.001810229 | 0.45454545 | 0.81617647 | 17 | 5 | SM.C18.0 | 29.47058824 | 17 |
| 2.05 | 0.004426682 | 0.48780488 | 0.76842105 | 20 | 4 | PC.aa.C34.4 | 25 | 20 |
| 1.7 | 0.019585053 | 0.58823529 | 0.56506239 | 34 | 4 | SM.C24.0 | 30.41176471 | 34 |
| 1.8125 | 0.014154363 | 0.55172414 | 0.64731183 | 31 | 4 | PC.aa.C32.3 | 29.22580645 | 31 |
| 1.85 | 0.00925412 | 0.54054054 | 0.70935961 | 29 | 5 | PC.ae.C40.3 | 30.89655172 | 29 |
| 1.775 | 0.017443846 | 0.56338028 | 0.66935484 | 32 | 5 | PC.ae.C40.4 | 31 | 32 |
| 2.275 | 2.05E-04 | 0.43956044 | 0.93406593 | 14 | 5 | SM.C18.1 | 30.5 | 14 |
| 1.8625 | 0.01179827 | 0.53691275 | 0.65747126 | 30 | 5 | PC.ae.C40.5 | 29.03333333 | 30 |
| 1.7875 | 0.013431459 | 0.55944056 | 0.67045455 | 33 | 5 | PC.ae.C32.2 | 30.3030303 | 33 |
| 2.5875 | 0.00177798 | 0.38647343 | 0.70909091 | 11 | 6 | PC.aa.C36.0 | 20.36363636 | 11 |
| 3.4375 | 0 | 0.29090909 | 1 | 2 | 7 | PC.ae.C44.5 | 7.5 | 2 |
| 2.875 | 0 | 0.34782609 | 1 | 5 | 6 | PC.aa.C38.1 | 18.8 | 5 |
| 1.9125 | 0.010026901 | 0.52287582 | 0.72333333 | 25 | 4 | PC.aa.C30.0 | 28.44 | 25 |
| 1.8125 | 0.006288018 | 0.55172414 | 0.69655172 | 30 | 5 | PC.aa.C42.5 | 32.5 | 30 |
| 2.25 | 1.25E-04 | 0.44444444 | 0.85714286 | 8 | 5 | PC.ae.C36.0 | 32.125 | 8 |
| 1 | 0 | 1 | 1 | 2 | 1 | Leu | 2 | 2 |
| 1 | 0 | 1 | 1 | 2 | 1 | Val | 2 | 2 |
| 2.0875 | 0.003844488 | 0.47904192 | 0.81617647 | 17 | 5 | PC.ae.C42.4 | 29.70588235 | 17 |
| 1.8875 | 0.018061747 | 0.52980132 | 0.59288538 | 23 | 4 | PC.ae.C40.1 | 28.26086957 | 23 |
| 1.8375 | 0.004445189 | 0.54421769 | 0.73333333 | 30 | 5 | SM.OH.C24.1 | 32.56666667 | 30 |
| 1.25 | 0 | 0.8 | 1 | 3 | 2 | C2 | 4 | 3 |
| 3.3875 | 0 | 0.29520295 | 1 | 2 | 6 | lysoPC.a.C16.1 | 9 | 2 |
| 3.3125 | 0.02525043 | 0.30188679 | 0.33333333 | 4 | 6 | lysoPC.a.C18.1 | 6.5 | 4 |
| 2.5875 | 8.82E-04 | 0.38647343 | 0.77777778 | 10 | 6 | SM.C24.1 | 22 | 10 |
| 2.425 | 0.057594206 | 0.41237113 | 0.62637363 | 14 | 5 | lysoPC.a.C20.3 | 19 | 14 |
| 1.25 | 0 | 0.8 | 1 | 3 | 2 | C10.1 | 4 | 3 |
| 2.5875 | 0.015605671 | 0.38647343 | 0.72222222 | 9 | 5 | lysoPC.a.C18.2 | 20.22222222 | 9 |
| 2.35 | 0 | 0.42553191 | 1 | 3 | 5 | PC.ae.C38.2 | 33 | 3 |
| 1 | 1 | 1 | 0 | 2 | 1 | C14 | 1 | 2 |
| 1.5 | 0 | 0.66666667 | 0 | 1 | 2 | C18.1 | 2 | 1 |
| 1 | 0 | 1 | 1 | 2 | 1 | Ile | 2 | 2 |
| 1.666666667 | 0 | 0.6 | 0 | 1 | 2 | Arg | 3 | 1 |
| 1 | 1 | 1 | 0 | 3 | 1 | Met | 1 | 3 |
| 1.666666667 | 0 | 0.6 | 0 | 1 | 2 | Thr | 3 | 1 |
| 3.85 | 0 | 0.25974026 | 0 | 1 | 6 | C3 | 4 | 1 |
| 2.8625 | 0.025 | 0.34934498 | 0.5 | 4 | 5 | Trp | 17.5 | 4 |
| 1.5 | 0 | 0.66666667 | 0 | 1 | 2 | Cit | 2 | 1 |
| 1.5 | 0 | 0.66666667 | 0 | 1 | 2 | C0 | 2 | 1 |
| 1 | 1 | 1 | 0 | 2 | 1 | Lys | 1 | 2 |
| 1.5 | 0 | 0.66666667 | 0 | 1 | 2 | His | 2 | 1 |
| 1.5 | 0 | 0.66666667 | 0 | 1 | 2 | C18.2 | 2 | 1 |
| 4.3 | 0 | 0.23255814 | 0 | 1 | 7 | lysoPC.a.C20.4 | 4 | 1 |
| 1.666666667 | 0 | 0.6 | 0 | 1 | 2 | Tyr | 3 | 1 |

Supplementary Table 12. Correlation coefficient of nodes from Early lung adenocarcinoma group

| Correlation coefficient | Edge Betweenness | Metabolites |
| --- | --- | --- |
| 0.985076621 | 5.405586705 | PC.aa.C38.0 (interacts with) PC.ae.C38.6 |
| 0.968838217 | 5.875283675 | PC.aa.C38.0 (interacts with) PC.ae.C40.6 |
| 0.910637919 | 6.444240017 | PC.aa.C38.0 (interacts with) PC.ae.C40.2 |
| 0.908164685 | 7.056902804 | PC.aa.C38.0 (interacts with) PC.aa.C38.6 |
| 0.90716969 | 6.601429487 | PC.aa.C38.0 (interacts with) SM.OH.C22.2 |
| 0.900357172 | 10.24315362 | PC.aa.C38.0 (interacts with) PC.ae.C38.5 |
| 0.8964479 | 5.790717848 | PC.aa.C38.0 (interacts with) SM.C24.1 |
| 0.885380847 | 10.2090487 | PC.aa.C38.0 (interacts with) PC.ae.C40.5 |
| 0.879604243 | 12.48664748 | PC.aa.C38.0 (interacts with) PC.ae.C36.5 |
| 0.872122663 | 15.69234481 | PC.aa.C38.0 (interacts with) PC.ae.C32.2 |
| 0.844900645 | 22.30619048 | PC.aa.C38.0 (interacts with) SM.C16.1 |
| 0.839869832 | 20.27293975 | PC.aa.C38.0 (interacts with) PC.aa.C38.1 |
| 0.825189421 | 14.39708558 | PC.aa.C38.0 (interacts with) PC.aa.C42.0 |
| 0.822271407 | 19.77822151 | PC.aa.C38.0 (interacts with) PC.ae.C38.0 |
| 0.810973773 | 23.42044436 | PC.aa.C38.0 (interacts with) SM.C16.0 |
| 0.806808028 | 28.07139408 | PC.aa.C38.0 (interacts with) SM.OH.C16.1 |
| 0.964822239 | 2.954711955 | PC.ae.C38.6 (interacts with) PC.ae.C40.6 |
| 0.931799638 | 4.020013518 | PC.ae.C38.6 (interacts with) PC.ae.C40.2 |
| 0.923113604 | 2.972562358 | PC.ae.C38.6 (interacts with) SM.OH.C22.2 |
| 0.909498214 | 5.137874846 | PC.ae.C38.6 (interacts with) PC.ae.C40.5 |
| 0.887721928 | 16.3659585 | PC.ae.C38.6 (interacts with) SM.C24.1 |
| 0.886265777 | 10.13550093 | PC.ae.C38.6 (interacts with) SM.C16.1 |
| 0.856854464 | 11.67756774 | PC.ae.C38.6 (interacts with) SM.C16.0 |
| 0.843399532 | 12.95807618 | PC.ae.C38.6 (interacts with) SM.OH.C16.1 |
| 0.833972185 | 17.21211654 | PC.ae.C38.6 (interacts with) PC.ae.C42.2 |
| 0.826198754 | 21.37903473 | PC.ae.C38.6 (interacts with) SM.OH.C22.1 |
| 0.810100701 | 11.10702034 | PC.ae.C38.6 (interacts with) PC.ae.C40.3 |
| 0.806730036 | 7.32793187 | PC.ae.C38.6 (interacts with) SM.C18.0 |
| 0.806275617 | 34.28627205 | PC.ae.C38.6 (interacts with) PC.ae.C42.3 |
| 0.804624364 | 9.357113712 | PC.ae.C38.6 (interacts with) SM.C18.1 |
| 0.984720972 | 3.330484256 | SM.OH.C14.1 (interacts with) SM.OH.C16.1 |
| 0.93687798 | 3.669478802 | SM.OH.C14.1 (interacts with) SM.C16.0 |
| 0.918535709 | 4.216021015 | SM.OH.C14.1 (interacts with) SM.C16.1 |
| 0.91683899 | 6.387071134 | SM.OH.C14.1 (interacts with) SM.OH.C22.1 |
| 0.880778313 | 11.65789751 | SM.OH.C14.1 (interacts with) SM.OH.C22.2 |
| 0.871066113 | 13.44705334 | SM.OH.C14.1 (interacts with) SM.C18.0 |
| 0.870044085 | 3.51385918 | SM.OH.C14.1 (interacts with) SM.OH.C24.1 |
| 0.819760367 | 18.67120536 | SM.OH.C14.1 (interacts with) SM.C18.1 |
| 0.958323534 | 2.317460317 | SM.OH.C16.1 (interacts with) SM.C16.0 |
| 0.941885756 | 3.709942048 | SM.OH.C16.1 (interacts with) SM.C16.1 |
| 0.924523238 | 9.347381479 | SM.OH.C16.1 (interacts with) SM.OH.C22.2 |
| 0.924362733 | 7.555921496 | SM.OH.C16.1 (interacts with) SM.OH.C22.1 |
| 0.914671853 | 11.24227713 | SM.OH.C16.1 (interacts with) SM.C18.0 |
| 0.882174631 | 5.396306613 | SM.OH.C16.1 (interacts with) SM.OH.C24.1 |
| 0.871406554 | 15.07698257 | SM.OH.C16.1 (interacts with) SM.C18.1 |
| 0.984659025 | 4.262951846 | PC.aa.C34.1 (interacts with) PC.aa.C36.1 |
| 0.927762731 | 5.791690884 | PC.aa.C34.1 (interacts with) PC.aa.C36.3 |
| 0.924000782 | 5.00897733 | PC.aa.C34.1 (interacts with) PC.aa.C40.4 |
| 0.9014844 | 3.433595234 | PC.aa.C34.1 (interacts with) PC.aa.C34.3 |
| 0.899084713 | 7.138520237 | PC.aa.C34.1 (interacts with) PC.aa.C38.3 |
| 0.897969808 | 13.86239121 | PC.aa.C34.1 (interacts with) PC.aa.C40.5 |
| 0.889308627 | 7.171688888 | PC.aa.C34.1 (interacts with) PC.aa.C36.2 |
| 0.862082591 | 26.22811123 | PC.aa.C34.1 (interacts with) PC.ae.C34.1 |
| 0.85111367 | 5.315801205 | PC.aa.C34.1 (interacts with) PC.aa.C34.2 |
| 0.849587205 | 4.725817869 | PC.aa.C34.1 (interacts with) PC.aa.C34.4 |
| 0.822878073 | 23.94768052 | PC.aa.C34.1 (interacts with) PC.ae.C36.1 |
| 0.942176683 | 3.440406462 | PC.aa.C36.1 (interacts with) PC.aa.C36.3 |
| 0.92788574 | 6.779058945 | PC.aa.C36.1 (interacts with) PC.aa.C40.4 |
| 0.911239913 | 9.31937942 | PC.aa.C36.1 (interacts with) PC.aa.C38.3 |
| 0.903882132 | 18.39012371 | PC.aa.C36.1 (interacts with) PC.aa.C40.5 |
| 0.898982745 | 8.453649863 | PC.aa.C36.1 (interacts with) PC.aa.C36.2 |
| 0.855817495 | 34.48578559 | PC.aa.C36.1 (interacts with) PC.ae.C34.1 |
| 0.84806667 | 32.54835982 | PC.aa.C36.1 (interacts with) PC.ae.C36.1 |
| 0.801858271 | 36.72714104 | PC.aa.C36.1 (interacts with) PC.ae.C30.0 |
| 0.983101516 | 3.293210418 | PC.aa.C34.2 (interacts with) PC.aa.C36.2 |
| 0.941800049 | 9.517707042 | PC.aa.C34.2 (interacts with) PC.ae.C34.3 |
| 0.927133349 | 8.440872712 | PC.aa.C34.2 (interacts with) PC.ae.C36.3 |
| 0.909547227 | 4.669344097 | PC.aa.C34.2 (interacts with) PC.aa.C34.3 |
| 0.908944565 | 5.666112499 | PC.aa.C34.2 (interacts with) PC.aa.C36.3 |
| 0.904317305 | 14.77898478 | PC.aa.C34.2 (interacts with) PC.ae.C36.2 |
| 0.90290572 | 18.65167094 | PC.aa.C34.2 (interacts with) PC.ae.C34.2 |
| 0.873731961 | 11.09806557 | PC.aa.C34.2 (interacts with) PC.ae.C30.0 |
| 0.868511473 | 10.37722651 | PC.aa.C34.2 (interacts with) PC.ae.C34.1 |
| 0.85019985 | 5.420037001 | PC.aa.C34.2 (interacts with) PC.aa.C40.4 |
| 0.843400733 | 10.13224471 | PC.aa.C34.2 (interacts with) PC.ae.C36.1 |
| 0.839341884 | 7.013359905 | PC.aa.C34.2 (interacts with) PC.aa.C36.1 |
| 0.838961413 | 31.43973033 | PC.aa.C34.2 (interacts with) PC.ae.C38.3 |
| 0.80695234 | 5.19136944 | PC.aa.C34.2 (interacts with) PC.aa.C34.4 |
| 0.952649298 | 5.983239277 | PC.aa.C36.2 (interacts with) PC.aa.C36.3 |
| 0.936166299 | 8.277863454 | PC.aa.C36.2 (interacts with) PC.ae.C34.3 |
| 0.932465655 | 7.792760224 | PC.aa.C36.2 (interacts with) PC.ae.C36.3 |
| 0.917086755 | 11.81384801 | PC.aa.C36.2 (interacts with) PC.ae.C36.2 |
| 0.903277268 | 4.267189934 | PC.aa.C36.2 (interacts with) PC.aa.C40.4 |
| 0.88568107 | 8.63078344 | PC.aa.C36.2 (interacts with) PC.ae.C30.0 |
| 0.885429269 | 7.687911091 | PC.aa.C36.2 (interacts with) PC.ae.C34.1 |
| 0.883502021 | 6.453750084 | PC.aa.C36.2 (interacts with) PC.ae.C36.1 |
| 0.882882155 | 17.45506936 | PC.aa.C36.2 (interacts with) PC.ae.C34.2 |
| 0.876675018 | 18.06411718 | PC.aa.C36.2 (interacts with) PC.ae.C38.3 |
| 0.85636075 | 6.189382578 | PC.aa.C36.2 (interacts with) PC.aa.C40.5 |
| 0.855103368 | 4.693178453 | PC.aa.C36.2 (interacts with) PC.aa.C38.3 |
| 0.837061325 | 9.996376614 | PC.aa.C36.2 (interacts with) PC.ae.C40.1 |
| 0.82694559 | 17.20201048 | PC.aa.C36.2 (interacts with) SM.C24.0 |
| 0.802201303 | 68.02513626 | PC.aa.C36.2 (interacts with) PC.ae.C42.3 |
| 0.982494817 | 7.575752353 | lysoPC.a.C16.0 (interacts with) lysoPC.a.C18.0 |
| 0.921475408 | 4.348484848 | lysoPC.a.C16.0 (interacts with) PC.aa.C34.4 |
| 0.919428322 | 3.373566414 | lysoPC.a.C16.0 (interacts with) PC.aa.C36.1 |
| 0.914366024 | 17.96560658 | lysoPC.a.C16.0 (interacts with) lysoPC.a.C17.0 |
| 0.910699367 | 3.268982933 | lysoPC.a.C16.0 (interacts with) PC.aa.C36.3 |
| 0.908097241 | 6.997923214 | lysoPC.a.C16.0 (interacts with) PC.aa.C40.4 |
| 0.907629643 | 4.556294376 | lysoPC.a.C16.0 (interacts with) PC.aa.C34.3 |
| 0.902321547 | 16.639088 | lysoPC.a.C16.0 (interacts with) PC.aa.C40.5 |
| 0.895965663 | 5.386324412 | lysoPC.a.C16.0 (interacts with) PC.aa.C34.1 |
| 0.89463669 | 8.52754583 | lysoPC.a.C16.0 (interacts with) PC.aa.C38.3 |
| 0.886329827 | 24.81639207 | lysoPC.a.C16.0 (interacts with) lysoPC.a.C20.3 |
| 0.885184354 | 7.336583491 | lysoPC.a.C16.0 (interacts with) PC.aa.C36.2 |
| 0.877912872 | 5.101551288 | lysoPC.a.C16.0 (interacts with) PC.aa.C32.2 |
| 0.862743272 | 25.28894211 | lysoPC.a.C16.0 (interacts with) PC.ae.C40.1 |
| 0.847547563 | 22.56680624 | lysoPC.a.C16.0 (interacts with) lysoPC.a.C18.2 |
| 0.845382132 | 7.752308804 | lysoPC.a.C16.0 (interacts with) PC.aa.C30.0 |
| 0.829298384 | 14.03681668 | lysoPC.a.C16.0 (interacts with) PC.aa.C32.3 |
| 0.824458489 | 30.55418872 | lysoPC.a.C16.0 (interacts with) PC.ae.C36.1 |
| 0.812762974 | 6.744793186 | lysoPC.a.C16.0 (interacts with) PC.aa.C34.2 |
| 0.808701544 | 86.94303 | lysoPC.a.C16.0 (interacts with) PC.ae.C38.3 |
| 0.923789903 | 4.423679267 | lysoPC.a.C18.0 (interacts with) PC.aa.C40.5 |
| 0.9096238 | 3.135642136 | lysoPC.a.C18.0 (interacts with) PC.aa.C38.3 |
| 0.907249702 | 4.684641836 | lysoPC.a.C18.0 (interacts with) PC.aa.C34.4 |
| 0.902796676 | 3.899357265 | lysoPC.a.C18.0 (interacts with) PC.aa.C40.4 |
| 0.89470559 | 9.166735573 | lysoPC.a.C18.0 (interacts with) PC.ae.C40.1 |
| 0.88815129 | 9.078531181 | lysoPC.a.C18.0 (interacts with) PC.aa.C36.1 |
| 0.88132698 | 4.655498355 | lysoPC.a.C18.0 (interacts with) PC.aa.C36.2 |
| 0.880228513 | 7.794676202 | lysoPC.a.C18.0 (interacts with) PC.aa.C36.3 |
| 0.876800051 | 6.470629186 | lysoPC.a.C18.0 (interacts with) PC.aa.C34.3 |
| 0.85874818 | 25.40127508 | lysoPC.a.C18.0 (interacts with) PC.ae.C38.3 |
| 0.855915536 | 10.08217058 | lysoPC.a.C18.0 (interacts with) PC.ae.C36.1 |
| 0.853545964 | 7.336712223 | lysoPC.a.C18.0 (interacts with) PC.aa.C34.1 |
| 0.843211021 | 6.726088395 | lysoPC.a.C18.0 (interacts with) PC.aa.C32.3 |
| 0.840785669 | 67.60802661 | lysoPC.a.C18.0 (interacts with) lysoPC.a.C20.3 |
| 0.838288486 | 7.348727255 | lysoPC.a.C18.0 (interacts with) PC.aa.C32.2 |
| 0.837073094 | 5.428484937 | lysoPC.a.C18.0 (interacts with) PC.aa.C30.0 |
| 0.833254664 | 15.27997932 | lysoPC.a.C18.0 (interacts with) SM.C24.0 |
| 0.823568264 | 56.93375988 | lysoPC.a.C18.0 (interacts with) PC.ae.C42.3 |
| 0.816150442 | 36.95604796 | lysoPC.a.C18.0 (interacts with) PC.aa.C42.6 |
| 0.815364678 | 21.44502008 | lysoPC.a.C18.0 (interacts with) PC.ae.C36.2 |
| 0.813820446 | 13.74113675 | lysoPC.a.C18.0 (interacts with) PC.aa.C38.4 |
| 0.804040136 | 13.60896506 | lysoPC.a.C18.0 (interacts with) PC.aa.C36.4 |
| 0.801280732 | 7.056001985 | lysoPC.a.C18.0 (interacts with) PC.aa.C34.2 |
| 0.979967616 | 2.479738562 | PC.ae.C34.3 (interacts with) PC.ae.C36.3 |
| 0.928508662 | 3.851815144 | PC.ae.C34.3 (interacts with) PC.ae.C36.2 |
| 0.901959743 | 6.267288657 | PC.ae.C34.3 (interacts with) PC.ae.C38.3 |
| 0.887063656 | 7.438922937 | PC.ae.C34.3 (interacts with) PC.ae.C38.4 |
| 0.856038049 | 17.07148904 | PC.ae.C34.3 (interacts with) PC.ae.C36.4 |
| 0.854231699 | 4.637457472 | PC.ae.C34.3 (interacts with) PC.ae.C36.1 |
| 0.852264856 | 13.85948497 | PC.ae.C34.3 (interacts with) PC.ae.C42.4 |
| 0.850756987 | 8.261861389 | PC.ae.C34.3 (interacts with) SM.OH.C24.1 |
| 0.828530106 | 17.02158393 | PC.ae.C34.3 (interacts with) PC.ae.C42.3 |
| 0.81318993 | 13.52964082 | PC.ae.C34.3 (interacts with) SM.OH.C14.1 |
| 0.810506887 | 8.247578425 | PC.ae.C34.3 (interacts with) SM.C24.0 |
| 0.808260748 | 16.32295467 | PC.ae.C34.3 (interacts with) PC.ae.C42.2 |
| 0.802727143 | 14.09195866 | PC.ae.C34.3 (interacts with) SM.OH.C22.1 |
| 0.800915171 | 15.80293359 | PC.ae.C34.3 (interacts with) PC.ae.C40.4 |
| 0.916433134 | 7.279980852 | PC.ae.C36.3 (interacts with) PC.ae.C38.3 |
| 0.887718823 | 8.76608018 | PC.ae.C36.3 (interacts with) PC.ae.C38.4 |
| 0.846800926 | 17.8507167 | PC.ae.C36.3 (interacts with) PC.ae.C36.4 |
| 0.842512833 | 21.77992296 | PC.ae.C36.3 (interacts with) PC.ae.C42.3 |
| 0.840672784 | 9.341760687 | PC.ae.C36.3 (interacts with) SM.OH.C24.1 |
| 0.83996056 | 13.84974851 | PC.ae.C36.3 (interacts with) PC.ae.C42.4 |
| 0.809791814 | 16.20566894 | PC.ae.C36.3 (interacts with) SM.OH.C14.1 |
| 0.801760251 | 16.99240413 | PC.ae.C36.3 (interacts with) PC.ae.C40.4 |
| 0.973868053 | 2 | C12.1 (interacts with) C14.1 |
| 0.950769129 | 2 | C12.1 (interacts with) C14.2 |
| 0.961773708 | 2 | C14.1 (interacts with) C14.2 |
| 0.971667241 | 4.626204343 | PC.ae.C34.2 (interacts with) PC.ae.C36.3 |
| 0.970468683 | 4.465182968 | PC.ae.C34.2 (interacts with) PC.ae.C34.3 |
| 0.919170114 | 3.829224178 | PC.ae.C34.2 (interacts with) PC.ae.C36.2 |
| 0.915256438 | 6.235871752 | PC.ae.C34.2 (interacts with) PC.ae.C36.4 |
| 0.913736897 | 5.741024568 | PC.ae.C34.2 (interacts with) PC.ae.C38.4 |
| 0.8999881 | 5.812233577 | PC.ae.C34.2 (interacts with) PC.ae.C42.4 |
| 0.886025468 | 7.748515432 | PC.ae.C34.2 (interacts with) PC.ae.C38.3 |
| 0.86576076 | 4.262097025 | PC.ae.C34.2 (interacts with) SM.OH.C24.1 |
| 0.84533799 | 5.636768636 | PC.ae.C34.2 (interacts with) SM.OH.C14.1 |
| 0.840025722 | 6.445451274 | PC.ae.C34.2 (interacts with) PC.ae.C40.4 |
| 0.829904422 | 9.329432705 | PC.ae.C34.2 (interacts with) PC.ae.C40.3 |
| 0.826404525 | 10.38439884 | PC.ae.C34.2 (interacts with) PC.ae.C42.2 |
| 0.815537004 | 9.502631792 | PC.ae.C34.2 (interacts with) SM.OH.C16.1 |
| 0.812099335 | 8.925277221 | PC.ae.C34.2 (interacts with) PC.ae.C36.1 |
| 0.80850499 | 13.33703626 | PC.ae.C34.2 (interacts with) PC.ae.C42.3 |
| 0.804552138 | 10.06582814 | PC.ae.C34.2 (interacts with) SM.C16.0 |
| 0.97163902 | 3.814274336 | PC.ae.C36.5 (interacts with) PC.ae.C38.5 |
| 0.932510058 | 6.318410552 | PC.ae.C36.5 (interacts with) PC.ae.C38.6 |
| 0.926176494 | 3.355802935 | PC.ae.C36.5 (interacts with) SM.C16.1 |
| 0.904031205 | 4.004111612 | PC.ae.C36.5 (interacts with) SM.C16.0 |
| 0.899536552 | 4.686807516 | PC.ae.C36.5 (interacts with) PC.ae.C40.5 |
| 0.893504109 | 4.442475431 | PC.ae.C36.5 (interacts with) PC.ae.C40.2 |
| 0.887475532 | 5.214541059 | PC.ae.C36.5 (interacts with) PC.ae.C42.2 |
| 0.877208579 | 6.088995483 | PC.ae.C36.5 (interacts with) PC.ae.C40.4 |
| 0.876553979 | 4.458230326 | PC.ae.C36.5 (interacts with) SM.OH.C16.1 |
| 0.876297375 | 8.773520102 | PC.ae.C36.5 (interacts with) PC.ae.C38.4 |
| 0.875511112 | 4.75865652 | PC.ae.C36.5 (interacts with) SM.OH.C22.2 |
| 0.872751753 | 6.79812909 | PC.ae.C36.5 (interacts with) SM.OH.C22.1 |
| 0.871432082 | 6.275323029 | PC.ae.C36.5 (interacts with) SM.OH.C24.1 |
| 0.869992897 | 11.31803874 | PC.ae.C36.5 (interacts with) PC.ae.C42.3 |
| 0.869854946 | 4.744406497 | PC.ae.C36.5 (interacts with) PC.ae.C40.3 |
| 0.858949602 | 7.014117622 | PC.ae.C36.5 (interacts with) PC.ae.C40.6 |
| 0.839555176 | 5.208043915 | PC.ae.C36.5 (interacts with) SM.OH.C14.1 |
| 0.820728567 | 29.8168009 | PC.ae.C36.5 (interacts with) PC.ae.C38.3 |
| 0.814063488 | 9.062988666 | PC.ae.C36.5 (interacts with) PC.ae.C38.0 |
| 0.801542696 | 16.75257131 | PC.ae.C36.5 (interacts with) SM.C24.0 |
| 0.800865652 | 9.238272487 | PC.ae.C36.5 (interacts with) SM.C18.1 |
| 0.932372926 | 4.494711793 | PC.ae.C38.5 (interacts with) SM.C16.1 |
| 0.931921909 | 6.805323394 | PC.ae.C38.5 (interacts with) PC.ae.C38.6 |
| 0.91970664 | 3.789325281 | PC.ae.C38.5 (interacts with) SM.C16.0 |
| 0.910705709 | 4.112714764 | PC.ae.C38.5 (interacts with) PC.ae.C40.2 |
| 0.899720872 | 4.136961862 | PC.ae.C38.5 (interacts with) SM.OH.C16.1 |
| 0.893642527 | 5.575464702 | PC.ae.C38.5 (interacts with) SM.OH.C22.2 |
| 0.890130888 | 7.601379433 | PC.ae.C38.5 (interacts with) PC.ae.C40.5 |
| 0.888010928 | 3.633423886 | PC.ae.C38.5 (interacts with) PC.ae.C40.3 |
| 0.883913293 | 6.470877782 | PC.ae.C38.5 (interacts with) PC.ae.C40.4 |
| 0.875701537 | 9.338081605 | PC.ae.C38.5 (interacts with) PC.ae.C42.2 |
| 0.868071656 | 8.177495666 | PC.ae.C38.5 (interacts with) PC.ae.C40.6 |
| 0.863141831 | 8.662137307 | PC.ae.C38.5 (interacts with) SM.OH.C24.1 |
| 0.855996158 | 12.855452 | PC.ae.C38.5 (interacts with) SM.OH.C22.1 |
| 0.853755776 | 6.196973762 | PC.ae.C38.5 (interacts with) SM.OH.C14.1 |
| 0.848938791 | 7.308474096 | PC.ae.C38.5 (interacts with) SM.C18.1 |
| 0.838702308 | 20.83573399 | PC.ae.C38.5 (interacts with) PC.ae.C42.3 |
| 0.834646472 | 5.484005656 | PC.ae.C38.5 (interacts with) SM.C18.0 |
| 0.820054102 | 35.56690368 | PC.ae.C38.5 (interacts with) PC.ae.C42.5 |
| 0.801033733 | 48.84280683 | PC.ae.C38.5 (interacts with) SM.C24.1 |
| 0.970755216 | 4 | Creatinine (interacts with) total.DMA |
| 0.929141617 | 3.337591773 | PC.ae.C40.6 (interacts with) SM.OH.C22.2 |
| 0.871262766 | 16.45313798 | PC.ae.C40.6 (interacts with) SM.C24.1 |
| 0.847496586 | 11.24216798 | PC.ae.C40.6 (interacts with) SM.C16.1 |
| 0.843312409 | 14.33024845 | PC.ae.C40.6 (interacts with) SM.OH.C16.1 |
| 0.832037365 | 21.0905353 | PC.ae.C40.6 (interacts with) SM.OH.C22.1 |
| 0.821308502 | 32.14602828 | PC.ae.C40.6 (interacts with) PC.ae.C42.3 |
| 0.819600332 | 13.04974001 | PC.ae.C40.6 (interacts with) SM.C16.0 |
| 0.816146444 | 17.56921143 | PC.ae.C40.6 (interacts with) PC.ae.C42.2 |
| 0.966980859 | 3.319795482 | PC.ae.C40.2 (interacts with) SM.OH.C22.2 |
| 0.93482966 | 7.469818918 | PC.ae.C40.2 (interacts with) SM.OH.C16.1 |
| 0.926847958 | 4.425519124 | PC.ae.C40.2 (interacts with) PC.ae.C40.6 |
| 0.916518748 | 4.82667389 | PC.ae.C40.2 (interacts with) PC.ae.C40.5 |
| 0.902754427 | 6.238779123 | PC.ae.C40.2 (interacts with) PC.ae.C40.3 |
| 0.901162359 | 6.79233606 | PC.ae.C40.2 (interacts with) SM.C16.0 |
| 0.900567379 | 6.400397686 | PC.ae.C40.2 (interacts with) SM.C16.1 |
| 0.888468738 | 9.205491894 | PC.ae.C40.2 (interacts with) SM.OH.C14.1 |
| 0.884956655 | 17.43184075 | PC.ae.C40.2 (interacts with) SM.OH.C22.1 |
| 0.871267688 | 18.7760296 | PC.ae.C40.2 (interacts with) SM.C24.1 |
| 0.867784418 | 6.582213326 | PC.ae.C40.2 (interacts with) SM.C18.0 |
| 0.85936378 | 12.15651825 | PC.ae.C40.2 (interacts with) PC.ae.C42.2 |
| 0.850419438 | 9.581304264 | PC.ae.C40.2 (interacts with) PC.ae.C40.4 |
| 0.831299929 | 24.97126957 | PC.ae.C40.2 (interacts with) PC.ae.C42.3 |
| 0.823284561 | 7.459823602 | PC.ae.C40.2 (interacts with) SM.C18.1 |
| 0.812101393 | 11.97021782 | PC.ae.C40.2 (interacts with) SM.OH.C24.1 |
| 0.927225697 | 7.427769445 | SM.OH.C22.2 (interacts with) SM.C16.1 |
| 0.879653918 | 6.495371087 | SM.OH.C22.2 (interacts with) SM.C18.0 |
| 0.878950192 | 8.558543938 | SM.OH.C22.2 (interacts with) SM.C16.0 |
| 0.87538464 | 7.629861599 | SM.OH.C22.2 (interacts with) SM.C18.1 |
| 0.855176889 | 18.1761464 | SM.OH.C22.2 (interacts with) SM.C24.1 |
| 0.964654512 | 5.699754783 | PC.aa.C36.5 (interacts with) PC.aa.C38.5 |
| 0.923611736 | 13.15626033 | PC.aa.C36.5 (interacts with) PC.aa.C42.6 |
| 0.909848806 | 4.581062276 | PC.aa.C36.5 (interacts with) PC.ae.C38.0 |
| 0.903188208 | 5.050370539 | PC.aa.C36.5 (interacts with) PC.aa.C40.6 |
| 0.902805981 | 5.259420445 | PC.aa.C36.5 (interacts with) PC.aa.C38.6 |
| 0.900910963 | 5.928345675 | PC.aa.C36.5 (interacts with) PC.aa.C36.6 |
| 0.885412883 | 8.627917791 | PC.aa.C36.5 (interacts with) PC.aa.C42.5 |
| 0.858816618 | 5.409973686 | PC.aa.C36.5 (interacts with) PC.ae.C40.6 |
| 0.857986679 | 8.113223829 | PC.aa.C36.5 (interacts with) PC.ae.C38.6 |
| 0.850123742 | 18.86283954 | PC.aa.C36.5 (interacts with) SM.OH.C22.1 |
| 0.838169856 | 5.902433747 | PC.aa.C36.5 (interacts with) SM.OH.C22.2 |
| 0.832626085 | 5.788501459 | PC.aa.C36.5 (interacts with) PC.ae.C40.5 |
| 0.830949315 | 9.671305534 | PC.aa.C36.5 (interacts with) PC.ae.C40.2 |
| 0.828872008 | 20.36590108 | PC.aa.C36.5 (interacts with) PC.ae.C42.2 |
| 0.814252145 | 23.22497525 | PC.aa.C36.5 (interacts with) PC.ae.C34.0 |
| 0.805543207 | 25.36654734 | PC.aa.C36.5 (interacts with) PC.ae.C42.3 |
| 0.952339754 | 3.881373522 | PC.aa.C38.5 (interacts with) PC.aa.C42.6 |
| 0.946590816 | 2.940681903 | PC.aa.C38.5 (interacts with) PC.aa.C40.6 |
| 0.926036714 | 3.79746094 | PC.aa.C38.5 (interacts with) PC.ae.C38.0 |
| 0.907523363 | 7.935494602 | PC.aa.C38.5 (interacts with) PC.aa.C38.6 |
| 0.881836128 | 8.9755262 | PC.aa.C38.5 (interacts with) SM.OH.C22.1 |
| 0.875392707 | 11.66979209 | PC.aa.C38.5 (interacts with) SM.C24.0 |
| 0.872512444 | 6.286879498 | PC.aa.C38.5 (interacts with) PC.aa.C42.5 |
| 0.863131655 | 11.55032019 | PC.aa.C38.5 (interacts with) PC.ae.C42.2 |
| 0.856603305 | 11.08050687 | PC.aa.C38.5 (interacts with) PC.ae.C42.3 |
| 0.839893022 | 10.78896244 | PC.aa.C38.5 (interacts with) PC.ae.C34.0 |
| 0.833675756 | 41.74146927 | PC.aa.C38.5 (interacts with) PC.aa.C40.5 |
| 0.822599613 | 7.239084331 | PC.aa.C38.5 (interacts with) PC.ae.C40.5 |
| 0.820542456 | 13.67669253 | PC.aa.C38.5 (interacts with) PC.ae.C38.6 |
| 0.818518303 | 10.78462512 | PC.aa.C38.5 (interacts with) PC.ae.C40.6 |
| 0.81104158 | 11.85156233 | PC.aa.C38.5 (interacts with) PC.ae.C36.5 |
| 0.811023879 | 15.3048986 | PC.aa.C38.5 (interacts with) PC.ae.C40.1 |
| 0.806077817 | 9.595331705 | PC.aa.C38.5 (interacts with) SM.OH.C22.2 |
| 0.963723234 | 6.312188317 | PC.aa.C36.4 (interacts with) PC.aa.C38.4 |
| 0.877325689 | 15.67148957 | PC.aa.C36.4 (interacts with) PC.aa.C40.4 |
| 0.821615275 | 13.29000726 | PC.aa.C36.4 (interacts with) PC.aa.C38.3 |
| 0.815942171 | 18.25488741 | PC.aa.C36.4 (interacts with) PC.aa.C40.5 |
| 0.813179892 | 52.5234273 | PC.aa.C36.4 (interacts with) PC.ae.C38.4 |
| 0.804516565 | 32.6259445 | PC.aa.C36.4 (interacts with) SM.C24.0 |
| 0.862895824 | 16.15162968 | PC.aa.C38.4 (interacts with) PC.aa.C40.4 |
| 0.846080719 | 12.58491272 | PC.aa.C38.4 (interacts with) PC.ae.C38.4 |
| 0.844176076 | 13.55898228 | PC.aa.C38.4 (interacts with) SM.OH.C24.1 |
| 0.841934599 | 13.90447654 | PC.aa.C38.4 (interacts with) PC.aa.C40.5 |
| 0.823782446 | 10.3878228 | PC.aa.C38.4 (interacts with) SM.C24.0 |
| 0.82366195 | 28.70516784 | PC.aa.C38.4 (interacts with) SM.C16.1 |
| 0.81388194 | 25.12115892 | PC.aa.C38.4 (interacts with) PC.ae.C42.2 |
| 0.807652236 | 16.0123963 | PC.aa.C38.4 (interacts with) PC.ae.C38.3 |
| 0.963061857 | 3.134355327 | PC.ae.C42.2 (interacts with) SM.OH.C22.1 |
| 0.938285749 | 4.174169453 | PC.ae.C42.2 (interacts with) SM.C16.1 |
| 0.917039607 | 5.556857328 | PC.ae.C42.2 (interacts with) SM.OH.C24.1 |
| 0.915281079 | 5.929075838 | PC.ae.C42.2 (interacts with) SM.C16.0 |
| 0.91409326 | 5.849492693 | PC.ae.C42.2 (interacts with) SM.OH.C16.1 |
| 0.912871233 | 8.11122397 | PC.ae.C42.2 (interacts with) SM.C24.0 |
| 0.909654514 | 5.176283365 | PC.ae.C42.2 (interacts with) SM.OH.C14.1 |
| 0.903843746 | 7.218745232 | PC.ae.C42.2 (interacts with) PC.ae.C42.3 |
| 0.860022051 | 11.19153594 | PC.ae.C42.2 (interacts with) SM.OH.C22.2 |
| 0.806247035 | 34.48375223 | PC.ae.C42.2 (interacts with) SM.C18.1 |
| 0.804870284 | 21.85926732 | PC.ae.C42.2 (interacts with) SM.C18.0 |
| 0.937105576 | 6.579082176 | SM.OH.C22.1 (interacts with) SM.C24.0 |
| 0.925586419 | 5.854148355 | SM.OH.C22.1 (interacts with) SM.C16.1 |
| 0.914958133 | 6.600267955 | SM.OH.C22.1 (interacts with) SM.OH.C24.1 |
| 0.903899017 | 7.800776075 | SM.OH.C22.1 (interacts with) SM.C16.0 |
| 0.899903396 | 14.03953372 | SM.OH.C22.1 (interacts with) SM.OH.C22.2 |
| 0.827489688 | 32.22734236 | SM.OH.C22.1 (interacts with) SM.C18.0 |
| 0.96185734 | 4.103418325 | PC.aa.C34.3 (interacts with) PC.aa.C36.3 |
| 0.951548839 | 5.306731944 | PC.aa.C34.3 (interacts with) PC.aa.C36.2 |
| 0.937152712 | 4.006376478 | PC.aa.C34.3 (interacts with) PC.aa.C34.4 |
| 0.92331883 | 4.505010422 | PC.aa.C34.3 (interacts with) PC.aa.C36.1 |
| 0.884447553 | 4.403252307 | PC.aa.C34.3 (interacts with) PC.aa.C40.4 |
| 0.84805609 | 22.81220319 | PC.aa.C34.3 (interacts with) PC.ae.C34.3 |
| 0.840353557 | 20.4290348 | PC.aa.C34.3 (interacts with) PC.ae.C36.3 |
| 0.838629303 | 6.903217744 | PC.aa.C34.3 (interacts with) PC.aa.C38.3 |
| 0.829328063 | 13.28028511 | PC.aa.C34.3 (interacts with) PC.aa.C40.5 |
| 0.80409412 | 26.40146822 | PC.aa.C34.3 (interacts with) PC.ae.C34.1 |
| 0.802585826 | 23.79365968 | PC.aa.C34.3 (interacts with) PC.ae.C40.1 |
| 0.801756875 | 24.34642981 | PC.aa.C34.3 (interacts with) PC.ae.C36.1 |
| 0.933604676 | 5.883296567 | PC.aa.C36.3 (interacts with) PC.aa.C40.4 |
| 0.903525252 | 8.356425535 | PC.aa.C36.3 (interacts with) PC.aa.C38.3 |
| 0.862712977 | 15.86520132 | PC.aa.C36.3 (interacts with) PC.aa.C40.5 |
| 0.852767952 | 26.78320727 | PC.aa.C36.3 (interacts with) PC.ae.C34.3 |
| 0.847669716 | 24.37685061 | PC.aa.C36.3 (interacts with) PC.ae.C36.3 |
| 0.830119126 | 22.76476565 | PC.aa.C36.3 (interacts with) PC.ae.C36.1 |
| 0.82265106 | 25.72904496 | PC.aa.C36.3 (interacts with) PC.ae.C34.1 |
| 0.821444285 | 26.59392861 | PC.aa.C36.3 (interacts with) PC.ae.C40.1 |
| 0.818613716 | 71.54197828 | PC.aa.C36.3 (interacts with) PC.ae.C38.3 |
| 0.811659542 | 42.55876147 | PC.aa.C36.3 (interacts with) PC.ae.C36.2 |
| 0.800371016 | 27.71663827 | PC.aa.C36.3 (interacts with) PC.ae.C30.0 |
| 0.961585427 | 3.076190476 | PC.aa.C38.3 (interacts with) PC.aa.C40.5 |
| 0.958461383 | 3.344501069 | PC.aa.C38.3 (interacts with) PC.aa.C40.4 |
| 0.90941224 | 13.12016729 | PC.aa.C38.3 (interacts with) SM.C24.0 |
| 0.87560792 | 20.10137282 | PC.aa.C38.3 (interacts with) PC.ae.C38.3 |
| 0.865103954 | 8.094305423 | PC.aa.C38.3 (interacts with) PC.ae.C36.1 |
| 0.847624297 | 10.40260637 | PC.aa.C38.3 (interacts with) PC.ae.C34.1 |
| 0.839001748 | 12.86335622 | PC.aa.C38.3 (interacts with) PC.aa.C38.4 |
| 0.832667877 | 10.1824873 | PC.aa.C38.3 (interacts with) PC.ae.C40.1 |
| 0.823284359 | 36.55183042 | PC.aa.C38.3 (interacts with) PC.aa.C42.6 |
| 0.805559709 | 11.60221605 | PC.aa.C38.3 (interacts with) PC.ae.C30.0 |
| 0.804974097 | 50.65553718 | PC.aa.C38.3 (interacts with) SM.OH.C22.1 |
| 0.927564592 | 10.51118004 | PC.aa.C40.5 (interacts with) SM.C24.0 |
| 0.868469731 | 7.327070861 | PC.aa.C40.5 (interacts with) PC.ae.C36.1 |
| 0.860291205 | 16.42896118 | PC.aa.C40.5 (interacts with) PC.ae.C38.3 |
| 0.846520443 | 9.725081829 | PC.aa.C40.5 (interacts with) PC.ae.C34.1 |
| 0.845860372 | 58.04118685 | PC.aa.C40.5 (interacts with) PC.ae.C42.2 |
| 0.844008781 | 28.40692324 | PC.aa.C40.5 (interacts with) PC.aa.C42.6 |
| 0.833634936 | 39.15105966 | PC.aa.C40.5 (interacts with) SM.OH.C22.1 |
| 0.831150761 | 49.69352981 | PC.aa.C40.5 (interacts with) PC.aa.C40.6 |
| 0.821101918 | 9.316090464 | PC.aa.C40.5 (interacts with) PC.ae.C40.1 |
| 0.811772774 | 10.87519995 | PC.aa.C40.5 (interacts with) PC.ae.C30.0 |
| 0.810733709 | 41.76520594 | PC.aa.C40.5 (interacts with) PC.ae.C34.0 |
| 0.960953839 | 3.526312487 | PC.aa.C28.1 (interacts with) PC.ae.C30.0 |
| 0.959654141 | 3.243878941 | PC.aa.C28.1 (interacts with) PC.aa.C32.0 |
| 0.95234053 | 3.879870041 | PC.aa.C28.1 (interacts with) PC.ae.C34.1 |
| 0.951159544 | 4.749330702 | PC.aa.C28.1 (interacts with) PC.ae.C36.1 |
| 0.944216162 | 6.50270832 | PC.aa.C28.1 (interacts with) PC.ae.C34.0 |
| 0.93739196 | 4.202226328 | PC.aa.C28.1 (interacts with) PC.ae.C38.3 |
| 0.936667441 | 7.484034888 | PC.aa.C28.1 (interacts with) SM.OH.C14.1 |
| 0.925422744 | 6.705488816 | PC.aa.C28.1 (interacts with) SM.OH.C22.1 |
| 0.921350145 | 3.443253968 | PC.aa.C28.1 (interacts with) PC.ae.C36.2 |
| 0.912800207 | 7.758387341 | PC.aa.C28.1 (interacts with) PC.ae.C42.2 |
| 0.908047651 | 5.403951185 | PC.aa.C28.1 (interacts with) SM.C24.0 |
| 0.904683059 | 12.04685459 | PC.aa.C28.1 (interacts with) SM.OH.C16.1 |
| 0.90161289 | 13.67645592 | PC.aa.C28.1 (interacts with) PC.aa.C30.0 |
| 0.896251232 | 12.54520223 | PC.aa.C28.1 (interacts with) SM.C16.0 |
| 0.892793118 | 8.196148762 | PC.aa.C28.1 (interacts with) PC.aa.C32.3 |
| 0.891669814 | 9.170887474 | PC.aa.C28.1 (interacts with) PC.ae.C32.1 |
| 0.887171024 | 5.085277276 | PC.aa.C28.1 (interacts with) PC.ae.C38.4 |
| 0.885260014 | 9.267426927 | PC.aa.C28.1 (interacts with) PC.ae.C42.3 |
| 0.883908731 | 4.211520036 | PC.aa.C28.1 (interacts with) PC.ae.C34.3 |
| 0.87985392 | 4.075690775 | PC.aa.C28.1 (interacts with) PC.ae.C34.2 |
| 0.877833547 | 5.331506352 | PC.aa.C28.1 (interacts with) SM.OH.C24.1 |
| 0.877606631 | 11.60845476 | PC.aa.C28.1 (interacts with) SM.C16.1 |
| 0.875027524 | 13.50990981 | PC.aa.C28.1 (interacts with) PC.aa.C36.2 |
| 0.869355698 | 17.60649091 | PC.aa.C28.1 (interacts with) PC.aa.C34.2 |
| 0.864904304 | 4.68085706 | PC.aa.C28.1 (interacts with) PC.ae.C36.3 |
| 0.85909316 | 8.984803047 | PC.aa.C28.1 (interacts with) PC.aa.C42.5 |
| 0.850429364 | 15.32158944 | PC.aa.C28.1 (interacts with) PC.aa.C40.5 |
| 0.850019154 | 9.45383412 | PC.aa.C28.1 (interacts with) PC.ae.C40.4 |
| 0.846455074 | 15.47916417 | PC.aa.C28.1 (interacts with) PC.ae.C40.3 |
| 0.841657819 | 15.57497577 | PC.aa.C28.1 (interacts with) PC.ae.C32.2 |
| 0.833589299 | 16.95420988 | PC.aa.C28.1 (interacts with) PC.aa.C38.3 |
| 0.828324186 | 21.81453149 | PC.aa.C28.1 (interacts with) PC.aa.C40.4 |
| 0.822393422 | 13.79151797 | PC.aa.C28.1 (interacts with) PC.ae.C42.4 |
| 0.818624912 | 13.36957147 | PC.aa.C28.1 (interacts with) PC.aa.C42.6 |
| 0.95107061 | 2.611544012 | PC.ae.C30.0 (interacts with) PC.ae.C34.1 |
| 0.935679947 | 3.850975225 | PC.ae.C30.0 (interacts with) PC.ae.C36.1 |
| 0.912089531 | 4.111435697 | PC.ae.C30.0 (interacts with) PC.ae.C36.2 |
| 0.909665986 | 5.394177261 | PC.ae.C30.0 (interacts with) PC.ae.C38.3 |
| 0.908947185 | 11.27736458 | PC.ae.C30.0 (interacts with) PC.ae.C34.0 |
| 0.89444438 | 3.244952372 | PC.ae.C30.0 (interacts with) PC.ae.C34.3 |
| 0.880254358 | 13.4388059 | PC.ae.C30.0 (interacts with) SM.OH.C14.1 |
| 0.877987555 | 3.801752859 | PC.ae.C30.0 (interacts with) PC.ae.C36.3 |
| 0.877354536 | 5.400788967 | PC.ae.C30.0 (interacts with) PC.ae.C34.2 |
| 0.866937937 | 16.24798042 | PC.ae.C30.0 (interacts with) PC.ae.C32.1 |
| 0.849262497 | 6.887446171 | PC.ae.C30.0 (interacts with) SM.C24.0 |
| 0.848870624 | 15.2321045 | PC.ae.C30.0 (interacts with) PC.ae.C42.3 |
| 0.846216414 | 7.857833956 | PC.ae.C30.0 (interacts with) PC.ae.C38.4 |
| 0.845085622 | 11.62126362 | PC.ae.C30.0 (interacts with) SM.OH.C22.1 |
| 0.836006523 | 13.96568837 | PC.ae.C30.0 (interacts with) PC.ae.C42.2 |
| 0.835832152 | 17.16838586 | PC.ae.C30.0 (interacts with) PC.ae.C42.4 |
| 0.829187881 | 23.31666222 | PC.ae.C30.0 (interacts with) SM.OH.C16.1 |
| 0.823294797 | 17.20513274 | PC.ae.C30.0 (interacts with) PC.ae.C40.4 |
| 0.809685642 | 25.49942467 | PC.ae.C30.0 (interacts with) SM.C16.0 |
| 0.945285811 | 4.530215654 | PC.aa.C32.0 (interacts with) PC.ae.C34.1 |
| 0.922637077 | 8.246169978 | PC.aa.C32.0 (interacts with) PC.ae.C42.2 |
| 0.922574265 | 7.485939968 | PC.aa.C32.0 (interacts with) PC.ae.C34.0 |
| 0.922208735 | 4.406092179 | PC.aa.C32.0 (interacts with) PC.ae.C30.0 |
| 0.914328494 | 4.935931362 | PC.aa.C32.0 (interacts with) SM.C24.0 |
| 0.911082304 | 15.63055785 | PC.aa.C32.0 (interacts with) SM.C16.0 |
| 0.906254127 | 4.942265666 | PC.aa.C32.0 (interacts with) PC.ae.C36.1 |
| 0.902200039 | 11.98582886 | PC.aa.C32.0 (interacts with) PC.ae.C32.1 |
| 0.901390714 | 7.650181007 | PC.aa.C32.0 (interacts with) SM.OH.C22.1 |
| 0.896070145 | 4.232017512 | PC.aa.C32.0 (interacts with) PC.ae.C38.3 |
| 0.895795659 | 9.330527774 | PC.aa.C32.0 (interacts with) SM.OH.C14.1 |
| 0.888715802 | 9.734510278 | PC.aa.C32.0 (interacts with) PC.aa.C42.5 |
| 0.886426605 | 13.90587751 | PC.aa.C32.0 (interacts with) PC.aa.C40.5 |
| 0.87934785 | 12.88743192 | PC.aa.C32.0 (interacts with) SM.C16.1 |
| 0.868979325 | 14.833269 | PC.aa.C32.0 (interacts with) SM.OH.C16.1 |
| 0.868519505 | 6.185083785 | PC.aa.C32.0 (interacts with) SM.OH.C24.1 |
| 0.86230791 | 5.815134925 | PC.aa.C32.0 (interacts with) PC.ae.C38.4 |
| 0.848206597 | 10.18545385 | PC.aa.C32.0 (interacts with) PC.ae.C42.3 |
| 0.845195261 | 21.14595406 | PC.aa.C32.0 (interacts with) PC.aa.C40.4 |
| 0.844366154 | 16.42914051 | PC.aa.C32.0 (interacts with) PC.aa.C38.3 |
| 0.842062557 | 4.936748054 | PC.aa.C32.0 (interacts with) PC.ae.C34.3 |
| 0.842009572 | 9.230931279 | PC.aa.C32.0 (interacts with) PC.aa.C32.3 |
| 0.839602128 | 4.914894243 | PC.aa.C32.0 (interacts with) PC.ae.C36.2 |
| 0.838595558 | 5.57456443 | PC.aa.C32.0 (interacts with) PC.ae.C34.2 |
| 0.830176237 | 12.26442892 | PC.aa.C32.0 (interacts with) PC.aa.C42.6 |
| 0.829910805 | 18.16756283 | PC.aa.C32.0 (interacts with) PC.aa.C40.6 |
| 0.82988351 | 14.49539185 | PC.aa.C32.0 (interacts with) PC.aa.C36.2 |
| 0.823739948 | 18.18617039 | PC.aa.C32.0 (interacts with) PC.ae.C36.5 |
| 0.815594968 | 11.80750886 | PC.aa.C32.0 (interacts with) PC.aa.C38.4 |
| 0.815337845 | 20.70547724 | PC.aa.C32.0 (interacts with) PC.aa.C34.2 |
| 0.812917544 | 19.66331155 | PC.aa.C32.0 (interacts with) PC.ae.C32.2 |
| 0.81008225 | 12.26736165 | PC.aa.C32.0 (interacts with) PC.ae.C40.4 |
| 0.808833603 | 44.47500741 | PC.aa.C32.0 (interacts with) PC.aa.C34.1 |
| 0.80641732 | 6.134558348 | PC.aa.C32.0 (interacts with) PC.ae.C36.3 |
| 0.805069483 | 16.33128047 | PC.aa.C32.0 (interacts with) PC.ae.C36.4 |
| 0.958746996 | 4.69219797 | PC.ae.C36.1 (interacts with) PC.ae.C38.3 |
| 0.937597317 | 4.903551481 | PC.ae.C36.1 (interacts with) PC.ae.C36.2 |
| 0.921566178 | 14.47366814 | PC.ae.C36.1 (interacts with) SM.OH.C22.1 |
| 0.912392111 | 15.33812668 | PC.ae.C36.1 (interacts with) PC.ae.C42.3 |
| 0.899688362 | 6.431774455 | PC.ae.C36.1 (interacts with) SM.C24.0 |
| 0.891981539 | 22.70589883 | PC.ae.C36.1 (interacts with) SM.OH.C14.1 |
| 0.880291076 | 18.78624991 | PC.ae.C36.1 (interacts with) PC.ae.C42.2 |
| 0.869788661 | 9.139532295 | PC.ae.C36.1 (interacts with) PC.ae.C40.1 |
| 0.861439684 | 4.998717388 | PC.ae.C36.1 (interacts with) PC.ae.C36.3 |
| 0.851150426 | 12.11521539 | PC.ae.C36.1 (interacts with) PC.ae.C38.4 |
| 0.850660999 | 39.52622339 | PC.ae.C36.1 (interacts with) SM.OH.C16.1 |
| 0.831245753 | 15.34696826 | PC.ae.C36.1 (interacts with) SM.OH.C24.1 |
| 0.804180853 | 30.23607859 | PC.ae.C36.1 (interacts with) PC.ae.C40.4 |
| 0.802340008 | 38.18313062 | PC.ae.C36.1 (interacts with) SM.C16.1 |
| 0.933422358 | 8.062508695 | PC.ae.C38.3 (interacts with) SM.OH.C22.1 |
| 0.933284245 | 9.188717761 | PC.ae.C38.3 (interacts with) PC.ae.C42.3 |
| 0.920859326 | 7.613221002 | PC.ae.C38.3 (interacts with) PC.ae.C38.4 |
| 0.91411519 | 10.0318347 | PC.ae.C38.3 (interacts with) PC.ae.C42.2 |
| 0.91021155 | 5.264407022 | PC.ae.C38.3 (interacts with) SM.C24.0 |
| 0.904851327 | 9.8722825 | PC.ae.C38.3 (interacts with) SM.OH.C24.1 |
| 0.8882939 | 14.1126964 | PC.ae.C38.3 (interacts with) SM.OH.C14.1 |
| 0.871619331 | 22.03439691 | PC.ae.C38.3 (interacts with) SM.OH.C16.1 |
| 0.861018974 | 17.88490765 | PC.ae.C38.3 (interacts with) PC.ae.C40.4 |
| 0.852894604 | 14.49416066 | PC.ae.C38.3 (interacts with) PC.ae.C40.1 |
| 0.847279842 | 19.26451298 | PC.ae.C38.3 (interacts with) SM.C16.1 |
| 0.840583729 | 30.95820998 | PC.ae.C38.3 (interacts with) PC.ae.C40.3 |
| 0.835514896 | 25.76050408 | PC.ae.C38.3 (interacts with) SM.C16.0 |
| 0.820978846 | 44.1512569 | PC.ae.C38.3 (interacts with) PC.ae.C40.5 |
| 0.816303135 | 64.40125961 | PC.ae.C38.3 (interacts with) PC.ae.C38.5 |
| 0.93692922 | 5.667809014 | PC.aa.C40.4 (interacts with) PC.aa.C40.5 |
| 0.864827808 | 12.11073279 | PC.aa.C40.4 (interacts with) PC.ae.C34.1 |
| 0.864392062 | 22.49194254 | PC.aa.C40.4 (interacts with) SM.C24.0 |
| 0.863687809 | 31.01564405 | PC.aa.C40.4 (interacts with) PC.ae.C38.3 |
| 0.852506789 | 10.51102607 | PC.aa.C40.4 (interacts with) PC.ae.C36.1 |
| 0.835393716 | 13.26416267 | PC.aa.C40.4 (interacts with) PC.ae.C34.3 |
| 0.819350832 | 13.5065874 | PC.aa.C40.4 (interacts with) PC.ae.C30.0 |
| 0.815585507 | 12.92525791 | PC.aa.C40.4 (interacts with) PC.ae.C36.3 |
| 0.807467402 | 15.859127 | PC.aa.C40.4 (interacts with) PC.ae.C40.1 |
| 0.957176537 | 3.744740583 | SM.C16.0 (interacts with) SM.C16.1 |
| 0.937875671 | 10.00615052 | SM.C16.0 (interacts with) SM.C18.0 |
| 0.876918984 | 13.08219062 | SM.C16.0 (interacts with) SM.C18.1 |
| 0.824568164 | 22.07584656 | SM.C16.0 (interacts with) SM.C24.0 |
| 0.95787989 | 2.981005565 | PC.ae.C34.0 (interacts with) SM.OH.C22.1 |
| 0.953767512 | 13.70596191 | PC.ae.C34.0 (interacts with) PC.ae.C36.1 |
| 0.949683795 | 3.748102845 | PC.ae.C34.0 (interacts with) PC.ae.C42.3 |
| 0.942405263 | 7.666364994 | PC.ae.C34.0 (interacts with) PC.ae.C38.3 |
| 0.93561474 | 3.742406836 | PC.ae.C34.0 (interacts with) PC.ae.C42.2 |
| 0.934744651 | 6.612982227 | PC.ae.C34.0 (interacts with) SM.OH.C14.1 |
| 0.92796839 | 7.983712575 | PC.ae.C34.0 (interacts with) SM.OH.C16.1 |
| 0.908758941 | 9.88966367 | PC.ae.C34.0 (interacts with) PC.ae.C40.5 |
| 0.90525628 | 8.523067251 | PC.ae.C34.0 (interacts with) PC.ae.C36.2 |
| 0.89976791 | 7.004739876 | PC.ae.C34.0 (interacts with) SM.C24.0 |
| 0.898923963 | 7.739321324 | PC.ae.C34.0 (interacts with) PC.ae.C40.4 |
| 0.894928451 | 13.35078675 | PC.ae.C34.0 (interacts with) PC.ae.C34.1 |
| 0.893766812 | 8.226410228 | PC.ae.C34.0 (interacts with) SM.C16.0 |
| 0.891082669 | 6.719793107 | PC.ae.C34.0 (interacts with) SM.C16.1 |
| 0.87746258 | 7.520116966 | PC.ae.C34.0 (interacts with) PC.ae.C38.4 |
| 0.874396792 | 19.01375523 | PC.ae.C34.0 (interacts with) PC.ae.C40.2 |
| 0.8652413 | 7.353326178 | PC.ae.C34.0 (interacts with) PC.ae.C36.5 |
| 0.863181061 | 18.18315703 | PC.ae.C34.0 (interacts with) SM.OH.C22.2 |
| 0.861667316 | 10.31728528 | PC.ae.C34.0 (interacts with) PC.ae.C40.3 |
| 0.8613515 | 11.07490232 | PC.ae.C34.0 (interacts with) PC.ae.C38.0 |
| 0.860671237 | 6.656445116 | PC.ae.C34.0 (interacts with) SM.OH.C24.1 |
| 0.847315699 | 14.0061463 | PC.ae.C34.0 (interacts with) PC.ae.C38.5 |
| 0.83260866 | 18.50315499 | PC.ae.C34.0 (interacts with) PC.ae.C40.1 |
| 0.825515943 | 26.78721954 | PC.ae.C34.0 (interacts with) PC.ae.C40.6 |
| 0.816818142 | 13.22037094 | PC.ae.C34.0 (interacts with) PC.ae.C34.3 |
| 0.814550651 | 17.96007131 | PC.ae.C34.0 (interacts with) PC.ae.C36.3 |
| 0.809389263 | 10.04341625 | PC.ae.C34.0 (interacts with) PC.ae.C34.2 |
| 0.806868648 | 27.97062121 | PC.ae.C34.0 (interacts with) PC.ae.C38.6 |
| 0.803863572 | 31.6492294 | PC.ae.C34.0 (interacts with) PC.ae.C36.0 |
| 0.957661237 | 2.43688127 | PC.aa.C32.2 (interacts with) PC.aa.C34.3 |
| 0.943730055 | 4.312656632 | PC.aa.C32.2 (interacts with) PC.aa.C36.3 |
| 0.925914319 | 4.551372157 | PC.aa.C32.2 (interacts with) PC.aa.C34.4 |
| 0.921036056 | 5.598008196 | PC.aa.C32.2 (interacts with) PC.aa.C36.2 |
| 0.917165137 | 4.162513661 | PC.aa.C32.2 (interacts with) PC.aa.C36.1 |
| 0.910258175 | 12.05219338 | PC.aa.C32.2 (interacts with) PC.aa.C32.3 |
| 0.898039244 | 3.195249464 | PC.aa.C32.2 (interacts with) PC.aa.C34.1 |
| 0.876827374 | 4.210930996 | PC.aa.C32.2 (interacts with) PC.aa.C34.2 |
| 0.872419141 | 4.611757161 | PC.aa.C32.2 (interacts with) PC.aa.C40.4 |
| 0.844470855 | 7.369087455 | PC.aa.C32.2 (interacts with) PC.aa.C38.3 |
| 0.834902043 | 24.73726547 | PC.aa.C32.2 (interacts with) PC.ae.C30.0 |
| 0.834637798 | 21.20999228 | PC.aa.C32.2 (interacts with) PC.ae.C34.3 |
| 0.83122301 | 23.12771713 | PC.aa.C32.2 (interacts with) PC.ae.C34.1 |
| 0.820615636 | 18.92140142 | PC.aa.C32.2 (interacts with) PC.ae.C36.3 |
| 0.820201893 | 14.93422483 | PC.aa.C32.2 (interacts with) PC.aa.C40.5 |
| 0.816231503 | 23.11500625 | PC.aa.C32.2 (interacts with) PC.ae.C36.1 |
| 0.957267191 | 7.028271402 | PC.aa.C38.6 (interacts with) PC.aa.C40.6 |
| 0.942282245 | 3.460942051 | PC.aa.C38.6 (interacts with) PC.ae.C40.6 |
| 0.932903324 | 3.154714945 | PC.aa.C38.6 (interacts with) PC.ae.C38.6 |
| 0.910460817 | 5.748576405 | PC.aa.C38.6 (interacts with) PC.ae.C38.0 |
| 0.903097791 | 10.83275354 | PC.aa.C38.6 (interacts with) PC.ae.C42.2 |
| 0.901635169 | 7.439842552 | PC.aa.C38.6 (interacts with) SM.C16.1 |
| 0.895826414 | 2.625103391 | PC.aa.C38.6 (interacts with) SM.OH.C22.2 |
| 0.891576971 | 12.95221025 | PC.aa.C38.6 (interacts with) SM.OH.C22.1 |
| 0.88601479 | 13.57476893 | PC.aa.C38.6 (interacts with) PC.aa.C42.6 |
| 0.873840726 | 4.160904923 | PC.aa.C38.6 (interacts with) PC.ae.C40.5 |
| 0.871003514 | 4.110046205 | PC.aa.C38.6 (interacts with) PC.ae.C40.2 |
| 0.870251128 | 5.91856468 | PC.aa.C38.6 (interacts with) PC.aa.C42.5 |
| 0.863496283 | 5.1228944 | PC.aa.C38.6 (interacts with) PC.ae.C36.5 |
| 0.858100909 | 16.00583258 | PC.aa.C38.6 (interacts with) PC.ae.C34.0 |
| 0.856580788 | 6.701294086 | PC.aa.C38.6 (interacts with) PC.ae.C38.5 |
| 0.850456225 | 9.992297862 | PC.aa.C38.6 (interacts with) SM.C16.0 |
| 0.84623667 | 8.244315535 | PC.aa.C38.6 (interacts with) PC.ae.C32.2 |
| 0.844039454 | 20.28935729 | PC.aa.C38.6 (interacts with) PC.ae.C42.3 |
| 0.834586034 | 11.14831343 | PC.aa.C38.6 (interacts with) SM.OH.C16.1 |
| 0.815843893 | 36.72062862 | PC.aa.C38.6 (interacts with) SM.C24.0 |
| 0.810831724 | 7.348057335 | PC.aa.C38.6 (interacts with) SM.C18.0 |
| 0.807502294 | 8.286347104 | PC.aa.C38.6 (interacts with) SM.C18.1 |
| 0.801738111 | 22.34685423 | PC.aa.C38.6 (interacts with) SM.C24.1 |
| 0.922772824 | 4.503620429 | PC.aa.C40.6 (interacts with) PC.aa.C42.6 |
| 0.900016805 | 4.741565232 | PC.aa.C40.6 (interacts with) PC.ae.C38.0 |
| 0.875052329 | 5.382271825 | PC.aa.C40.6 (interacts with) PC.aa.C42.5 |
| 0.871851039 | 8.637142082 | PC.aa.C40.6 (interacts with) SM.OH.C22.1 |
| 0.871566592 | 9.907967553 | PC.aa.C40.6 (interacts with) PC.ae.C42.2 |
| 0.855991805 | 9.468904337 | PC.aa.C40.6 (interacts with) PC.ae.C40.6 |
| 0.854852858 | 10.08664176 | PC.aa.C40.6 (interacts with) PC.ae.C34.0 |
| 0.851575334 | 11.67961321 | PC.aa.C40.6 (interacts with) SM.C24.0 |
| 0.840479639 | 12.17980544 | PC.aa.C40.6 (interacts with) PC.ae.C38.6 |
| 0.838914012 | 12.17120545 | PC.aa.C40.6 (interacts with) SM.C16.1 |
| 0.838536786 | 11.94595874 | PC.aa.C40.6 (interacts with) PC.ae.C42.3 |
| 0.814094616 | 8.47275444 | PC.aa.C40.6 (interacts with) SM.OH.C22.2 |
| 0.810401495 | 6.653292498 | PC.aa.C40.6 (interacts with) PC.ae.C40.5 |
| 0.931047749 | 12.78055795 | SM.C16.1 (interacts with) SM.C18.1 |
| 0.910386769 | 10.25084309 | SM.C16.1 (interacts with) SM.C18.0 |
| 0.821636725 | 13.89652078 | SM.C16.1 (interacts with) SM.C24.0 |
| 0.941376743 | 3.317208991 | PC.ae.C34.1 (interacts with) PC.ae.C36.1 |
| 0.93258683 | 5.664733234 | PC.ae.C34.1 (interacts with) PC.ae.C38.3 |
| 0.89330216 | 4.516752603 | PC.ae.C34.1 (interacts with) PC.ae.C36.2 |
| 0.891397719 | 3.444519472 | PC.ae.C34.1 (interacts with) PC.ae.C34.3 |
| 0.88565534 | 16.98285113 | PC.ae.C34.1 (interacts with) SM.OH.C14.1 |
| 0.881804746 | 3.756875514 | PC.ae.C34.1 (interacts with) PC.ae.C36.3 |
| 0.877772985 | 9.647589322 | PC.ae.C34.1 (interacts with) PC.ae.C38.4 |
| 0.872889452 | 6.509582446 | PC.ae.C34.1 (interacts with) PC.ae.C34.2 |
| 0.852818787 | 6.961455073 | PC.ae.C34.1 (interacts with) SM.C24.0 |
| 0.851250835 | 22.63109919 | PC.ae.C34.1 (interacts with) PC.ae.C42.4 |
| 0.849758004 | 13.48091738 | PC.ae.C34.1 (interacts with) SM.OH.C22.1 |
| 0.846767293 | 16.48713792 | PC.ae.C34.1 (interacts with) PC.ae.C42.2 |
| 0.835792896 | 30.02355733 | PC.ae.C34.1 (interacts with) SM.OH.C16.1 |
| 0.833801143 | 11.44723907 | PC.ae.C34.1 (interacts with) SM.OH.C24.1 |
| 0.830595534 | 33.71710645 | PC.ae.C34.1 (interacts with) SM.C16.0 |
| 0.821875309 | 17.4719139 | PC.ae.C34.1 (interacts with) PC.ae.C42.3 |
| 0.935985893 | 5.705690051 | PC.aa.C42.6 (interacts with) PC.ae.C38.0 |
| 0.919820002 | 6.882109801 | PC.aa.C42.6 (interacts with) PC.ae.C42.3 |
| 0.916362182 | 6.643929103 | PC.aa.C42.6 (interacts with) PC.ae.C34.0 |
| 0.913452494 | 6.345200115 | PC.aa.C42.6 (interacts with) SM.OH.C22.1 |
| 0.908781807 | 7.595157382 | PC.aa.C42.6 (interacts with) SM.C24.0 |
| 0.88156314 | 8.407693206 | PC.aa.C42.6 (interacts with) PC.ae.C42.2 |
| 0.877606145 | 9.56421803 | PC.aa.C42.6 (interacts with) PC.ae.C40.5 |
| 0.877510512 | 17.033721 | PC.aa.C42.6 (interacts with) PC.ae.C36.1 |
| 0.871081794 | 12.72542467 | PC.aa.C42.6 (interacts with) PC.ae.C38.3 |
| 0.868637072 | 11.60693645 | PC.aa.C42.6 (interacts with) PC.ae.C40.1 |
| 0.823138807 | 20.02055805 | PC.aa.C42.6 (interacts with) PC.ae.C40.6 |
| 0.804727216 | 12.39324999 | PC.aa.C42.6 (interacts with) PC.ae.C36.5 |
| 0.80359942 | 16.19291775 | PC.aa.C42.6 (interacts with) SM.OH.C22.2 |
| 0.803038047 | 22.99474841 | PC.aa.C42.6 (interacts with) PC.ae.C36.0 |
| 0.801365254 | 23.82461528 | PC.aa.C42.6 (interacts with) PC.ae.C38.6 |
| 0.952196382 | 4.174992663 | PC.ae.C36.2 (interacts with) PC.ae.C36.3 |
| 0.952024539 | 4.695109938 | PC.ae.C36.2 (interacts with) PC.ae.C38.3 |
| 0.910872658 | 10.01583652 | PC.ae.C36.2 (interacts with) PC.ae.C42.3 |
| 0.894323962 | 8.570701285 | PC.ae.C36.2 (interacts with) SM.OH.C14.1 |
| 0.893137712 | 8.569318434 | PC.ae.C36.2 (interacts with) SM.OH.C22.1 |
| 0.887416997 | 5.583750694 | PC.ae.C36.2 (interacts with) PC.ae.C38.4 |
| 0.873033468 | 9.962057686 | PC.ae.C36.2 (interacts with) PC.ae.C42.2 |
| 0.868235686 | 5.915447889 | PC.ae.C36.2 (interacts with) SM.OH.C24.1 |
| 0.858237168 | 14.17295097 | PC.ae.C36.2 (interacts with) SM.OH.C16.1 |
| 0.850420597 | 13.8473804 | PC.ae.C36.2 (interacts with) PC.ae.C40.1 |
| 0.841642929 | 10.50740702 | PC.ae.C36.2 (interacts with) PC.ae.C40.4 |
| 0.840474231 | 6.915868664 | PC.ae.C36.2 (interacts with) SM.C24.0 |
| 0.823121396 | 16.47451268 | PC.ae.C36.2 (interacts with) PC.ae.C40.3 |
| 0.806032497 | 14.46057955 | PC.ae.C36.2 (interacts with) PC.ae.C42.4 |
| 0.800719349 | 13.8975654 | PC.ae.C36.2 (interacts with) SM.C16.1 |
| 0.950932426 | 6.568735258 | lysoPC.a.C17.0 (interacts with) lysoPC.a.C18.0 |
| 0.912611705 | 8.558325328 | lysoPC.a.C17.0 (interacts with) PC.ae.C38.3 |
| 0.906969292 | 5.463755414 | lysoPC.a.C17.0 (interacts with) PC.ae.C36.1 |
| 0.902641142 | 4.098268398 | lysoPC.a.C17.0 (interacts with) PC.ae.C40.1 |
| 0.880792109 | 14.83749726 | lysoPC.a.C17.0 (interacts with) PC.ae.C42.3 |
| 0.877911997 | 7.866382856 | lysoPC.a.C17.0 (interacts with) PC.ae.C36.2 |
| 0.872418592 | 11.05827348 | lysoPC.a.C17.0 (interacts with) PC.aa.C42.6 |
| 0.865119488 | 5.574035637 | lysoPC.a.C17.0 (interacts with) PC.aa.C40.5 |
| 0.85807831 | 14.39104196 | lysoPC.a.C17.0 (interacts with) PC.ae.C34.0 |
| 0.851396467 | 6.290149425 | lysoPC.a.C17.0 (interacts with) PC.aa.C38.3 |
| 0.84610436 | 11.63433356 | lysoPC.a.C17.0 (interacts with) PC.aa.C34.4 |
| 0.844478323 | 14.93440054 | lysoPC.a.C17.0 (interacts with) SM.OH.C22.1 |
| 0.837122543 | 4.941048229 | lysoPC.a.C17.0 (interacts with) PC.aa.C32.3 |
| 0.837084687 | 6.45087077 | lysoPC.a.C17.0 (interacts with) PC.aa.C36.2 |
| 0.833573796 | 33.43200957 | lysoPC.a.C17.0 (interacts with) PC.ae.C38.0 |
| 0.831757807 | 9.541431649 | lysoPC.a.C17.0 (interacts with) PC.aa.C40.4 |
| 0.831510329 | 20.6343957 | lysoPC.a.C17.0 (interacts with) PC.ae.C42.2 |
| 0.827955075 | 6.320625916 | lysoPC.a.C17.0 (interacts with) SM.C24.0 |
| 0.817000239 | 7.028713374 | lysoPC.a.C17.0 (interacts with) PC.ae.C34.1 |
| 0.816216412 | 15.90566547 | lysoPC.a.C17.0 (interacts with) PC.aa.C38.5 |
| 0.815285101 | 17.06704361 | lysoPC.a.C17.0 (interacts with) PC.aa.C36.3 |
| 0.81456542 | 23.18770313 | lysoPC.a.C17.0 (interacts with) PC.aa.C36.1 |
| 0.810870865 | 8.902014246 | lysoPC.a.C17.0 (interacts with) PC.ae.C36.3 |
| 0.807846737 | 8.52284254 | lysoPC.a.C17.0 (interacts with) PC.aa.C28.1 |
| 0.939426538 | 4.534778942 | PC.ae.C42.3 (interacts with) SM.OH.C22.1 |
| 0.901792405 | 8.283986948 | PC.ae.C42.3 (interacts with) SM.C24.0 |
| 0.860514878 | 14.21587361 | PC.ae.C42.3 (interacts with) SM.OH.C16.1 |
| 0.85818593 | 11.30715205 | PC.ae.C42.3 (interacts with) SM.OH.C14.1 |
| 0.851065164 | 11.45000481 | PC.ae.C42.3 (interacts with) SM.OH.C24.1 |
| 0.848767532 | 11.07773824 | PC.ae.C42.3 (interacts with) SM.C16.1 |
| 0.841868985 | 22.57692592 | PC.ae.C42.3 (interacts with) SM.OH.C22.2 |
| 0.823573167 | 14.67704985 | PC.ae.C42.3 (interacts with) SM.C16.0 |
| 0.947929959 | 55.891368 | PC.ae.C42.5 (interacts with) PC.ae.C44.6 |
| 0.849487333 | 50.86582307 | PC.ae.C42.5 (interacts with) SM.C16.0 |
| 0.818282955 | 154.3074519 | PC.ae.C42.5 (interacts with) PC.ae.C44.5 |
| 0.817937285 | 54.05782682 | PC.ae.C42.5 (interacts with) SM.OH.C16.1 |
| 0.945824038 | 9.209603212 | PC.aa.C42.0 (interacts with) PC.ae.C42.5 |
| 0.910647355 | 13.57941569 | PC.aa.C42.0 (interacts with) PC.ae.C32.2 |
| 0.895533929 | 20.99250171 | PC.aa.C42.0 (interacts with) PC.ae.C32.1 |
| 0.886610143 | 19.42163787 | PC.aa.C42.0 (interacts with) SM.C16.0 |
| 0.863484805 | 105.8011801 | PC.aa.C42.0 (interacts with) PC.ae.C44.6 |
| 0.85921378 | 22.00105353 | PC.aa.C42.0 (interacts with) SM.OH.C16.1 |
| 0.84437388 | 27.10435351 | PC.aa.C42.0 (interacts with) SM.C16.1 |
| 0.841649867 | 10.62551022 | PC.aa.C42.0 (interacts with) PC.ae.C38.5 |
| 0.837127097 | 15.01438909 | PC.aa.C42.0 (interacts with) PC.ae.C40.2 |
| 0.836403468 | 12.36649189 | PC.aa.C42.0 (interacts with) PC.ae.C40.3 |
| 0.829495882 | 24.9246309 | PC.aa.C42.0 (interacts with) PC.ae.C40.4 |
| 0.825773546 | 25.30834353 | PC.aa.C42.0 (interacts with) PC.ae.C38.6 |
| 0.82484317 | 6.675063333 | PC.aa.C42.0 (interacts with) SM.C18.1 |
| 0.823755578 | 7.384965565 | PC.aa.C42.0 (interacts with) SM.C18.0 |
| 0.813364408 | 20.84916943 | PC.aa.C42.0 (interacts with) PC.ae.C36.5 |
| 0.81008186 | 27.4885964 | PC.aa.C42.0 (interacts with) SM.OH.C14.1 |
| 0.941867405 | 9.491379248 | PC.aa.C32.1 (interacts with) PC.aa.C34.1 |
| 0.912619986 | 12.16142367 | PC.aa.C32.1 (interacts with) PC.aa.C36.1 |
| 0.871546067 | 11.78453658 | PC.aa.C32.1 (interacts with) PC.aa.C32.2 |
| 0.850321914 | 12.51708651 | PC.aa.C32.1 (interacts with) PC.aa.C34.3 |
| 0.848120922 | 25.12621494 | PC.aa.C32.1 (interacts with) PC.aa.C36.3 |
| 0.831935066 | 32.26236103 | PC.aa.C32.1 (interacts with) PC.aa.C40.4 |
| 0.941527529 | 8.897898927 | PC.aa.C36.6 (interacts with) PC.ae.C38.0 |
| 0.913140192 | 8.151286817 | PC.aa.C36.6 (interacts with) PC.aa.C38.5 |
| 0.903098456 | 16.56105528 | PC.aa.C36.6 (interacts with) PC.aa.C42.6 |
| 0.852930081 | 9.272177533 | PC.aa.C36.6 (interacts with) PC.aa.C40.6 |
| 0.835781036 | 45.3380637 | PC.aa.C36.6 (interacts with) PC.ae.C40.1 |
| 0.834505362 | 47.27863277 | PC.aa.C36.6 (interacts with) PC.ae.C42.3 |
| 0.80623225 | 21.75975694 | PC.aa.C36.6 (interacts with) PC.ae.C40.5 |
| 0.918882779 | 11.51572014 | PC.ae.C38.0 (interacts with) PC.ae.C42.3 |
| 0.893817685 | 6.576151005 | PC.ae.C38.0 (interacts with) PC.ae.C40.6 |
| 0.890570974 | 4.562233017 | PC.ae.C38.0 (interacts with) PC.ae.C40.5 |
| 0.875026858 | 31.0243719 | PC.ae.C38.0 (interacts with) PC.ae.C40.1 |
| 0.869346191 | 10.03668275 | PC.ae.C38.0 (interacts with) SM.OH.C22.1 |
| 0.857402701 | 8.867602725 | PC.ae.C38.0 (interacts with) PC.ae.C38.6 |
| 0.8486893 | 6.801665783 | PC.ae.C38.0 (interacts with) SM.OH.C22.2 |
| 0.832541882 | 11.6682746 | PC.ae.C38.0 (interacts with) PC.ae.C42.2 |
| 0.817874289 | 9.366621339 | PC.ae.C38.0 (interacts with) PC.ae.C40.2 |
| 0.809208033 | 32.50434411 | PC.ae.C38.0 (interacts with) PC.ae.C38.3 |
| 0.806750456 | 19.35157673 | PC.ae.C38.0 (interacts with) SM.C24.0 |
| 0.939662683 | 4.665516048 | PC.ae.C32.1 (interacts with) SM.C16.0 |
| 0.92398462 | 4.578315555 | PC.ae.C32.1 (interacts with) PC.ae.C42.4 |
| 0.920645003 | 5.044186973 | PC.ae.C32.1 (interacts with) PC.ae.C32.2 |
| 0.910289521 | 3.500374536 | PC.ae.C32.1 (interacts with) SM.OH.C14.1 |
| 0.907362891 | 4.526521503 | PC.ae.C32.1 (interacts with) SM.OH.C16.1 |
| 0.906020346 | 7.195357618 | PC.ae.C32.1 (interacts with) PC.ae.C38.4 |
| 0.898719355 | 5.615138595 | PC.ae.C32.1 (interacts with) PC.ae.C34.2 |
| 0.895564464 | 3.239873598 | PC.ae.C32.1 (interacts with) PC.ae.C36.4 |
| 0.882893019 | 4.047402509 | PC.ae.C32.1 (interacts with) PC.ae.C40.4 |
| 0.881159127 | 6.578724275 | PC.ae.C32.1 (interacts with) SM.C16.1 |
| 0.880228185 | 39.3659592 | PC.ae.C32.1 (interacts with) PC.ae.C42.5 |
| 0.874567626 | 9.270812993 | PC.ae.C32.1 (interacts with) PC.ae.C42.2 |
| 0.872148439 | 21.10971527 | PC.ae.C32.1 (interacts with) PC.ae.C34.1 |
| 0.865771706 | 4.121288212 | PC.ae.C32.1 (interacts with) PC.ae.C40.3 |
| 0.859297958 | 11.53086987 | PC.ae.C32.1 (interacts with) PC.ae.C34.0 |
| 0.852558101 | 7.489040813 | PC.ae.C32.1 (interacts with) PC.ae.C38.5 |
| 0.847829995 | 14.66235767 | PC.ae.C32.1 (interacts with) PC.ae.C34.3 |
| 0.845955621 | 4.1833245 | PC.ae.C32.1 (interacts with) SM.OH.C24.1 |
| 0.842476293 | 7.756782846 | PC.ae.C32.1 (interacts with) PC.ae.C36.5 |
| 0.835936796 | 21.12539212 | PC.ae.C32.1 (interacts with) PC.ae.C38.3 |
| 0.830081769 | 10.81699492 | PC.ae.C32.1 (interacts with) SM.OH.C22.1 |
| 0.821964845 | 13.11744313 | PC.ae.C32.1 (interacts with) SM.C18.0 |
| 0.816715251 | 10.52855138 | PC.ae.C32.1 (interacts with) PC.ae.C36.2 |
| 0.81613332 | 15.89749605 | PC.ae.C32.1 (interacts with) PC.ae.C36.3 |
| 0.938169328 | 7.690781735 | PC.ae.C36.4 (interacts with) PC.ae.C38.4 |
| 0.904982918 | 8.93709242 | PC.ae.C36.4 (interacts with) PC.ae.C38.5 |
| 0.889165147 | 9.269877578 | PC.ae.C36.4 (interacts with) PC.ae.C36.5 |
| 0.86578872 | 4.638997776 | PC.ae.C36.4 (interacts with) SM.OH.C24.1 |
| 0.864397174 | 6.483912275 | PC.ae.C36.4 (interacts with) SM.C16.0 |
| 0.859182641 | 8.387645433 | PC.ae.C36.4 (interacts with) SM.C16.1 |
| 0.857423079 | 5.037575216 | PC.ae.C36.4 (interacts with) PC.ae.C40.3 |
| 0.845834952 | 5.944388956 | PC.ae.C36.4 (interacts with) PC.ae.C40.4 |
| 0.837514141 | 4.004854415 | PC.ae.C36.4 (interacts with) PC.ae.C42.4 |
| 0.827475365 | 6.55651385 | PC.ae.C36.4 (interacts with) SM.OH.C16.1 |
| 0.816450084 | 5.158785054 | PC.ae.C36.4 (interacts with) SM.OH.C14.1 |
| 0.812563866 | 24.70473923 | PC.ae.C36.4 (interacts with) PC.ae.C42.5 |
| 0.809860306 | 28.70368054 | PC.ae.C36.4 (interacts with) PC.ae.C38.3 |
| 0.800360884 | 14.52086447 | PC.ae.C36.4 (interacts with) PC.ae.C42.2 |
| 0.921528335 | 3.331070155 | PC.ae.C38.4 (interacts with) SM.OH.C24.1 |
| 0.91373438 | 7.531783341 | PC.ae.C38.4 (interacts with) PC.ae.C40.4 |
| 0.90435779 | 9.099363502 | PC.ae.C38.4 (interacts with) SM.OH.C16.1 |
| 0.903697476 | 15.15761036 | PC.ae.C38.4 (interacts with) PC.ae.C38.5 |
| 0.901485818 | 6.329394627 | PC.ae.C38.4 (interacts with) SM.OH.C14.1 |
| 0.898847078 | 9.02167517 | PC.ae.C38.4 (interacts with) PC.ae.C40.3 |
| 0.893999415 | 8.183668293 | PC.ae.C38.4 (interacts with) SM.C16.0 |
| 0.888050008 | 7.035314995 | PC.ae.C38.4 (interacts with) SM.C16.1 |
| 0.884963983 | 9.910201963 | PC.ae.C38.4 (interacts with) PC.ae.C42.4 |
| 0.881102335 | 6.748597313 | PC.ae.C38.4 (interacts with) PC.ae.C42.2 |
| 0.867566758 | 7.216831263 | PC.ae.C38.4 (interacts with) SM.OH.C22.1 |
| 0.847896521 | 10.48800722 | PC.ae.C38.4 (interacts with) PC.ae.C42.3 |
| 0.805560051 | 7.087843062 | PC.ae.C38.4 (interacts with) SM.C24.0 |
| 0.802478206 | 16.9459509 | PC.ae.C38.4 (interacts with) PC.ae.C40.5 |
| 0.934465177 | 3.045741164 | SM.C18.0 (interacts with) SM.C18.1 |
| 0.835767966 | 12.11830932 | SM.C18.0 (interacts with) SM.C24.1 |
| 0.91847153 | 5.651875902 | PC.aa.C34.4 (interacts with) PC.aa.C36.3 |
| 0.905847769 | 4.663193795 | PC.aa.C34.4 (interacts with) PC.aa.C40.4 |
| 0.889967016 | 5.757243082 | PC.aa.C34.4 (interacts with) PC.aa.C36.1 |
| 0.889304315 | 5.40897047 | PC.aa.C34.4 (interacts with) PC.aa.C38.3 |
| 0.880854386 | 15.01847037 | PC.aa.C34.4 (interacts with) PC.ae.C40.1 |
| 0.880796468 | 5.025554033 | PC.aa.C34.4 (interacts with) PC.aa.C36.2 |
| 0.857355192 | 10.56027311 | PC.aa.C34.4 (interacts with) PC.aa.C40.5 |
| 0.829295399 | 20.13987712 | PC.aa.C34.4 (interacts with) PC.ae.C36.1 |
| 0.814737824 | 12.35547944 | PC.aa.C34.4 (interacts with) PC.aa.C36.4 |
| 0.805654004 | 53.29056875 | PC.aa.C34.4 (interacts with) PC.ae.C38.3 |
| 0.936759351 | 4.243859337 | PC.aa.C32.3 (interacts with) PC.aa.C36.2 |
| 0.923503661 | 7.105554947 | PC.aa.C32.3 (interacts with) PC.ae.C36.2 |
| 0.922695277 | 6.252948949 | PC.aa.C32.3 (interacts with) PC.ae.C34.3 |
| 0.92031345 | 6.198348018 | PC.aa.C32.3 (interacts with) PC.ae.C36.3 |
| 0.920207979 | 6.010557876 | PC.aa.C32.3 (interacts with) PC.ae.C30.0 |
| 0.91122962 | 4.503481366 | PC.aa.C32.3 (interacts with) PC.ae.C36.1 |
| 0.907306582 | 10.7709738 | PC.aa.C32.3 (interacts with) PC.aa.C34.3 |
| 0.907160969 | 7.442457514 | PC.aa.C32.3 (interacts with) PC.aa.C34.2 |
| 0.903016424 | 9.052970488 | PC.aa.C32.3 (interacts with) PC.aa.C34.4 |
| 0.893782919 | 9.093590516 | PC.aa.C32.3 (interacts with) PC.ae.C38.3 |
| 0.886144899 | 5.738744131 | PC.aa.C32.3 (interacts with) PC.ae.C34.1 |
| 0.878803402 | 21.43849275 | PC.aa.C32.3 (interacts with) PC.ae.C42.3 |
| 0.874480732 | 12.09493439 | PC.aa.C32.3 (interacts with) PC.aa.C36.3 |
| 0.869086794 | 11.25118481 | PC.aa.C32.3 (interacts with) PC.ae.C34.2 |
| 0.864048752 | 6.328582542 | PC.aa.C32.3 (interacts with) PC.ae.C40.1 |
| 0.861033898 | 21.14923048 | PC.aa.C32.3 (interacts with) PC.ae.C34.0 |
| 0.839659996 | 44.64013772 | PC.aa.C32.3 (interacts with) PC.ae.C38.2 |
| 0.837051135 | 7.192857261 | PC.aa.C32.3 (interacts with) PC.aa.C40.4 |
| 0.830024009 | 8.857086725 | PC.aa.C32.3 (interacts with) SM.C24.0 |
| 0.827052319 | 15.67743905 | PC.aa.C32.3 (interacts with) PC.aa.C36.1 |
| 0.818831398 | 23.24095684 | PC.aa.C32.3 (interacts with) SM.OH.C22.1 |
| 0.814618327 | 6.543071412 | PC.aa.C32.3 (interacts with) PC.aa.C38.3 |
| 0.806549329 | 15.81178289 | PC.aa.C32.3 (interacts with) PC.ae.C38.4 |
| 0.805196977 | 7.029276069 | PC.aa.C32.3 (interacts with) PC.aa.C40.5 |
| 0.935667408 | 3.742771317 | PC.ae.C40.3 (interacts with) PC.ae.C40.4 |
| 0.9303904 | 3.307116754 | PC.ae.C40.3 (interacts with) SM.OH.C16.1 |
| 0.907879614 | 3.33891243 | PC.ae.C40.3 (interacts with) SM.C16.0 |
| 0.900357465 | 4.087626142 | PC.ae.C40.3 (interacts with) SM.OH.C14.1 |
| 0.87698248 | 9.102534742 | PC.ae.C40.3 (interacts with) PC.ae.C40.5 |
| 0.870709476 | 5.461730387 | PC.ae.C40.3 (interacts with) SM.OH.C24.1 |
| 0.865194006 | 4.564547956 | PC.ae.C40.3 (interacts with) SM.C16.1 |
| 0.85966602 | 30.71520113 | PC.ae.C40.3 (interacts with) PC.ae.C42.5 |
| 0.853992527 | 8.341161165 | PC.ae.C40.3 (interacts with) SM.OH.C22.2 |
| 0.853408846 | 9.889801524 | PC.ae.C40.3 (interacts with) SM.OH.C22.1 |
| 0.839649444 | 15.87843171 | PC.ae.C40.3 (interacts with) PC.ae.C42.3 |
| 0.835077329 | 7.449444427 | PC.ae.C40.3 (interacts with) SM.C18.0 |
| 0.823933563 | 7.782287149 | PC.ae.C40.3 (interacts with) PC.ae.C42.2 |
| 0.808480946 | 9.501859161 | PC.ae.C40.3 (interacts with) PC.ae.C42.4 |
| 0.807431646 | 12.14352827 | PC.ae.C40.3 (interacts with) PC.ae.C40.6 |
| 0.929711508 | 11.40510892 | PC.ae.C40.4 (interacts with) PC.ae.C40.5 |
| 0.892896399 | 13.32847669 | PC.ae.C40.4 (interacts with) PC.ae.C42.3 |
| 0.887354146 | 3.879616473 | PC.ae.C40.4 (interacts with) SM.OH.C16.1 |
| 0.881288046 | 4.232499907 | PC.ae.C40.4 (interacts with) SM.C16.0 |
| 0.861264159 | 3.317482517 | PC.ae.C40.4 (interacts with) SM.OH.C14.1 |
| 0.859288051 | 8.595933184 | PC.ae.C40.4 (interacts with) SM.OH.C22.1 |
| 0.859229622 | 52.56039894 | PC.ae.C40.4 (interacts with) PC.ae.C42.5 |
| 0.85252377 | 7.403876381 | PC.ae.C40.4 (interacts with) PC.ae.C42.2 |
| 0.848322407 | 5.57385011 | PC.ae.C40.4 (interacts with) SM.C16.1 |
| 0.842704842 | 4.496148283 | PC.ae.C40.4 (interacts with) SM.OH.C24.1 |
| 0.820034073 | 8.484885674 | PC.ae.C40.4 (interacts with) PC.ae.C42.4 |
| 0.811277945 | 13.20136826 | PC.ae.C40.4 (interacts with) SM.OH.C22.2 |
| 0.93247341 | 4.10958765 | PC.ae.C40.5 (interacts with) PC.ae.C40.6 |
| 0.918788375 | 12.13749424 | PC.ae.C40.5 (interacts with) PC.ae.C42.3 |
| 0.891422194 | 3.624036578 | PC.ae.C40.5 (interacts with) SM.OH.C22.2 |
| 0.88377094 | 9.055708782 | PC.ae.C40.5 (interacts with) SM.OH.C22.1 |
| 0.863096012 | 9.671234847 | PC.ae.C40.5 (interacts with) SM.OH.C16.1 |
| 0.857708697 | 8.179085063 | PC.ae.C40.5 (interacts with) PC.ae.C42.2 |
| 0.840141236 | 6.961779076 | PC.ae.C40.5 (interacts with) SM.C16.1 |
| 0.839122856 | 9.244436256 | PC.ae.C40.5 (interacts with) SM.C16.0 |
| 0.819424119 | 10.77108475 | PC.ae.C40.5 (interacts with) SM.OH.C14.1 |
| 0.93206804 | 2.835859316 | PC.ae.C32.2 (interacts with) PC.ae.C40.3 |
| 0.929849304 | 2.723657252 | PC.ae.C32.2 (interacts with) SM.C16.0 |
| 0.927553702 | 2.674796567 | PC.ae.C32.2 (interacts with) SM.OH.C16.1 |
| 0.924209656 | 4.408393055 | PC.ae.C32.2 (interacts with) PC.ae.C40.4 |
| 0.91684474 | 3.654895862 | PC.ae.C32.2 (interacts with) PC.ae.C36.5 |
| 0.915675796 | 2.923876841 | PC.ae.C32.2 (interacts with) PC.ae.C38.5 |
| 0.912867706 | 3.478233789 | PC.ae.C32.2 (interacts with) SM.C16.1 |
| 0.90463104 | 8.12274453 | PC.ae.C32.2 (interacts with) PC.ae.C40.5 |
| 0.899295751 | 5.367465853 | PC.ae.C32.2 (interacts with) PC.ae.C40.2 |
| 0.896086354 | 4.138971582 | PC.ae.C32.2 (interacts with) SM.OH.C14.1 |
| 0.892657316 | 9.042335621 | PC.ae.C32.2 (interacts with) PC.ae.C38.6 |
| 0.880828031 | 6.678626985 | PC.ae.C32.2 (interacts with) PC.ae.C42.2 |
| 0.878607237 | 9.478902431 | PC.ae.C32.2 (interacts with) PC.ae.C34.0 |
| 0.877256306 | 10.41450789 | PC.ae.C32.2 (interacts with) PC.ae.C40.6 |
| 0.874711732 | 38.09417556 | PC.ae.C32.2 (interacts with) PC.ae.C42.5 |
| 0.873603456 | 6.9812628 | PC.ae.C32.2 (interacts with) SM.OH.C22.2 |
| 0.865872989 | 10.04033935 | PC.ae.C32.2 (interacts with) PC.ae.C38.4 |
| 0.865462669 | 8.9986975 | PC.ae.C32.2 (interacts with) SM.OH.C22.1 |
| 0.858492205 | 6.295617713 | PC.ae.C32.2 (interacts with) PC.ae.C36.4 |
| 0.85580563 | 15.47479009 | PC.ae.C32.2 (interacts with) PC.ae.C42.3 |
| 0.845411546 | 7.494873771 | PC.ae.C32.2 (interacts with) SM.C18.0 |
| 0.839139257 | 11.05145719 | PC.ae.C32.2 (interacts with) PC.ae.C34.2 |
| 0.833907065 | 5.974578144 | PC.ae.C32.2 (interacts with) SM.OH.C24.1 |
| 0.828307921 | 31.19018688 | PC.ae.C32.2 (interacts with) PC.ae.C38.3 |
| 0.820699364 | 9.490297347 | PC.ae.C32.2 (interacts with) SM.C18.1 |
| 0.808479112 | 18.32265127 | PC.ae.C32.2 (interacts with) PC.ae.C36.2 |
| 0.930930149 | 9.327511164 | PC.aa.C36.0 (interacts with) PC.aa.C38.0 |
| 0.928556087 | 7.839757239 | PC.aa.C36.0 (interacts with) PC.aa.C38.1 |
| 0.92177733 | 14.26345824 | PC.aa.C36.0 (interacts with) PC.ae.C40.6 |
| 0.913822555 | 17.15748612 | PC.aa.C36.0 (interacts with) PC.ae.C38.6 |
| 0.837655574 | 6.331463857 | PC.aa.C36.0 (interacts with) SM.C24.1 |
| 0.836621761 | 28.23806607 | PC.aa.C36.0 (interacts with) PC.ae.C40.5 |
| 0.830797926 | 17.95489706 | PC.aa.C36.0 (interacts with) PC.aa.C38.6 |
| 0.827116944 | 35.64446274 | PC.aa.C36.0 (interacts with) PC.ae.C38.0 |
| 0.804091826 | 18.56866895 | PC.aa.C36.0 (interacts with) SM.OH.C22.2 |
| 0.802372809 | 9.83387819 | PC.aa.C36.0 (interacts with) PC.aa.C36.5 |
| 0.801284769 | 17.31401643 | PC.aa.C36.0 (interacts with) PC.aa.C36.6 |
| 0.928921863 | 5.692548106 | PC.ae.C44.5 (interacts with) PC.ae.C44.6 |
| 0.844212719 | 5.95090337 | PC.aa.C38.1 (interacts with) SM.C24.1 |
| 0.835036624 | 61.17837251 | PC.aa.C38.1 (interacts with) PC.ae.C40.6 |
| 0.818823593 | 64.75802713 | PC.aa.C38.1 (interacts with) PC.ae.C38.6 |
| 0.926773632 | 13.75110602 | PC.aa.C30.0 (interacts with) PC.aa.C32.0 |
| 0.917815483 | 7.747348733 | PC.aa.C30.0 (interacts with) PC.ae.C34.1 |
| 0.917086842 | 4.892232724 | PC.aa.C30.0 (interacts with) PC.aa.C32.2 |
| 0.915511398 | 8.37485087 | PC.aa.C30.0 (interacts with) PC.ae.C30.0 |
| 0.907194832 | 6.059405397 | PC.aa.C30.0 (interacts with) PC.aa.C32.3 |
| 0.905509776 | 3.798751797 | PC.aa.C30.0 (interacts with) PC.aa.C40.4 |
| 0.905266358 | 6.505455864 | PC.aa.C30.0 (interacts with) PC.aa.C40.5 |
| 0.903279038 | 6.993937031 | PC.aa.C30.0 (interacts with) PC.aa.C36.1 |
| 0.901575887 | 5.261692657 | PC.aa.C30.0 (interacts with) PC.aa.C34.1 |
| 0.893295541 | 5.480626365 | PC.aa.C30.0 (interacts with) PC.aa.C34.4 |
| 0.889046336 | 4.823496804 | PC.aa.C30.0 (interacts with) PC.aa.C38.3 |
| 0.887773406 | 7.099369794 | PC.aa.C30.0 (interacts with) PC.ae.C36.1 |
| 0.887138152 | 3.798368709 | PC.aa.C30.0 (interacts with) PC.aa.C36.2 |
| 0.879749681 | 5.159122162 | PC.aa.C30.0 (interacts with) PC.aa.C34.3 |
| 0.865419641 | 15.76918442 | PC.aa.C30.0 (interacts with) SM.C24.0 |
| 0.863701376 | 6.401472802 | PC.aa.C30.0 (interacts with) PC.aa.C36.3 |
| 0.851251223 | 8.108531058 | PC.aa.C30.0 (interacts with) PC.ae.C34.3 |
| 0.850499818 | 3.976899874 | PC.aa.C30.0 (interacts with) PC.aa.C34.2 |
| 0.845092048 | 19.34685952 | PC.aa.C30.0 (interacts with) PC.ae.C38.3 |
| 0.838219641 | 47.19185907 | PC.aa.C30.0 (interacts with) PC.aa.C32.1 |
| 0.833991779 | 61.58093027 | PC.aa.C30.0 (interacts with) PC.ae.C34.0 |
| 0.804232433 | 7.761282522 | PC.aa.C30.0 (interacts with) PC.ae.C36.3 |
| 0.925615312 | 5.801450486 | PC.aa.C42.5 (interacts with) PC.aa.C42.6 |
| 0.913769368 | 4.526795874 | PC.aa.C42.5 (interacts with) PC.ae.C34.0 |
| 0.913131683 | 4.100160091 | PC.aa.C42.5 (interacts with) SM.OH.C22.1 |
| 0.893970418 | 4.150677547 | PC.aa.C42.5 (interacts with) PC.ae.C42.2 |
| 0.88979614 | 9.705236397 | PC.aa.C42.5 (interacts with) SM.C24.0 |
| 0.877704929 | 6.265789348 | PC.aa.C42.5 (interacts with) SM.C16.0 |
| 0.85900467 | 6.428285185 | PC.aa.C42.5 (interacts with) SM.OH.C16.1 |
| 0.858638152 | 21.52250645 | PC.aa.C42.5 (interacts with) PC.ae.C36.1 |
| 0.858573883 | 13.65709233 | PC.aa.C42.5 (interacts with) PC.ae.C38.3 |
| 0.853861984 | 6.992353464 | PC.aa.C42.5 (interacts with) PC.ae.C42.3 |
| 0.845540818 | 4.897493076 | PC.aa.C42.5 (interacts with) PC.ae.C40.5 |
| 0.84548565 | 5.952344676 | PC.aa.C42.5 (interacts with) SM.OH.C14.1 |
| 0.840835006 | 6.860971821 | PC.aa.C42.5 (interacts with) PC.ae.C40.2 |
| 0.832952686 | 6.294857722 | PC.aa.C42.5 (interacts with) PC.ae.C38.0 |
| 0.832328884 | 4.732116533 | PC.aa.C42.5 (interacts with) SM.C16.1 |
| 0.820964457 | 18.31044912 | PC.aa.C42.5 (interacts with) PC.ae.C34.1 |
| 0.819194421 | 7.525095107 | PC.aa.C42.5 (interacts with) PC.ae.C38.5 |
| 0.816264655 | 4.261025312 | PC.aa.C42.5 (interacts with) PC.ae.C36.5 |
| 0.815218616 | 8.912277623 | PC.aa.C42.5 (interacts with) PC.ae.C40.6 |
| 0.81510714 | 6.241254686 | PC.aa.C42.5 (interacts with) SM.OH.C22.2 |
| 0.814449356 | 9.313742825 | PC.aa.C42.5 (interacts with) PC.ae.C38.6 |
| 0.813917469 | 15.20888759 | PC.aa.C42.5 (interacts with) PC.ae.C30.0 |
| 0.811310084 | 6.261834218 | PC.aa.C42.5 (interacts with) SM.OH.C24.1 |
| 0.803530547 | 6.622900397 | PC.aa.C42.5 (interacts with) PC.ae.C32.2 |
| 0.925093134 | 10.43308993 | PC.ae.C36.0 (interacts with) PC.ae.C40.5 |
| 0.8356296 | 11.25152923 | PC.ae.C36.0 (interacts with) PC.ae.C40.6 |
| 0.829375681 | 26.73519122 | PC.ae.C36.0 (interacts with) PC.ae.C40.4 |
| 0.816752598 | 33.86467656 | PC.ae.C36.0 (interacts with) PC.ae.C42.3 |
| 0.816139272 | 14.49872242 | PC.ae.C36.0 (interacts with) PC.ae.C40.2 |
| 0.80512829 | 10.15006402 | PC.ae.C36.0 (interacts with) PC.ae.C38.0 |
| 0.924927193 | 2 | Leu (interacts with) Val |
| 0.830309582 | 28.81205741 | PC.ae.C42.4 (interacts with) PC.ae.C42.5 |
| 0.826497111 | 8.231740136 | PC.ae.C42.4 (interacts with) SM.OH.C14.1 |
| 0.807434577 | 13.23010516 | PC.ae.C42.4 (interacts with) SM.C16.0 |
| 0.804779991 | 13.02557056 | PC.ae.C42.4 (interacts with) SM.OH.C16.1 |
| 0.800834072 | 7.241691328 | PC.ae.C42.4 (interacts with) SM.OH.C24.1 |
| 0.922284098 | 16.40614073 | PC.ae.C40.1 (interacts with) PC.ae.C42.3 |
| 0.843629839 | 8.869524554 | PC.ae.C40.1 (interacts with) SM.C24.0 |
| 0.821545969 | 19.84847137 | PC.ae.C40.1 (interacts with) SM.OH.C22.1 |
| 0.891899986 | 4.970334162 | SM.OH.C24.1 (interacts with) SM.C16.1 |
| 0.883826214 | 5.465459889 | SM.OH.C24.1 (interacts with) SM.C16.0 |
| 0.87526579 | 8.557375444 | SM.OH.C24.1 (interacts with) SM.C24.0 |
| 0.919383782 | 2.666666667 | C2 (interacts with) C14.1 |
| 0.881352832 | 2.666666667 | C2 (interacts with) C12.1 |
| 0.870486724 | 2.666666667 | C2 (interacts with) C14.2 |
| 0.910834322 | 5.582716049 | lysoPC.a.C16.1 (interacts with) lysoPC.a.C18.1 |
| 0.889916897 | 154.417284 | lysoPC.a.C16.1 (interacts with) lysoPC.a.C20.3 |
| 0.887160354 | 102.4086351 | lysoPC.a.C18.1 (interacts with) lysoPC.a.C18.2 |
| 0.870205722 | 211.174081 | lysoPC.a.C18.1 (interacts with) lysoPC.a.C20.3 |
| 0.810314094 | 160 | lysoPC.a.C18.1 (interacts with) lysoPC.a.C20.4 |
| 0.893484613 | 18.94115511 | lysoPC.a.C20.3 (interacts with) PC.aa.C34.1 |
| 0.882866064 | 23.81409285 | lysoPC.a.C20.3 (interacts with) PC.aa.C36.1 |
| 0.878845478 | 51.77317564 | lysoPC.a.C20.3 (interacts with) PC.aa.C40.4 |
| 0.875406005 | 41.04871396 | lysoPC.a.C20.3 (interacts with) PC.aa.C36.3 |
| 0.863275097 | 10.63685611 | lysoPC.a.C20.3 (interacts with) PC.aa.C32.1 |
| 0.846440618 | 75.35349336 | lysoPC.a.C20.3 (interacts with) PC.aa.C38.3 |
| 0.818191979 | 21.37960395 | lysoPC.a.C20.3 (interacts with) PC.aa.C34.3 |
| 0.81620356 | 157.9279065 | lysoPC.a.C20.3 (interacts with) PC.aa.C40.5 |
| 0.810663577 | 22.02775529 | lysoPC.a.C20.3 (interacts with) PC.aa.C32.2 |
| 0.889362021 | 2.666666667 | C10.1 (interacts with) C12.1 |
| 0.876877368 | 2.666666667 | C10.1 (interacts with) C14.2 |
| 0.8378811 | 2.666666667 | C10.1 (interacts with) C14.1 |
| 0.882854171 | 18.51289566 | lysoPC.a.C18.2 (interacts with) PC.aa.C34.3 |
| 0.85129729 | 35.36644578 | lysoPC.a.C18.2 (interacts with) PC.aa.C36.3 |
| 0.836556617 | 84.59123244 | lysoPC.a.C18.2 (interacts with) PC.aa.C36.2 |
| 0.824266084 | 7.072226512 | lysoPC.a.C18.2 (interacts with) lysoPC.a.C20.3 |
| 0.811605034 | 18.7518209 | lysoPC.a.C18.2 (interacts with) PC.aa.C32.2 |
| 0.804860955 | 43.5798625 | lysoPC.a.C18.2 (interacts with) PC.aa.C34.2 |
| 0.80316777 | 24.40576013 | lysoPC.a.C18.2 (interacts with) PC.aa.C34.4 |
| 0.874737664 | 28.27518063 | PC.ae.C38.2 (interacts with) PC.ae.C40.1 |
| 0.803705049 | 87.08468165 | PC.ae.C38.2 (interacts with) PC.ae.C42.3 |
| 0.867588099 | 4 | C14 (interacts with) C18.1 |
| 0.817381344 | 4 | C14 (interacts with) C18.2 |
| 0.857604421 | 2 | Ile (interacts with) Val |
| 0.834694058 | 2 | Ile (interacts with) Leu |
| 0.85576409 | 6 | Arg (interacts with) Met |
| 0.853292244 | 6 | Met (interacts with) Thr |
| 0.801399868 | 6 | Met (interacts with) Tyr |
| 0.849913381 | 160 | C3 (interacts with) Trp |
| 0.827156814 | 91.82925971 | Trp (interacts with) lysoPC.a.C16.0 |
| 0.801134278 | 150.1607943 | Trp (interacts with) PC.aa.C36.3 |
| 0.800939708 | 74.00994603 | Trp (interacts with) PC.aa.C36.1 |
| 0.837823445 | 4 | Cit (interacts with) total.DMA |
| 0.833064383 | 4 | C0 (interacts with) Lys |
| 0.829054619 | 4 | His (interacts with) Lys |

Supplementary Table 13. Network parameters from advanced lung adenocarcinoma group

| Average Shortest  PathLength | Betweenness Centrality | Closeness Centrality | Clustering Coefficient | Degree | Eccentricity | Metabolite | Neighborhood Connectivity | Number Of  Undirected Edges |
| --- | --- | --- | --- | --- | --- | --- | --- | --- |
| 3.666666667 | 0.007278206 | 0.272727273 | 0.560606061 | 12 | 8 | PC.aa.C34.1 | 9.083333333 | 12 |
| 3.956989247 | 0.003714054 | 0.252717391 | 0.545454545 | 11 | 8 | PC.aa.C36.1 | 8.727272727 | 11 |
| 2.956989247 | 0.008640853 | 0.338181818 | 0.593406593 | 14 | 6 | PC.aa.C38.0 | 12.92857143 | 14 |
| 2.860215054 | 0.038618774 | 0.34962406 | 0.491666667 | 16 | 5 | PC.ae.C40.6 | 11.8125 | 16 |
| 2.602150538 | 0.109554993 | 0.384297521 | 0.454545455 | 12 | 6 | PC.ae.C34.2 | 12.33333333 | 12 |
| 2.505376344 | 0.037964466 | 0.399141631 | 0.516666667 | 16 | 6 | PC.ae.C36.3 | 13.5 | 16 |
| 1 | 0.277777778 | 1 | 0.476190476 | 7 | 1 | C12.1 | 3.857142857 | 7 |
| 1.142857143 | 0.111111111 | 0.875 | 0.6 | 6 | 2 | C14.1 | 4.333333333 | 6 |
| 3.989247312 | 0.002653085 | 0.250673854 | 0.611111111 | 9 | 8 | PC.aa.C40.5 | 8.777777778 | 9 |
| 3.903225806 | 0.001582081 | 0.256198347 | 0.714285714 | 7 | 8 | PC.aa.C32.1 | 9.428571429 | 7 |
| 2.76344086 | 0.027760688 | 0.361867704 | 0.527777778 | 9 | 6 | PC.ae.C40.3 | 13.66666667 | 9 |
| 2.655913978 | 0.035864425 | 0.376518219 | 0.417582418 | 14 | 6 | PC.ae.C42.5 | 12.71428571 | 14 |
| 1.285714286 | 0.087301587 | 0.777777778 | 0.6 | 5 | 2 | C14.2 | 4.6 | 5 |
| 2.720430108 | 0.033908443 | 0.367588933 | 0.575757576 | 12 | 6 | PC.ae.C36.4 | 13.5 | 12 |
| 2.548387097 | 0.041121401 | 0.392405063 | 0.516483516 | 14 | 6 | PC.ae.C36.5 | 13.64285714 | 14 |
| 2.301075269 | 0.060990123 | 0.434579439 | 0.471428571 | 21 | 5 | PC.ae.C38.5 | 14.33333333 | 21 |
| 3.752688172 | 0.003115482 | 0.266475645 | 0.678571429 | 8 | 8 | lysoPC.a.C16.1 | 9.375 | 8 |
| 3.838709677 | 0.007411299 | 0.260504202 | 0.555555556 | 10 | 8 | lysoPC.a.C20.3 | 9.1 | 10 |
| 3.462365591 | 0.005478841 | 0.288819876 | 0.8 | 5 | 7 | PC.aa.C36.5 | 10.6 | 5 |
| 3.440860215 | 0.00712959 | 0.290625 | 0.619047619 | 7 | 7 | PC.aa.C38.5 | 8.571428571 | 7 |
| 3.365591398 | 8.37E-04 | 0.297124601 | 0.8 | 5 | 6 | PC.aa.C42.0 | 8.8 | 5 |
| 2.817204301 | 0.025325908 | 0.354961832 | 0.428571429 | 7 | 6 | PC.ae.C42.4 | 12.42857143 | 7 |
| 2.430107527 | 0.030340591 | 0.411504425 | 0.558823529 | 17 | 6 | PC.ae.C38.3 | 15.35294118 | 17 |
| 2.516129032 | 0.059408574 | 0.397435897 | 0.45 | 16 | 6 | PC.ae.C38.4 | 13.75 | 16 |
| 2.870967742 | 0.026840741 | 0.348314607 | 0.458333333 | 16 | 6 | PC.ae.C40.5 | 12.25 | 16 |
| 3.47311828 | 0.002069298 | 0.287925697 | 0.666666667 | 6 | 7 | PC.ae.C44.5 | 8 | 6 |
| 3.35483871 | 0.007023273 | 0.298076923 | 0.666666667 | 6 | 6 | PC.ae.C44.6 | 7.666666667 | 6 |
| 2.720430108 | 0.008522982 | 0.367588933 | 0.666666667 | 12 | 6 | PC.ae.C34.3 | 14.33333333 | 12 |
| 3.075268817 | 0.026269617 | 0.325174825 | 0.454545455 | 11 | 7 | PC.ae.C42.3 | 11.18181818 | 11 |
| 3.913978495 | 0.010967309 | 0.255494505 | 0.733333333 | 6 | 8 | C4 | 5.666666667 | 6 |
| 3.913978495 | 0.010967309 | 0.255494505 | 0.733333333 | 6 | 8 | Creatinine | 5.666666667 | 6 |
| 2.376344086 | 0.105863603 | 0.42081448 | 0.525641026 | 13 | 6 | PC.ae.C36.1 | 16.53846154 | 13 |
| 3.548387097 | 0.020124277 | 0.281818182 | 0.535714286 | 8 | 8 | lysoPC.a.C16.0 | 8.75 | 8 |
| 3.47311828 | 0.026450903 | 0.287925697 | 0.527777778 | 9 | 8 | lysoPC.a.C18.1 | 9.111111111 | 9 |
| 3.344086022 | 0.007597426 | 0.29903537 | 0.714285714 | 7 | 7 | PC.aa.C38.3 | 11 | 7 |
| 3.258064516 | 0.020182891 | 0.306930693 | 0.5 | 12 | 7 | PC.aa.C40.4 | 9.333333333 | 12 |
| 3.655913978 | 0.043081563 | 0.273529412 | 0.523809524 | 7 | 7 | total.DMA | 5 | 7 |
| 2.935483871 | 0.029099919 | 0.340659341 | 0.461538462 | 13 | 5 | SM.OH.C22.1 | 10.69230769 | 13 |
| 3.35483871 | 0.001967322 | 0.298076923 | 0.638888889 | 9 | 6 | SM.OH.C24.1 | 11.44444444 | 9 |
| 3.935483871 | 0.063113604 | 0.254098361 | 0.333333333 | 4 | 7 | Ile | 5.25 | 4 |
| 4.892473118 | 0.010635811 | 0.204395604 | 0.666666667 | 3 | 8 | Leu | 3 | 3 |
| 2.64516129 | 0.050040555 | 0.37804878 | 0.457142857 | 15 | 6 | PC.ae.C40.4 | 14.4 | 15 |
| 3.290322581 | 0.042202817 | 0.303921569 | 0.666666667 | 6 | 7 | t4.OH.Pro | 7 | 6 |
| 1.285714286 | 0.047619048 | 0.777777778 | 0.7 | 5 | 2 | C2 | 4.8 | 5 |
| 3.075268817 | 0.037618207 | 0.325174825 | 0.5 | 9 | 7 | PC.aa.C32.0 | 9.777777778 | 9 |
| 2.903225806 | 0.031627641 | 0.344444444 | 0.472222222 | 9 | 7 | PC.aa.C32.2 | 10.77777778 | 9 |
| 3.344086022 | 0.00229098 | 0.29903537 | 0.611111111 | 9 | 6 | PC.ae.C40.2 | 10.88888889 | 9 |
| 3.35483871 | 0.005790593 | 0.298076923 | 0.5 | 8 | 6 | SM.C24.1 | 9 | 8 |
| 2.397849462 | 0.117048086 | 0.417040359 | 0.363636364 | 23 | 6 | PC.ae.C38.6 | 13.43478261 | 23 |
| 2.569892473 | 0.035155082 | 0.389121339 | 0.433823529 | 17 | 6 | PC.ae.C40.1 | 12.76470588 | 17 |
| 2.64516129 | 0.085359985 | 0.37804878 | 0.416666667 | 16 | 6 | SM.OH.C22.2 | 11.6875 | 16 |
| 4.11827957 | 0 | 0.242819843 | 1 | 4 | 8 | Cit | 6.25 | 4 |
| 3.204301075 | 0.001058558 | 0.312080537 | 0.8 | 5 | 7 | PC.aa.C30.0 | 11.4 | 5 |
| 3.182795699 | 0.001058417 | 0.314189189 | 0.761904762 | 7 | 6 | PC.ae.C36.0 | 13.71428571 | 7 |
| 2.258064516 | 0.100664475 | 0.442857143 | 0.414285714 | 21 | 6 | PC.ae.C32.1 | 13.47619048 | 21 |
| 2.677419355 | 0.01862127 | 0.373493976 | 0.5 | 13 | 6 | PC.ae.C36.2 | 14.38461538 | 13 |
| 3.075268817 | 0.00136283 | 0.325174825 | 0.7 | 5 | 6 | SM.OH.C14.1 | 11.8 | 5 |
| 2.946236559 | 0.02196973 | 0.339416058 | 0.590909091 | 12 | 5 | PC.ae.C38.0 | 12.5 | 12 |
| 3.107526882 | 0.009448643 | 0.321799308 | 0.722222222 | 9 | 6 | PC.aa.C36.0 | 13 | 9 |
| 2.935483871 | 0.011277232 | 0.340659341 | 0.466666667 | 6 | 6 | PC.aa.C28.1 | 11.33333333 | 6 |
| 3.107526882 | 0.005806085 | 0.321799308 | 0.523809524 | 7 | 7 | PC.aa.C34.2 | 10.71428571 | 7 |
| 3.333333333 | 6.61E-04 | 0.3 | 0.6 | 5 | 7 | PC.aa.C36.2 | 9.8 | 5 |
| 2.440860215 | 0.051234635 | 0.40969163 | 0.45751634 | 18 | 5 | SM.C16.0 | 13.22222222 | 18 |
| 2.720430108 | 0.045026425 | 0.367588933 | 0.340659341 | 14 | 6 | SM.C16.1 | 11.57142857 | 14 |
| 3.47311828 | 0.037387098 | 0.287925697 | 0.5 | 4 | 6 | PC.aa.C38.6 | 10.75 | 4 |
| 3.849462366 | 0.028835288 | 0.259776536 | 0 | 2 | 6 | PC.aa.C40.6 | 5.5 | 2 |
| 3.161290323 | 0.02499785 | 0.316326531 | 0.484848485 | 12 | 7 | PC.aa.C36.3 | 9.416666667 | 12 |
| 3.11827957 | 0.010648332 | 0.320689655 | 0.392857143 | 8 | 6 | SM.OH.C16.1 | 9 | 8 |
| 2.548387097 | 0.022051382 | 0.392405063 | 0.470588235 | 17 | 6 | PC.ae.C32.2 | 14.29411765 | 17 |
| 5.870967742 | 0 | 0.17032967 | 1 | 2 | 9 | Trp | 3 | 2 |
| 4.892473118 | 0.010635811 | 0.204395604 | 0.666666667 | 3 | 8 | Val | 3 | 3 |
| 3.279569892 | 0.00517764 | 0.304918033 | 0.571428571 | 7 | 6 | SM.C18.0 | 9 | 7 |
| 3.387096774 | 0.002020745 | 0.295238095 | 0.333333333 | 3 | 7 | SM.C18.1 | 9.666666667 | 3 |
| 1.571428571 | 0 | 0.636363636 | 1 | 3 | 2 | C10.1 | 6 | 3 |
| 3.784946237 | 6.86E-05 | 0.264204545 | 0.666666667 | 3 | 6 | SM.C24.0 | 11 | 3 |
| 2.569892473 | 0.078753538 | 0.389121339 | 0.423076923 | 13 | 6 | PC.ae.C34.1 | 13.15384615 | 13 |
| 2.612903226 | 0.174636756 | 0.382716049 | 0.21978022 | 14 | 6 | PC.aa.C34.4 | 8.642857143 | 14 |
| 3.043010753 | 0.012378575 | 0.328621908 | 0.733333333 | 10 | 6 | PC.aa.C36.6 | 13.8 | 10 |
| 4.602150538 | 0.021505376 | 0.21728972 | 0.5 | 4 | 8 | Orn | 5 | 4 |
| 3.870967742 | 0 | 0.258333333 | 1 | 3 | 7 | PC.aa.C42.1 | 7.666666667 | 3 |
| 2.827956989 | 3.31E-04 | 0.353612167 | 0.733333333 | 6 | 6 | PC.ae.C38.2 | 16.5 | 6 |
| 3.129032258 | 0.074822743 | 0.319587629 | 0.380952381 | 7 | 7 | lysoPC.a.C18.2 | 7.714285714 | 7 |
| 2.806451613 | 5.13E-04 | 0.356321839 | 0.785714286 | 8 | 6 | PC.ae.C34.0 | 16.5 | 8 |
| 3.193548387 | 0.06074384 | 0.313131313 | 0.416666667 | 9 | 6 | Ala | 10.77777778 | 9 |
| 3.344086022 | 0.028946402 | 0.29903537 | 0.6 | 6 | 6 | Pro | 10.33333333 | 6 |
| 3.376344086 | 0.016153128 | 0.296178344 | 0 | 3 | 7 | lysoPC.a.C18.0 | 6.333333333 | 3 |
| 3.053763441 | 0.028655735 | 0.327464789 | 0 | 4 | 7 | PC.aa.C36.4 | 8.5 | 4 |
| 3.193548387 | 0.007152371 | 0.313131313 | 0.5 | 4 | 7 | PC.aa.C32.3 | 10 | 4 |
| 3.322580645 | 0.016822511 | 0.300970874 | 0.619047619 | 7 | 7 | PC.aa.C34.3 | 11 | 7 |
| 3.870967742 | 0.004501729 | 0.258333333 | 0.333333333 | 3 | 8 | lysoPC.a.C20.4 | 7 | 3 |
| 3.559139785 | 0 | 0.280966767 | 1 | 2 | 7 | PC.aa.C42.6 | 10.5 | 2 |
| 4.010752688 | 0 | 0.249329759 | 1 | 2 | 7 | PC.aa.C38.1 | 9.5 | 2 |
| 2.827956989 | 0.001050285 | 0.353612167 | 0.857142857 | 7 | 6 | PC.ae.C30.0 | 16.85714286 | 7 |
| 2.720430108 | 0.005228511 | 0.367588933 | 0.611111111 | 9 | 6 | PC.ae.C42.2 | 15.88888889 | 9 |
| 3.086021505 | 7.23E-04 | 0.324041812 | 0.7 | 5 | 7 | PC.ae.C38.1 | 16.2 | 5 |
| 4.043010753 | 0 | 0.247340426 | 0 | 1 | 8 | PC.aa.C38.4 | 4 | 1 |
| 1.571428571 | 0 | 0.636363636 | 1 | 3 | 2 | C18.1 | 6 | 3 |
| 1.571428571 | 0 | 0.636363636 | 1 | 3 | 2 | C14 | 6 | 3 |
| 3.537634409 | 0 | 0.282674772 | 0 | 1 | 7 | PC.aa.C42.5 | 14 | 1 |
| 5.591397849 | 0 | 0.178846154 | 0 | 1 | 9 | Phe | 4 | 1 |
| 1.714285714 | 0 | 0.583333333 | 1 | 2 | 2 | Ser | 6 | 2 |
| 3.35483871 | 0 | 0.298076923 | 1 | 2 | 6 | lysoPC.a.C17.0 | 15 | 2 |

Supplementary Table 14. Correlation coefficient of nodes from advanced lung adenocarcinoma

| Correlation coefficient | Edge Betweenness | Metabolites |
| --- | --- | --- |
| 0.977078621 | 12.3832989 | PC.aa.C34.1 (interacts with) PC.aa.C36.1 |
| 0.919361723 | 16.03029683 | PC.aa.C34.1 (interacts with) PC.aa.C40.5 |
| 0.908177052 | 27.53201258 | PC.aa.C34.1 (interacts with) PC.aa.C40.4 |
| 0.893183254 | 20.74241358 | PC.aa.C34.1 (interacts with) PC.aa.C36.3 |
| 0.853258023 | 28.59356243 | PC.aa.C34.1 (interacts with) PC.aa.C34.3 |
| 0.835330483 | 32.13306829 | PC.aa.C34.1 (interacts with) PC.aa.C38.3 |
| 0.965592701 | 3.053846154 | PC.aa.C36.1 (interacts with) PC.aa.C40.5 |
| 0.930083623 | 28.42738543 | PC.aa.C36.1 (interacts with) PC.aa.C40.4 |
| 0.895207952 | 23.12517345 | PC.aa.C36.1 (interacts with) PC.aa.C38.3 |
| 0.862614938 | 40.729682 | PC.aa.C36.1 (interacts with) PC.aa.C36.3 |
| 0.833627462 | 34.62800428 | PC.aa.C36.1 (interacts with) PC.aa.C38.5 |
| 0.817211153 | 31.39083988 | PC.aa.C36.1 (interacts with) PC.aa.C36.5 |
| 0.973296353 | 6.391025641 | PC.aa.C38.0 (interacts with) PC.ae.C40.6 |
| 0.945310665 | 14.07797666 | PC.aa.C38.0 (interacts with) PC.ae.C40.5 |
| 0.927509534 | 9.511754912 | PC.aa.C38.0 (interacts with) PC.ae.C36.0 |
| 0.90289859 | 81.53941157 | PC.aa.C38.0 (interacts with) PC.ae.C38.6 |
| 0.902203089 | 10.80213675 | PC.aa.C38.0 (interacts with) PC.ae.C38.0 |
| 0.896318838 | 30.04376966 | PC.aa.C38.0 (interacts with) PC.ae.C40.1 |
| 0.881517982 | 21.90083095 | PC.aa.C38.0 (interacts with) SM.OH.C22.2 |
| 0.864539233 | 38.96539885 | PC.aa.C38.0 (interacts with) PC.ae.C40.4 |
| 0.848147272 | 16.10926199 | PC.aa.C38.0 (interacts with) SM.OH.C22.1 |
| 0.845879288 | 19.49853596 | PC.aa.C38.0 (interacts with) PC.ae.C40.2 |
| 0.816805651 | 24.98815086 | PC.aa.C38.0 (interacts with) SM.OH.C24.1 |
| 0.890090216 | 29.34372219 | PC.ae.C40.6 (interacts with) SM.OH.C22.2 |
| 0.836900108 | 13.65156969 | PC.ae.C40.6 (interacts with) SM.OH.C22.1 |
| 0.80250101 | 31.86592864 | PC.ae.C40.6 (interacts with) SM.OH.C24.1 |
| 0.972585605 | 51.22165837 | PC.ae.C34.2 (interacts with) PC.ae.C36.3 |
| 0.931507747 | 64.80850989 | PC.ae.C34.2 (interacts with) PC.ae.C36.4 |
| 0.928825478 | 49.83815802 | PC.ae.C34.2 (interacts with) PC.ae.C34.3 |
| 0.867180732 | 225.7421492 | PC.ae.C34.2 (interacts with) PC.ae.C38.5 |
| 0.850925318 | 133.1443734 | PC.ae.C34.2 (interacts with) PC.ae.C36.5 |
| 0.832339742 | 181.6488605 | PC.ae.C34.2 (interacts with) SM.C16.0 |
| 0.806986231 | 119.2015723 | PC.ae.C34.2 (interacts with) PC.ae.C42.4 |
| 0.956601167 | 27.35489423 | PC.ae.C36.3 (interacts with) PC.ae.C36.4 |
| 0.917418657 | 29.46209099 | PC.ae.C36.3 (interacts with) PC.ae.C38.5 |
| 0.887233306 | 46.7378948 | PC.ae.C36.3 (interacts with) PC.ae.C36.5 |
| 0.8564165 | 30.00335197 | PC.ae.C36.3 (interacts with) PC.ae.C38.3 |
| 0.85399603 | 48.2726724 | PC.ae.C36.3 (interacts with) PC.ae.C42.4 |
| 0.83084929 | 24.48109257 | PC.ae.C36.3 (interacts with) SM.C16.0 |
| 0.813704359 | 59.80102675 | PC.ae.C36.3 (interacts with) PC.ae.C38.4 |
| 0.970135886 | 3 | C12.1 (interacts with) C14.1 |
| 0.954397775 | 3.333333333 | C12.1 (interacts with) C14.2 |
| 0.832041962 | 6.333333333 | C12.1 (interacts with) C18.1 |
| 0.825916312 | 5.666666667 | C12.1 (interacts with) C14 |
| 0.800058264 | 9 | C12.1 (interacts with) Ser |
| 0.959253377 | 4.333333333 | C14.1 (interacts with) C14.2 |
| 0.850642343 | 4.333333333 | C14.1 (interacts with) C18.1 |
| 0.965556551 | 7.945010545 | PC.aa.C32.1 (interacts with) PC.aa.C34.1 |
| 0.940914213 | 9.939287895 | PC.aa.C32.1 (interacts with) PC.aa.C36.1 |
| 0.875269809 | 10.59700286 | PC.aa.C32.1 (interacts with) PC.aa.C40.5 |
| 0.819386758 | 110.8209553 | PC.aa.C32.1 (interacts with) PC.aa.C34.3 |
| 0.95978601 | 21.64575942 | PC.ae.C40.3 (interacts with) PC.ae.C42.5 |
| 0.930882404 | 23.00178694 | PC.ae.C40.3 (interacts with) PC.ae.C42.3 |
| 0.91676241 | 14.33688719 | PC.ae.C40.3 (interacts with) PC.ae.C40.4 |
| 0.862072496 | 37.40800849 | PC.ae.C40.3 (interacts with) PC.ae.C40.5 |
| 0.853165631 | 62.48128966 | PC.ae.C40.3 (interacts with) PC.ae.C44.5 |
| 0.814187029 | 163.5428604 | PC.ae.C40.3 (interacts with) SM.C16.0 |
| 0.855106655 | 95.81183795 | PC.ae.C42.5 (interacts with) PC.ae.C44.5 |
| 0.853077885 | 67.73291206 | PC.ae.C42.5 (interacts with) PC.ae.C44.6 |
| 0.824639534 | 71.53761489 | PC.ae.C42.5 (interacts with) SM.C16.0 |
| 0.811400539 | 5 | C14.2 (interacts with) Ser |
| 0.950470654 | 72.78275303 | PC.ae.C36.4 (interacts with) PC.ae.C38.5 |
| 0.950108906 | 38.16133191 | PC.ae.C36.4 (interacts with) PC.ae.C36.5 |
| 0.893782055 | 56.62349585 | PC.ae.C36.4 (interacts with) PC.ae.C38.4 |
| 0.864070452 | 62.99423442 | PC.ae.C36.4 (interacts with) PC.ae.C38.3 |
| 0.836364993 | 56.53766926 | PC.ae.C36.4 (interacts with) SM.C16.0 |
| 0.954914574 | 21.99238702 | PC.ae.C36.5 (interacts with) PC.ae.C38.5 |
| 0.893152153 | 29.05984767 | PC.ae.C36.5 (interacts with) PC.ae.C38.4 |
| 0.876650383 | 162.4786127 | PC.ae.C36.5 (interacts with) PC.ae.C38.6 |
| 0.840587489 | 25.50612329 | PC.ae.C36.5 (interacts with) PC.ae.C38.3 |
| 0.907256908 | 103.4531947 | PC.ae.C38.5 (interacts with) PC.ae.C38.6 |
| 0.891698656 | 11.59498584 | PC.ae.C38.5 (interacts with) SM.C16.0 |
| 0.848371084 | 159.0381794 | PC.ae.C38.5 (interacts with) PC.ae.C40.4 |
| 0.845857039 | 60.68856374 | PC.ae.C38.5 (interacts with) SM.C16.1 |
| 0.828441817 | 44.78625209 | PC.ae.C38.5 (interacts with) PC.ae.C42.2 |
| 0.819982055 | 53.71529627 | PC.ae.C38.5 (interacts with) PC.ae.C42.5 |
| 0.813708131 | 129.4775625 | PC.ae.C38.5 (interacts with) PC.ae.C40.1 |
| 0.951924721 | 7.506565087 | lysoPC.a.C16.1 (interacts with) lysoPC.a.C20.3 |
| 0.935273587 | 7.268263588 | lysoPC.a.C16.1 (interacts with) PC.aa.C32.1 |
| 0.93247882 | 27.50421203 | lysoPC.a.C16.1 (interacts with) lysoPC.a.C18.1 |
| 0.911766588 | 5.286951567 | lysoPC.a.C16.1 (interacts with) PC.aa.C34.1 |
| 0.858013114 | 17.86372625 | lysoPC.a.C16.1 (interacts with) PC.aa.C36.1 |
| 0.806975456 | 128.8109861 | lysoPC.a.C16.1 (interacts with) PC.aa.C32.0 |
| 0.800362491 | 18.84673558 | lysoPC.a.C16.1 (interacts with) PC.aa.C40.5 |
| 0.920050115 | 7.315662656 | lysoPC.a.C20.3 (interacts with) PC.aa.C34.1 |
| 0.89532352 | 8.567532468 | lysoPC.a.C20.3 (interacts with) PC.aa.C32.1 |
| 0.89525193 | 18.34549498 | lysoPC.a.C20.3 (interacts with) PC.aa.C36.1 |
| 0.853211129 | 65.15507791 | lysoPC.a.C20.3 (interacts with) PC.aa.C36.3 |
| 0.830822933 | 19.89269314 | lysoPC.a.C20.3 (interacts with) PC.aa.C40.5 |
| 0.824569412 | 47.13694028 | lysoPC.a.C20.3 (interacts with) lysoPC.a.C20.4 |
| 0.804740635 | 72.04726823 | lysoPC.a.C20.3 (interacts with) PC.aa.C40.4 |
| 0.948953156 | 3.4 | PC.aa.C36.5 (interacts with) PC.aa.C38.5 |
| 0.876410609 | 39.12839126 | PC.aa.C36.5 (interacts with) PC.aa.C40.5 |
| 0.819419107 | 14.65878812 | PC.aa.C36.5 (interacts with) PC.aa.C40.4 |
| 0.896949748 | 15.56261919 | PC.aa.C38.5 (interacts with) PC.aa.C40.4 |
| 0.896364592 | 42.67374311 | PC.aa.C38.5 (interacts with) PC.aa.C40.5 |
| 0.871236478 | 15.72380952 | PC.aa.C38.5 (interacts with) PC.aa.C42.6 |
| 0.948620631 | 80.107556 | PC.aa.C42.0 (interacts with) PC.ae.C42.4 |
| 0.940363559 | 2.666666667 | PC.aa.C42.0 (interacts with) PC.ae.C44.6 |
| 0.882776398 | 61.73284333 | PC.aa.C42.0 (interacts with) PC.ae.C42.5 |
| 0.832093746 | 45.5086734 | PC.aa.C42.0 (interacts with) PC.ae.C42.3 |
| 0.818307331 | 10.31267962 | PC.aa.C42.0 (interacts with) PC.ae.C44.5 |
| 0.870434674 | 126.3677366 | PC.ae.C42.4 (interacts with) PC.ae.C44.6 |
| 0.850143013 | 55.01344177 | PC.ae.C42.4 (interacts with) PC.ae.C42.5 |
| 0.945603699 | 17.1958917 | PC.ae.C38.3 (interacts with) PC.ae.C38.4 |
| 0.936035336 | 6.438095238 | PC.ae.C38.3 (interacts with) PC.ae.C38.5 |
| 0.908657986 | 45.25436457 | PC.ae.C38.3 (interacts with) SM.C16.1 |
| 0.902835697 | 10.04711418 | PC.ae.C38.3 (interacts with) SM.C16.0 |
| 0.860163814 | 39.02767594 | PC.ae.C38.3 (interacts with) PC.ae.C42.5 |
| 0.859806077 | 88.37660674 | PC.ae.C38.3 (interacts with) PC.ae.C40.3 |
| 0.844606483 | 75.92851654 | PC.ae.C38.3 (interacts with) PC.ae.C38.6 |
| 0.840739864 | 91.72011984 | PC.ae.C38.3 (interacts with) PC.ae.C40.4 |
| 0.943473722 | 26.26702253 | PC.ae.C38.4 (interacts with) PC.ae.C38.5 |
| 0.865561791 | 62.7844489 | PC.ae.C38.4 (interacts with) SM.C16.1 |
| 0.836139216 | 39.84047149 | PC.ae.C38.4 (interacts with) SM.C16.0 |
| 0.831404442 | 139.2648077 | PC.ae.C38.4 (interacts with) PC.ae.C38.6 |
| 0.800212452 | 152.4471278 | PC.ae.C38.4 (interacts with) PC.ae.C40.4 |
| 0.935593036 | 29.96862523 | PC.ae.C40.5 (interacts with) PC.ae.C40.6 |
| 0.867918491 | 68.31510595 | PC.ae.C40.5 (interacts with) PC.ae.C42.3 |
| 0.83752422 | 97.56718446 | PC.ae.C40.5 (interacts with) PC.ae.C42.5 |
| 0.833861769 | 29.56437898 | PC.ae.C40.5 (interacts with) PC.ae.C42.2 |
| 0.821885687 | 55.82697693 | PC.ae.C40.5 (interacts with) SM.OH.C22.2 |
| 0.943871297 | 9.646012948 | PC.ae.C44.5 (interacts with) PC.ae.C44.6 |
| 0.939449203 | 7.76070831 | PC.ae.C34.3 (interacts with) PC.ae.C36.4 |
| 0.930538904 | 16.50408417 | PC.ae.C34.3 (interacts with) PC.ae.C36.5 |
| 0.925454426 | 17.92509945 | PC.ae.C34.3 (interacts with) PC.ae.C36.3 |
| 0.906546227 | 30.29361367 | PC.ae.C34.3 (interacts with) PC.ae.C38.5 |
| 0.879586153 | 27.87097133 | PC.ae.C34.3 (interacts with) PC.ae.C36.2 |
| 0.836490626 | 33.49412682 | PC.ae.C34.3 (interacts with) PC.ae.C38.4 |
| 0.835371207 | 19.84900481 | PC.ae.C34.3 (interacts with) SM.C16.0 |
| 0.802099231 | 28.17817539 | PC.ae.C34.3 (interacts with) PC.ae.C38.3 |
| 0.939416823 | 24.13319034 | PC.ae.C42.3 (interacts with) PC.ae.C42.5 |
| 0.85866579 | 28.43387608 | PC.ae.C42.3 (interacts with) PC.ae.C44.5 |
| 0.849046173 | 44.84200674 | PC.ae.C42.3 (interacts with) PC.ae.C44.6 |
| 0.939371556 | 2 | C4 (interacts with) Creatinine |
| 0.914907222 | 11.4962871 | C4 (interacts with) Cit |
| 0.911899894 | 28.61948578 | C4 (interacts with) total.DMA |
| 0.868874585 | 89.67334626 | C4 (interacts with) Orn |
| 0.861279457 | 83.57446441 | C4 (interacts with) t4.OH.Pro |
| 0.821195147 | 158.3090165 | C4 (interacts with) lysoPC.a.C18.2 |
| 0.937752749 | 28.61948578 | Creatinine (interacts with) total.DMA |
| 0.935762878 | 83.57446441 | Creatinine (interacts with) t4.OH.Pro |
| 0.882230728 | 158.3090165 | Creatinine (interacts with) lysoPC.a.C18.2 |
| 0.939295995 | 21.75337513 | PC.ae.C36.1 (interacts with) PC.ae.C38.3 |
| 0.872080017 | 63.46060618 | PC.ae.C36.1 (interacts with) SM.C16.1 |
| 0.865735238 | 43.78641668 | PC.ae.C36.1 (interacts with) PC.ae.C38.4 |
| 0.863354468 | 26.97578852 | PC.ae.C36.1 (interacts with) PC.ae.C38.5 |
| 0.844179165 | 139.7796372 | PC.ae.C36.1 (interacts with) PC.ae.C38.6 |
| 0.807986076 | 163.4335548 | PC.ae.C36.1 (interacts with) PC.ae.C40.1 |
| 0.802517507 | 548.3526325 | PC.ae.C36.1 (interacts with) SM.OH.C22.2 |
| 0.802313264 | 40.30100914 | PC.ae.C36.1 (interacts with) SM.C16.0 |
| 0.939043382 | 6.435236985 | lysoPC.a.C16.0 (interacts with) lysoPC.a.C18.1 |
| 0.920131952 | 29.27053196 | lysoPC.a.C16.0 (interacts with) lysoPC.a.C20.3 |
| 0.879867359 | 26.22468965 | lysoPC.a.C16.0 (interacts with) lysoPC.a.C16.1 |
| 0.872082508 | 57.28302071 | lysoPC.a.C16.0 (interacts with) lysoPC.a.C20.4 |
| 0.86901176 | 72.63812981 | lysoPC.a.C16.0 (interacts with) PC.aa.C36.3 |
| 0.839299363 | 38.70305416 | lysoPC.a.C16.0 (interacts with) PC.aa.C34.1 |
| 0.832651206 | 211.3087749 | lysoPC.a.C16.0 (interacts with) lysoPC.a.C18.2 |
| 0.830613011 | 88.50319116 | lysoPC.a.C16.0 (interacts with) PC.aa.C32.0 |
| 0.930177993 | 37.58438695 | lysoPC.a.C18.1 (interacts with) lysoPC.a.C20.3 |
| 0.894670504 | 47.60569882 | lysoPC.a.C18.1 (interacts with) PC.aa.C32.0 |
| 0.877832412 | 52.77966856 | lysoPC.a.C18.1 (interacts with) PC.aa.C36.3 |
| 0.872981535 | 36.4571209 | lysoPC.a.C18.1 (interacts with) PC.aa.C34.1 |
| 0.849731244 | 228.608778 | lysoPC.a.C18.1 (interacts with) lysoPC.a.C18.2 |
| 0.822210702 | 57.93451475 | lysoPC.a.C18.1 (interacts with) PC.aa.C32.1 |
| 0.80719881 | 143.7182381 | lysoPC.a.C18.1 (interacts with) PC.aa.C32.2 |
| 0.938360545 | 5.29047619 | PC.aa.C38.3 (interacts with) PC.aa.C40.4 |
| 0.894395374 | 33.54372966 | PC.aa.C38.3 (interacts with) PC.aa.C40.5 |
| 0.886111754 | 6.754609116 | PC.aa.C38.3 (interacts with) PC.aa.C38.5 |
| 0.923223397 | 47.63315162 | PC.aa.C40.4 (interacts with) PC.aa.C40.5 |
| 0.889432593 | 218.0300379 | total.DMA (interacts with) lysoPC.a.C18.2 |
| -0.814830599 | 326.1998526 | total.DMA (interacts with) PC.aa.C40.6 |
| 0.937073011 | 14.32552236 | SM.OH.C22.1 (interacts with) SM.OH.C24.1 |
| 0.933248369 | 32.6123354 | SM.OH.C22.1 (interacts with) SM.OH.C22.2 |
| 0.902887099 | 70.76412099 | SM.OH.C22.1 (interacts with) SM.C24.0 |
| 0.866145464 | 40.3023846 | SM.OH.C22.1 (interacts with) SM.C24.1 |
| 0.82638703 | 41.43941146 | SM.OH.C22.1 (interacts with) SM.C18.0 |
| 0.804939751 | 8.859464712 | SM.OH.C24.1 (interacts with) SM.C24.1 |
| 0.8003797 | 11.53086038 | SM.OH.C24.1 (interacts with) SM.C18.0 |
| 0.936683798 | 273 | Ile (interacts with) Leu |
| 0.880444543 | 253.9568374 | Ile (interacts with) Pro |
| 0.830826847 | 273 | Ile (interacts with) Val |
| 0.904703377 | 2 | Leu (interacts with) Val |
| 0.855284278 | 93 | Leu (interacts with) Trp |
| 0.935992398 | 14.25510119 | PC.ae.C40.4 (interacts with) PC.ae.C40.5 |
| 0.931065681 | 42.66089008 | PC.ae.C40.4 (interacts with) PC.ae.C42.5 |
| 0.920302967 | 48.41229036 | PC.ae.C40.4 (interacts with) PC.ae.C42.3 |
| 0.826555872 | 66.18942788 | PC.ae.C40.4 (interacts with) PC.ae.C40.6 |
| 0.814487744 | 16.18471803 | PC.ae.C40.4 (interacts with) PC.ae.C42.2 |
| 0.908118878 | 91.13570015 | t4.OH.Pro (interacts with) total.DMA |
| 0.879506097 | 38.66854817 | t4.OH.Pro (interacts with) lysoPC.a.C18.2 |
| 0.817175064 | 490.1678252 | t4.OH.Pro (interacts with) PC.ae.C34.2 |
| 0.935703414 | 3.666666667 | C2 (interacts with) C12.1 |
| 0.903094514 | 3.333333333 | C2 (interacts with) C10.1 |
| 0.901627909 | 2.666666667 | C2 (interacts with) C14.1 |
| 0.858520221 | 5 | C2 (interacts with) C14.2 |
| 0.850016008 | 3.333333333 | C2 (interacts with) C18.1 |
| 0.934677362 | 17.7483895 | PC.aa.C32.0 (interacts with) PC.aa.C32.2 |
| 0.89998213 | 13.39035689 | PC.aa.C32.0 (interacts with) PC.aa.C36.3 |
| 0.840685525 | 77.42221479 | PC.aa.C32.0 (interacts with) PC.aa.C34.1 |
| 0.818948917 | 410.0513758 | PC.aa.C32.0 (interacts with) PC.ae.C34.1 |
| 0.804564335 | 28.69936255 | PC.aa.C32.0 (interacts with) PC.aa.C40.4 |
| 0.89920501 | 41.14769748 | PC.aa.C32.2 (interacts with) PC.aa.C36.3 |
| 0.879479698 | 52.44767729 | PC.aa.C32.2 (interacts with) PC.aa.C34.4 |
| 0.837703057 | 52.75068258 | PC.aa.C32.2 (interacts with) PC.aa.C34.3 |
| 0.824870187 | 96.02319454 | PC.aa.C32.2 (interacts with) PC.ae.C34.1 |
| 0.81926483 | 250.3612383 | PC.aa.C32.2 (interacts with) PC.ae.C36.3 |
| 0.812900664 | 60.95555927 | PC.aa.C32.2 (interacts with) PC.aa.C40.4 |
| 0.934003771 | 7.240234645 | PC.ae.C40.2 (interacts with) SM.C24.1 |
| 0.871811835 | 27.08001744 | PC.ae.C40.2 (interacts with) PC.ae.C40.6 |
| 0.848299448 | 78.09366234 | PC.ae.C40.2 (interacts with) SM.OH.C22.2 |
| 0.817991892 | 26.4823553 | PC.ae.C40.2 (interacts with) SM.OH.C22.1 |
| 0.812738561 | 9.60318875 | PC.ae.C40.2 (interacts with) SM.OH.C24.1 |
| 0.93348618 | 8.622786364 | PC.ae.C38.6 (interacts with) PC.ae.C40.1 |
| 0.920016097 | 45.79621165 | PC.ae.C38.6 (interacts with) PC.ae.C40.5 |
| 0.887652053 | 21.04298785 | PC.ae.C38.6 (interacts with) PC.ae.C40.4 |
| 0.861022928 | 22.25312792 | PC.ae.C38.6 (interacts with) PC.ae.C42.2 |
| 0.843295151 | 124.8114015 | PC.ae.C38.6 (interacts with) PC.ae.C40.6 |
| 0.832660547 | 204.9364028 | PC.ae.C38.6 (interacts with) SM.OH.C22.1 |
| 0.815942243 | 91.83279442 | PC.ae.C38.6 (interacts with) SM.OH.C22.2 |
| 0.800193082 | 132.4826617 | PC.ae.C38.6 (interacts with) PC.ae.C42.3 |
| 0.896365988 | 20.77523119 | PC.ae.C40.1 (interacts with) PC.ae.C40.5 |
| 0.853332194 | 47.20011181 | PC.ae.C40.1 (interacts with) PC.ae.C40.6 |
| 0.842029637 | 16.46263065 | PC.ae.C40.1 (interacts with) PC.ae.C40.4 |
| 0.820286146 | 40.22207079 | PC.ae.C40.1 (interacts with) SM.OH.C22.2 |
| 0.811300853 | 16.24611434 | PC.ae.C40.1 (interacts with) PC.ae.C42.2 |
| 0.891365623 | 96.59111919 | SM.OH.C22.2 (interacts with) SM.C24.1 |
| 0.856150392 | 62.73957294 | SM.OH.C22.2 (interacts with) SM.OH.C24.1 |
| 0.808027888 | 78.57779977 | SM.OH.C22.2 (interacts with) SM.C18.0 |
| 0.932875307 | 11.4962871 | Cit (interacts with) Creatinine |
| 0.898886241 | 121.0535962 | Cit (interacts with) t4.OH.Pro |
| 0.891122094 | 41.95382956 | Cit (interacts with) total.DMA |
| 0.928346455 | 17.49118511 | PC.aa.C30.0 (interacts with) PC.aa.C32.0 |
| 0.901478684 | 12.05951069 | PC.aa.C30.0 (interacts with) PC.aa.C32.2 |
| 0.896308569 | 100.221245 | PC.aa.C30.0 (interacts with) PC.ae.C34.1 |
| 0.853967589 | 47.13271146 | PC.aa.C30.0 (interacts with) PC.aa.C34.4 |
| 0.82021401 | 27.20939489 | PC.aa.C30.0 (interacts with) PC.aa.C36.3 |
| 0.910923997 | 23.12127872 | PC.ae.C36.0 (interacts with) PC.ae.C40.6 |
| 0.887309715 | 18.79650209 | PC.ae.C36.0 (interacts with) PC.ae.C40.5 |
| 0.84457751 | 20.69405676 | PC.ae.C36.0 (interacts with) PC.ae.C40.2 |
| 0.815909177 | 64.70072538 | PC.ae.C36.0 (interacts with) PC.ae.C40.1 |
| 0.807041535 | 59.77128913 | PC.ae.C36.0 (interacts with) PC.ae.C40.4 |
| 0.925379953 | 12.09823528 | PC.ae.C32.1 (interacts with) PC.ae.C38.5 |
| 0.920385884 | 18.38214528 | PC.ae.C32.1 (interacts with) PC.ae.C36.3 |
| 0.906732407 | 37.66491875 | PC.ae.C32.1 (interacts with) PC.ae.C32.2 |
| 0.898536069 | 19.02597967 | PC.ae.C32.1 (interacts with) PC.ae.C38.3 |
| 0.889571421 | 64.35366068 | PC.ae.C32.1 (interacts with) PC.ae.C34.1 |
| 0.885344093 | 67.52127595 | PC.ae.C32.1 (interacts with) PC.ae.C36.4 |
| 0.884640255 | 63.20969709 | PC.ae.C32.1 (interacts with) PC.ae.C36.5 |
| 0.869736523 | 24.33404925 | PC.ae.C32.1 (interacts with) PC.ae.C36.1 |
| 0.866866435 | 181.3532516 | PC.ae.C32.1 (interacts with) PC.ae.C34.2 |
| 0.866418139 | 234.9684692 | PC.ae.C32.1 (interacts with) PC.ae.C38.6 |
| 0.852097132 | 25.1974731 | PC.ae.C32.1 (interacts with) SM.C16.0 |
| 0.851509892 | 41.53619175 | PC.ae.C32.1 (interacts with) PC.ae.C34.0 |
| 0.842487978 | 92.40688015 | PC.ae.C32.1 (interacts with) PC.ae.C42.2 |
| 0.839783837 | 38.78424862 | PC.ae.C32.1 (interacts with) PC.ae.C34.3 |
| 0.836405543 | 74.4061439 | PC.ae.C32.1 (interacts with) PC.ae.C42.4 |
| 0.834368818 | 85.81942111 | PC.ae.C32.1 (interacts with) PC.ae.C38.2 |
| 0.823144076 | 48.33655922 | PC.ae.C32.1 (interacts with) PC.ae.C38.4 |
| 0.807093162 | 66.83014053 | PC.ae.C32.1 (interacts with) SM.C16.1 |
| 0.803700402 | 137.0021861 | PC.ae.C32.1 (interacts with) PC.ae.C42.5 |
| 0.923255452 | 30.8743225 | PC.ae.C36.2 (interacts with) SM.OH.C14.1 |
| 0.888514059 | 23.14355249 | PC.ae.C36.2 (interacts with) SM.C16.0 |
| 0.869694205 | 17.1441655 | PC.ae.C36.2 (interacts with) PC.ae.C38.3 |
| 0.867590517 | 24.61515562 | PC.ae.C36.2 (interacts with) PC.ae.C38.5 |
| 0.85330949 | 28.45874546 | PC.ae.C36.2 (interacts with) SM.C16.1 |
| 0.84485803 | 28.31965967 | PC.ae.C36.2 (interacts with) PC.ae.C38.4 |
| 0.82498378 | 111.3051887 | PC.ae.C36.2 (interacts with) SM.OH.C16.1 |
| 0.822577414 | 21.16839077 | PC.ae.C36.2 (interacts with) PC.ae.C38.2 |
| 0.812284937 | 67.60692196 | PC.ae.C36.2 (interacts with) PC.ae.C38.6 |
| 0.811271544 | 30.25593891 | PC.ae.C36.2 (interacts with) PC.ae.C36.5 |
| 0.801415372 | 71.29866395 | PC.ae.C36.2 (interacts with) PC.ae.C36.3 |
| 0.90884075 | 38.68663643 | SM.OH.C14.1 (interacts with) SM.OH.C16.1 |
| 0.899385479 | 85.88971471 | SM.OH.C14.1 (interacts with) SM.C16.0 |
| 0.846922387 | 41.18825578 | SM.OH.C14.1 (interacts with) SM.C16.1 |
| 0.923165317 | 7.072853958 | PC.ae.C38.0 (interacts with) SM.OH.C22.1 |
| 0.921673632 | 35.86812691 | PC.ae.C38.0 (interacts with) SM.OH.C22.2 |
| 0.910995647 | 9.511111111 | PC.ae.C38.0 (interacts with) PC.ae.C40.6 |
| 0.871716888 | 53.56581028 | PC.ae.C38.0 (interacts with) PC.ae.C40.1 |
| 0.846497705 | 23.37922054 | PC.ae.C38.0 (interacts with) SM.OH.C24.1 |
| 0.838759366 | 145.4695898 | PC.ae.C38.0 (interacts with) PC.ae.C38.6 |
| 0.835193277 | 45.71449761 | PC.ae.C38.0 (interacts with) PC.ae.C40.5 |
| 0.820132994 | 89.73818125 | PC.ae.C38.0 (interacts with) SM.C24.0 |
| 0.92098185 | 7.516027451 | PC.aa.C36.0 (interacts with) PC.ae.C36.0 |
| 0.917638498 | 11.76199151 | PC.aa.C36.0 (interacts with) PC.aa.C38.0 |
| 0.882598967 | 25.48215927 | PC.aa.C36.0 (interacts with) PC.ae.C40.6 |
| 0.867866533 | 80.88104713 | PC.aa.C36.0 (interacts with) PC.aa.C38.1 |
| 0.83806005 | 43.87134163 | PC.aa.C36.0 (interacts with) PC.ae.C40.1 |
| 0.83497102 | 4.267136487 | PC.aa.C36.0 (interacts with) PC.aa.C36.6 |
| 0.832254239 | 128.4728506 | PC.aa.C36.0 (interacts with) PC.ae.C38.6 |
| 0.82509011 | 27.74745894 | PC.aa.C36.0 (interacts with) PC.ae.C40.5 |
| 0.805307985 | 17.6851582 | PC.aa.C36.0 (interacts with) PC.ae.C38.0 |
| 0.918352351 | 12.68181818 | PC.aa.C28.1 (interacts with) SM.OH.C14.1 |
| 0.894279823 | 16.79370713 | PC.aa.C28.1 (interacts with) SM.OH.C16.1 |
| 0.86918787 | 130.5856472 | PC.aa.C28.1 (interacts with) SM.C16.0 |
| 0.852993761 | 77.01143876 | PC.aa.C28.1 (interacts with) SM.C16.1 |
| 0.8280801 | 87.07291678 | PC.aa.C28.1 (interacts with) SM.OH.C22.2 |
| 0.810733622 | 54.83047441 | PC.aa.C28.1 (interacts with) SM.C18.0 |
| 0.913348828 | 11.66341991 | PC.aa.C34.2 (interacts with) PC.aa.C36.2 |
| 0.842521224 | 75.73623225 | PC.aa.C34.2 (interacts with) PC.ae.C34.2 |
| 0.841059006 | 24.46804283 | PC.aa.C34.2 (interacts with) PC.ae.C36.4 |
| 0.82793901 | 50.84076872 | PC.aa.C34.2 (interacts with) PC.ae.C36.3 |
| 0.820481963 | 19.5817479 | PC.aa.C34.2 (interacts with) PC.ae.C34.3 |
| 0.806928499 | 53.70697176 | PC.aa.C34.2 (interacts with) SM.C16.1 |
| 0.832269571 | 41.76533351 | PC.aa.C36.2 (interacts with) PC.ae.C34.3 |
| 0.803910298 | 46.39083086 | PC.aa.C36.2 (interacts with) PC.ae.C36.4 |
| 0.800418406 | 63.95660619 | PC.aa.C36.2 (interacts with) SM.C16.1 |
| 0.91168362 | 36.15267244 | SM.C16.0 (interacts with) SM.C16.1 |
| 0.840325088 | 147.1550664 | SM.C16.1 (interacts with) SM.C18.1 |
| 0.911290625 | 353.229589 | PC.aa.C38.6 (interacts with) PC.aa.C40.6 |
| 0.894725455 | 111.4956619 | PC.aa.C38.6 (interacts with) PC.ae.C38.0 |
| 0.840929562 | 222.3864078 | PC.aa.C38.6 (interacts with) PC.ae.C40.6 |
| 0.815196575 | 138.6563684 | PC.aa.C38.6 (interacts with) SM.OH.C22.1 |
| 0.910027951 | 11.26244283 | PC.aa.C36.3 (interacts with) PC.aa.C40.4 |
| 0.816026218 | 19.02351766 | PC.aa.C36.3 (interacts with) PC.aa.C38.3 |
| 0.892862273 | 32.37290454 | SM.OH.C16.1 (interacts with) SM.OH.C24.1 |
| 0.889729722 | 72.84285463 | SM.OH.C16.1 (interacts with) SM.OH.C22.2 |
| 0.869192892 | 12.88551008 | SM.OH.C16.1 (interacts with) SM.C18.0 |
| 0.852914541 | 53.50299026 | SM.OH.C16.1 (interacts with) SM.OH.C22.1 |
| 0.829723755 | 29.82446055 | SM.OH.C16.1 (interacts with) SM.C18.1 |
| 0.89072549 | 31.72698812 | PC.ae.C32.2 (interacts with) PC.ae.C38.6 |
| 0.8897631 | 17.72063492 | PC.ae.C32.2 (interacts with) PC.ae.C38.2 |
| 0.871330416 | 9.78816399 | PC.ae.C32.2 (interacts with) PC.ae.C38.3 |
| 0.870338141 | 13.34831992 | PC.ae.C32.2 (interacts with) PC.ae.C38.5 |
| 0.855802131 | 14.4840612 | PC.ae.C32.2 (interacts with) PC.ae.C34.0 |
| 0.855512994 | 24.24171845 | PC.ae.C32.2 (interacts with) SM.C16.0 |
| 0.851155493 | 22.58549634 | PC.ae.C32.2 (interacts with) PC.ae.C36.2 |
| 0.848163676 | 35.09421193 | PC.ae.C32.2 (interacts with) PC.ae.C40.1 |
| 0.845999614 | 40.83653751 | PC.ae.C32.2 (interacts with) PC.ae.C40.3 |
| 0.837207509 | 36.58524382 | PC.ae.C32.2 (interacts with) PC.ae.C36.1 |
| 0.837025325 | 38.14370566 | PC.ae.C32.2 (interacts with) PC.ae.C40.4 |
| 0.830649475 | 80.16152873 | PC.ae.C32.2 (interacts with) PC.ae.C42.3 |
| 0.822772259 | 66.98312162 | PC.ae.C32.2 (interacts with) PC.ae.C36.3 |
| 0.812512935 | 34.78998121 | PC.ae.C32.2 (interacts with) PC.ae.C36.5 |
| 0.811668079 | 19.82161777 | PC.ae.C32.2 (interacts with) PC.ae.C42.5 |
| 0.906730231 | 93 | Trp (interacts with) Val |
| 0.906196002 | 43.59946869 | SM.C18.0 (interacts with) SM.C18.1 |
| 0.800050574 | 31.73624604 | SM.C18.0 (interacts with) SM.C24.1 |
| 0.88835515 | 6.333333333 | C10.1 (interacts with) C12.1 |
| 0.828545241 | 4.333333333 | C10.1 (interacts with) C14.1 |
| 0.832833311 | 26.6716108 | SM.C24.0 (interacts with) SM.C24.1 |
| 0.900669782 | 135.7998191 | PC.ae.C34.1 (interacts with) PC.ae.C36.1 |
| 0.889254554 | 116.8062366 | PC.ae.C34.1 (interacts with) PC.ae.C38.3 |
| 0.867572525 | 116.0078214 | PC.ae.C34.1 (interacts with) PC.ae.C42.4 |
| 0.866061186 | 28.79362978 | PC.ae.C34.1 (interacts with) PC.ae.C36.3 |
| 0.841154235 | 108.7953125 | PC.ae.C34.1 (interacts with) SM.C16.1 |
| 0.819459405 | 121.3137854 | PC.ae.C34.1 (interacts with) PC.ae.C38.5 |
| 0.814251676 | 96.43090623 | PC.ae.C34.1 (interacts with) SM.C16.0 |
| 0.895344072 | 189.2587573 | PC.aa.C34.4 (interacts with) PC.aa.C38.5 |
| 0.878249987 | 213.481521 | PC.aa.C34.4 (interacts with) PC.aa.C40.4 |
| 0.845533504 | 237.2828624 | PC.aa.C34.4 (interacts with) PC.aa.C36.3 |
| 0.843769721 | 170.2761905 | PC.aa.C34.4 (interacts with) PC.aa.C42.6 |
| 0.838584131 | 191.1759042 | PC.aa.C34.4 (interacts with) PC.aa.C36.5 |
| 0.834695546 | 196.136577 | PC.aa.C34.4 (interacts with) PC.aa.C38.3 |
| 0.826097437 | 102.9360165 | PC.aa.C34.4 (interacts with) PC.ae.C34.1 |
| 0.824951237 | 723.8790939 | PC.aa.C34.4 (interacts with) PC.ae.C36.1 |
| 0.81574965 | 145.4215683 | PC.aa.C34.4 (interacts with) PC.aa.C36.4 |
| 0.809788158 | 546.6903438 | PC.aa.C34.4 (interacts with) PC.ae.C32.1 |
| 0.894476688 | 11.64367299 | PC.aa.C36.6 (interacts with) PC.ae.C38.0 |
| 0.860908687 | 19.38898324 | PC.aa.C36.6 (interacts with) PC.ae.C40.6 |
| 0.852923669 | 40.69479391 | PC.aa.C36.6 (interacts with) SM.OH.C22.2 |
| 0.852062767 | 105.1189529 | PC.aa.C36.6 (interacts with) PC.aa.C38.1 |
| 0.835217548 | 39.99568132 | PC.aa.C36.6 (interacts with) PC.ae.C40.1 |
| 0.833085949 | 8.906421356 | PC.aa.C36.6 (interacts with) PC.aa.C38.0 |
| 0.807132599 | 27.25193418 | PC.aa.C36.6 (interacts with) PC.ae.C40.5 |
| 0.805811165 | 24.10224124 | PC.aa.C36.6 (interacts with) SM.OH.C22.1 |
| 0.805112961 | 116.4523611 | PC.aa.C36.6 (interacts with) PC.ae.C38.6 |
| 0.894171317 | 188.6533075 | Orn (interacts with) total.DMA |
| 0.893106489 | 89.67334626 | Orn (interacts with) Creatinine |
| 0.81477147 | 186 | Orn (interacts with) Phe |
| 0.891449767 | 14.72413781 | PC.aa.C42.1 (interacts with) PC.ae.C44.5 |
| 0.865611147 | 116.3489462 | PC.aa.C42.1 (interacts with) PC.ae.C42.3 |
| 0.846014241 | 54.92691599 | PC.aa.C42.1 (interacts with) PC.ae.C44.6 |
| 0.876176722 | 27.13426378 | PC.ae.C38.2 (interacts with) PC.ae.C40.1 |
| 0.845029728 | 35.25079365 | PC.ae.C38.2 (interacts with) PC.ae.C38.6 |
| 0.80299532 | 453.1326112 | lysoPC.a.C18.2 (interacts with) PC.ae.C34.2 |
| 0.887709933 | 31.55425685 | PC.ae.C34.0 (interacts with) PC.ae.C38.6 |
| 0.863952646 | 29.09674264 | PC.ae.C34.0 (interacts with) PC.ae.C36.1 |
| 0.858701507 | 4.572222222 | PC.ae.C34.0 (interacts with) PC.ae.C38.2 |
| 0.844736636 | 25.60499305 | PC.ae.C34.0 (interacts with) PC.ae.C40.1 |
| 0.820851767 | 22.06362135 | PC.ae.C34.0 (interacts with) PC.ae.C36.5 |
| 0.800761085 | 25.8639865 | PC.ae.C34.0 (interacts with) PC.ae.C38.5 |
| 0.887049013 | 21.69859692 | Ala (interacts with) Pro |
| 0.87964951 | 466.0431626 | Ala (interacts with) Ile |
| -0.810183173 | 77.18294585 | Ala (interacts with) PC.ae.C40.5 |
| -0.81374147 | 51.9339363 | Ala (interacts with) SM.C24.1 |
| -0.821641978 | 24.86833507 | Ala (interacts with) PC.ae.C40.2 |
| -0.83362057 | 72.28163209 | Ala (interacts with) PC.ae.C40.6 |
| -0.840698267 | 209.4111574 | Ala (interacts with) PC.ae.C40.3 |
| -0.849264321 | 262.6632194 | Ala (interacts with) PC.ae.C40.4 |
| -0.849592146 | 39.36560645 | Ala (interacts with) PC.aa.C38.0 |
| -0.828636182 | 98.17106205 | Pro (interacts with) PC.ae.C40.6 |
| -0.849686705 | 274.1078518 | Pro (interacts with) SM.OH.C22.2 |
| -0.85151883 | 21.75362319 | Pro (interacts with) SM.C24.1 |
| -0.852067407 | 11.64285714 | Pro (interacts with) PC.ae.C40.2 |
| 0.883468572 | 62.96117547 | lysoPC.a.C18.0 (interacts with) PC.aa.C36.4 |
| 0.856088407 | 158.6136265 | lysoPC.a.C18.0 (interacts with) lysoPC.a.C20.4 |
| 0.804290639 | 240.8375326 | lysoPC.a.C18.0 (interacts with) PC.ae.C36.4 |
| 0.874751961 | 281.9741879 | PC.aa.C36.4 (interacts with) PC.ae.C38.4 |
| 0.854337755 | 186 | PC.aa.C36.4 (interacts with) PC.aa.C38.4 |
| 0.878301404 | 101.0489946 | PC.aa.C32.3 (interacts with) SM.C16.1 |
| 0.849467609 | 49.35654623 | PC.aa.C32.3 (interacts with) PC.aa.C34.2 |
| 0.832670729 | 33.53333333 | PC.aa.C32.3 (interacts with) PC.aa.C36.2 |
| 0.815195078 | 124.4524985 | PC.aa.C32.3 (interacts with) PC.aa.C34.4 |
| 0.875914214 | 12.4019647 | PC.aa.C34.3 (interacts with) PC.aa.C36.3 |
| 0.875795863 | 29.66815294 | PC.aa.C34.3 (interacts with) PC.aa.C36.1 |
| 0.837655098 | 5.819047619 | PC.aa.C34.3 (interacts with) PC.aa.C40.4 |
| 0.813093957 | 233.812441 | PC.aa.C34.3 (interacts with) PC.aa.C34.4 |
| 0.86103035 | 28.64922597 | PC.ae.C30.0 (interacts with) PC.ae.C32.1 |
| 0.841275966 | 21.70471064 | PC.ae.C30.0 (interacts with) SM.C16.0 |
| 0.83890121 | 36.09753764 | PC.ae.C30.0 (interacts with) PC.ae.C34.1 |
| 0.836612214 | 34.70984586 | PC.ae.C30.0 (interacts with) PC.ae.C34.2 |
| 0.822097579 | 13.72859322 | PC.ae.C30.0 (interacts with) PC.ae.C36.3 |
| 0.8185804 | 39.36700494 | PC.ae.C30.0 (interacts with) PC.ae.C32.2 |
| 0.804748343 | 29.71556543 | PC.ae.C30.0 (interacts with) PC.ae.C38.5 |
| 0.844677133 | 23.88561721 | PC.ae.C42.2 (interacts with) PC.ae.C42.3 |
| 0.806124094 | 12.30958845 | PC.ae.C42.2 (interacts with) PC.ae.C42.5 |
| 0.855372064 | 17.83359939 | PC.ae.C38.1 (interacts with) PC.ae.C42.2 |
| 0.823984269 | 25.12289767 | PC.ae.C38.1 (interacts with) PC.ae.C40.1 |
| 0.819614361 | 77.16995165 | PC.ae.C38.1 (interacts with) PC.ae.C38.4 |
| 0.816534204 | 43.20206293 | PC.ae.C38.1 (interacts with) PC.ae.C38.6 |
| 0.806102719 | 35.05062416 | PC.ae.C38.1 (interacts with) PC.ae.C40.5 |
| 0.845428514 | 3.666666667 | C14 (interacts with) C14.2 |
| 0.808529524 | 4.666666667 | C14 (interacts with) C14.1 |
| 0.822097385 | 186 | PC.aa.C42.5 (interacts with) PC.ae.C36.5 |
| 0.809764189 | 79.76552106 | lysoPC.a.C17.0 (interacts with) PC.ae.C36.5 |
| 0.809511032 | 106.2344789 | lysoPC.a.C17.0 (interacts with) PC.ae.C38.4 |
